# Direct, stereoselective thioglycosylation enabled by an organophotoredox radical strategy†

**DOI:** 10.1039/d0sc04136j

**Published:** 2020-10-19

**Authors:** Peng Ji, Yueteng Zhang, Feng Gao, Fangchao Bi, Wei Wang

**Affiliations:** Departments of Pharmacology and Toxicology and Chemistry and Biochemistry, BIO5 Institute, and University of Arizona Cancer Centre, University of Arizona Tucson AZ 85721 USA wwang@pharmacy.arizona.edu

## Abstract

While strategies involving a 2e^−^ transfer pathway have dictated glycosylation development, the direct glycosylation of readily accessible glycosyl donors as radical precursors is particularly appealing because of high radical anomeric selectivity and atom- and step-economy. However, the development of the radical process has been challenging owing to notorious competing reduction, elimination and/or S_N_ side reactions of commonly used, labile glycosyl donors. Here we introduce an organophotocatalytic strategy through which glycosyl bromides can be efficiently converted into corresponding anomeric radicals by photoredox mediated HAT catalysis without a transition metal or a directing group and achieve highly anomeric selectivity. The power of this platform has been demonstrated by the mild reaction conditions enabling the synthesis of challenging α-1,2-*cis*-thioglycosides, the tolerance of various functional groups and the broad substrate scope for both common pentoses and hexoses. Furthermore, this general approach is compatible with both sp^2^ and sp^3^ sulfur electrophiles and late-stage glycodiversification for a total of 50 substrates probed.

## Abstract 2

Organophotoredox mediated HAT catalysis is developed for achieving high anomerically selective thioglycosylation of glycosyl bromides.

## Graphical abstract

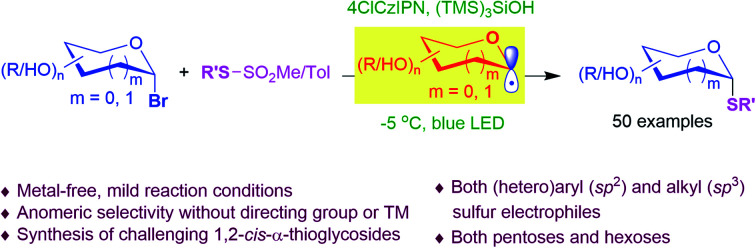

## Introduction

**Scheme 1:**
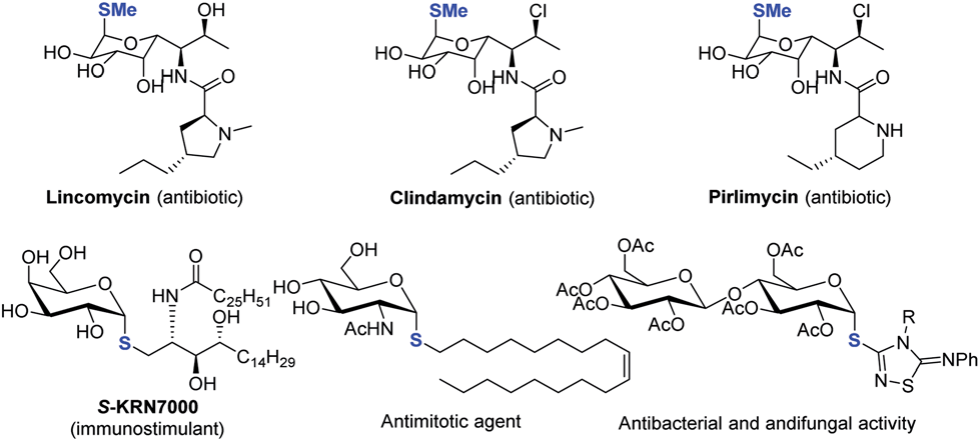
Selected examples of thioglycosides with α-1,2-*cis*-configuration.

Despite the fact that *O*-linked glycosides are a dominant form in biologically important glycoconjugates,^1^ the replacement of “*O*” by *C*-, *N*- and *S*-linked glycosides offers the merits of improved hydrolytic stability and/or bioactivity while maintaining similar conformational preferences. ^2^ In particular, thioglycosides have emerged as a privileged class of structures owing to their broad spectrum of biological activities (see representative examples in Scheme 1).^2–5^ Moreover, they are widely used as glycosyl donors in glycosylation reactions.^6^ The broad biological and synthetic utility has triggered significant interest in the development of efficient methods to construct a C–S bond with a defined anomeric configuration, which plays key roles in biological activities.

**Scheme 2:**
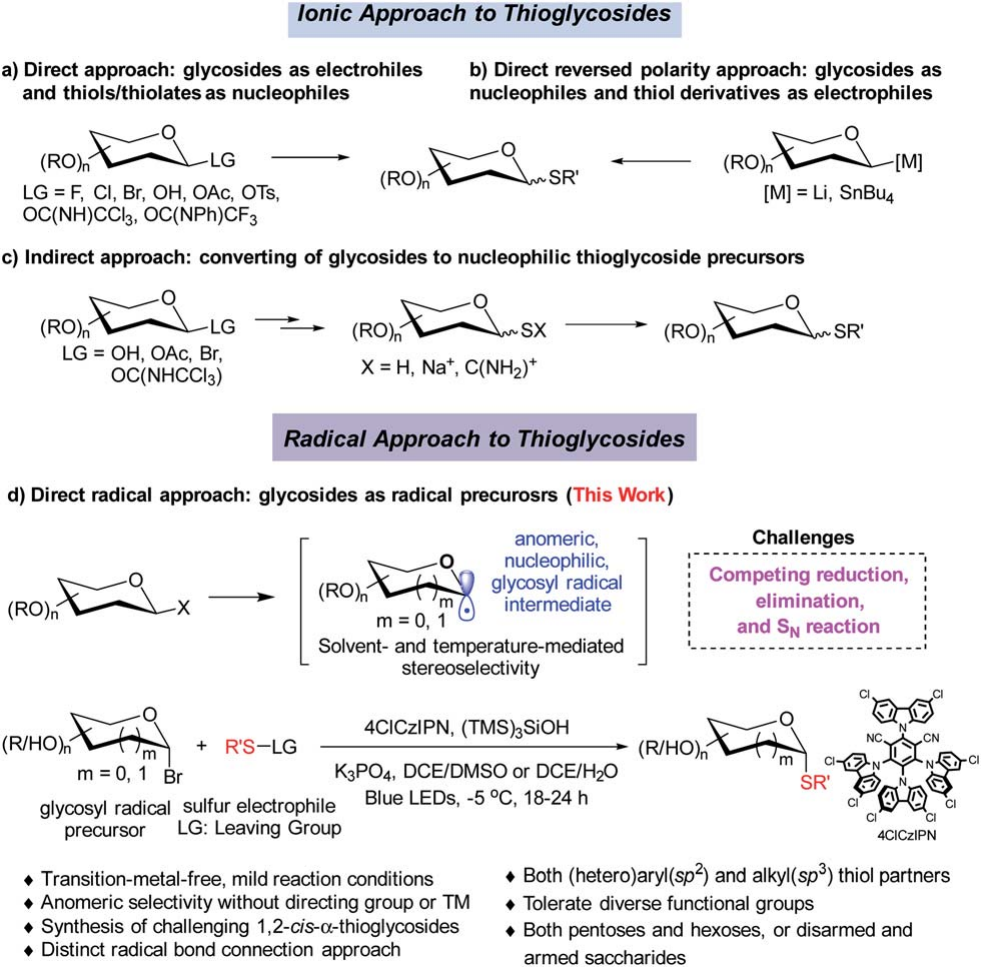
Ionic and radical thioglycosylation.

Strategies involving an ionic 2e^−^ transfer pathway have dictated the C–S bond formation development.^7–13^ Direct replacement by a thiol with a glycosyl donor is an attractive approach in that both starting materials are readily accessible, but gives a mixture of α/β anomers in most cases (Scheme 2a).^8^ To overcome these limitations, the methods of reversing the polarity at the anomeric carbon have been developed (Scheme 2b).^9^ These elegant methods enable the stereoselective control formation of both α and β anomers but with limited scope of saccharides.^9*a*^ Indirect methods using preformed anomeric thiols offer versatile approaches to thioglycosides (Scheme 2c).^10–13^ Nonetheless, the anomeric stereoselectivity of these processes depends on the nature of the anomeric thiols. In particular, few methods are capable of selectively constructing the challenging α-1,2-*cis*-thioglycosides,^8*b*^ featured in a number of natural products and bioactive molecules (Scheme 1).

Radical cross coupling offers a distinct paradigm for stereoselective construction of glycosidic bonds.^14^ Anomeric radicals have been elegantly explored for highly stereoselective *C*-glycosidic bond formation with a transition metal (TM).^15–17^ However, stereoselective C–S bond formation through the glycosyl radical has remained elusive (Scheme 2d).^17^ This is attributed to: (1) reduction of glycosyl radicals by HAT (hydrogen atom transfer) donors;^18^ (2) elimination reaction of labile glycosyl donors with a TM catalyst;^19^ (3) competing S_N_2 reaction with thiols, which could compromise the anomeric selectivity.^2*b*,7^ Therefore, stable radical precursors such as glycosyl stannanes are designed to minimize these issues.^17^ Given the fact that the glycosyl radical can favour the formation of anomeric C1 conformation, we deliberately push the limit by developing an organophotocatalytic approach without a directing group or a TM for stereoselective *S*-glycosylation. Herein, we wish to disclose the results of the investigation, which has led to a general organophotocatalyzed thiolation of glycosyl bromides with highly stereoselective control (Scheme 2d).

## Results and discussion

In our own efforts, recently we have developed visible-light-mediated glycosyl radical reactions for the synthesis of *C*-glycosides.^15^ In addition, we reported an organophotocatalytic thiolation of acyl radical method with thiosulfonates.^20^ These chemistries guided us to explore a new thioglycosylation reaction. The reaction of α-glucopyranosyl bromide **1a** with thiosulfonate **2a** and 4CzIPN^21^ as a photocatalyst (PS) was probed (Table 1 and Tables S1–S6†). First, we examined several commonly used reductants including *i*Pr_2_NEt, Hantzsch ester, and ascorbic acid (Table S1,† entries 2, 6 and 7) for the generation of the glycosyl radical. Disappointedly, only the reduced product **4** was obtained. It should be pointed out that this is a general problem in using glycosyl halides as radical progenitors in glycosylation.^18^ Minimizing the issue requires a radical capable of effective dehalogenation whereas the hydrogenated product should be a weak H-donor. A silyl or a silyloxy radical can induce dehalogenation while the strong Si–H and Si–O–H make them more difficult to abstract.^22^ Therefore, various silanes were screened and (TMS)_3_SiOH was the best, giving **3a** in 37% yield (Table S1,† entries 3–5 and 8–9). A survey of PSs revealed 4ClCzIPN^21b,c^ as the optimal promoter (65% yield, Table S2† and 1, entries 2–4). The process was also sensitive to bases (entries 4–6 and Table S4†) and K_3_PO_4_ gave **3a** in high yield. Among the thiosulfonates probed (entries 6–12), methanethiosulfonate (**2a**) was the best, possibly attributed to the lower hindrance and relatively high redox stability (*E*_red_ = −1.65 V *vs.* SCE, Fig. S3†). Glycosyl chloride (**1b**) did not undergo the dechlorination presumably due to its strong C–Cl bond (entry 13). To further improve the stereoselectivity (entry 6), we conducted reaction optimization including the solvent and reaction temperature (Table 1, entries 14–15 and Tables S3 and S6†). It was found that the biphasic solvent (DCE : H_2_O = 2 : 1) could not only retain the high anomeric selectivity but also increase the yield (76%, entry 1), and a low temperature (−5 °C) is also required to maintain good yield and anomeric selectivity (entry 14, 15). The control experiments confirmed that base, light, (TMS)_3_SiOH, and PS were essential for this transformation (entries 16–17).

**Table 1:** Reaction optimization

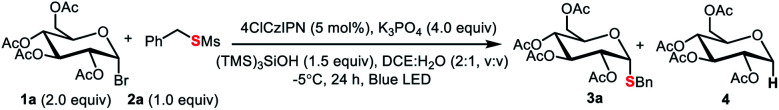			
Entry	Variation from the “standard conditions”^a^	Yield^b^ (**3a**, %)	α : β^c^
1	None	76 (72)^d^	>20 : 1
2	4CzIPN (5 mol%), **2c**, Na_2_CO_3_ (4.0 equiv.), DMSO, rt	37	<10 : 1
3	4BrCzIPN (5 mol%), **2c**, Na_2_CO_3_ (4.0 equiv.), DMSO, rt	33	<10 : 1
4	4ClCzIPN (5 mol%), **2c**, Na_2_CO_3_ (4.0 equiv.), DMSO, rt	65	<10 : 1
5	Cs_2_CO_3_ instead of K_3_PO_4_, DCE : DMSO (1 : 1, v/v), rt	Trace	—
6	DCE : DMSO (1 : 1, v/v), rt	80	<10 : 1
7	**2b** instead of **2a**, DCE : DMSO (1 : 1, v/v), rt	72	<10 : 1
8	**2d** instead of **2a**, DCE : DMSO (1 : 1, v/v), rt	66	<10 : 1
9	**2d** instead of **2a**, DCE : DMSO (1 : 1, v/v), rt	68	<10 : 1
10	**2e** instead of **2a**, DCE : DMSO (1 : 1, v/v), rt	Trace	—
11	**2f** instead of **2a**, DCE : DMSO (1 : 1, v/v), rt	66	<10 : 1
12	**2g** instead of **2a**, DCE : DMSO (1 : 1, v/v), rt	Trace	—
13	**1b** instead of **1a**	Trace	—
14	DCE instead of DCE : H_2_O (2 : 1, v/v), rt	60	17 : 1
15	DCE instead of DCE : H_2_O (2 : 1, v/v), −5 °C	67	>20 : 1
16	Without 4ClCzIPN, (TMS)_3_SiOH or K_3_PO_4_	Trace	—
17	Under dark conditions	Trace	—
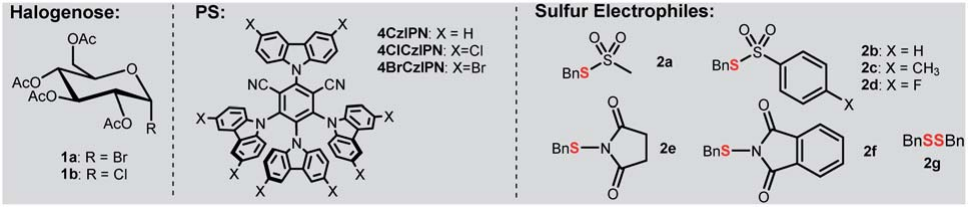			

a Standard conditions: unless specified, a mixture of glycosyl bromide (0.2 mmol), sulfur electrophile (0.1 mmol), 4ClCzIPN (0.005 mmol), K_3_PO_4_ (0.4 mmol), and (TMS)_3_SiOH (0.15 mmol) in DCE/DMSO (1 mL, 1 : 1, v/v) or DCE/H_2_O (1.5 mL, 2 : 1, v/v) was irradiated with 40 W Kessil blue LEDs in a N_2_ atmosphere at −5 °C for 24 h.

b Yield determined by ^1^H NMR using 1,1,2,2-tetrachloroethane as an internal reference.

c Ratio determined by crude ^1^H NMR.

d Isolated yield.

**Scheme 3:**
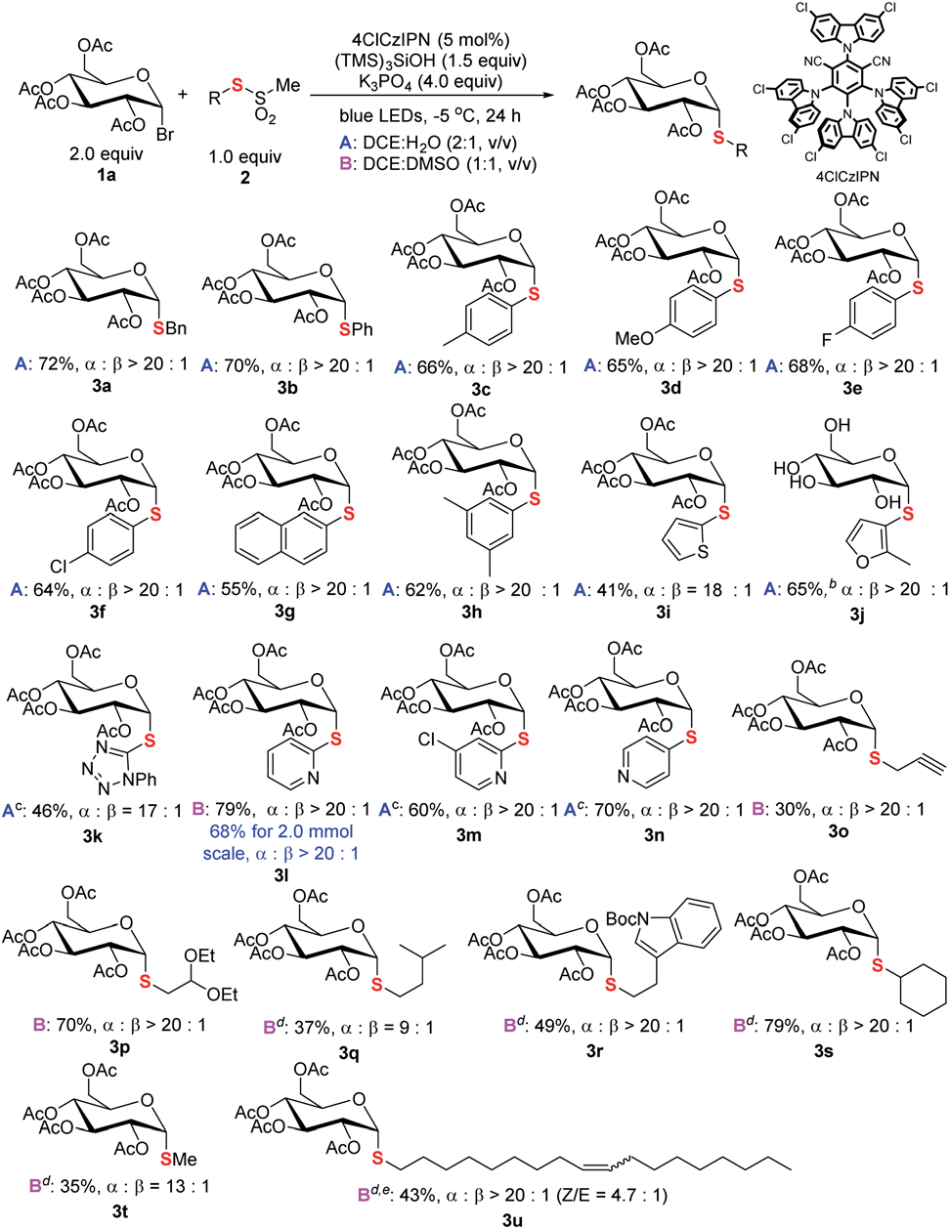
Scope of thiosulfonates. ^*a*^Reaction conditions: unless specified, see footnote a of Table 1 and the ESI;† isolated yield; the ratio of α and β anomers determined by crude ^1^H NMR. ^*b*^Yield after hydrolysis of the acyl group. ^*c*^Disulfide used. ^*d*^Toluenethiosulfonate used. ^*e*^*Z*/*E* ratio determined by ^1^H NMR.

The generality of the new *S*-glycosylation was examined. We first evaluated the performance using glucosyl bromide (**1a**) as a radical donor for coupling with various thiosulfonates **2** (Scheme 3). The process serves as a general approach to both aryl and alkyl thioglycosides. Uniformly high axial selectivities are observed regardless of the nature of the sulfur electrophiles. With respect to aryls, electron-neutral (**3b**), -donating (**3c–3d**, **3h**), and -withdrawing (**3e–3f**) groups on the phenyl ring and fused aromatic (**3g**) can be tolerated. Moreover, heteroaromatic thiosulfonates such as thiophenyl (**3i**) and furanyl (**3j**) enabled access to medicinally valued thioglycosides. The tetrazole derived disulfide instead of labile thiosulfonate could serve as an alternative and delivered the desired **3k**. The reaction performed in DCE : H_2_O failed for pyridinyl thiosulfonate. Decent results (**3l**, 79%, α : β > 20 : 1) were obtained with DCE : DMSO (condition B). The protocol can also be applied in gram scale synthesis. Notably, less reactive sp^3^ alkyl glycosides **3o–3s** could be synthesized with the protocol.^17^

For even less electrophilic substrates, *p*-tolylthiosulfonates (**3q–3u**) displayed better performance than methylthiosulfonates. Particularly, a long alkyl chain with a Z-double bond product (**3u**), which exhibits intriguing antitumor activity (Scheme 1), is efficiently prepared with high diastereoselectivity. The limitation of the method is also realized. C_2_–*N*–Ac-saccharides such as d-glucosamine failed to react due to the lability of these reactants (see Fig. S5 in the ESI†).

**Scheme 4:**
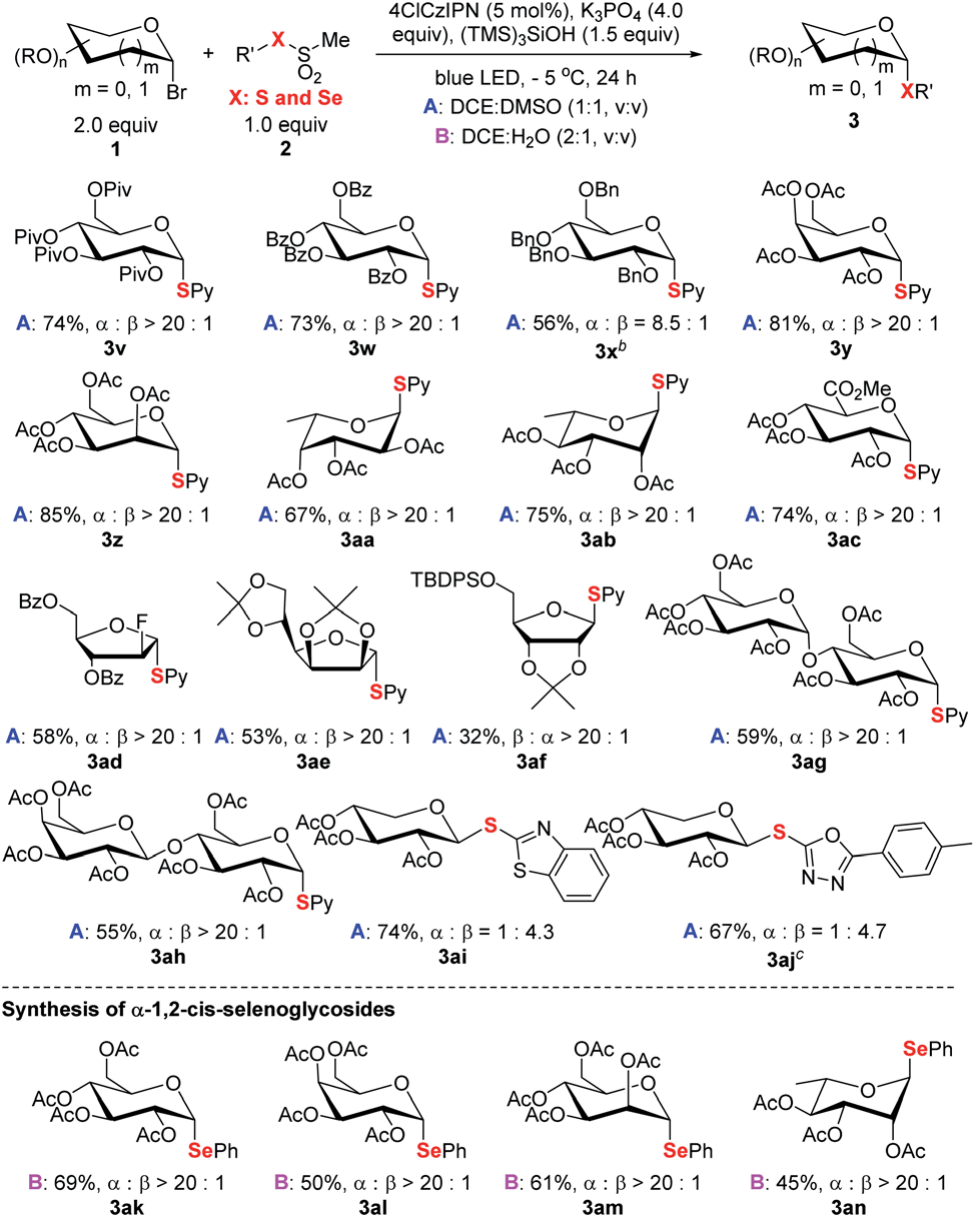
Scope of saccharides and selenoglycosylation. ^*a*^Reaction conditions: unless specified see footnote *a* of Table 1 and the ESI;† isolated yield; the ratio of α and β anomers determined by crude ^1^H NMR. ^*b*^3.0 equiv. of glycosyl bromide used. ^*c*^Disulfide used.

The alternation of sugars was probed next (Scheme 4). Both common hexoses (glucose **3v–3x**, galactose **3y**, mannose **3z**, fucose **3aa**, rhamnopyranose **3ab**, and glucuronic acid **3ac**) and pentoses (**3ad–3af**) gave good yields and high stereoselectivity. Among the tested monosaccharides, except ribose (**3af**) adopting expected β selectivity owning to the steric effect, the others gave expected α-selectivity. Furthermore, disaccharides (**3ag** and **3ah**) could participate in the process smoothly. For xyloses (**3ai–3aj**), the obtained products adopted β orientation since the anomeric xylosyl radical is β selective.^23^ Besides pyridyl (Py), other pharmaceutically relevant heteroaromatics such as benzothiazole and oxadiazole (**3ai**, **3aj**) could be efficiently incorporated. This offers a viable strategy for the synthesis of xylose-derived bioactive analogs.^4*c*^ Finally, the strategy can also be extended for the synthesis of synthetically challenging α-1,2-*cis*-selenoglycosides (Scheme 4 and Table S7†).^24^ For example, under the reaction conditions (see footnote a of Table 1, DCE : H_2_O, v/v, 2 : 1), four glycosyl bromides could couple with methyl phenylselenyl sulfonate to deliver the corresponding α-seleno-glycosides **3ak–3an** with uniformly high stereoselectivity (α : β > 20 : 1).

**Scheme 5:**
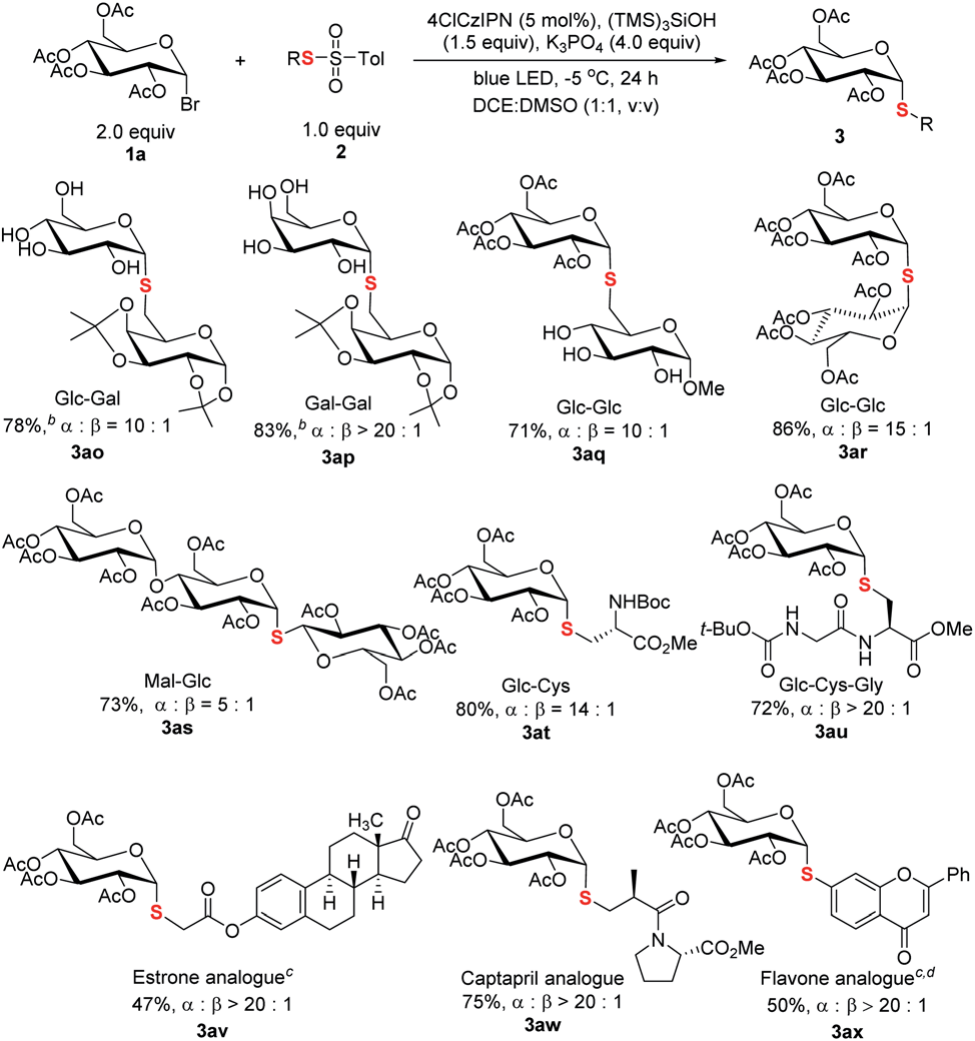
Thiodiversification of pharmaceutically relevant structures. ^*a*^Reaction conditions: unless specified, see footnote a of Table 1 and the ESI;† isolated yield; ratio of α and β anomers determined by crude ^1^H NMR. ^*b*^The product after hydrolysis. ^*c*^Methylthiosulfonate used. ^*d*^DCE : H_2_O (1.5 mL, 2 : 1, v/v) used as the solvent.

The capacity of selective functionalization of biologically relevant structures and therapeutics is the testament to the synthetic power of a methodology. As demonstrated (Scheme 5), C1-6′ connected thioglycosides **3ao–3aq** were efficiently synthesized. It is noted that a native unprotected saccharide thiosulfonate could be used for efficient cross coupling (**3aq**). Moreover, it is particularly noteworthy that the protocol is amenable for the synthesis of α-*S*-linked 1,1′-disaccharides with C1 thiol electrophiles, a synthetic challenge in glycosylation,^25^ as demonstrated in 1-thiodisaccharides (**3ar**) and thiotrisaccharide (**3as**). Furthermore, α-linked thioglycosyl amino acid **3at** and peptide **3au** could be efficiently constructed. The synthetic manifold was further exemplified by late-stage thioglycosylation of therapeutics. The incorporation of thioglycosyl moieties into estrone (**3av**), Captopril (**3aw**), and flavone (**3ax**) has been realized smoothly.

**Scheme 6:**
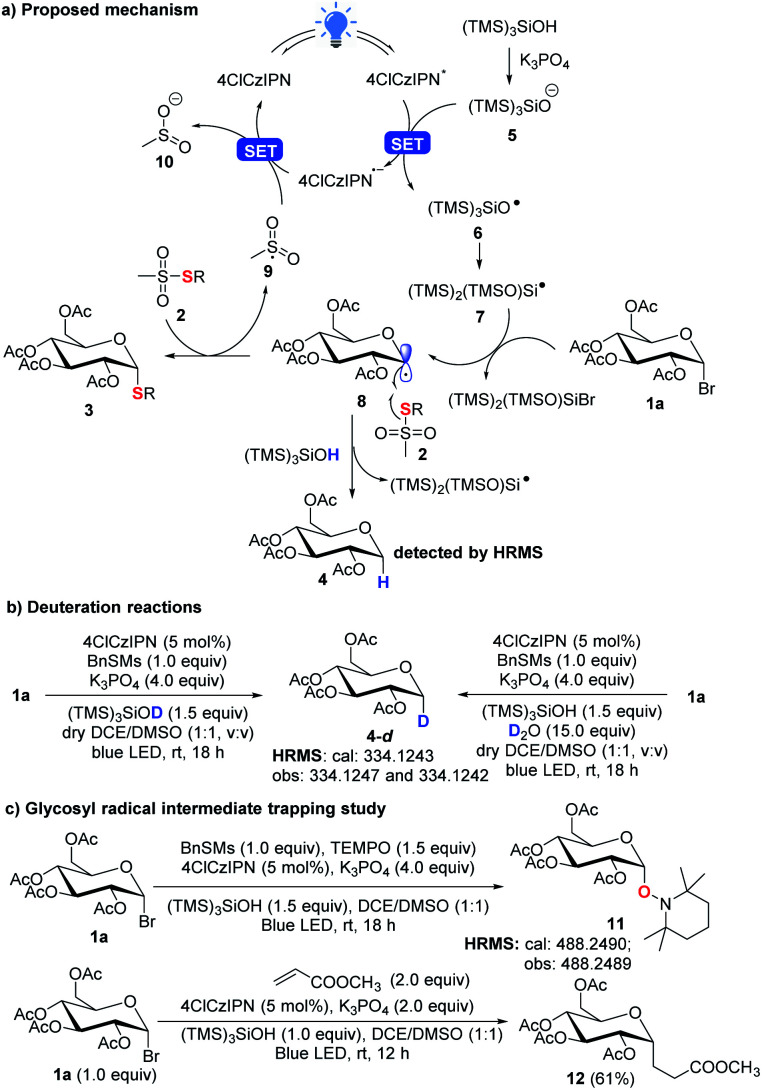
Proposed mechanism and mechanism studies.

In the new thioglycosylation reaction, critically (TMS)_3_SiOH was identified as a HAT reagent, which could efficiently suppress the undesired reduction of the radical **8** (Scheme 6a). This may be attributed to the strong O–H bond (calculated BDE = 98 kcal mol^−1^, see the ESI,† BDE of S–H: 83 kcal mol^−1^)^26,27^ and steric hindrance, making the H difficult for **8** to abstract. This strong bond also echoes the use of stronger 4ClCzIPN (E*/*E*˙^−^ = 1.58 V *vs.* SCE)^21^ to oxidize the silyloxide [((TMS)_3_SiO^−^/TMS)_3_SiO˙ = 1.54 V *vs.* SCE)]. A spontaneous Brook rearrangement of silyloxy radical **6** forms a silicon-centred radical **7**,^28,21*b*^ which acts as an effective debrominator. The anomeric effect makes the radical **8** axially positioned and directs α-selective coupling with thiosulfonate **2**. In the reactions, we still observed a notable amount of the reduction product **4**. It is believed that it is produced from the reaction of **8** with (TMS)_3_SiOH, which was confirmed by deuteration experiments with observed deuterated product **4-d** (Scheme 6b). This also rationalizes that 2 equiv. of glycosyl bromide **1** is used to ensure high efficiency of the thioglycosylation process. Finally, a radical trapping study with TEMPO and methyl acrylate^16*d*^ further confirms the radical engaged process (Scheme 6c).

## Conclusions

In conclusion, we have developed a metal-free, glycosyl radical strategy for the stereoselective synthesis of thioglycosides by employing commonly used glycosyl bromides as radical precursors. The uncovered organophotoredox mediated HAT radical pathway can highly stereoselectively induce the formation of an anomeric C–S bond while minimizing the side reactions. The power of the platform has been underscored by the mild reaction conditions enabling the synthesis of challenging α-1,2-*cis*-thioglycosides, the tolerance of various functional groups and the broad substrate scope for both common pentoses and hexoses. Furthermore, this general approach is compatible with both sp^2^ and sp^3^ sulfur electrophiles and late-stage glycodiversification. It is expected that the strategy enabling the efficient generation of glycosyl radicals from labile glycosyl bromides can offer a reliable alternative for the synthesis of *C*- and other hetero-glycosides.

## Conflicts of interest

There are no conflicts to declare.

## Supplementary Material

SC-011-D0SC04136J-s001

## References

[cit1] Rudd P. M., Elliot T., Cresswell P., Wilson I. A., Dwek R. A. (2001). Science.

[cit2] Thayer D. A., Yu H. N., Galan M. C., Wong C.-H. (2005). Angew. Chem., Int. Ed..

[cit3] Schwarz S., Shen J., Kadlec K., Wang Y., Michael G. B., Feßler A. T., Vester B. (2016). Cold Spring Harb. Perspect. Med..

[cit4] Witczak Z. J. (1999). Curr. Med. Chem..

[cit5] Comber R. N., Friedrich J. D., Dunshee D. A., Petty S. L., Secrist J. A. (1994). Carbohydr. Res..

[cit6] Ikuta D., Hirata Y., Wakamori S., Shimada H., Tomabechi Y., Kawasaki Y., Ikeuchi K., Hagimori T., Matsumoto S., Yamada H. (2019). Science.

[cit7] Gerz M., Matter H., Kessler H. (1993). Angew. Chem., Int. Ed. Engl..

[cit8] Ecopy S., Singh Y., Demchenko A. V. (2019). Org. Biomol. Chem..

[cit9] Baryal K. N., Zhu D., Li X., Zhu J. (2013). Angew. Chem., Int. Ed..

[cit10] Driguez H. (2001). Chembiochem.

[cit11] Montoir D., Amoura M., Ababsa Z. E. A., Vishwanatha T. M., Yen-Pon E., Robert E. V., Beltramo M., Piller V., Alami M., Aucagne V., Messaoudi S. (2018). Chem. Sci..

[cit12] Zhao G., Kaur S., Wang T. (2017). Org. Lett..

[cit13] Gamblin D. P., Garnier P., Kasteren S., Oldham N. J., Fairbanks A. J., Davis B. G. (2004). Angew. Chem., Int. Ed..

[cit14] Smith J. M., Harwood S. J., Baran P. S. (2018). Acc. Chem. Res..

[cit15] Ji P., Zhang Y., Wei Y., Huang H., Hu W., Mariano P. A., Wang W. (2019). Org. Lett..

[cit16] Yang Y., Yu B. (2017). Chem. Rev..

[cit17] Zhu F., Zhang S.-Q., Chen Z., Rui J., Hong X., Walczak M. A. (2020). J. Am. Chem. Soc..

[cit18] Andrews R. S., Becker J. J., Gagné M. R. (2011). Org. Lett..

[cit19] Miquel N., Doisneau G., Beau J.-M. (2000). Angew. Chem., Int. Ed..

[cit20] Zhang Y., Ji P., Hu W., Wei Y., Huang H., Wang W. (2019). Chem.–Eur. J..

[cit21] Uoyama H., Goushi K., Shizu K., Nomura H., Adachi C. (2012). Nature.

[cit22] Chatgilialoglu C. (1995). Chem. Rev..

[cit23] Giese B., Dupuis J., Leising M., Nix M., Lindner H. J. (1987). Carbohydr. Res..

[cit24] McDonagh A. W., Mahon M. F., Murphy P. V. (2016). Org. Lett..

[cit25] Izumi S., Kobayashi Y., Takemoto Y. (2020). Angew. Chem., Int. Ed..

[cit26] Denes F., Pichowicz M., Povie G., Renaud P. (2014). Chem. Rev..

[cit27] http://butane.chem.uiuc.edu/cyerkes/Chem104ACSpring2009/Genchemref/bondenergies.html.

[cit28] Paredes M. D., Alonso R. (2000). J. Org. Chem..

