# Supplementary material for: Direct, stereoselective thioglycosylation enabled by an organophotoredox radical strategy
Source: Chem Sci. 2020 Oct 19;11(48):13079–84. doi: 10.1039/d0sc04136j (PMC8163235; doi:10.1039/d0sc04136j)
Supplement: SC-011-D0SC04136J-s001 [file SC-011-D0SC04136J-s001.pdf]

Electronic Supplementary Material (ESI) for Chemical Science.
This journal is © The Royal Society of Chemistry 2020

Electronic Supplementary Information (ESI)

Direct, Stereoselective Thioglycosylation Enabled by an Organophotoredox Radical Strategy

Peng Ji, Yueteng Zhang, Feng Gao, Fangchao Bi, and Wei Wang*

Departments of Pharmacology and Toxicology and Chemistry and Biochemistry, BIO5 Institute, and University of Arizona Cancer Centre, University of Arizona, Tucson, Arizona 85721

Table of Contents

1. General Information S2
2. Optimization of Reaction Conditions S3
3. General Procedures for Synthesis of 3 .... S9
4. Reaction Setup and Measurement of Redox Potential S11
5. Substrates Synthesis S13
6. Synthesis of 3l in Gram Scale S23
7. Glycosylation Reaction Using Thioglycoside 3v as the Glycosyl Donor S24
8. References S25
9. Compound Characterization Data S26
10. Spectral Data S52
11. HRMS Spectra of 4a and 11 .... S108

1. General Information

Commercially available reagents were purchased from Sigma Aldrich, Matrix Chemical, AKSci, Acros, Ambeed, Alfa Aesar, TCI, and Adamas-beta, and used as received unless otherwise noted. Except $Ir(ppy)_3$ purchased form Adamas-beta, photosensitizers including 4CzIPN, 4ClCIPN, 4BrCzIPN are prepared according to corresponding literatures. Merck 60 silica gel was used for chromatography, and Whatman silica gel plates with a fluorescence F254 indicator were used for thin-layer chromatography (TLC) analysis. ^1H and ^{13}C NMR spectra were recorded on Varian 400 MHz. Chemical shifts in ^1H NMR spectra are reported in parts per million (ppm) relative to residual chloroform (7.26 ppm) or dimethyl sulfoxide (2.50 ppm) as internal standards. ^1H NMR data are reported as follows: chemical shift, multiplicity (s = singlet, d = doublet, q = quartet, quintet, m = multiplet), coupling constant in Hertz (Hz) and hydrogen numbers based on integration intensities. ^{13}C NMR chemical shifts are reported in ppm relative to the central peak of $CDCl_3$ (77.16 ppm) or $(CD_3)_2SO$ (39.52 ppm) as internal standards. Cyclic voltammetry was performed at 25 °C on a CH Instrument CHI604xD electrochemical analyzer using a glassy carbon working electrode, a platinum wire counter electrode, and the Ag/AgCl reference electrode calibrated using ferrocene redox couple (4.8 eV below vacuum). Acetonitrile was degassed by the freeze-pump-thaw method and used within one week.

2. Optimization of Reaction Conditions

2.1. Optimization of reaction conditions:

Table S1. Effect of different reductant on reaction efficiency

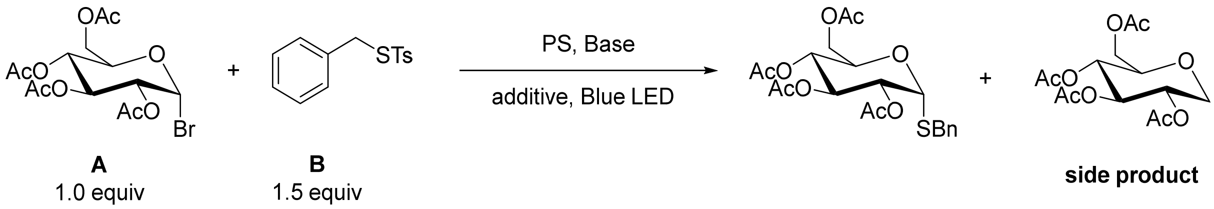

Photocatalyst (5 mol%)	Additive (1.0 equiv)	Base (1.0 equiv)	Solvent (0.1 M)	Time (h)	Yield (%)[a]	Note
4CzIPN	TTMSS	Cs_2CO_3	MeCN	18	29	A (1.0), B (2.0)
4CzIPN	$AscH_2$	Cs_2CO_3	MeCN	18	trace	
4CzIPN	Ph_3SiH	Cs_2CO_3	MeCN	18	trace	
4CzIPN	Et_3SiH	Cs_2CO_3	MeCN	18	trace	
4CzIPN	TTMSS	Cs_2CO_3	MeCN	18	25	
4CzIPN	DIPEA	-	MeCN	18	trace	side product
4CzIPN	HE	DIPEA	MeCN	18	trace	side product
4CzIPN	$(TMS)_3SiOH$	Cs_2CO_3	MeCN	18	trace	side product
4CzIPN	$(TMS)_3SiOH$	Na_2CO_3	DMSO	18	37	
4ClCzIPN	$(TMS)_3SiOH$	Na_2CO_3	DMSO	18	65	
4BrCzIPN	$(TMS)_3SiOH$	Na_2CO_3	DMSO	18	33	

[a]The yields are determined by ^{1}H NMR using 1,1,2,2-tetrachloroethane as internal reference.

Table S2. Effect of different photocatalysts on the reaction efficiency

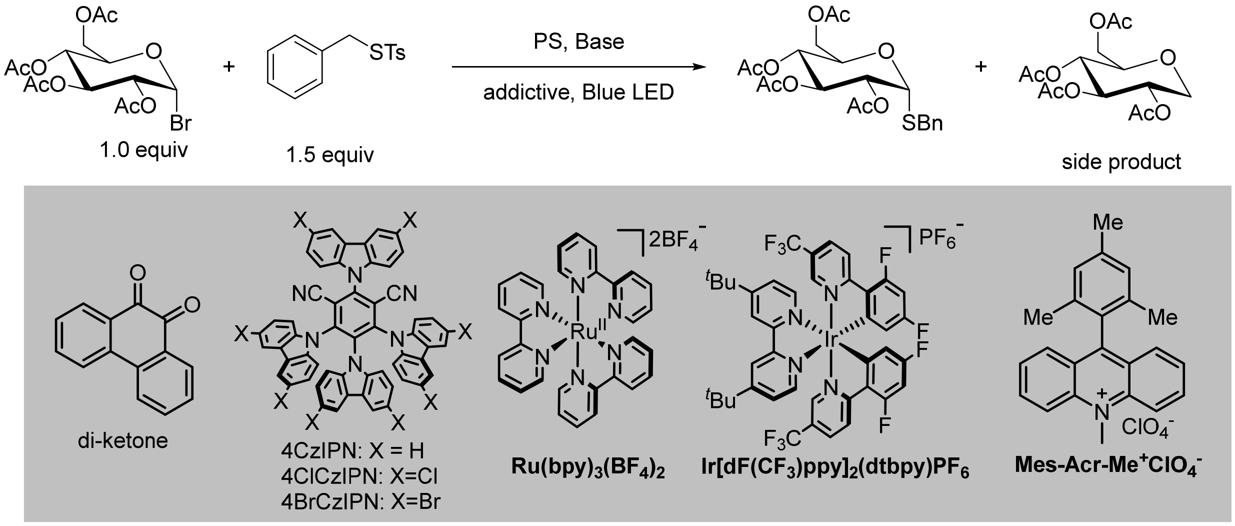

Photocatalyst (5 mol%)	Additive (0.8 equiv)	Base (1.0 equiv)	Solvent (0.1 M)	Time (h)	Yield (%)[a]
Di-ketone (HAT, 10 mol%)	TTMSS	Cs_2CO_3	MeCN	18	21
$Ir[dF(CF_3)ppy]_2(dtbpy)PF_6$	TTMSS	Cs_2CO_3	MeCN	18	41
$Ru(bpy)_3(BF_4)_2$	TTMSS	Cs_2CO_3	MeCN	18	41
Mes-Acr-$Me^+ClO_4^-$	TTMSS	Cs_2CO_3	MeCN	18	16
$Ir(ppy)_2(dtbpy)PF_6$	TTMSS	Cs_2CO_3	MeCN	18	41
Eosin Y	TTMSS	-	MeCN	18	13
Rose Begal	TTMSS	Cs_2CO_3	MeCN	18	33
Eosin Y	TTMSS	Cs_2CO_3	MeCN	18	24
4CzIPN	TTMSS	Cs_2CO_3	MeCN	18	49
4ClCzIPN	TTMSS	Cs_2CO_3	MeCN	18	29
4BrCzIPN	TTMSS	Cs_2CO_3	MeCN	18	45

[a]The yields were determined by ^1H NMR using 1,1,2,2-tetrachloroethane as internal reference.

Table S3. Optimization using $(TMS)_3SiOH$ as additive and 4ClCzIPN as photocatalyst

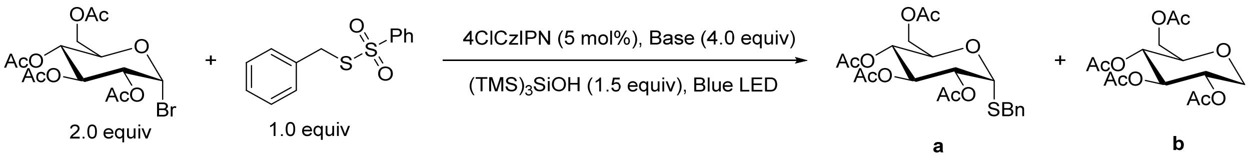

Photocatalyst (5 mol%)	Additive (1.5 equiv)	Base (4.0 equiv)	Solvent (0.1 M)	Time	Yield (%),[a] a : b[b]	Note
4ClCzIPN	$(TMS)_3SiOH$	Na_2CO_3	DMSO	18 h	25 (4 : 1)	Sugar: 1.0, Si: 1.0, S: 1.5
4ClCzIPN	$(TMS)_3SiOH$	K_3PO_4	MeCN	18 h	70 (3.1 : 1)	Sugar: 2.0, Si: 1.5, S: 1.0
4ClCzIPN	$(TMS)_3SiOH$	K_3PO_4	DCE	18 h	60 (1 : 2.5)	Sugar: 2.0, Si: 1.5, S: 1.0
4ClCzIPN	$(TMS)_3SiOH$	K_3PO_4	EtOAc	18 h	40 (1 : 4.2)	Sugar: 2.0, Si: 1.5, S: 1.0
4ClCzIPN	$(TMS)_3SiOH$	K_3PO_4	$PhCF_3$	18 h	60 (1 : 1.6)	Sugar: 2.0, Si: 1.5, S: 1.0
4ClCzIPN	$(TMS)_3SiOH$	K_3PO_4	*t*-BuCN or Dioxane	18 h	28 (1: 2.8), 40 (1:3.2)	Sugar: 2.0, Si: 1.5, S: 1.0
4ClCzIPN	$(TMS)_3SiOH$	K_3PO_4	DCE/DMSO (1:1)	18 h	72 (2.3 : 1)	Sugar: 2.0, Si: 1.5, S: 1.0
4ClCzIPN	$(TMS)_3SiOH$	K_3PO_4	DCE/DMSO (2:1)	18 h	68 (2.3 : 1)	Sugar: 2.0, Si: 1.5, S: 1.0
4ClCzIPN	$(TMS)_3SiOH$	K_3PO_4	DCE/DMSO (5:1)	18 h	68 (1.3 : 1)	Sugar: 2.0, Si: 1.5, S: 1.0
4ClCzIPN	$(TMS)_3SiOH$	K_3PO_4	Toluene	18 h	54 (1 : 1.7)	Sugar: 2.0, Si: 1.5, S: 1.0
4ClCzIPN	$(TMS)_3SiOH$	K_3PO_4	DMSO	18 h	55 (2.3 : 1)	Sugar: 2.0, Si: 1.5, S: 1.0
4ClCzIPN	$(TMS)_3SiOH$	$NaHCO_3$	DMSO	18 h	68 (1 : 1.25)	Sugar: 2.0, Si: 1.5, S: 1.0
4ClCzIPN	$(TMS)_3SiOH$	Na_2CO_3	DMSO	18 h	Trace, sugar left	Glycosyl chloride as RP
4ClCzIPN	$(TMS)_3SiOH$	Na_3CO_3	DCE/DMSO (1:1)	18 h	42 (1 : 1.3)	Sugar: 2.0, Si: 1.5, S: 1.0

[a]The yields were determined by ^1H NMR using 1,1,2,2-tetrachloroethane as internal reference. [b]The ratio is determined by the crude ^1H NMR.

Table S4. Optimization of reaction conditions

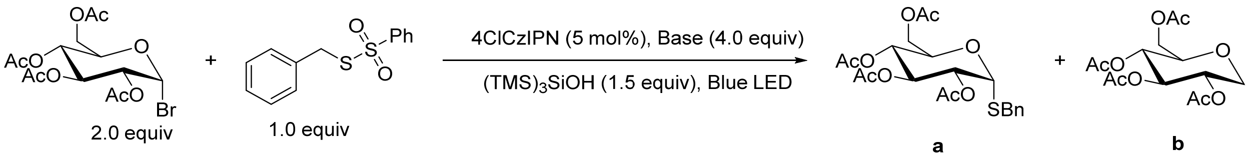

Photocatalyst (5 mol%)	Addictive (1.5 equiv)	Base (4.0 equiv)	Solvent (0.1 M)	Time	Yield (%),[a] a : b[b]	Note
4ClCzIPN	$(TMS)_3SiOH$	Cs_2CO_3	DCE/DMSO (1:1)	18 h	trace	Sugar: 1.0, Si: 1.5, S: 1.0
4ClCzIPN	$(TMS)_3SiOH$	K_3PO_4	$PhCF_3$	18 h	32 (1 : 2.8)	Sugar: 1.0, Si: 1.0, S: 1.5
4ClCzIPN	$(TMS)_3SiOH$	K_3PO_4	DCE/DMSO (1:1)	18 h	38 (1 : 1.8)	Sugar: 1.0, Si: 1.0, S: 1.5
4ClCzIPN	$(TMS)_3SiOH$	K_3PO_4	$PhCF_3$/DMSO (1:1)	18 h	64 (2.1 : 1)	Sugar: 2.0, Si: 1.5, S: 1.0
4ClCzIPN	$(TMS)_3SiOH$	K_3PO_4	DCE/ACN (1:1)	18 h	50 (1 : 3)	Sugar: 2.0, Si: 1.5, S: 1.0
4ClCzIPN	$(TMS)_3SiOH$	K_3PO_4	DCE	18 h	70 (1 : 3)	Sugar: 2.5, Si: 1.8, S: 1.0
4ClCzIPN	$(TMS)_3SiOH$	2,6-lutidine	DCE	18 h	40 (1 : 3)	Sugar: 2.0, Si: 1.5, S: 1.0
4ClCzIPN	$(TMS)_3SiOH$	K_3PO_4	Dry DCE/DMSO (1:1)	18 h	72 (2 : 1)	CH_3SO_2SBn
4ClCzIPN	$(TMS)_3SiOH$	Na_2CO_3	DMSO	18 h	76 (2.5 : 1)	CH_3SO_2SBn

[a]The yields were determined by ^{1}H NMR using 1,1,2,2-tetrachloroethane as internal reference. [b]The ratio is determined by the crude ^{1}H NMR.

Table S5. Temperature effect on reaction efficacy

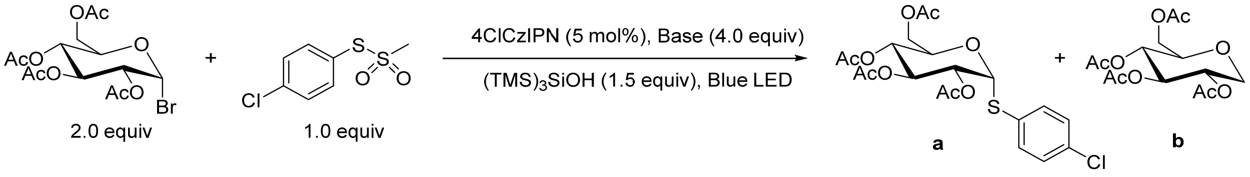

Photocatalyst (5 mol%)	Addictive (1.5 equiv)	Base (4.0 equiv)	Solvent (0.1 M)	Time	Yield (%)[a]	Temperature
4ClCzIPN	$(TMS)_3SiOH$	K_3PO_4	DCE/DMSO (1:1)	18 h	40	rt
4ClCzIPN	$(TMS)_3SiOH$	K_3PO_4	DCE/DMSO (1:1)	18 h	72	-5 °C
4ClCzIPN	$(TMS)_3SiOH$	K_3PO_4	DCE/DMSO (1:1)	18 h	36	-15 °C

[a]Yield is determined by ^{1}H NMR using 1,1,2,2-tetrachloroethane as internal reference. $\alpha : \beta$=5:1.

Table S6. Optimization of reaction conditions

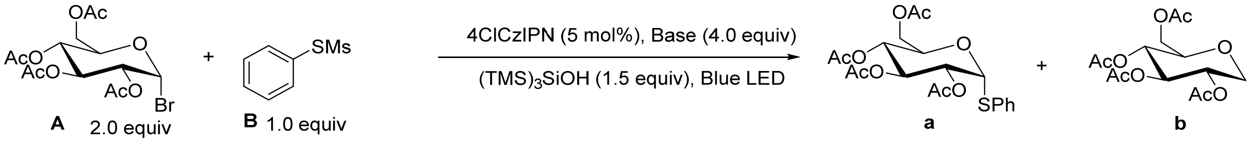

Photocatalyst (5 mol%)	Additive (1.5 equiv)	Base (4.0 equiv)	Solvent	Time (h)	Yield (%)[a] a : b[b]	α : β[b]	Note
4ClCzIPN	$(TMS)_3SiOH$	K_3PO_4	DCE/DMSO (1:1)	rt, 18-24 h	72 (2.3:1)	6.3 : 1	
4ClCzIPN	$(TMS)_3SiOH$	K_3PO_4	DCE/DMSO (5:1)	rt, 18-24 h	72 (1.8:1.3)	4.5 : 1	
4ClCzIPN	$(TMS)_3SiOH$	K_3PO_4	DCE/DMSO (10:1)	rt, 18-24 h	76 (1:2)	5 : 1	
4ClCzIPN	$(TMS)_3SiOH$	K_3PO_4	DCE	rt, 18-24 h	40 (1:6)	17 : 1	
4ClCzIPN	$(TMS)_3SiOH$	Na_2CO_3	DMSO	rt, 18-24 h	74 (2.7:3.2)	2.3 : 1	
4ClCzIPN	$(TMS)_3SiOH$	K_3PO_4	DCE/DMSO (10:1)	rt, 18-24 h	64 (1:2.8)	7.1 : 1	2.0 equiv [Si]
4ClCzIPN	$(TMS)_3SiOH$	K_3PO_4	DCE	-5 °C, 18-24 h	38 (1:1.6)	29 : 1	
4ClCzIPN	$(TMS)_3SiOH$	K_3PO_4	DCE/H_2O (10:1)	-5 °C, 18-24 h	60 (1:2.9)	42 : 1	
4ClCzIPN	$(TMS)_3SiOH$	K_3PO_4	DCE/H_2O (5:1)	-5 °C, 18-24 h	68 (1:2.4)	28 : 1	
4ClCzIPN	$(TMS)_3SiOH$	K_3PO_4	DCE/H_2O (2:1)	-5 °C, 18-24 h	76, 70[c] (1:1.6)	35 : 1	
4ClCzIPN	$(TMS)_3SiOH$	K_3PO_4	DCE/H_2O (1:1)	-5 °C, 18-24 h	64 (1:2.4)	17 : 1	
4ClCzIPN	$(TMS)_3SiOH$	K_3PO_4 (2.0 equiv)	DCE/H_2O (2:1)	-5 °C, 18-24 h	68 (1:1.9)	48 : 1	
4ClCzIPN	$(TMS)_3SiOH$	KOH	DCE/H_2O (1:1)	-5 °C, 18-24 h	48 (1:3)	35 : 1	

[a]The Yield is determined by ^{1}H NMR using 1,1,2,2-tetrachloroethane as internal reference. [b]The ratio is determined by the crude ^{1}H NMR. [c]The isolated yield

Table S7. Optimization of α-selective seleno-glycosides

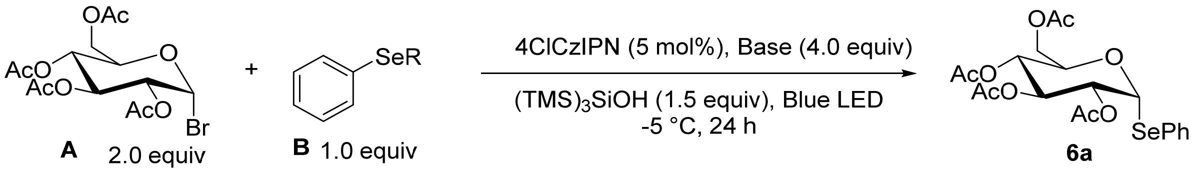

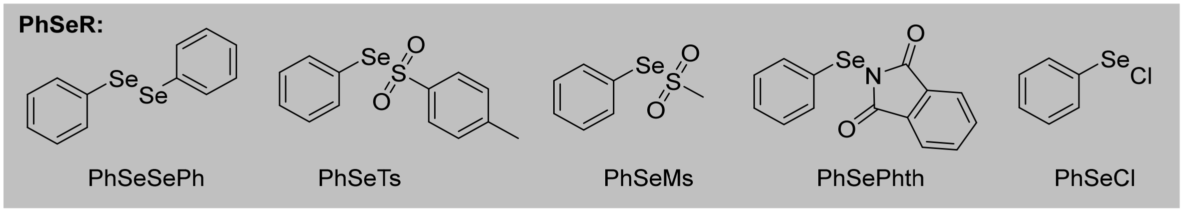

Photocatalyst (5 mol%)	Additive (1.5 equiv)	Base (4.0 equiv)	Solvent	Time (h)	Yield (%)[a]	α : β	PhSeR
4ClCzIPN	$(TMS)_3SiOH$	K_3PO_4	DCE/H_2O (2:1)	-5 °C, 24 h	58	>20:1	PhSeTs
4ClCzIPN	$(TMS)_3SiOH$	K_3PO_4	DCE/H_2O (2:1)	-5 °C, 24 h	54	>20:1	PhSeSePh
4ClCzIPN	$(TMS)_3SiOH$	K_3PO_4	DCE/H_2O (2:1)	-5 °C, 24 h	46	>20:1	PhSeCl
4ClCzIPN	$(TMS)_3SiOH$	K_3PO_4	DCE/H_2O (2:1)	-5 °C, 24 h	71	>20:1	PhSeMs
4ClCzIPN	$(TMS)_3SiOH$	Na_2CO_3	DCE/H_2O (2:1)	-5 °C, 24 h	73 (69)[b]	>20:1	PhSeMs
4ClCzIPN	$(TMS)_3SiOH$	K_3PO_4	DCE/DMSO (1:1)	-5 °C, 24 h	72	>20:1	PhSeMs

[a]Reaction condition: Glucosyl bromide (0.2 mmol), selenium electrophiles (0.1 mmol), 4ClCzIPN (0.005 mmol), $(TMS)_3SiOH$ (0.15 mmol), K_3PO_4 (0.4 mmol), DCE : H_2O (2:1, v/v), Blue LEDs, - 5 °C, 24h. Yield is determined by determined by ^1H NMR using 1,1,2,2-tetrachloroethane as internal reference [b]The isolated yield.

3. General Procedures for Synthesis of 3

3.1. <u>General procedure A:</u> using DCE:H_2O (2:1, v/v) as the solvent for compounds 3a-3m and 3b, 3c, 3d, 3e, 3f, 3g, 3h, 3i, 3j, 3k, 3m, 3ar in Schemes 3 and 5

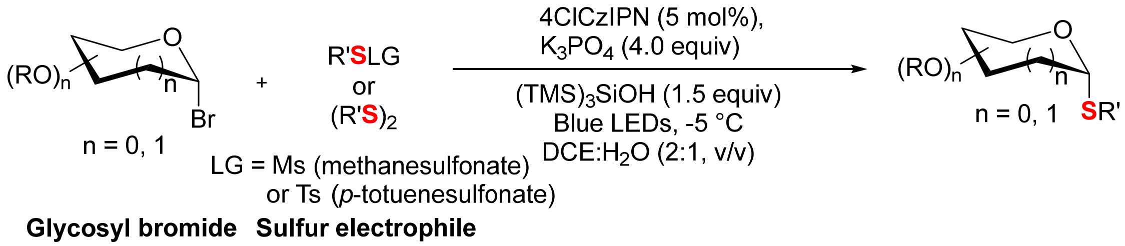

To an oven-dried 20 mL-Schlenk tube equipped with a stir bar, was added glycosyl bromide (0.2 mmol), sulfur electrophiles (0.1 mmol), 4ClCzIPN (5.3 mg, 0.005 mmol), and K_3PO_4 (85 mg, 0.4 mmol). The tube was evacuated and back-filled with N_2, then sealed with rubber stopper and parafilm. Then, DCE (1 mL) and distilled H_2O (0.5 mL) were injected into the tube by syringe under N_2 atmosphere. The solution was degassed with Freeze-Pump-Thaw Cycling technique (three times) using the dry ice/acetone as the cooling bath. After the degassing, $(TMS)_3SiOH$ (48 μL, 0.15 mmol) was added through syringe. Subsequently, the solution was stirred at about -5 °C under the irradiation of two 40 W Kessil Blue LEDs for 18-24 h (Figure 1S). After completion of the reaction, first remove the solvent in vacuum directly, and then DCE (1 mL) and DMSO (1 mL) was added, followed by the addition of K_3PO_4 (42 mg, 0.2 mmol), and stirred at room temperature for 6 h without the irradiation of blue LEDs to remove the remaining glycosyl bromide. After the reaction, 10 mL ethyl acetate was added, and the organic phase was washed with H_2O (5 mL, one time), brine (5 mL, two times), and then dried with anhydrous $MgSO_4$, removed the solvent under vacuum. The desired products were obtained in the corresponding yields after purification by flash column chromatography on silica gel eluting with hexane/ethyl acetate or ethyl acetate/DCM in proper ratio. The ratio of α:β was determined by ^{1}H NMR analysis of unpurified reaction mixture.

3.2. <u>General procedure B:</u> using DCE:DMSO (1:1, v/v) as the solvent for compounds 3n-3ah and 3am-3av in Schemes 3, 4, and 5

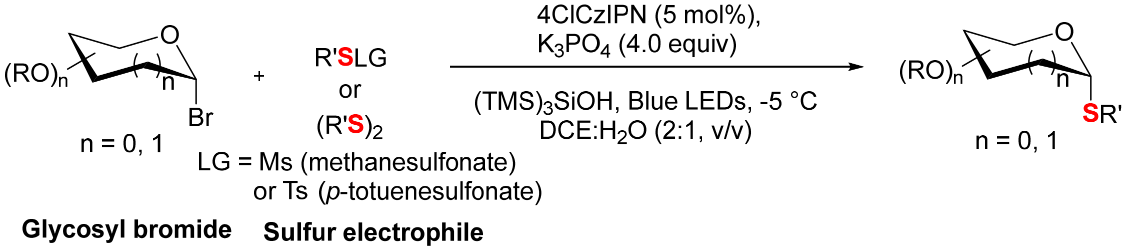

To an oven-dried 20 mL-Schlenk tube equipped with a stir bar, was added glycosyl bromide (0.2 mmol), sulfur electrophiles (0.1 mmol), 4ClCzIPN (5.3 mg, 0.005 mmol), and K_3PO_4 (85 mg, 0.4 mmol). The tube was evacuated and back-filled with N_2, then sealed with rubber stopper and parafilm. Then, the dichloroethane (0.5 mL) and DMSO (0.5 mL) were injected into the tube by a syringe under N_2 atmosphere. The solution was degassed with Freeze-Pump-Thaw Cycling (three times) using the dry ice/acetone as the cooling bath. After the degassing, $(TMS)_3SiOH$ (48 μL, 0.15 mmol) was added through syringe. Subsequently, the solution was stirred at about -5 °C under the irradiation of two 40 W Kessil Blue LEDs for 18-24 h (Figure S1).

After completion of the reaction, the tube was stirred at room temperature for additional 6 hours without the irradiation of blue LEDs to remove the remaining glycosyl bromide. Then, 5 mL of water was added and extracted by ethyl acetate (3 × 5 mL). The combined organic layer was washed with H_2O (5 mL, one time), brine (5 mL, two times) and then dried over anhydrous $MgSO_4$ and evaporated in vacuum. The desired products were obtained in the corresponding yields after purification by flash column chromatography on silica gel eluting with hexane/ethyl acetate or DCM/ethyl acetate in proper ratio. The ratio of α:β was determined by ^{1}H NMR analysis of unpurified reaction mixture.

For compound **3j**, **3ai**, and **3aj**, the workup is same to the general procedure. However, because the side product is hard to separate from the desired product, the additional step was needed to purify the thioglycosides. The detailed procedure was shown as following: the obtained impure product which was purified via flash column chromatography using hexane/ethyl acetate as eluent was dissolved in anhydrous MeOH (3 mL). The catalytic amount of sodium methoxide was added and stirred at room temperature for 6 hours. After the completion of the reaction, directly remove the solvent in vacuum. The obtained product was purified via flash column chromatography using ethyl acetate/MeOH (5:1) as the eluent.

3.3. <u>General procedure C:</u> using DCE:H_2O (2:1, v/v) as the solvent for compounds 3ai-3al in Scheme 4

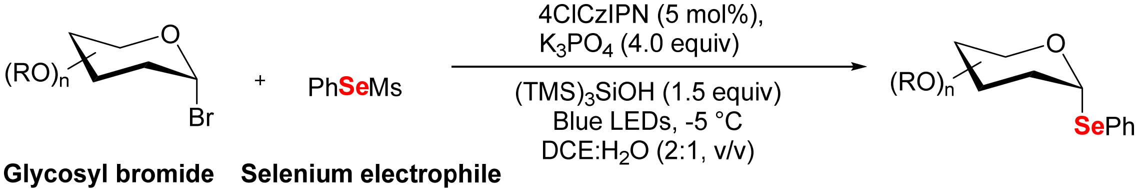

To an oven-dried 20 mL-Schlenk tube equipped with a stir bar, was added glycosyl bromide (0.2 mmol), methyl phenylselenyl sulfone (24 mg, 0.1 mmol), 4ClCzIPN (5.3 mg, 0.005 mmol), and K_3PO_4 (85 mg, 0.4 mmol). The tube was evacuated and back-filled with N_2, then sealed with rubber stopper and parafilm. Then, DCE (1 mL) and distilled H_2O (0.5 mL) were injected into the tube by syringe under N_2 atmosphere. The solution was degassed with Freeze-Pump-Thaw Cycling technique (three times) using the dry ice/acetone as the cooling bath. After the degassing, $(TMS)_3SiOH$ (48 μL, 0.15 mmol) was added through syringe. Subsequently, the solution was stirred at about -5 °C under the irradiation of two 40 W Kessil Blue LEDs for 18-24 h (Figure 1S). After completion of the reaction, first remove the solvent in vacuum directly, and then DCE (1 mL) and DMSO (1 mL) was added, followed by the addition of K_3PO_4 (42 mg, 0.2 mmol), and stirred at room temperature for 6 h without the irradiation of blue LEDs to remove the remaining glycosyl bromide. After the reaction, 10 mL ethyl acetate was added, and the organic phase was washed with H_2O (5 mL, one time), brine (5 mL, two times), and then dried with anhydrous $MgSO_4$, removed the solvent under vacuum. The desired products were obtained in the corresponding yields shown in Scheme 3, 5, and 6 after purification by flash column chromatography on silica gel eluting with hexane/ethyl acetate or ethyl acetate/DCM in proper ratio. The ratio of α:β was determined by ^{1}H NMR analysis of unpurified reaction mixture.

4. Reaction Setup and Measurement of Redox Potential

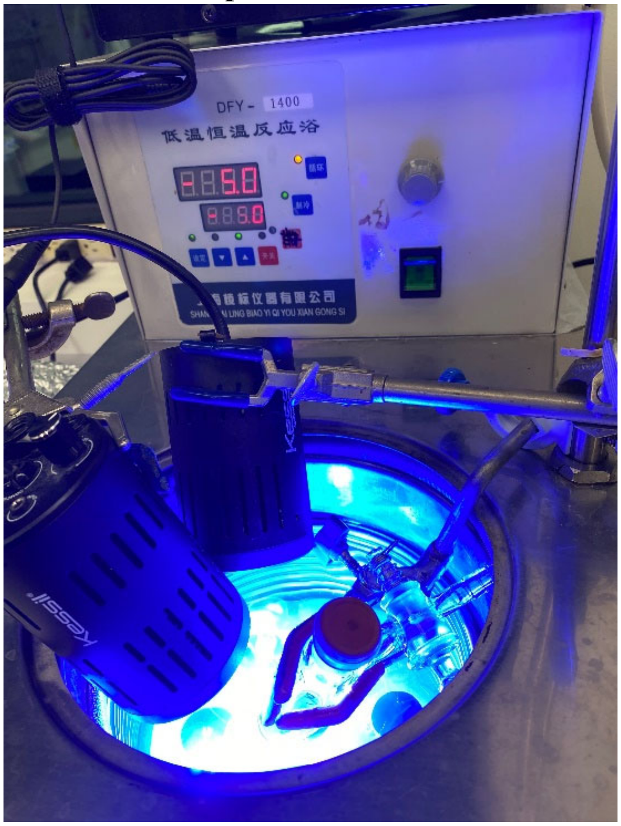

Figure S1. Reaction Setup

Procedure for measurement of the redox potential: Voltammetric measurements were recorded on a CH Instruments: Model 600E Series Electrochemical Analyzer using a standard three electrodes setup in dry and degassed MeCN (10 mL), with ferrocene as an internal reference (E_0 $_{1/2}$ = + 0.40 V vs SCE) and Bu_4NPF_6 as the electrolyte (0.10 mmol). Cyclic voltammograms were recorded at a scan rate of 0.2 V/s.

Comparison of reduction potential between different sugar bromide

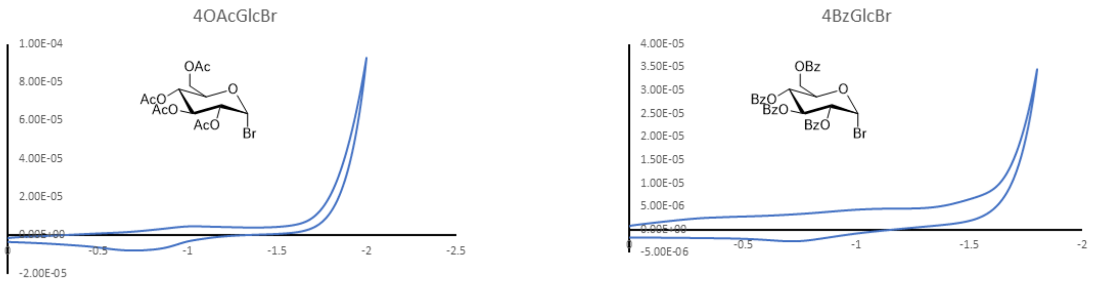

Figure S2. Cyclic voltammetries of 4AcOGlcBr and 4BzGlcBr.

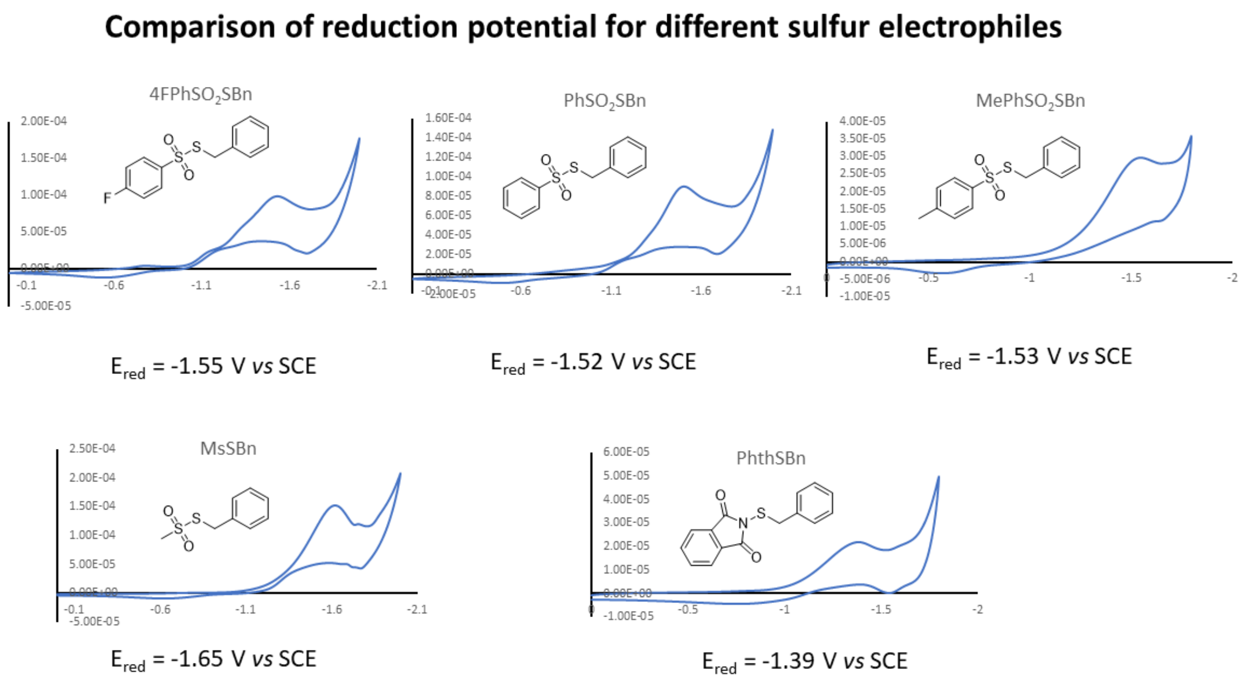

Figure S3. Cyclic voltammetries of thiosulfonates.

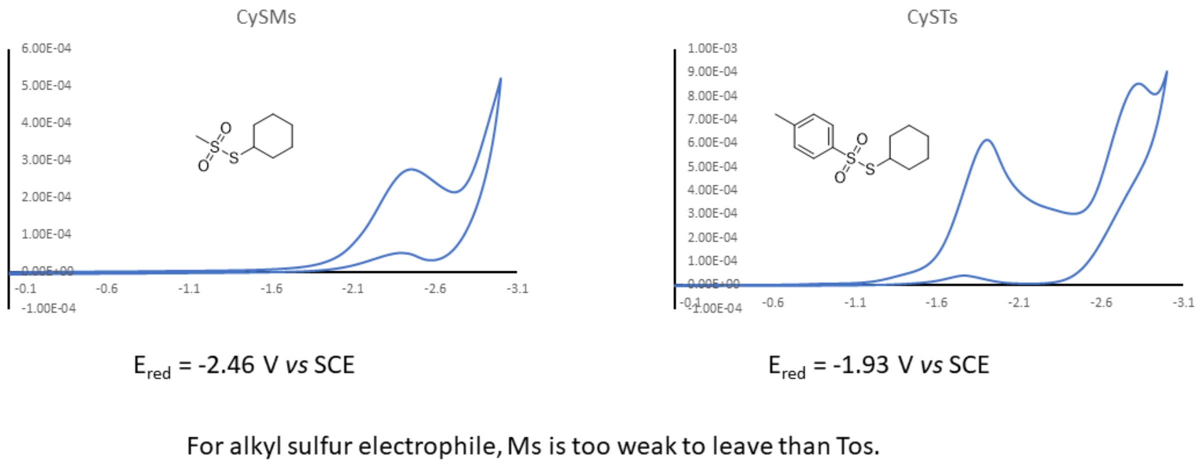

Figure S4. Cyclic voltammetries of alkyl thiosulfonates.

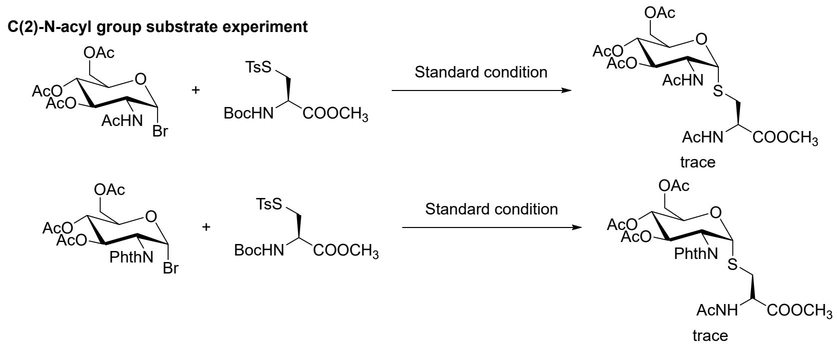

Figure S5. N-acyl glucosamine as the substrate for C-S couping reaction. no desired product formed.

Mechanism studies

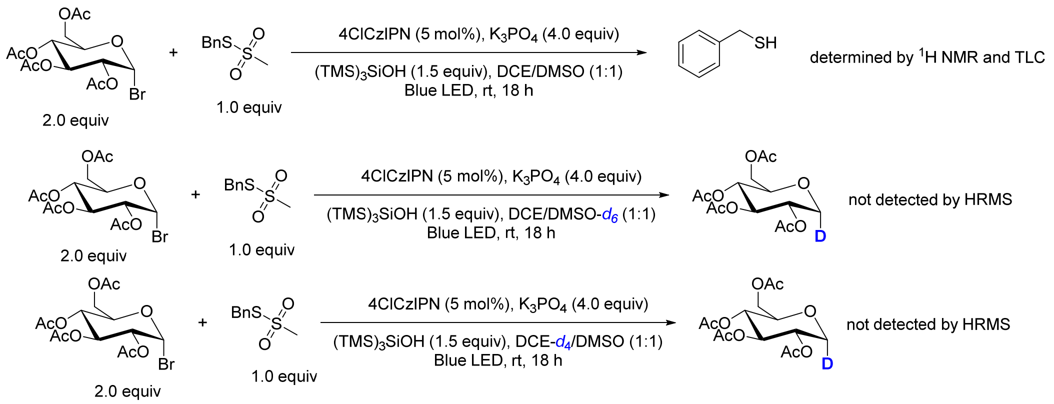

Figure S6. Mechanism studies using the deuterated solvent. The crude product was tested via HRMS, but no desired peak was detected, indicating that the reduced product did not come from the DCE or DMSO.

5. Substrates Synthesis

5.1. Sulfur electrophiles used in the reaction

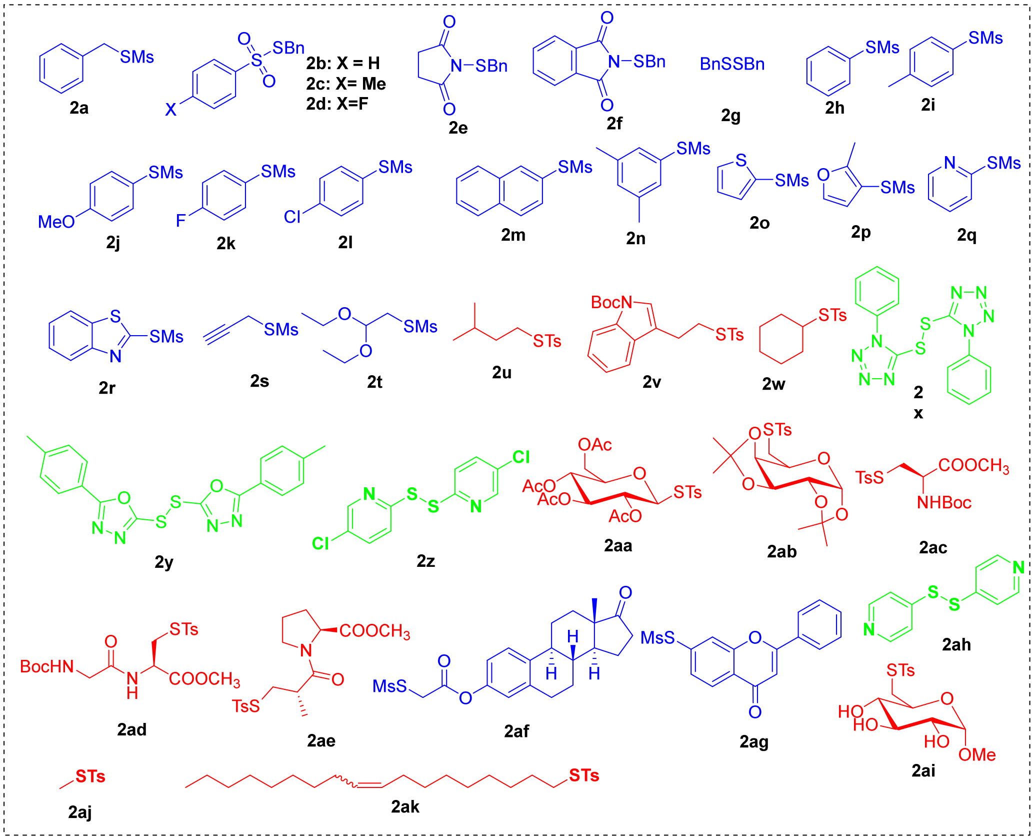

Figure S6. The sulfur electrophiles used in the newly developed C-S coupling reaction

The detailed procedure for synthesis of the representative sulfur electrophiles were demonstrated in **part 5.3**.

5.2. Glycosyl bromides applied in the C-S cross coupling reaction

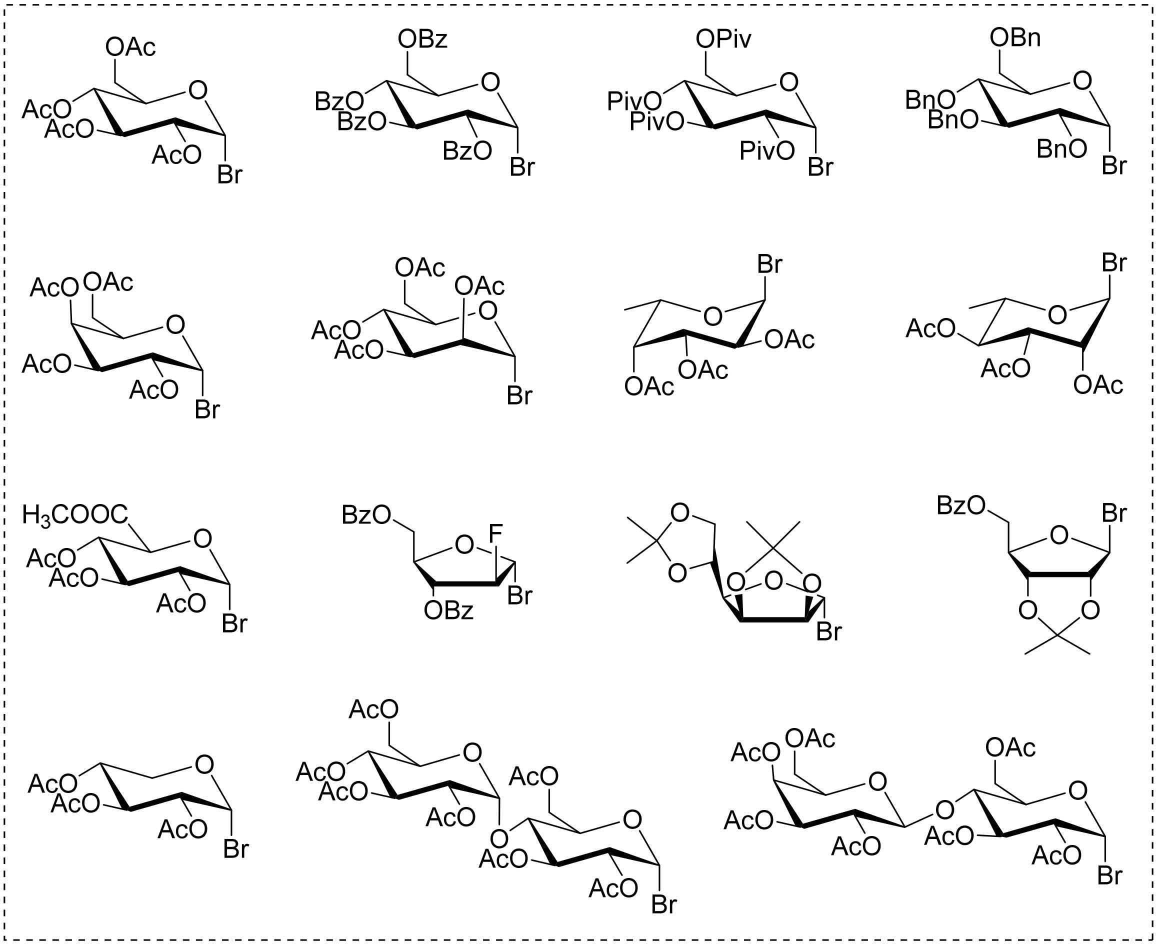

Figure S7. The glycosyl bromide used in the newly developed C-S coupling. The detailed procedure for synthesis of glycosyl bromide were demonstrated in **Part 5.4**.

5.3. General procedure for preparation of thiosulfonates

Scheme S1:

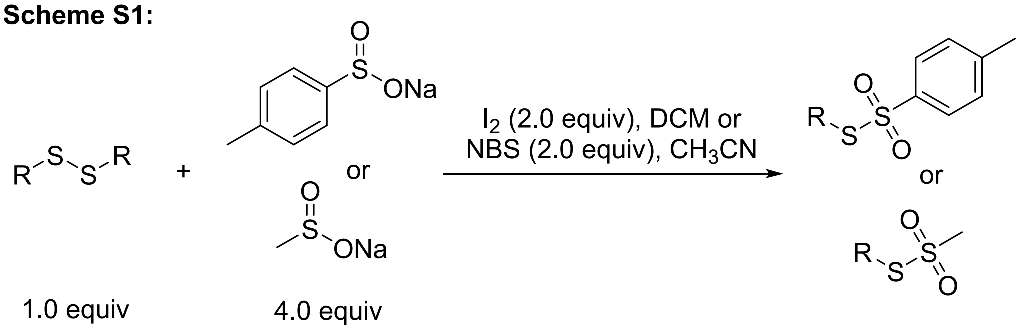

The procedure shown in **Scheme S1** works for **2a, 2b, 2c, 2d, 2h, 2i, 2j, 2k, 2l, 2m, 2n, 2o, 2p, 2q, 2r, 2t, 2u**, **2w**, **2aa**, **2ac**, **2ad**, **2ae**, **2ag**, **2aj**.

To a stirred solution of disulfides (1.0 equiv) in DCM was added sodium *p*-toluenesulfinate (4.0 equiv) and iodine (2.0 equiv) and the mixture was stirred at room temperature overnight. After

the completion of the reaction, the saturated $Na_2S_2O_3$ was added with stirring until the I_2 color disappeared. The organic layer was washed with H_2O and brine, and then dried over $MgSO_4$. After the solvent was removed at reduced, the residue was purified on silica-gel column to afford the desired compound.

For the thiosulfonates with aromatic ring substituted by electron withdrawing group, the iodine was replaced by NBS (2.0 equiv) as shown above. After the completion of the reaction, the reaction solvent was directly removed and purified on silica-gel column to afford the desired compound.

Scheme S2:

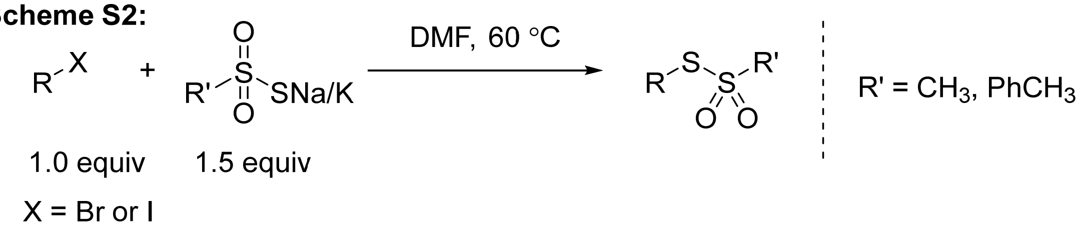

The procedure shown in **Scheme S2** works for the following substrates: **2s**, **2v**, **2ab**, **2af**, **2ai, 2ak**.

The alkyl bromide (10.0 mmol, 1.0 equiv) was dissolved in DMF, and then sodium methanethiosulfonate or potassium *p*-toluenethiosulfonate (15.0 mmol, 1.5 equiv) was added and stirred at 60 °C overnight. After the completion of reaction, the H_2O was poured into the solution and extracted with EA (3 × 10 mL), combined the organic phase and washed with brine (2 × 10 mL). The obtained solution was dried and evaporated in vacuum. The crude product was purified by silica-gel using ethyl acetate/hexane as eluent.

Detailed procedure for synthesis of 2s, 2v, 2aa, 2ab, 2ad, 2ae, 2af, 2ag

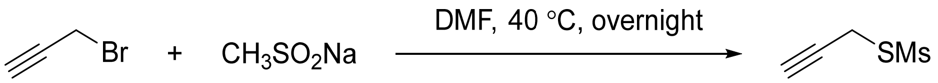

2s

***S*-(prop-2-yn-1-yl) methanesulfonothioate (2s):**

The propargyl bromide (0.262 mL, 1.0 equiv) was dissolved in dry DMF (5 mL), and then the CH_3SO_2Na was added at room temperature. The obtained solution was stirred at room temperature overnight. In the second day, 10 mL H_2O was added and extracted by ethyl acetate (3 × 10 mL). After combination of the organic phase, the solution was dried using $MgSO_4$, and concentrated in vacuum. The crude product was purified by flash column chromatography using hexane/ethyl acetate as eluent. The yield is about 70%.

^{1}H NMR (400 MHz, $CDCl_3$) δ 3.89 (d, *J* = 2.7 Hz, 2H), 3.46 (s, 3H), 2.45 (dd, *J* = 3.2, 2.3 Hz, 1H).

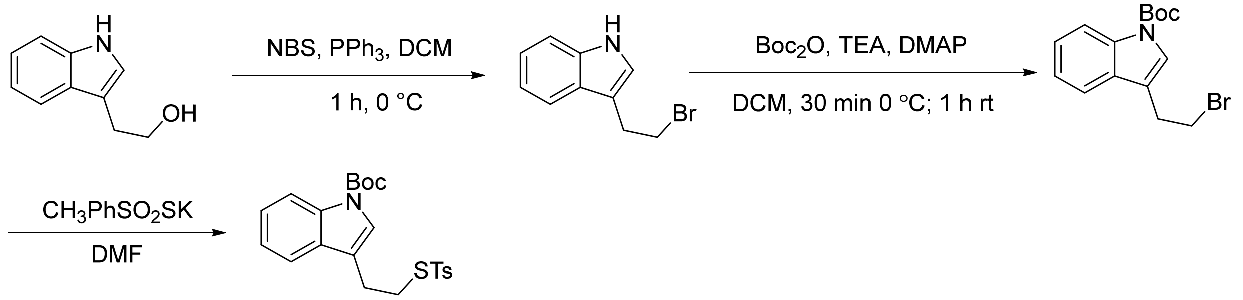

2v

***tert*-Butyl 3-(2-(tosylthio)ethyl)-1*H*-indole-1-carboxylate (2v)**:[1]

Step1: To an ice bath cooled solution of 2-(1*H*-indol-3-yl)ethanol (8.2 g, 51 mmol) and PPh_3 (13.4 g, 51 mmol) in CH_2Cl_2 (20 mL) was added NBS (9.0 g, 51 mmol) in small portions. The mixture was stirred for 1 hour and quenched with water. The organic phase was dried over sodium sulfate and concentrated. The crude material was purified by flash column chromatography (hexane/ethyl acetate = 6:1) to give 3-(2-bromoethyl)indole as a white solid (9.3 g, 82%).

Step2: To a solution of 3-(2-bromoethyl)indole (9.0 g, 40 mmol), Et_3N (8.0 g, 80 mmol) and DMAP (1.0 g, 8 mmol) in CH_2Cl_2 (100 mL) at 0 °C was added $(Boc)_2O$ (9.2 g, 42mmol) in CH_2Cl_2 (50 mL) over 30 minutes. After stirring for 1 h at room temperature, the reaction mixture was washed three times with water, dried over sodium sulfate and concentrated. The crude material was purified by flash column chromatography (hexane/ethyl acetate = 12 : 1) to give 1-Boc-3-(2-bromoethyl)indole as a white solid (12.3 g, 95 %).

Step3: 1-Boc-3-(2-bromoethyl)indole (1.0 equiv) and CH_3PhSO_2SK (1.2 equiv) were dissolved in DMF (10 mL) at room temperature, and stirred overnight. After the completion of reaction, H_2O (10 mL) was added and extracted with ethyl acetate (3 × 10 mL), and then combine the organic phase and remove the solvent in vacuum to get the crude product. The crude product was purified by silica-gel using hexane/ethyl acetate as the eluent to deliver desired product **2v** with yield about 80%.

^{1}H NMR (400 MHz, $CDCl_3$) δ 8.10 (d, *J* = 8.2 Hz, 1H), 7.81 (dd, *J* = 8.3, 2.8 Hz, 2H), 7.43 (d, *J* = 7.8 Hz, 1H), 7.38 – 7.28 (m, 4H), 7.22 (t, *J* = 7.5 Hz, 1H), 3.30 (t, *J* = 7.4 Hz, 2H), 3.03 (t, *J* = 7.5 Hz, 2H), 2.44 (s, 3H), 1.67 (s, 9H).

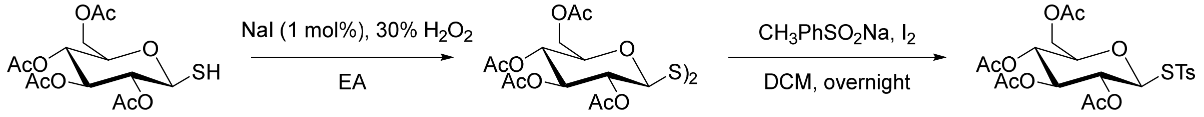

2aa

(2*R*,3*R*,4*S*,5*R*,6*S*)-2-(acetoxymethyl)-6-(tosylthio)tetrahydro-2*H*-pyran-3,4,5-triyl triacetate (2aa):

To the 50 mL round bottom flask, the 1-thio-β-D-glucose tetraacetate (500 mg, 1.0 equiv), NaI (2.0 mg, 0.01 equiv) were added. The 30% H_2O_2 (0.22 mL, 1.0 equiv) was added, and the solution was stirred at room temperature for 30 min. After the completion of the reaction, directly remove the solvent to get the crude product. The obtained product was dissolved in DCM (10 mL),

and then the CH_3PhSO_2Na (489.02 mg, 4.0 equiv) and iodine (348.29 mg, 2.0 equiv) was added. The obtained solution was stirred at room temperature overnight. After the completion of the reaction, the saturated NaS_2O_3 (10 mL) was added and stirred for 20 min. The resulting solution was extracted by DCM (2 × 10 mL), and combine the organic phase, dry the solution by $MgSO_4$, evaporate the solvent in vacuum. The crude product was purified by flash column chromatography using hexane/ethyl acetate as eluent. The desired product was delivered as white solid (80%).

^{1}H NMR (400 MHz, $CDCl_3$) δ 8.30 – 7.68 (m, 2H), 7.52 – 7.22 (m, 2H), 5.48 – 5.14 (m, 2H), 5.11 – 4.85 (m, 2H), 4.25 – 4.01 (m, 2H), 3.79 – 3.59 (m, 1H), 2.51 – 2.29 (m, 3H), 2.14 – 1.79 (m, 12H).

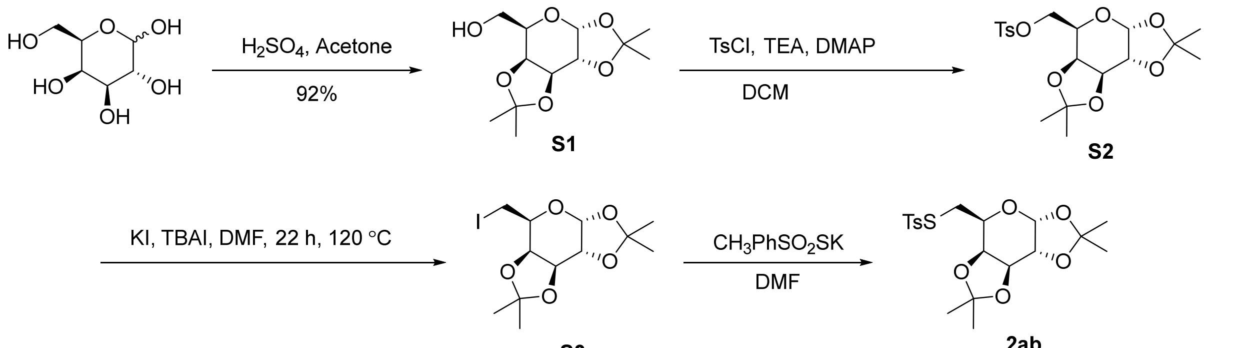

S1 S2 S3 **2ab**

S-(((3a*R*,5*S*,5a*R*,8a*S*,8b*R*)-2,2,7,7-tetramethyltetrahydro-5*H*-bis([1,3]dioxolo)[4,5-*b*:4',5'-*d*]pyran-5-yl)methyl) 4-methylbenzenesulfonothioate

_S_-(((3a_R_,5_S_,5a_R_,8a_S_,8b_R_)-2,2,7,7-tetramethyltetrahydro-5_H_-bis([1,3]dioxolo)[4,5-b:4',5'-d]pyran-5-yl)methyl) 4-methylbenzenesulfonothioate (2ab):[2]

Step1: Concentrated H_2SO_4 (10 mL) was added dropwise to acetone (350 mL) with stirring in ice-water bath, followed by D-galactopyranose (10 g, 55.5 mmol, 1.0 equiv) portionwise. The mixture was stirred for 7 h at room temperature until the starting material reacted completely and then neutralized with aqueous $NaHCO_3$ to pH = 8-9 with stirring in ice water bath, the acetone was evaporated. The aqueous layer was extracted with EtOAc and the combined organic layers were washed with brine, dried over Na_2SO_4. After evaporation of the solvent, the residue was purified by silica gel chromatography (hexane/EA = 2:1) to give **S1** (13.0 g, 92% yield) as a colorless oil.

Step2: A solution of **S1** (13.0 g, 50.0 mmol, 1.0 equiv) in DCM (63 mL) was cooled to 0 °C and p-toluenesulfonyl chloride (10.5 g, 55.0 mmol, 1.1 equiv), Et_3N (13.9 mL, 100.0 mmol, 2.0 equiv), DMAP (1.2 g, 10.0 mmol, 0.2 equiv) was added sequentially. The mixture was stirred for 20 min at room temperature and then diluted with water. The aqueous layer was extracted with EtOAc and the combined organic layers were washed with brine, dried over Na_2SO_4. After evaporation of the solvent, the residue was purified by silica gel chromatography (hexane/EA = 2:1) to give **S2** (18.2 g, 88% yield) as a white solid.

Step3: To a solution of **S2** (18.2 g, 44.0 mmol, 1.0 equiv) in DMF (30 mL) was sequentially added TBAI (8.13 g, 22.0 mmol, 1.5 equiv) and KI (11.0 g, 66.0 mmol, 0.5 equiv). The mixture was stirred for 22 h at 120 °C and then cooled to room temperature, poured into ice water, extracted with EtOAc, and the organic layer was washed with saturated aqueous $Na_2S_2O_3$ and brine, dried over Na_2SO_4. After evaporation of the solvent, the residue was purified by silica gel

chromatography (hexane/EA = 15:1) to give **S3** (15.0 g, 93% yield) as a white solid. R_f = 0.55 (Hexane/EA = 10:1).

Step4: To a solution of **S3** (258 mg, 0.69 mmol, 1 equiv) in DMF (2 mL) was added potassium *p*-toluenethiosulfonate (285 mg, 1.38 mmol, 2 equiv) and the reaction was stirred at 60 °C for 16 h before being quenched by saturated NaCl solution (20 mL). The mixture was extracted by DCM (3 × 10 mL) and combined organic was dried by Na_2SO_4. After evaporation of the solvent, the residue was purified by silica gel chromatography (hexane/EA= 3:1) to give **2ab** (252 mg, 91% yield). The ^{1}H NMR data is consistent with the literature report.[2]

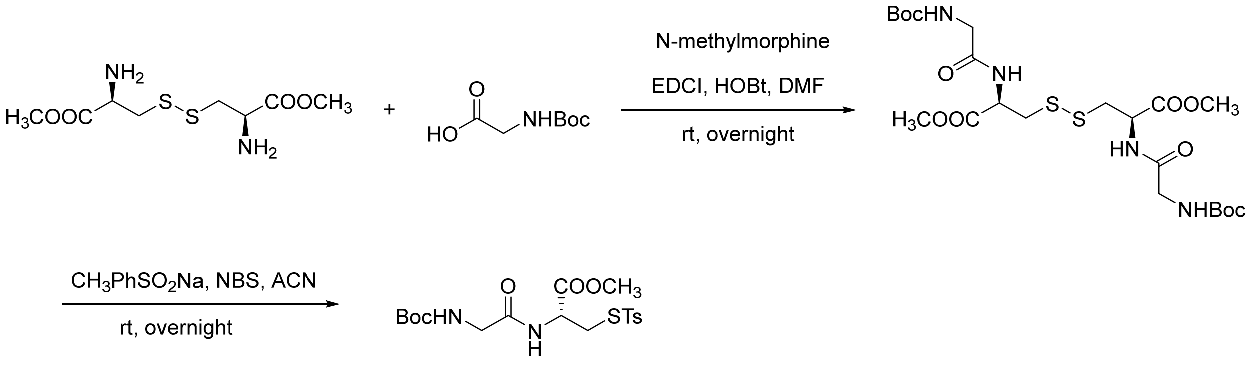

methyl *N*-((*tert*-butoxycarbonyl)glycyl)-*S*-tosyl-*L*-cysteinate (**2ad**)

Methyl *N*-((*tert*-butoxycarbonyl)glycyl)-*S*-tosyl-*L*-cysteinate (2ad)[3]

Step 1: To a round bottom flask was added *N*-Boc-glycine (385.4 mg, 2.2 equiv) and DMF 7.5 mL, and the solution was cooled to 0 °C with iced bath for 15 min. 1-Hydroxybenzotriazole (297.3 mg, 2.2 equiv) was added until the active ester formation by TLC monitor. The *N*-ethyl-*N′*-(3-dimethylaminopropyl)carbodiimide hydrochloride (421.7 mg, 2.2 equiv) was added over 15 min. Finally, the cystine (341.6 mg, 1.0 equiv) and *N*-methylmorphine (0.242 mL) was added at 0 °C for 40 min. After that, the iced bath was removed and stirred at room temperature overnight. After the completion of the reaction, ethyl acetate (50 mL) was added, washed with H_2O. After removal of the solvent, the crude product was purified via column chromatography using DCM/MeOH (10:1) as eluent with yield about 80%.

Step 2: The protected cystine (470 mg, 1.0 equiv) obtained from above procedure was dissolved in acetonitrile (8 mL), and then NBS (290 mg, 2.0 equiv) and $MePhSO_2Na$ (575 mg, 4.0 equiv) was added at room temperature and stirred overnight. After the completion of the reaction, the solvent was removed directly and purified by DCM/MeOH to get the desired product **2ad** with yield about 50%.

^{1}H NMR (400 MHz, $CDCl_3$) δ 8.01 – 7.67 (m, 2H), 7.33 (d, *J* = 8.0 Hz, 2H), 7.14 (d, *J* = 7.6 Hz, 1H), 5.47 – 5.13 (m, 1H), 4.83 (dt, *J* = 7.5, 5.1 Hz, 1H), 3.83 (dd, *J* = 12.6, 5.9 Hz, 12H), 3.72 (s, 3H), 3.44 (qd, *J* = 14.6, 5.0 Hz, 2H), 2.43 (s, 3H), 1.43 (s, 9H).

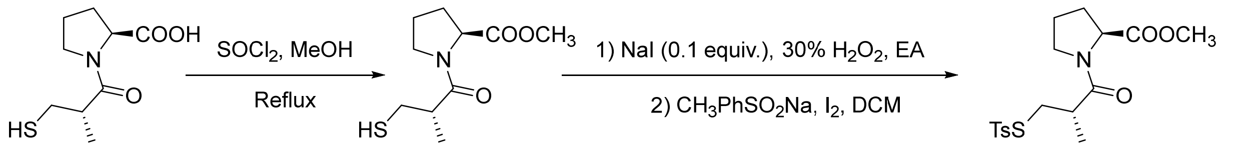

Captopril

methyl ((*S*)-2-methyl-3-(tosylthio)propanoyl)-*L*-prolinate (**2ae**)

Methyl ((*S*)-2-methyl-3-(tosylthio)propanoyl)-L-prolinate (2ae)[4]

Step 1: Captopril (1.09 g, 5 mmol) was dissolved in methanol (25 mL). Thionyl chloride (1.0 mL, 13.5 mmol) was then added dropwise at 0 °C. The reaction mixture was heated at 60 °C for 4 h. The excess alcohol and thionyl chloride produced hydrogen chloride gas was removed in-vacuo to yield the crude ester. The crude product was purified by flash column chromatography on silica gel. The light yellow oil liquid was obtained in 82% yield.

Step 2: The ester of Captopril (243 mg) was dissolved in ethyl acetate (5 mL), and then NaI (3.24 mg, 0.01 equiv), 30% H_2O_2 (0.24 mL, 1.0 equiv) was added. Stirred at room temperature for 1 h. One hour later, the ethyl acetate was removed in vacuum. The obtained crude product was dissolved in DCM (10 mL), and then sodium *p*-tolylsulfinate (750 mg, 4.0 equiv), iodine (534 mg, 2.0 equiv) was added and stirred at room temperature overnight. After completion of the reaction, the saturated $Na_2S_2O_3$ was added until the color disappearance. And then, extraction using DCM (2 × 10 mL). and combine the organic phase and evaporate in vacuum. The obtained crude was purified using silica-gel and gave the desired product **2ae** the yield is about 70%.

^{1}H NMR (400 MHz, $CDCl_3$) δ 8.13 – 7.71 (m, 2H), 7.34 (d, *J* = 8.0 Hz, 2H), 4.49 (dd, *J* = 8.6, 4.0 Hz, 1H), 3.70 (s, 3H), 3.58 (t, *J* = 6.6 Hz, 2H), 3.25 (dd, *J* = 13.8, 9.1 Hz, 1H), 3.18 – 3.04 (m, 1H), 2.88 (dd, *J* = 13.9, 4.9 Hz, 1H), 2.45 (s, 3H), 2.37 – 2.15 (m, 1H), 2.13 – 1.89 (m, 3H).

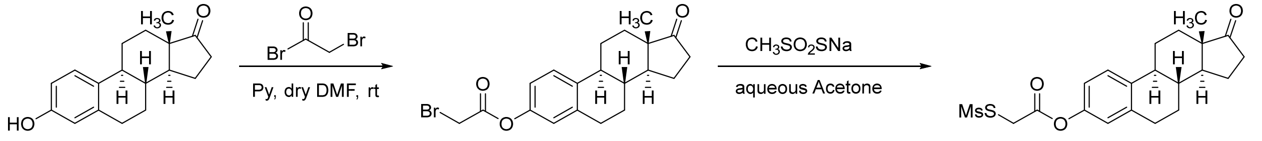

(8*R*,9*S*,13*S*,14*S*)-13-methyl-17-oxo-7,8,9,11,12,13,14,15,16,17-decahydro-6*H*-cyclopenta[*a*]phenanthren-3-yl 2-((methylsulfonyl)thio)acetate
2af

(8*R*,9*S*,13*S*,14*S*)-13-methyl-17-oxo-7,8,9,11,12,13,14,15,16,17-decahydro-6*H*-cyclopenta[*a*] phenanthren-3-yl 2-((methylsulfonyl)thio)acetate (2af)[5]

Step 1: The 1,3,5(10)-estratrien-3-ol-17-one (500 mg) was dissolved in dry DMF, and cooled to 0 °C, after that, the pyridine (0.149 mL), bromoacetyl bromide (0.161 mL) were added at 0 °C, respectively. Stirred at 0 °C for 15 min. And then it was stirred at room temperature for 20 min. H_2O (10 mL) was added and extracted with DCM (3 × 10 mL), combined the organic phase and evaporated in vacuum. The obtained crude will be purified using silica-gel by hexane/dichloromethane/ethyl acetate (6:3:1) as eluent.

Step 2: The obtained compound (300 mg) from above step was dissolved in aqueous acetone (acetone : H_2O = 5:1, 5 mL), and the CH_3SO_2SNa (113 mg) was added in one portion.

After 6 hours, the solvent was removed and purified directly through silica-gel using hexane/dichloromethane/ethyl acetate (5:4:1) as eluent, and the yield of **2af** is about 70%.

^{1}H NMR (400 MHz, $CDCl_3$) δ 7.32 (d, *J* = 8.5 Hz, 1H), 6.89 (d, *J* = 8.9 Hz, 1H), 6.85 (s, 1H), 4.20 (s, 2H), 3.50 (s, 3H), 2.92 (dd, *J* = 9.5, 4.4 Hz, 2H), 2.51 (dd, *J* = 18.7, 8.5 Hz, 1H), 2.40 (d, *J* = 9.8 Hz, 1H), 2.31 (d, *J* = 11.6 Hz, 1H), 2.22 – 1.93 (m, 4H), 1.54 (ddt, *J* = 31.8, 22.9, 11.7 Hz, 6H), 0.91 (s, 3H).

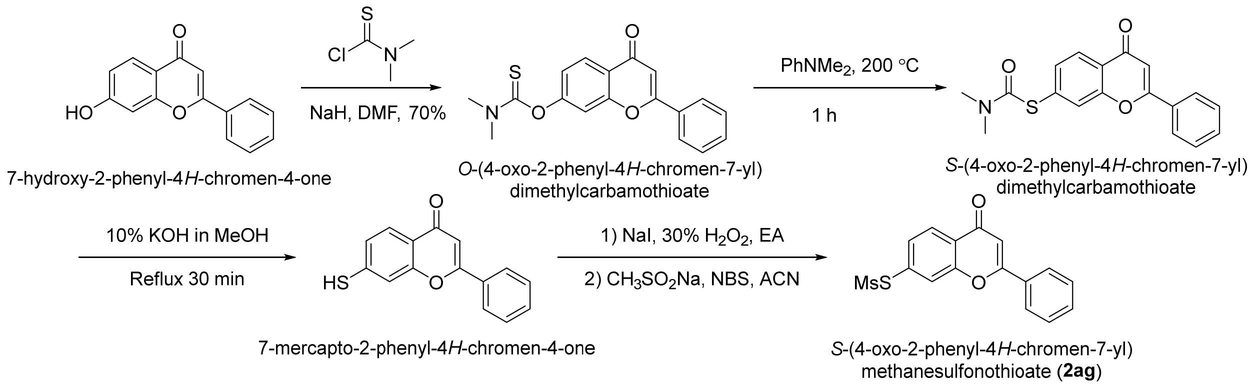

***S*-(4-oxo-2-phenyl-4*H*-chromen-7-yl) methanesulfonothioate (2ag)**[6]

Step 1: The 7-hydroxy-2-phenyl-4*H*-chromen-4-one (1.0 g, 1.0 equiv) was dissolved in dry DMF (15-20 mL), then NaH (200 mg, 1.2 equiv) was added under N_2 atmosphere. After the no release of H_2, the solution was cooled to 0 °C, and then dimethylcarbamothioic chloride (778 mg, 1.5 equiv) was added and then the reaction was heated to 80 °C for 1 h. after the completion of the reaction, the reaction was warmed to room temperature, and then poured into 30 mL cold H_2O. after the filtration, the crude product was purified by silica-gel using DCM as the eluant and give desired product about 70% yield.

Step 2: The obtained *O*-(4-oxo-2-phenyl-4*H*-chromen-7-yl) dimethylcarbamothioate (0.5 g) was dissolved in *N*, *N*-dimethylaniline 5 mL, and reflux for 1 h. After the completion of the reaction, the solution was poured into 10% HCl (20 mL). After filtration of the precipitate, washed with acid and crystallized from MeOH to get the desired product in 50%.

Step 3: The *S*-(4-oxo-2-phenyl-4*H*-chromen-7-yl) dimethylcarbamothioate (300 mg) was dissolved in 10% KOH in MeOH and refluxed for 30 min. After the completion of the reaction, water was added and triturated, washed with Et_2O. And then the aqueous phase was acidified with HCl. The formed precipitate was filtered off. Crystallized from MeOH and get the desired product about 80%.

Step 4: The obtained 7-mecapto-2-phenyl-4*H*-chromen-4-one (250 mg, 1.0 equiv) was dissolved in ethyl acetate (4 mL), and then NaI (2.0 mg, 0.01 equiv), H_2O_2 (0.11 mL, 1.0 equiv) was added. After 1 hours, the solvent was removed directly. The crude was dissolved in acetonitrile (5-10 mL), and then NBS (360 mg), CH_3SO_2Na (410 mg) was added and stirred at rt overnight. After the completion of the reaction, directly remove the solvent and purify by silica-gel using DCM/ethyl acetate (20:1) (60%).

^{1}H NMR (400 MHz, DMSO-d_6) δ 8.26 (s, 1H), 8.20 – 8.09 (m, 3H), 7.79 (d, *J* = 8.2 Hz, 1H), 7.61 (q, *J* = 7.6, 6.7 Hz, 3H), 7.15 (s, 1H), 3.55 (s, 3H).

5.4. Procedures for the synthesis of glycosyl bromides

Rxn1:

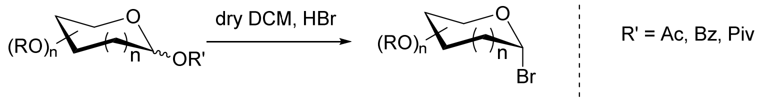

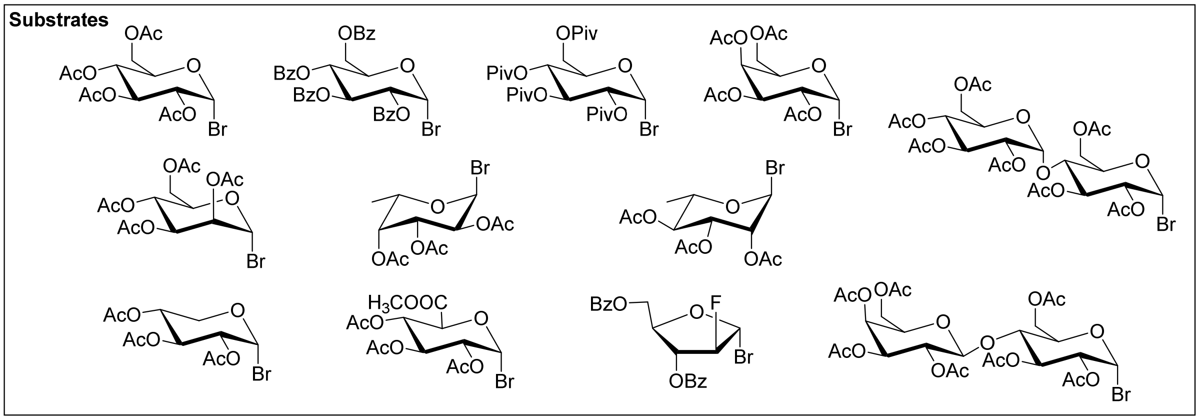

Rxn2:

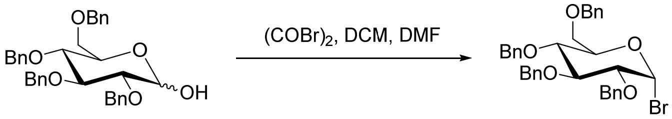

Rxn3:

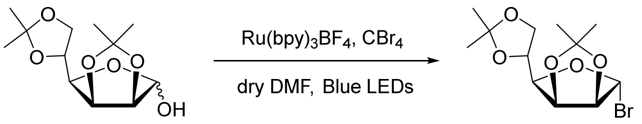

Rxn4:

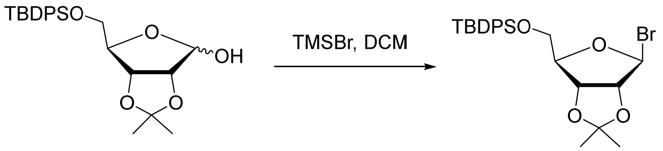

General procedure for Rxn1: The substrates shown in the Rxn1 were prepared according to the reported literature. The Ac/Piv/Bz protected sugar was added in DCM (10 mL), and cooled to 0 °C under N_2 atmosphere. The 33% HBr in HOAc was added at 0 °C. The iced bath was removed and stirred at room temperature. The reaction was stirred at room temperature until the reactant disappeared. After the completion of the reaction, the solution was poured into iced H_2O, and diluted with DCM. The organic phase was washed with saturated $NaHCO_3$, brine, and dried by Na_2SO_4 and evaporated in vacuum. The obtained crude product was purified through silica-gel using hexane/EA as eluent.

For example, synthesis of 2,3,4,6-tetraacetyl-α-D-galactosyl bromide:[7] To a solution of pentaacetyl-β-D-galactose (1.0 g, 2.56 mmol) in CH_2Cl_2 (10 mL) was added 33% HBr/AcOH (1.35 mL, 7.68 mol) at 0 °C. The mixture was stirred at 0 °C and was then allowed to gradually warm up to room temperature and stirred overnight. The reaction was quenched with CH_2Cl_2 (20 mL). The mixture was washed with H_2O (20 mL × 1) and $NaHCO_3$ (20 mL × 2). The organic layer was dried with anhydrous $MgSO_4$, filtered and evaporated under reduced pressure to obtain crude compound. The crude compound was purified by flash column chromatography on silica-gel using ethyl acetate/hexane (1:5) as the eluent to obtain desired product as a colorless oil (89%).

Procedure for Rxn2.[8] 2,3,4,6-tetra-*O*-benzyl glucopyranose (0.40 g, 0.74 mmol) was dissolved in a mixture of dry dichloromethane (2.40 mL) and dry DMF (1.36 mL) under an atmosphere of nitrogen. Oxalyl bromide (1.11 mL, 2.22 mmol, 2.0M solution in DCM) was added slowly via syringe. Stirring was continued at room temperature for one hour. The solvent was then removed in vacuo and the residue taken up in DCM (10 mL). The DCM solution was washed with saturated aqueous sodium bicarbonate (2 × 10 mL) to render it alkaline and then washed with water (1 × 10 mL) and brine (1 × 10 mL). The organic layer was dried over $MgSO_4$ and the solvent evacuated in vacuo. The resulting residue was taken up in toluene and then evaporated under high vacuum. The glycosyl bromide was used either immediately or stored at -20 °C for up to two weeks.

Procedure for Rxn3.[9] A dry 25 mL Schlenk tube was charged with 2,3,5,6-bis-*O*-(1-methylethylidene)-D-mannofuranose (1.0 mmol), CBr_4 (2.0 mmol, 2.0 equiv), $Ru(bpy)_3(BF_4)_2$ (0.05 mmol, 0.05 equiv), and tetra-n-butylammonium bromide (2.0 mmol, 2.0 equiv) in anhydrous DMF (5 mL). The tube was sealed with a rubber septum and the solution was degassed by three freeze-pump-thaw cycles under argon. The tube was placed approximately 5 cm from the irradiation source (blue LEDs, 40 W), and the mixture was stirred and irradiated for 10-15 h until the starting alcohol was completely consumed (TLC). Et_2O (25 mL) and H_2O (25 mL) were added, the layers were separated, and the aqueous layer was extracted with Et_2O (2 × 25 mL). The organic layers were combined, washed with sat. aq $Na_2S_2O_3$, dried by Na_2SO_4, and concentrated in vacuum. The residue was purified by chromatography (silica gel). Use hexane/EA (10:1) as the eluent.

Procedure for Rxn4.[10] To a solution of (3a*S*,6*R*,6a*S*)-6-((*tert*-butyldiphenylsilyloxy)methyl)-2,2-dimethyltetrahydrofuro[3,4-*d*][1,3]dioxol-4-ol (236 mg, 0.55 mmol) in dry CH_2Cl_2 (5.5 mL) at 0 °C was added bromo-trimethylsilane (0.15 mL, 1.1 mmol, 2.0 equiv). After 2 h at room temperature, the solvent was removed under reduced pressure, and the product was eluated through a pad of silica gel (hexane/Et_2O, 90/10) to yield (((3a*S*,4*R*,6a*S*)-6-bromo-2,2-dimethyl-tetrahydrofuro[3,4-*d*]-[1,3]dioxol-4-yl)methoxy)(*tert*-butyl)diphenylsilane which was directly engaged in the C-S coupling reaction. (174 mg, 0.26 mmol, 64%).

5.5. Procedure for preparation of $(TMS)_3SiOH/D$[11]

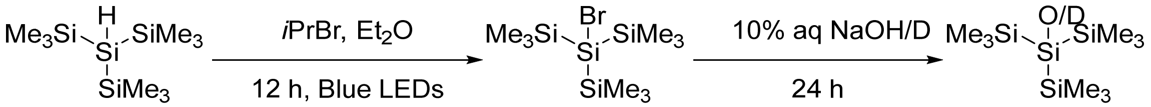

Under air, a 40-mL vial equipped with a magnetic stir bar was charged with TMS_3SiH (3.6 mL, 12.5 mmol), 2-bromopropane (2.3 mL, 25.0 mmol, 2.0 eq) and Et_2O (4.0 mL). The reaction vial was capped under air and irradiated with Kessil 40 W blue LEDs (7 cm away, with fan cooling) for 12 hours. After irradiation, the reaction vial was slowly opened to allow for a slow gas evolution. After gas evolution completed, the organic solution was poured into a round-bottom flask containing a 10% aq NaOH solution (11 mL, 1.1 eq). More Et_2O is used to ensure complete transfer. This mixture was stirred at room temperature under air for 24 hours. Et_2O was then added and the organic layer was separated. The organic solution was dried with Na_2SO_4, followed by concentration to yield the crude silanol as a clear oil. Purification via distillation under high vac yielded the pure silanol as a clear oil. The purity of the silane reagent was confirmed by ^1H NMR.

^{1}H NMR (400 MHz, CD_3CN) δ 1.81 (s, 1H), 0.16 (s, 27H).

Using the same procedure above, just use 10% aq NaOD solution replace the 10%. The obtained $(TMS)_3SiOD$ was used directly for the mechanism studies.

^{1}H NMR (400 MHz, $CDCl_3$) δ 0.18 (s, 27H).

5.6. Synthesis of 4ClCzIPN[12]

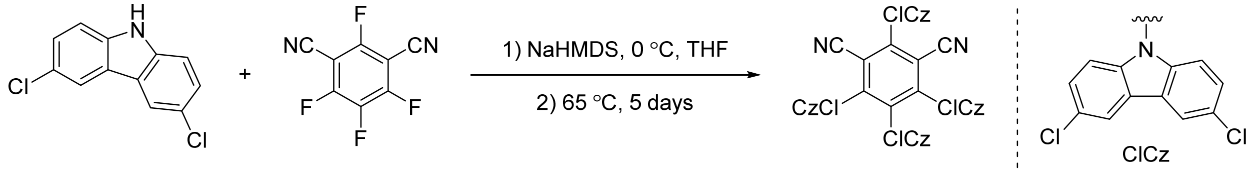

The 3,6-dichlorocarbazole (1.0 g, 4.4 equiv) was added to 2-necked round-bottom flask, then evacuated 5 min and back-filled nitrogen three times. Then the THF (20 mL) was added and then the flask was cooled to 0 °C. 2.0 M NaHMDS (2.06 mL, 4.2 equiv) was added dropwise over 5 min. The reaction was stirred at room temperature for 30 min after the addition of base. Then then solution of tetrafluoroisophthalonitrile (192.6 mg, 1.0 equiv) in THF (10 mL) was added. The reaction was heated up to 65 °C for 5 days. After the completion of the reaction, first remove the solvent and then purified by flash clumn chromatography using DCM as eluent. The obtained product could be further purifed by crystallization using Et_2O as eluent followed by filtration to deliver the light yellow solid.

^{1}H NMR (400 MHz, DMSO-d_6) δ 8.59 (d, J = 2.2 Hz, 2H), 8.11 (dd, J = 20.3, 5.4 Hz, 6H), 7.96 – 7.85 (m, 2H), 7.78 (d, J = 2.2 Hz, 2H), 7.69 (d, J = 8.6 Hz, 4H), 7.46 (d, J = 8.9 Hz, 2H), 7.36 – 7.26 (m, 4H), 6.99 – 6.85 (m, 2H).

6. Synthesis of 3l in Gram Scale

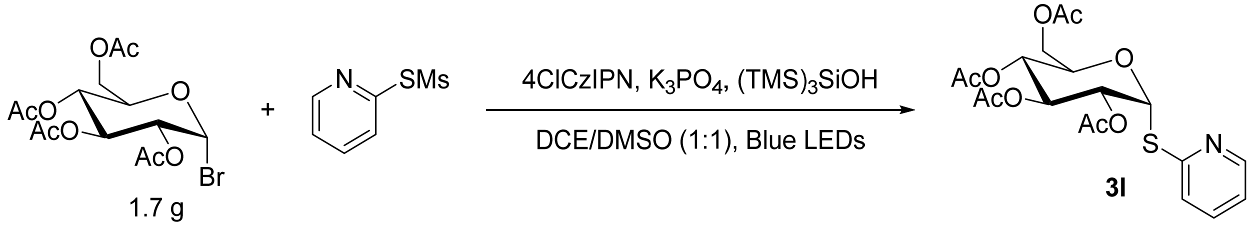

To an oven-dried 100 mL-Schlenk tube equipped with a stir bar, was added glucosyl bromide (1.7 g, 4.0 mmol), sulfur electrophiles (380 mg, 2 mmol), 4ClCzIPN (108 mg, 0.1 mmol), and K_3PO_4 (1.7 g, 4.0 mmol). The tube was evacuated and back-filled with N_2, then sealed with rubber stopper and parafilm. Then, the dichloroethane/DMSO (1:1) was added using a syringe. The solution was degassed with Freeze-Pump-Thaw Cycling (three times). After the degassing, $(TMS)_3SiOH$ (0.96 mL, 3.0 mmol) was added through syringe. Subsequently, the solution was stirred at about -5 °C under the irradiation of two 40 W Kessil Blue LEDs for 24 h (Figure 1S). After completion of the reaction, 20 mL water was added and extracted by ethyl acetate (3 × 20 mL). The combined organic layer was washed with brine (20 mL, three time) and then dried over anhydrous $MgSO_4$ and evaporated in vacuum. The desired products were obtained in the corresponding yields after purification by flash chromatography on silica gel eluting with ethyl acetate/dichloromethane (20:1), the desired product was obtained as the white solid (610 mg, 68%).

7. Glycosylation Reaction Using Thioglycoside 3v as the Glycosyl Donor

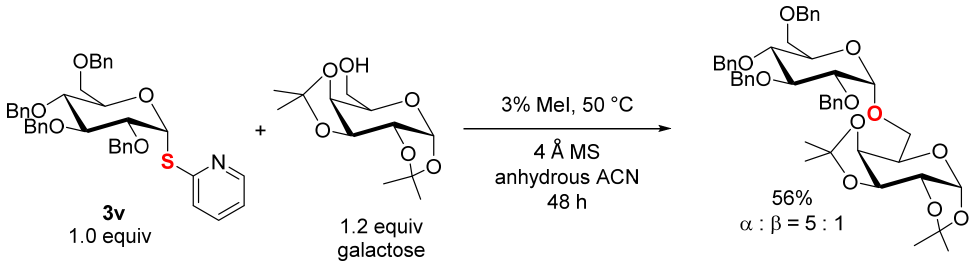

To an oven-dried 25 mL round flask equipped with a stir bar, was added the thioglycoside **3v** (30 mg, 0.047 mmol), galactose (15 mg, 0.056 mmol), and 4 Å powdery molecular sieve (20 mg). The tube was evacuated and back-filled with N_2, then sealed with rubber stopper and parafilm. The anhydrous acetonitrile (2 mL containing 3% methyl iodide) was injected into the flash through the syringe. The flask was heated to 50 °C for 48 h. After the completion of the reaction, directly remove the solvent and purified the product using hexane/ethyl acetate (5:1). The ratio of α/β anomer was calculated via the impure product after removal of galactose since the peak of galactose will overlap with the β of desired product. The ^{1}H NMR of desired product is consistent with the literature.[13]

^{1}H NMR (400 MHz, $CDCl_3$) δ 7.58 – 7.20 (m, 18H), 7.14 (dd, *J* = 7.0, 2.4 Hz, 2H), 5.52 (d, *J* = 5.0 Hz, 1H), 5.08 – 4.94 (m, 2H), 4.81 (t, *J* = 10.8, 9.0 Hz, 2H), 4.72 (q, *J* = 11.9 Hz, 2H), 4.65 – 4.57 (m, 2H), 4.48 (dd, *J* = 11.6, 6.7 Hz, 2H), 4.36 (dd, *J* = 8.0, 1.9 Hz, 1H), 4.31 (dd, *J* = 5.1, 2.4 Hz, 1H), 4.10 – 4.02 (m, 1H), 3.99 (t, *J* = 9.2 Hz, 1H), 3.88 – 3.63 (m, 6H), 3.59 (dd, *J* = 9.6, 3.7 Hz, 1H), 1.54 (s, 3H), 1.46 (s, 3H), 1.33 (s, 3H), 1.32 (s, 3H).

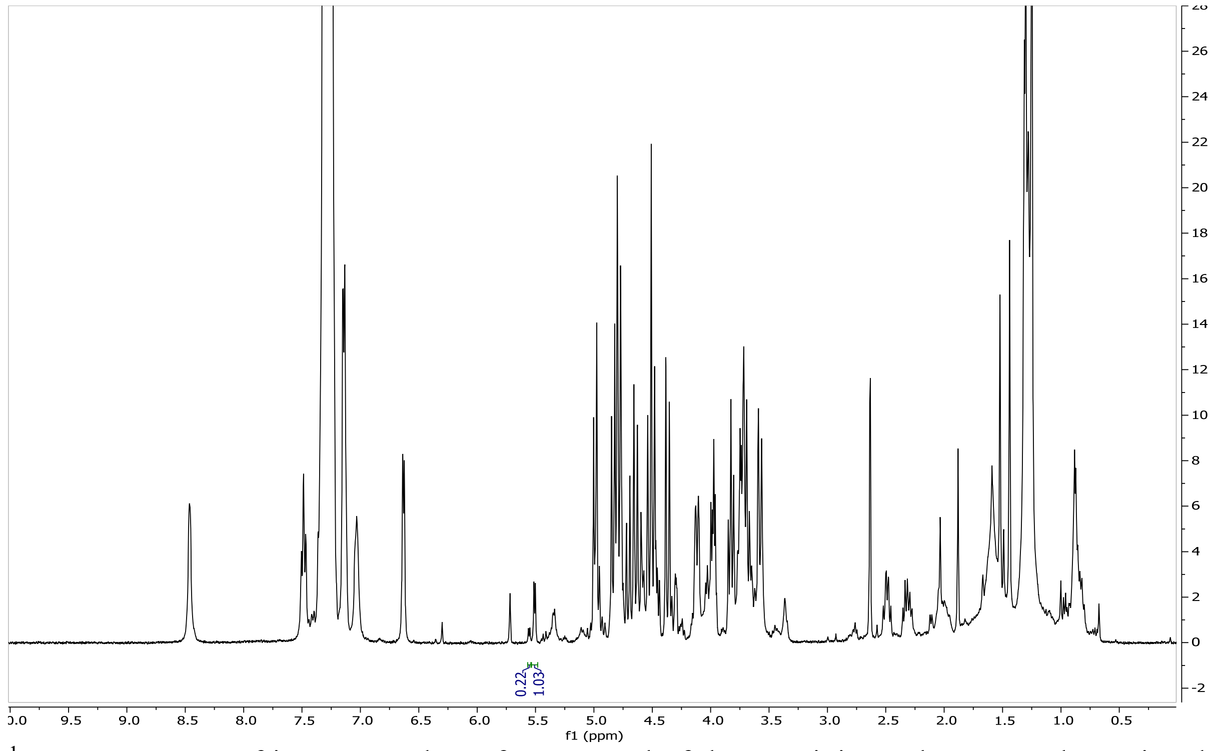

^{1}H NMR spectra of impure product after removal of the remaining galactose to determine the ratio of α/β.

8. References

[1] Yang, J.; Song, H.; Xiao, X.; Wang, J.; Qin, Y. Biomimetic Approach to Perophoramidine and Communesin via an Intramolecular Cyclopropanation Reaction. *Org. Lett.* **2006**, *8*, 2187-2190.

[2] Zeng, J.; Sun, G.; Yao, W.; Zhu, Y.; Wang, R.; Cai, L.; Liu, K.; Zhang, Q.; Liu, X.-.W.; Wan, Q. 3-Aminodeoxypyranoses in Glycosylation: Diversity-Oriented Synthesis and Assembly in Oligosaccharides. *Angew. Chem. Int. Ed.* **2017**, *56*, 5227-5231

[3] Verhoog, S.; Kee, C. W.; Wang, Y.; Khotavivattana, T.; Wilson, T. C.; Kersemans, V.; Smart, S.; Tredwell, M.; Davis, B. G.; Gouverneur, V. ^{18}F-Trifluoromethylation of Unmodified Peptides with 5-^{18}F-(Trifluoromethyl)dibenzothiophenium Trifluoromethanesulfonate. *J. Am. Chem. Soc.* **2018**, *140*, 1572-1575.

[4] Liu, M.; van Hensbergen, J.; Burford, R. P.; Lowe, A. B. Thiol-Michael coupling chemistry: facile access to a library of functional *exo*-7-oxanorbornenes and their ring-opening metathesis (co)polymerization. *Polym. Chem.* **2012**, *3*, 1647-1658.

[5] Yoneda, N.; Hotta, A.; Asano, K.; Matsubara, S. Asymmetric Oxy-Michael Addition to γ-Hydroxy-α,β-Unsaturated Carbonyls Using Formaldehyde as an Oxygen-Centered Nucleophile. *Org. Lett.* **2014**, *16*, 6264-6266.

[6] Levai, A.; Sebok, P. New Procedures for preparation of isoflavones with unsubstituted ring A. *Synthetic Communications*. **1992**, *22*, 1735-1750

[7] Chang, C.-W.; Wu, C.-H.; Lin, M.-H.; Liao, P.-H.; Chang, C.-C.; Chuang, H.-H.; Lin, S.-C.; Lam, S.; Verma, V. P.; Hsu, C.-P.; Wang, C.-C. Establishment of Guidelines for the Control of Glycosylation Reactions and Intermediates by Quantitative Assessment of Reactivity. *Angew. Chem., Int. Ed.* **2019**, *58*, 16775-16779

[8] Allen, C. L.; Miller, S. J. Chiral Copper (II) Complex-Catalyzed Reactions of Partially Protected Carbohydrates. *Org. lett.* **2013**, *15*, 6178-6181

[9] Yuan, X.; Cheng, S.; Shi, Y.; Xue, W. Photocatalytic synthesis of glycosyl bromides. *Synthesis*. **2014**, *46*, 331-335.

[10] Niclolas, L.; Izquierdo, E.; Angibaud, P.; Stansfield, I.; Meerpoel, L,; Reymond, S.; Cossy, J. Cobalt-Catalyzed Diastereoselective Synthesis of *C*-Furanosides. Total Synthesis of (-)-Isoaltholactone. *J. Org. Chem.* **2013**, *78*, 11807-11814.

[11] Le, C.; Chen, T. Q.; Liang, T.; Zhang, P.; MacMillan, D. W. C. A radical approach to the copper oxidative addition problem: Trifluoromethylation of bromoarenes. *Science* **2018**, *360*, 1010-1014.

[12] Engle, S. M.; Kirkner, T. R.; Kelly, C. B. Preparation of 2,4,5,6-Tetra(9*H*-carbazol-9-yl)isophthalonitrile. *Org. Synth.* **2019**, *96*, 455-473.

[13] Yu, F.; Li, J.; DeMent, P. M.; Tu Y.-J.; Schlegel, H. B.; Nguyen, H. M. Phenanthroline-catalyzed stereoretentive glycosylations. *Angew. Chem., Int. Ed.* **2019**, *58*, 6957-6961.

9. Compound Characterization Data

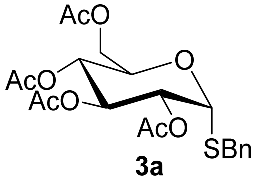

3a

(2*R*,3*R*,4*S*,5*R*,6*R*)-2-(acetoxymethyl)-6-(benzylthio)tetrahydro-2*H*-pyran-3,4,5-triyl triacetate (3a)

Following the **general procedrue A** using the **2a** as sulfur electrophile, the desired product **3a** was prepared as the colorless oil (32 mg, 72%) after purification by flash column chromatography on silica gel eluting with hexane/ethyl acetate (10:1 to 5:1).

^{1}H NMR (400 MHz, $CDCl_3$) δ 7.40 – 7.19 (m, 5H), 5.53 (d, *J* = 5.8 Hz, 1H), 5.36 (t, *J* = 9.8 Hz, 1H), 5.16 – 4.96 (m, 2H), 4.37 (ddd, *J* = 10.2, 4.7, 2.2 Hz, 1H), 4.24 (dd, *J* = 12.4, 4.6 Hz, 1H), 3.88 (dd, *J* = 12.4, 2.3 Hz, 1H), 3.72 (q, *J* = 13.5 Hz, 2H), 2.09 (s, 3H), 2.02 (s, 3H), 2.00 (d, *J* = 1.4 Hz, 6H).

^{13}C NMR (101 MHz, $CDCl_3$) δ 170.7, 170.1, 169.8, 169.7, 137.4, 129.0, 128.7, 127.5, 81.4, 70.8, 70.6, 68.7, 67.9, 61.9, 34.2, 20.9, 20.8 (2C), 20.8.

HRMS (ESI^+): calcd for $C_{21}H_{26}O_9S$ $[M+Na]^+$ 477.1190, found 477.1188.

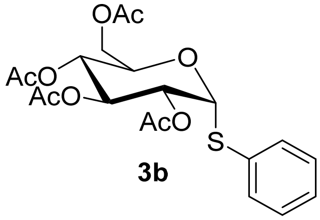

3b

(2*R*,3*R*,4*S*,5*R*,6*R*)-2-(acetoxymethyl)-6-(phenylthio)tetrahydro-2*H*-pyran-3,4,5-triyl triacetate (3b)

Following the **general procedure A** using **2h** as sulfur electrophile, the desired product **3b** was prepared as the white solid (30 mg, 70%) after purification by flash column chromatography on silica gel eluting with hexane/ethyl acetate (10:1 to 5:1).

^{1}H NMR (400 MHz, $CDCl_3$) δ 7.53 – 7.39 (m, 2H), 7.37 – 7.27 (m, 3H), 5.92 (d, *J* = 5.7 Hz, 1H), 5.44 (t, *J* = 9.8 Hz, 1H), 5.19 – 5.03 (m, 2H), 4.57 (ddd, *J* = 10.3, 5.1, 2.3 Hz, 1H), 4.28 (dd, *J* = 12.3, 5.1 Hz, 1H), 4.03 (dd, *J* = 12.4, 2.3 Hz, 1H), 2.10 (s, 3H), 2.05 (s, 3H), 2.04 (s, 3H), 2.02 (s, 3H).

^{13}C NMR (101 MHz, $CDCl_3$) δ 170.7, 170.1, 170.0, 169.8, 132.6, 132.0, 129.3, 127.9, 85.1, 70.9, 70.6, 68.7, 68.3, 62.1, 20.9, 20.8 (2C), 20.8.

HRMS (ESI^+): calcd for $C_{20}H_{24}O_9S$ $[M+Na]^+$ 463.1033, found 463.1032.

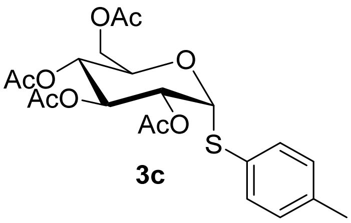

(2*R*,3*R*,4*S*,5*R*,6*R*)-2-(acetoxymethyl)-6-(p-tolylthio)tetrahydro-2*H*-pyran-3,4,5-triyl triacetate (3c)

Following the **general procedure A** using **2i** as sulfur electrophile, the desired product **3c** was prepared as the colorless oil (30 mg, 66%) after purification by flash column chromatography on silica gel eluting with hexane/ethyl acetate (10:1 to 5:1).

^{1}H NMR (400 MHz, $CDCl_3$) δ 7.32 (d, *J* = 8.2 Hz, 2H), 7.11 (d, *J* = 7.8 Hz, 2H), 5.83 (d, *J* = 5.8 Hz, 1H), 5.43 (t, *J* = 9.8 Hz, 1H), 5.18 – 4.99 (m, 2H), 4.58 (ddd, *J* = 10.4, 5.1, 2.3 Hz, 1H), 4.27 (dd, *J* = 12.3, 5.0 Hz, 1H), 4.03 (dd, *J* = 12.3, 2.3 Hz, 1H), 2.32 (s, 3H), 2.10 (s, 3H), 2.05 (s, 3H), 2.03 (s, 6H).

^{13}C NMR (101 MHz, $CDCl_3$) δ 170.7, 170.1, 170.0, 169.8, 138.3, 132.7, 130.1, 128.7, 85.6, 71.0, 70.6, 68.8, 68.2, 62.1, 21.3, 20.9, 20.8 (2C), 20.8.

HRMS (ESI$^+$): calcd for $C_{21}H_{26}O_9S$ $[M+Na]^+$ 477.1190, found 477.1186.

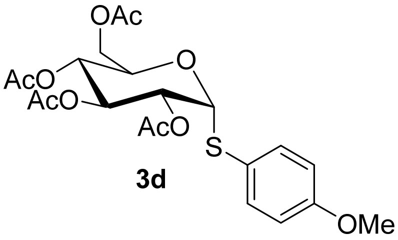

(2*R*,3*R*,4*S*,5*R*,6*R*)-2-(acetoxymethyl)-6-((4-methoxyphenyl)thio)tetrahydro-2*H*-pyran-3,4,5-triyl triacetate (3d)

Following the **general procedure A** using **2j** as sulfur electrophile, the desired product **3d** was prepared as the colorless oil (31 mg, 65%) after purification by flash column chromatography on silica gel eluting with hexane/ethyl acetate (10:1 to 5:1).

^{1}H NMR (400 MHz, $CDCl_3$) δ 7.37 (d, *J* = 8.8 Hz, 2H), 7.02 – 6.69 (m, 2H), 5.74 (d, *J* = 5.7 Hz, 1H), 5.43 (t, 1H), 5.20 – 4.96 (m, 2H), 4.68 – 4.56 (m, 1H), 4.28 (dd, *J* = 12.3, 5.1 Hz, 1H), 4.04 (dd, *J* = 12.3, 2.3 Hz, 1H), 3.79 (s, 3H), 2.12 (s, 3H), 2.06 (s, 3H), 2.05 (s, 3H), 2.04 (s, 3H).

^{13}C NMR (101 MHz, $CDCl_3$) δ 170.7, 170.1, 170.1, 169.8, 160.1, 135.2, 122.6, 115.0, 86.2, 71.1, 70.6, 68.8, 68.1, 62.2, 55.5, 21.0, 20.9, 20.8, 20.7.

HRMS (ESI$^+$): calcd for $C_{21}H_{26}O_{10}S$ $[M+Na]^+$ 493.1139, found 493.1138.

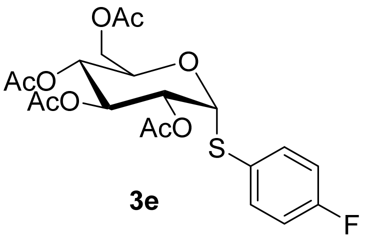

3e

(2*R*,3*R*,4*S*,5*R*,6*R*)-2-(acetoxymethyl)-6-((4-fluorophenyl)thio)tetrahydro-2*H*-pyran-3,4,5-triyl triacetate (3e)

Following the **general procedure A** using **2k** as the sulfur electrophile, the desired product **3e** was prepared as the white solid (32 mg, 68%) after purification by flash column chromatography on silica gel eluting with hexane/ethyl acetate (10:1 to 5:1).

^{1}H NMR (400 MHz, $CDCl_3$) δ 7.53 – 7.34 (m, 2H), 7.01 (t, *J* = 8.7 Hz, 2H), 5.82 (d, *J* = 5.7 Hz, 1H), 5.42 (t, *J* = 9.8 Hz, 1H), 5.22 – 4.98 (m, 2H), 4.55 (ddd, *J* = 10.4, 5.3, 2.3 Hz, 1H), 4.27 (dd, *J* = 12.4, 5.2 Hz, 1H), 4.04 (dd, *J* = 12.4, 2.3 Hz, 1H), 2.11 (s, 3H), 2.05 (s, 3H), 2.04 (s, 6H).

^{13}C NMR (101 MHz, $CDCl_3$) δ 170.6, 170.1, 170.0, 169.7, 162.9 (d, *J* = 248.8 Hz), 134.8 (d, *J* = 8.3 Hz), 127.5 (d, *J* = 3.4 Hz), 116.5 (d, *J* = 21.9 Hz), 85.8, 70.9, 70.5, 68.7, 68.3, 62.1, 20.9, 20.8 (2C), 20.8.

HRMS (ESI$^+$): calcd for $C_{20}H_{23}FO_9S$ $[M+Na]^+$ 481.0939, found 481.0938.

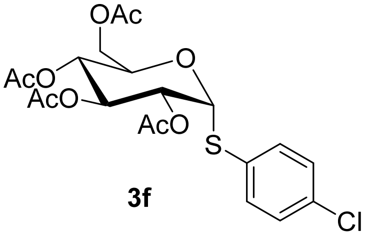

3f

(2*R*,3*R*,4*S*,5*R*,6*R*)-2-(acetoxymethyl)-6-((4-chlorophenyl)thio)tetrahydro-2*H*-pyran-3,4,5-triyl triacetate (3f)

Following the **general procedure A** using **2l** as the sulfur electrophile, the desired product **3f** was prepared as the white solid (30 mg, 64%) after purification by flash column chromatography on silica gel eluting with hexane/ethyl acetate (10:1 to 5:1).

^{1}H NMR (400 MHz, $CDCl_3$) δ 7.37 (d, *J* = 8.5 Hz, 2H), 7.27 (d, *J* = 11.0 Hz, 2H), 5.88 (d, *J* = 5.7 Hz, 1H), 5.42 (t, *J* = 9.8 Hz, 1H), 5.20 – 4.98 (m, 2H), 4.51 (ddd, *J* = 10.3, 5.3, 2.3 Hz, 1H), 4.25 (dd, *J* = 12.3, 5.3 Hz, 1H), 4.03 (dd, *J* = 12.4, 2.3 Hz, 1H), 2.10 (s, 3H), 2.05 (s, 3H), 2.03 (s, 3H), 2.02 (s, 3H).

^{13}C NMR (101 MHz, $CDCl_3$) δ 170.6, 170.1, 170.0, 169.7, 134.3, 133.3, 131.0, 129.5, 85.1, 70.9, 70.5, 68.7, 68.4, 62.1, 20.9, 20.8 (2C), 20.8.

HRMS (ESI$^+$): calcd for $C_{20}H_{23}ClO_9S$ $[M+Na]^+$ 497.0644, found 497.0645.

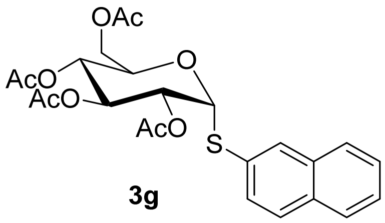

3g

(2R,3*R*,4*S*,5*R*,6*R*)-2-(acetoxymethyl)-6-(naphthalen-2-ylthio)tetrahydro-2*H*-pyran-3,4,5-triyl triacetate (3g)

Following the **general procedure A** using **2m** as the sulfur electrophile, the desired product **3g** was prepared as the white solid (27 mg, 55%) after purification by flash column chromatography on silica gel eluting with hexane/ethyl acetate (10:1 to 5:1).

^{1}H NMR (400 MHz, $CDCl_3$) δ 7.94 (d, *J* = 1.9 Hz, 1H), 7.84 – 7.68 (m, 3H), 7.57 – 7.40 (m, 3H), 6.06 (d, *J* = 5.7 Hz, 1H), 5.50 (t, *J* = 9.8 Hz, 1H), 5.15 (dd, *J* = 10.3, 5.7 Hz, 1H), 5.09 (t, *J* = 9.8 Hz, 1H), 4.61 (ddd, *J* = 10.3, 5.2, 2.3 Hz, 1H), 4.27 (dd, *J* = 12.3, 5.3 Hz, 1H), 4.02 (dd, *J* = 12.3, 2.3 Hz, 1H), 2.13 (s, 3H), 2.06 (s, 4H), 2.05 (s, 3H), 1.89 (s, 3H).

^{13}C NMR (101 MHz, $CDCl_3$) δ 170.70, 170.12, 170.05, 169.79, 133.81, 132.65, 130.93, 129.75, 128.94, 128.87, 127.88, 127.57, 126.88, 126.65, 84.85, 70.95, 70.65, 68.80, 68.44, 62.09, 20.93, 20.84, 20.78, 20.65.

HRMS (ESI$^+$): calcd for $C_{24}H_{26}O_9S$ $[M+Na]^+$ 513.1190, found 513.1185.

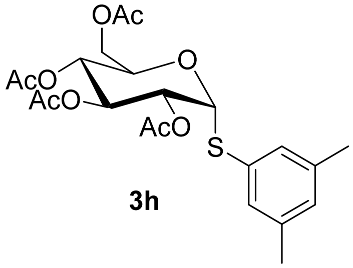

3h

(2*R*,3*R*,4*S*,5*R*,6*R*)-2-(acetoxymethyl)-6-((3,5-dimethylphenyl)thio)tetrahydro-2*H*-pyran-3,4,5-triyl triacetate (3h)

Following the **general procedure A** using **2n** as the sulfur electrophile, the desired product **3h** was prepared as the white solid (29 mg, 62%) after purification by flash column chromatography on silica gel eluting with hexane/ethyl acetate (10:1 to 5:1).

^{1}H NMR (400 MHz, $CDCl_3$) δ 7.04 (s, 2H), 6.89 (s, 1H), 5.90 (d, *J* = 5.7 Hz, 1H), 5.42 (t, *J* = 9.8 Hz, 1H), 5.28 – 5.01 (m, 2H), 4.56 (ddd, *J* = 10.4, 4.8, 2.2 Hz, 1H), 4.30 (dd, *J* = 12.3, 4.8 Hz, 1H), 4.02 (dd, *J* = 12.3, 2.3 Hz, 1H), 2.28 (s, 6H), 2.10 (s, 3H), 2.05 (s, 3H), 2.03 (s, 3H), 2.02 (s, 3H).

^{13}C NMR (101 MHz, $CDCl_3$) δ 170.7, 170.1, 170.0, 169.8, 138.9, 132.1, 129.8, 129.4, 85.1, 70.9, 70.6, 68.7, 68.2, 62.0, 21.3 (2C), 20.9, 20.8, 20.8, 20.8.

HRMS (ESI$^+$): calcd for $C_{22}H_{28}O_9S$ $[M+Na]^+$ 491.1346, found 491.1344.

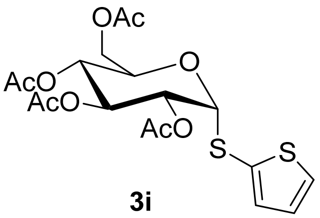

3i

(2*R*,3*R*,4*S*,5*R*,6*R*)-2-(acetoxymethyl)-6-(thiophen-2-ylthio)tetrahydro-2*H*-pyran-3,4,5-triyl triacetate (3i)

Following the **general procedure A** using **2o** as the sulfur electrophile, the desired product **3i** was prepared as the colorless oil (19 mg, 41%) after purification by flash column chromatography on silica gel eluting with hexane/ethyl acetate (10:1 to 5:1)..

^{1}H NMR (400 MHz, $CDCl_3$) δ 7.40 (dd, *J* = 5.4, 1.2 Hz, 1H), 7.19 – 7.12 (m, 1H), 7.00 (dd, *J* = 5.4, 3.6 Hz, 1H), 5.67 (d, *J* = 5.7 Hz, 1H), 5.43 (t, *J* = 9.8 Hz, 1H), 5.24 – 5.03 (m, 2H), 4.60 (ddd, *J* = 10.3, 4.7, 2.3 Hz, 1H), 4.33 (dd, *J* = 12.5, 4.6 Hz, 1H), 4.10 (dd, *J* = 12.4, 2.3 Hz, 1H), 2.13 (s, 3H), 2.08 (s, 3H), 2.06 (s, 3H), 2.03 (s, 3H).

^{13}C NMR (101 MHz, $CDCl_3$) δ 170.7, 170.1, 169.9, 169.7, 135.4, 130.9, 129.0, 127.8, 87.0, 70.8, 70.5, 68.6, 68.4, 61.9, 20.9, 20.9, 20.8, 20.8.

HRMS (ESI^+): calcd for $C_{18}H_{22}O_9S_2$ $[M+Na]^+$ 469.0597, found 469.0593.

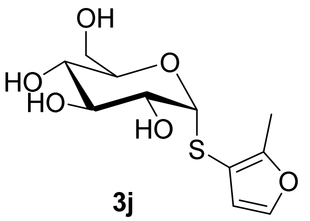

3j

(2*R*,3*S*,4*S*,5*R*,6*R*)-2-(hydroxymethyl)-6-((2-methylfuran-3-yl)thio)tetrahydro-2*H*-pyran-3,4,5-triol (3j)

Following the **general procedure A** using **2p** as the sulfur electrophile, the desired product **3j** was prepared using ethyl acetate/MeOH (5:1) as the eluent giving the white solid (18 mg, 65%) after purification by flash column chromatography on silica gel eluting with hexane/ethyl acetate (10:1 to 5:1).

^{1}H NMR (400 MHz, CD_3OD) δ 7.35 (d, *J* = 2.0 Hz, 1H), 6.48 (d, *J* = 2.0 Hz, 1H), 5.24 (d, *J* = 5.3 Hz, 1H), 4.12 (ddd, *J* = 10.0, 4.8, 2.6 Hz, 1H), 3.86 – 3.68 (m, 3H), 3.59 (t, *J* = 9.3 Hz, 1H), 3.37 (t, *J* = 9.4 Hz, 1H), 2.34 (s, 3H).

^{13}C NMR (101 MHz, CD_3OD) δ 156.5, 141.9, 116.4, 110.0, 91.5, 75.5, 74.5, 73.2, 71.6, 62.4, 11.9.

HRMS (ESI^+): calcd for $C_{11}H_{16}O_6S$ $[M+H]^+$ 299.0560, found 299.0556.

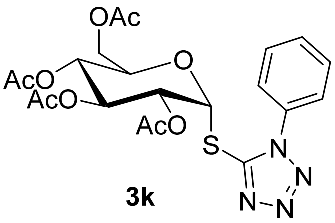

3k

(2R,3R,4S,5R,6R)-2-(acetoxymethyl)-6-((1-phenyl-1H-tetrazol-5-yl)thio)tetrahydro-2H-pyran-3,4,5-triyl triacetate (3k)

Following the **general procedure A** using difulfide **2q** as sulfur elctrophile, the desired product **3k** was prepared as the colorless oil (24 mg, 46%) after purification by flash column chromatography on silica gel eluting with DCM/ethyl acetate (20:1 to 5:1).

^{1}H NMR (400 MHz, $CDCl_3$) δ 7.80 – 7.50 (m, 5H), 6.53 (d, *J* = 5.3 Hz, 1H), 5.34 (t, *J* = 9.7 Hz, 1H), 5.25 (dd, *J* = 10.3, 5.4 Hz, 1H), 5.09 (t, *J* = 9.4 Hz, 1H), 4.37 – 4.14 (m, 2H), 3.95 (d, *J* = 10.9 Hz, 1H), 2.02 (d, *J* = 3.6 Hz, 12H).

^{13}C NMR (101 MHz, $CDCl_3$) δ 170.7, 170.1, 169.4, 169.3, 150.4, 133.5, 130.8, 130.1, 124.5, 85.0, 70.6, 70.3, 69.7, 67.9, 61.5, 20.8, 20.8, 20.7, 20.7.

HRMS (ESI$^+$): calcd for $C_{21}H_{24}N_4O_9S$ [M+Na]$^+$ 531.1156, found 531.1154.

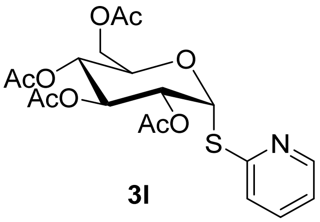

3l

(2*R*,3*R*,4*S*,5*R*,6*R*)-2-(acetoxymethyl)-6-(pyridin-2-ylthio)tetrahydro-2*H*-pyran-3,4,5-triyl triacetate (3l)

Following the **general procedure B** using the sulfur electrophile **2q** as sulfur electrophile, the desired product **3l** was prepared as the colorless oil (35 mg, 79%) after purification by flash column chromatography on silica gel eluting with DCM/ethyl acetate (20:1 to 5:1).

^{1}H NMR (400 MHz, $CDCl_3$) δ 8.45 (d, *J* = 4.9 Hz, 1H), 7.53 (t, *J* = 7.6 Hz, 1H), 7.28 (d, *J* = 8.0 Hz, 1H), 7.06 (dd, *J* = 7.4, 4.8 Hz, 1H), 6.65 (d, *J* = 5.6 Hz, 1H), 5.38 (t, *J* = 9.8 Hz, 1H), 5.24 (dd, *J* = 10.3, 5.7 Hz, 1H), 5.11 (t, *J* = 9.7 Hz, 1H), 4.45 – 4.31 (m, 1H), 4.25 (dd, *J* = 12.4, 4.5 Hz, 1H), 4.09 – 3.83 (m, 1H), 2.02 (s, 6H), 2.00 (s, 3H), 1.97 (s, 3H).

^{13}C NMR (101 MHz, $CDCl_3$) δ 170.68, 170.09, 169.82, 169.68, 155.50, 149.97, 136.82, 123.87, 121.09, 81.63, 71.06, 69.99, 69.52, 68.46, 61.85, 20.80, 20.77, 20.75, 20.72.

HRMS (ESI$^+$): calcd for $C_{19}H_{23}NO_9S$ [M+Na]$^+$ 464.0986, found 464.0981.

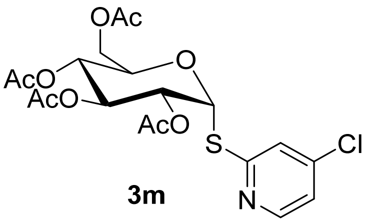

3m

(2*R*,3*R*,4*S*,5*R*,6*R*)-2-(acetoxymethyl)-6-((4-chloropyridin-2-yl)thio)tetrahydro-2*H*-pyran-3,4,5-triyl triacetate (3m)

Following the **general procedure A** using the disulfide **2z** as the sulfur electrophile, the desired product **3m** was obtained as the colorless oil (28.5 mg, 60%) after purification by flash column chromatography on silica gel eluting with DCM/ethyl acetate (20:1 to 5:1).

^{1}H NMR (400 MHz, $CDCl_3$) δ 8.43 (d, *J* = 2.5 Hz, 1H), 7.53 (dd, *J* = 8.5, 2.6 Hz, 1H), 7.25 (d, *J* = 8.1 Hz, 1H), 6.61 (d, *J* = 5.7 Hz, 1H), 5.38 (t, *J* = 9.8 Hz, 1H), 5.24 (dd, *J* = 10.4, 5.7 Hz, 1H), 5.12 (t, *J* = 9.7 Hz, 1H), 4.34 (ddd, *J* = 10.2, 4.6, 2.3 Hz, 1H), 4.26 (dd, *J* = 12.4, 4.5 Hz, 1H), 4.00 (dd, *J* = 12.4, 2.3 Hz, 1H), 2.03 (d, *J* = 2.2 Hz, 9H), 2.00 (s, 3H).

^{13}C NMR (101 MHz, $CDCl_3$) δ 170.66, 170.10, 169.80, 169.67, 153.61, 148.77, 136.66, 129.73, 124.57, 82.02, 71.00, 70.02, 69.69, 68.43, 61.85, 20.81, 20.78 (2C), 20.73.

HRMS (ESI$^+$): calcd for $C_{19}H_{22}ClNO_9S$ $[M+Na]^+$ 498.0596, found 498.0592.

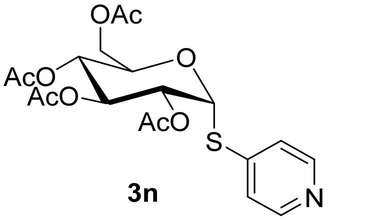

3n

(2*R*,3*R*,4*S*,5*R*,6*R*)-2-(acetoxymethyl)-6-(pyridin-4-ylthio)tetrahydro-2H-pyran-3,4,5-triyl triacetate (3n)

Following the **general procedure A** using disulfide **2ah** as the sulfur electrophile, the desired product **3n** was prepared as the colorless oil (31 mg, 70%) after purification by flash column chromatography on silica gel eluting with DCM/ethyl acetate (20:1 to 5:1).

^{1}H NMR (400 MHz, $CDCl_3$) δ 8.77 – 8.19 (m, 2H), 7.30 (d, *J* = 5.4 Hz, 2H), 6.18 (d, *J* = 5.7 Hz, 1H), 5.43 (t, *J* = 9.8 Hz, 1H), 5.16 (dd, *J* = 10.3, 5.8 Hz, 1H), 5.09 (t, *J* = 9.8 Hz, 1H), 4.40 (ddd, *J* = 10.4, 5.3, 2.1 Hz, 1H), 4.25 (dd, *J* = 12.4, 5.3 Hz, 1H), 4.03 (dd, *J* = 12.4, 2.2 Hz, 1H), 2.08 (s, 3H), 2.04 (s, 6H), 1.98 (s, 3H).

^{13}C NMR (101 MHz, $CDCl_3$) δ 170.57, 170.03, 169.86, 169.68, 149.82 (2C), 144.95, 122.92, 82.22, 70.38, 70.36, 69.00, 68.39, 61.79, 20.79, 20.77, 20.73, 20.71.

HRMS (ESI$^+$): calcd for $C_{19}H_{23}NO_9S$ $[M+H]^+$ 442.1166, found 442.1167.

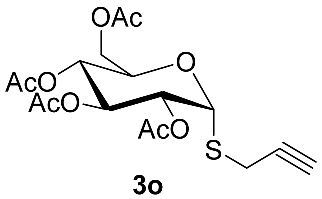

3o

(2*R*,3*R*,4*S*,5*R*,6*R*)-2-(acetoxymethyl)-6-(prop-2-yn-1-ylthio)tetrahydro-2*H*-pyran-3,4,5-triyl triacetate (3o)

Following the **general procedure B** using the sulfur electrophile **2s** as the sulfur electrophile, the desired product **3o** was prepared as the colorless oil (12 mg, 30%) after purification by flash column chromatography on silica gel eluting with hexane/ethyl acetate (20:1 to 10:1).

^{1}H NMR (400 MHz, $CDCl_3$) δ 5.86 (d, *J* = 5.8 Hz, 1H), 5.35 (t, *J* = 9.8 Hz, 1H), 5.24 – 4.95 (m, 2H), 4.62 – 4.36 (m, 1H), 4.31 (dd, *J* = 12.3, 4.7 Hz, 1H), 4.08 (dd, *J* = 12.3, 2.2 Hz, 1H), 3.34 (dd, *J* = 16.8, 2.6 Hz, 1H), 3.18 (dd, *J* = 16.7, 2.7 Hz, 1H), 2.23 (s, 1H), 2.09 (s, 3H), 2.06 (s, 3H), 2.03 (s, 3H), 2.01 (s, 3H).

^{13}C NMR (101 MHz, $CDCl_3$) δ 170.7, 170.1, 169.8, 169.7, 81.4, 78.9, 71.8, 70.7, 70.5, 68.6, 68.2, 61.9, 20.9, 20.8, 20.8, 20.7, 17.3.

HRMS (ESI$^+$): calcd for $C_{17}H_{22}O_9S$ $[M+Na]^+$ 425.0877, found 425.0872.

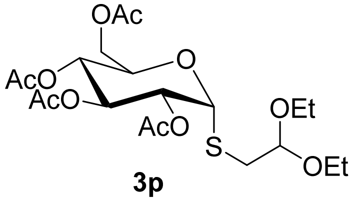

3p

(2*R*,3*R*,4*S*,5*R*,6*R*)-2-(acetoxymethyl)-6-((2,2-diethoxyethyl)thio)tetrahydro-2*H*-pyran-3,4,5-triyl triacetate (3p)

Following the **general procedure B** using sulfur electrophile **2t** as the sulfur electrophile, the desired product **3p** was prepared as the white solid (34 mg, 70%) after purification by flash column chromatography on silica gel eluting with hexane/ethyl acetate (20:1 to 5:1).

^{1}H NMR (400 MHz, $CDCl_3$) δ 5.76 (d, *J* = 5.8 Hz, 1H), 5.38 (t, *J* = 9.8 Hz, 1H), 5.22 – 4.94 (m, 2H), 4.59 (dd, *J* = 6.4, 4.8 Hz, 1H), 4.44 (ddd, *J* = 10.3, 4.7, 2.2 Hz, 1H), 4.29 (dd, *J* = 12.4, 4.6 Hz, 1H), 4.06 (dd, *J* = 12.4, 2.3 Hz, 1H), 3.85 – 3.59 (m, 2H), 3.58 – 3.38 (m, 2H), 2.80 (dd, *J* = 13.7, 6.4 Hz, 1H), 2.62 (dd, *J* = 13.7, 4.8 Hz, 1H), 2.08 (s, 3H), 2.06 (s, 3H), 2.02 (s, 3H), 2.01 (s, 3H), 1.20 (t, *J* = 7.1 Hz, 6H).

^{13}C NMR (101 MHz, $CDCl_3$) δ 170.76, 170.08, 169.93, 169.77, 102.27, 81.98, 70.78, 70.56, 68.72, 67.75, 62.38, 62.07, 61.98, 32.43, 20.84 (2C), 20.80, 20.75, 15.42, 15.32.

HRMS (ESI$^+$): calcd for $C_{20}H_{32}O_{11}S$ $[M+Na]^+$ 503.1558, found 503.1553.

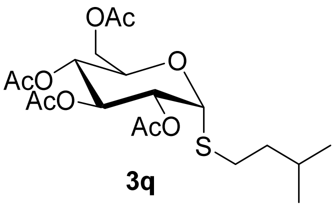

3q

(2*R*,3*R*,4*S*,5*R*,6*R*)-2-(acetoxymethyl)-6-(isopentylthio)tetrahydro-2*H*-pyran-3,4,5-triyl triacetate (3q)

Following the **general procedure B** using **2u** as the sulfur electrophile, the desired product **3q** was prepared as the colorless oil (16 mg, 37%) after purification by flash column chromatography on silica gel eluting with hexane/ethyl acetate (50:1 to 10:1).

^{1}H NMR (400 MHz, $CDCl_3$) δ 5.66 (d, *J* = 5.8 Hz, 1H), 5.37 (t, *J* = 9.8 Hz, 1H), 5.16 – 4.82 (m, 2H), 4.43 (ddd, *J* = 10.3, 4.7, 2.1 Hz, 1H), 4.30 (dd, *J* = 12.3, 4.8 Hz, 1H), 4.07 (dd, *J* = 12.3, 2.3 Hz, 1H), 2.77 – 2.48 (m, 2H), 2.09 (s, 3H), 2.06 (s, 3H), 2.03 (s, 3H), 2.01 (s, 3H), 1.74 – 1.61 (m, 1H), 1.54 – 1.39 (m, 2H), 0.90 (dd, *J* = 6.6, 2.0 Hz, 6H).

^{13}C NMR (101 MHz, $CDCl_3$) δ 170.76, 170.10, 170.07, 169.79, 82.08, 70.91, 70.70, 68.77, 67.66, 62.18, 38.36, 28.31, 27.62, 22.44, 22.27, 20.91, 20.88, 20.84, 20.78.

HRMS (ESI^+): calcd for $C_{19}H_{30}O_9S$ $[M+Na]^+$ 457.1503, found 457.1499.

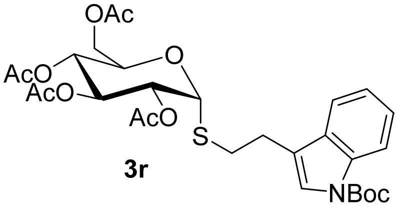

3r

(2*R*,3*R*,4*S*,5*R*,6*R*)-2-(acetoxymethyl)-6-((2-(1-(tert-butoxycarbonyl)-1*H*-indol-3-yl)ethyl)thio)tetrahydro-2H-pyran-3,4,5-triyl triacetate (3r)

Following the **general procedure B** using **2v** as the sulfur electrophile, the desired product **3r** was prepared as the colorless oil (29 mg, 49%) after purification by flash column chromatography on silica gel eluting with hexane/ethyl acetate (10:1 to 5:1).

^{1}H NMR (400 MHz, $CDCl_3$) δ 8.12 (d, *J* = 7.9 Hz, 1H), 7.50 (d, *J* = 7.7 Hz, 1H), 7.40 (s, 1H), 7.32 (t, *J* = 8.0 Hz, 1H), 7.23 (d, *J* = 7.4 Hz, 1H), 5.76 (d, *J* = 5.8 Hz, 1H), 5.37 (t, *J* = 9.8 Hz, 1H), 5.26 – 4.92 (m, 2H), 4.42 (ddd, *J* = 10.4, 4.9, 2.1 Hz, 1H), 4.27 (dd, *J* = 12.4, 5.0 Hz, 1H), 4.04 (dd, *J* = 12.4, 2.2 Hz, 1H), 3.26 – 2.71 (m, 4H), 2.05 (s, 3H), 2.03 (s, 3H), 2.02 (s, 3H), 1.99 (s, 3H), 1.67 (s, 9H).

^{13}C NMR (101 MHz, $CDCl_3$) δ 170.70, 170.06, 170.04, 169.76, 149.82, 135.65, 130.20, 124.65, 123.15, 122.66, 118.91, 118.83, 115.53, 83.76, 82.15, 70.89, 70.64, 68.76, 67.85, 62.13, 29.93, 28.38 (3C), 25.60, 20.88, 20.82, 20.79, 20.76.

HRMS (ESI^+): calcd for $C_{29}H_{37}NO_{11}S$ $[M+Na]^+$ 630.1980, found 630.1979.

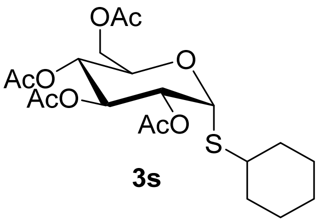

3s

(2*R*,3*R*,4*S*,5*R*,6*R*)-2-(acetoxymethyl)-6-(cyclohexylthio)tetrahydro-2*H*-pyran-3,4,5-triyl triacetate (3s)

Following the **general procedure B** using **2w** as the sulfur electrophile, the desired product **3s** was prepared as the colorless oil (35 mg, 79%) after purification by flash column chromatography on silica gel eluting with hexane/ethyl acetate (20:1 to 5:1).

^{1}H NMR (400 MHz, $CDCl_3$) δ 5.76 (d, *J* = 5.8 Hz, 1H), 5.33 (t, *J* = 9.8 Hz, 1H), 5.10 – 4.90 (m, 2H), 4.66 – 4.43 (m, 1H), 4.28 (dd, *J* = 12.3, 4.8 Hz, 1H), 4.07 (dd, *J* = 12.3, 2.3 Hz, 1H), 2.76 (t, *J* = 10.6 Hz, 1H), 2.08 (s, 3H), 2.06 (s, 3H), 2.03 (s, 3H), 2.01 (s, 3H), 1.98 – 1.89 (m, 2H), 1.80 – 1.67 (m, 2H), 1.50 – 1.16 (m, 6H).

^{13}C NMR (101 MHz, $CDCl_3$) δ 170.77, 170.11, 170.06, 169.81, 81.29, 70.93, 70.72, 68.83, 67.69, 62.21, 43.66, 34.18, 33.71, 26.23, 25.99, 25.76, 20.95, 20.87, 20.83, 20.78.

HRMS (ESI$^+$): calcd for $C_{20}H_{30}O_9S$ $[M+Na]^+$ 469.1503, found 469.1498.

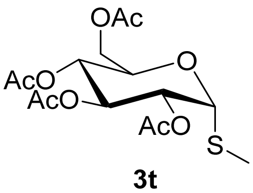

3t

(2*R*,3*R*,4*S*,5*R*,6*R*)-2-(acetoxymethyl)-6-(methylthio)tetrahydro-2*H*-pyran-3,4,5-triyl triacetate (**3t**)

Following the **general procedure B** using **2aj** as the sulfur electrophile, the desired product **3t** was prepared as the colorless oil (13 mg, 35%) after purification by flash column chromatography on silica gel eluting with hexane/ethyl acetate (20:1 to 5:1).

^{1}H NMR (400 MHz, $CDCl_3$) δ 5.55 (d, *J* = 5.8 Hz, 1H), 5.39 (t, *J* = 9.7 Hz, 1H), 5.14 – 4.91 (m, 2H), 4.39 (ddd, *J* = 10.2, 4.9, 2.3 Hz, 1H), 4.30 (dd, *J* = 12.3, 4.8 Hz, 1H), 4.09 (dd, *J* = 12.3, 2.3 Hz, 1H), 2.09 (s, 3H), 2.07 (s, 3H), 2.06 (s, 3H), 2.04 (s, 3H), 2.02 (s, 3H).

^{13}C NMR (101 MHz, $CDCl_3$) δ 170.74, 170.11, 170.03, 169.78, 82.98, 70.92, 70.67, 68.75, 67.54, 62.12, 20.86 (2C), 20.82, 20.75, 12.55.

LRMS (ESI$^+$): calcd for $C_{15}H_{22}O_9S$ $[M+NH_4]^+$ 396, found 396.

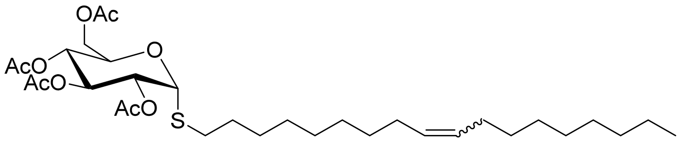

3u

(2*R*,3*R*,4*S*,5*R*,6*R*)-2-(acetoxymethyl)-6-(octadec-9-en-1-ylthio)tetrahydro-2*H*-pyran-3,4,5-triyl triacetate (**3u**)

Following the **general procedure B** using **2ak** as the sulfur electrophile, the desired product **3u** was prepared as the colorless oil (27 mg, 43%) after purification by flash column chromatography on silica gel eluting with hexane/ethyl acetate (20:1 to 5:1).

^{1}H NMR (400 MHz, $CDCl_3$) δ 5.65 (d, *J* = 5.8 Hz, 1H), 5.45 – 5.28 (m, 3H), 5.13 – 4.94 (m, 2H), 4.43 (ddd, *J* = 10.2, 4.7, 2.3 Hz, 1H), 4.30 (dd, *J* = 12.3, 4.7 Hz, 1H), 4.07 (dd, *J* = 12.3, 2.3 Hz, 1H), 2.52 (ddt, *J* = 20.1, 14.8, 6.4 Hz, 2H), 2.09 (s, 3H), 2.06 (s, 3H), 2.04 (s, 3H), 2.02 (s, 3H), 1.96 (q, *J* = 6.6 Hz, 3.3H, *trans*), 1.68 – 1.49 (m, 2.7H, *cis*), 1.38 – 1.16 (m, 22H), 0.88 (t, *J* = 6.7 Hz, 3H).

^{13}C NMR (101 MHz, $CDCl_3$) δ 170.74, 170.09, 170.05, 169.78, 130.61 (*cis*), 130.37 (*cis*), 130.14 (*trans*), 129.90 (*trans*), 82.15, 70.91, 70.70, 68.74, 67.64, 62.11, 32.75, 32.72, 32.04, 30.30, 29.80, 29.75, 29.63, 29.54, 29.52, 29.45, 29.32, 29.26, 29.22, 29.03, 27.36, 27.33, 22.82, 20.89, 20.85, 20.81, 20.76, 14.25.

HRMS (ESI$^+$): calcd for $C_{32}H_{54}O_9S$ $[M+H]^+$ 614.3489, found 614.3485.

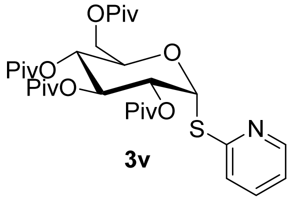

3v

(2*R*,3*R*,4*S*,5*R*,6*R*)-2-((pivaloyloxy)methyl)-6-(pyridin-2-ylthio)tetrahydro-2*H*-pyran-3,4,5-triyl tris(2,2-dimethylpropanoate) (3v)

Following the **general procedure B** using **2q** as the sulfur electrophile, the desired product **3v** was prepared as the white solid (45 mg, 74%) after purification by flash column chromatography on silica gel eluting with DCM/ethyl acetate (10:1 to 5:1).

^{1}H NMR (400 MHz, $CDCl_3$) δ 8.67 – 8.29 (m, 1H), 7.52 (td, *J* = 7.7, 1.9 Hz, 1H), 7.27 (d, *J* = 9.8 Hz, 1H), 7.04 (dd, *J* = 7.5, 4.9 Hz, 1H), 6.73 (d, *J* = 5.9 Hz, 1H), 5.46 (t, *J* = 9.8 Hz, 1H), 5.22 (dd, *J* = 10.3, 5.8 Hz, 1H), 5.16 (t, *J* = 9.8 Hz, 1H), 4.64 – 4.31 (m, 1H), 4.28 – 3.90 (m, 2H), 1.17 (s, 9H), 1.14 (s, 9H), 1.12 (s, 9H), 1.07 (s, 9H).

^{13}C NMR (101 MHz, $CDCl_3$) δ 178.12, 177.31, 177.01, 176.70, 155.79, 149.96, 136.78, 123.53, 120.87, 81.50, 70.58, 70.25, 70.07, 67.92, 62.01, 38.94, 38.89, 38.87, 38.82, 27.34, 27.22, 27.14, 27.11.

HRMS (ESI$^+$): calcd for $C_{31}H_{47}NO_9S$ $[M+Na]^+$ 632.2864, found 632.2858.

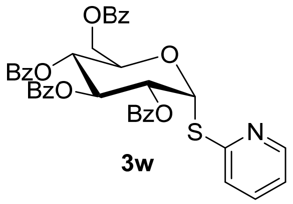

3w

(2*R*,3*R*,4*S*,5*R*,6*R*)-2-((benzoyloxy)methyl)-6-(pyridin-2-ylthio)tetrahydro-2*H*-pyran-3,4,5-triyl tribenzoate (3w)

Following the **general procedure B** using **2q** as the sulfur electrophile, the desired product **3w** was prepared as the colorless oil (50 mg, 73%) after purification by flash column chromatography on silica gel eluting with DCM/ethyl acetate (10:1 to 5:1).

^{1}H NMR (400 MHz, $CDCl_3$) δ 8.40 (d, *J* = 4.8 Hz, 1H), 7.96 (t, *J* = 6.8 Hz, 4H), 7.88 (t, *J* = 8.9 Hz, 4H), 7.50 (q, *J* = 7.8 Hz, 3H), 7.36 (ddq, *J* = 29.1, 15.5, 7.8 Hz, 11H), 7.11 – 6.87 (m, 2H), 6.12 (t, *J* = 9.9 Hz, 1H), 5.87 – 5.56 (m, 2H), 5.00 – 4.78 (m, 1H), 4.64 – 4.40 (m, 2H).

^{13}C NMR (101 MHz, $CDCl_3$) δ 166.13, 165.77, 165.38 (2C), 155.49, 149.93, 136.74, 133.63, 133.61, 133.39, 133.11, 130.13, 130.04, 129.87, 129.82, 129.71, 129.07, 128.86, 128.85, 128.57, 128.54, 128.46, 128.39, 123.77, 120.94, 81.90, 71.42, 70.99, 69.92, 69.53, 63.11.

HRMS (ESI^+): calcd for $C_{39}H_{31}NO_9S$ $[M+Na]^+$ 712.1612, found 712.1614.

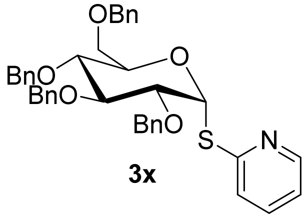

3x

2-(((2*R*,3*R*,4*S*,5*R*,6*R*)-3,4,5-tris(benzyloxy)-6-((benzyloxy)methyl)tetrahydro-2*H*-pyran-2-yl)thio)pyridine (3x)

Following the **general procedure B** using **2q** as the sulfur electrophile, and the glucosyl bromide (0.3 mmol 3.0 equiv) was used, the desired product **3x** was prepared as the colorless oil (35 mg, 56%) after purification by flash column chromatography on silica gel eluting with hexane/ethyl acetate (20:1 to 5:1).

^{1}H NMR (400 MHz, $CDCl_3$) δ 8.48 (d, *J* = 4.9 Hz, 1H), 7.52 (t, *J* = 7.8 Hz, 1H), 7.38 (d, *J* = 7.7 Hz, 1H), 7.35 – 7.11 (m, 20H), 7.07 (t, *J* = 6.1 Hz, 1H), 6.69 (d, *J* = 5.3 Hz, 1H), 4.99 (d, *J* = 10.8 Hz, 1H), 4.81 (q, *J* = 10.3 Hz, 3H), 4.66 (d, *J* = 11.6 Hz, 1H), 4.49 (d, *J* = 11.2 Hz, 2H), 4.35 (d, *J* = 12.0 Hz, 1H), 4.10 (dd, *J* = 10.3, 3.7 Hz, 1H), 3.99 (dd, *J* = 9.6, 4.9 Hz, 1H), 3.85 (t, *J* = 9.2 Hz, 1H), 3.68 (dq, *J* = 13.5, 9.4, 6.7 Hz, 2H), 3.57 (d, *J* = 10.8 Hz, 1H).

^{13}C NMR (101 MHz, $CDCl_3$) δ 156.39, 148.36, 138.75, 138.30, 138.07, 137.97, 137.91, 137.68, 128.51, 128.43, 128.33, 128.15, 128.02, 127.99, 127.96, 127.92, 127.85, 127.76, 125.16, 121.00, 84.19, 82.94, 79.22, 77.31, 75.87, 75.27, 73.45, 72.86, 72.53, 68.74.

HRMS (ESI^+): calcd for $C_{39}H_{39}NO_5S$ $[M+H]^+$ 634.2622, found 634.2613.

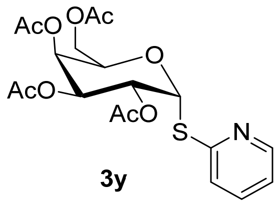

3y

(2*R*,3*S*,4*S*,5*R*,6*R*)-2-(acetoxymethyl)-6-(pyridin-2-ylthio)tetrahydro-2*H*-pyran-3,4,5-triyl triacetate (3y)

Following the **general procedure B** using **2w** as the sulfur electrophile, the desired product **3y** was prepared as the colorless oil (36 mg, 81%) after purification by flash column chromatography on silica gel eluting with DCM/ethyl acetate (10:1 to 5:1).

^{1}H NMR (400 MHz, $CDCl_3$) δ 8.66 – 8.36 (m, 1H), 7.54 (td, *J* = 7.7, 2.0 Hz, 1H), 7.30 (d, 1H), 7.07 (ddd, *J* = 7.4, 4.9, 1.1 Hz, 1H), 6.70 (d, *J* = 5.7 Hz, 1H), 5.60 – 5.44 (m, 2H), 5.25 (dd, *J* = 11.0, 3.3 Hz, 1H), 4.65 – 4.48 (m, 1H), 4.24 – 3.98 (m, 2H), 2.15 (s, 3H), 2.02 (s, 3H), 2.00 (s, 3H), 1.89 (s, 3H).

^{13}C NMR (101 MHz, $CDCl_3$) δ 170.4, 170.3, 170.1, 170.0, 155.9, 149.9, 136.7, 124.0, 121.0, 82.3, 68.7, 68.4, 67.8, 67.3, 61.5, 20.9, 20.8, 20.7, 20.7.

HRMS (ESI$^+$): calcd for $C_{19}H_{23}NO_9S$ $[M+Na]^+$ 464.0986, found 464.0981.

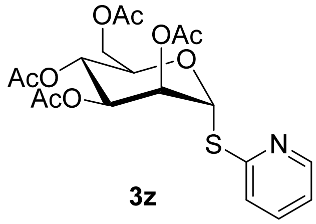

3z

(2*R*,3*R*,4*S*,5*S*,6*R*)-2-(acetoxymethyl)-6-(pyridin-2-ylthio)tetrahydro-2*H*-pyran-3,4,5-triyl triacetate (3z)

Following the **general procedure B** using **2q** as the sulfur electrophile, the desired product **3z** was prepared as the colorless oil (38 mg, 85%) after purification by flash column chromatography on silica gel eluting with DCM/ethyl acetate (10:1 to 5:1).

^{1}H NMR (400 MHz, $CDCl_3$) δ 8.68 – 8.35 (m, 1H), 7.55 (td, *J* = 7.7, 1.9 Hz, 1H), 7.26 (d, *J* = 7.9 Hz, 1H), 7.08 (dd, *J* = 6.6, 5.0 Hz, 1H), 6.45 (d, *J* = 1.8 Hz, 1H), 5.53 (dd, *J* = 3.3, 1.8 Hz, 1H), 5.38 (dd, *J* = 11.2, 8.5 Hz, 1H), 5.33 – 5.21 (m, 2H), 4.46 – 4.25 (m, 2H), 4.05 (d, *J* = 9.5 Hz, 1H), 2.19 (s, 3H), 2.05 (s, 3H), 2.01 (s, 3H), 2.00 (s, 3H).

^{13}C NMR (101 MHz, $CDCl_3$) δ 170.72, 170.02, 169.99, 169.80, 154.66, 150.04, 136.90, 123.76, 121.20, 81.60, 71.33, 70.93, 69.97, 66.30, 62.41, 21.07, 20.84, 20.80, 20.78.

HRMS (ESI$^+$): calcd for $C_{19}H_{23}NO_9S$ $[M+Na]^+$ 464.0986, found 464.0984.

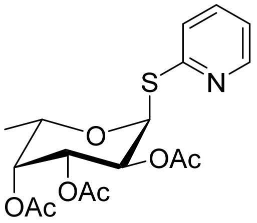

3aa

(2*S*,3*R*,4*R*,5*S*,6*S*)-2-methyl-6-(pyridin-2-ylthio)tetrahydro-2*H*-pyran-3,4,5-triyl triacetate (3aa)

Following the **general procedure B** uisng **2q** as the sulfur electrophile, the desired product **3aa** was prepared as the colorless oil (26 mg, 67%) after purification by flash column chromatography on silica gel eluting with DCM/ethyl acetate (10:1 to 5:1).

^{1}H NMR (400 MHz, $CDCl_3$) δ 8.45 (d, *J* = 3.5 Hz, 1H), 7.53 (td, *J* = 7.7, 1.9 Hz, 1H), 7.29 (d, *J* = 8.1 Hz, 1H), 7.05 (dd, *J* = 7.4, 4.8 Hz, 1H), 6.67 (d, *J* = 5.6 Hz, 1H), 5.48 (dd, *J* = 11.0, 5.6 Hz, 1H), 5.33 (d, *J* = 3.4 Hz, 1H), 5.25 (dd, *J* = 11.0, 3.4 Hz, 1H), 4.44 (q, *J* = 6.4 Hz, 1H), 2.17 (s, 3H), 2.08 – 1.85 (m, 6H), 1.12 (d, *J* = 6.4 Hz, 3H).

^{13}C NMR (101 MHz, $CDCl_3$) δ 170.69, 170.20, 170.09, 156.44, 149.89, 136.73, 123.70, 120.81, 82.34, 70.93, 69.21, 67.39, 67.16, 20.95, 20.82, 20.77, 16.18.

HRMS (ESI^+): calcd for $C_{17}H_{21}NO_7S$ $[M+Na]^+$ 406.0931, found 406.0928.

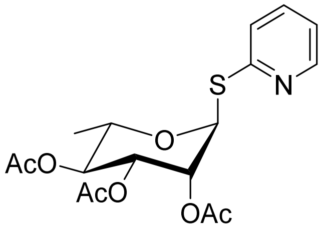

3ab

(2*S*,3*S*,4*R*,5*R*,6*S*)-2-methyl-6-(pyridin-2-ylthio)tetrahydro-2*H*-pyran-3,4,5-triyl triacetate (3ab)

Following the **general procedure B** uisng **2q** as the sulfur electrophile, the desired product **3ab** was prepared as the colorless oil (29 mg, 75%) after purification by flash column chromatography on silica gel eluting with DCM/ethyl acetate (10:1 to 5:1).

^{1}H NMR (400 MHz, $CDCl_3$) δ 8.47 (d, *J* = 4.1 Hz, 1H), 7.54 (td, *J* = 7.7, 1.9 Hz, 3H), 7.24 (d, *J* = 8.1 Hz, 1H), 7.06 (ddd, *J* = 7.4, 4.8, 1.1 Hz, 3H), 6.41 (d, *J* = 1.8 Hz, 3H), 5.53 (dd, *J* = 3.3, 1.8 Hz, 3H), 5.25 (dd, *J* = 10.0, 3.3 Hz, 3H), 5.16 (t, *J* = 9.8 Hz, 3H), 4.14 (dq, *J* = 9.3, 6.2 Hz, 3H), 2.18 (s, 10H), 2.07 (s, 10H), 2.00 (s, 10H), 1.23 (d, *J* = 6.2 Hz, 10H).

^{13}C NMR (101 MHz, $CDCl_3$) δ 170.14, 170.11 (2C), 155.21, 150.01, 136.84, 123.54, 120.97, 81.29, 71.83, 71.21, 69.98, 69.46, 21.12, 20.95, 20.83, 17.63.

HRMS (ESI^+): calcd for $C_{17}H_{21}NO_7S$ $[M+Na]^+$ 406.0931, found 406.0927.

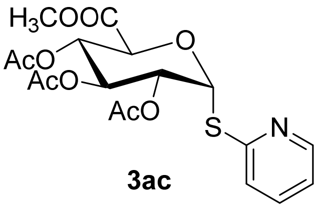

3ac

(2*S*,3*S*,4*S*,5*R*,6*R*)-2-(methoxycarbonyl)-6-(pyridin-2-ylthio)tetrahydro-2*H*-pyran-3,4,5-triyl triacetate (3ac)

Following the **general procedure B** using **2q** as the sulfur electrophile, the desired product **3ac** was prepared as the colorless oil (32 mg, 74%) after purification by flash column chromatography on silica gel eluting with DCM/ethyl acetate (10:1 to 5:1).

^{1}H NMR (400 MHz, $CDCl_3$) δ 8.45 (d, *J* = 3.3 Hz, 1H), 7.55 (td, *J* = 7.8, 1.9 Hz, 1H), 7.32 (d, *J* = 8.0 Hz, 1H), 7.16 – 7.00 (m, 1H), 6.76 (d, *J* = 5.3 Hz, 1H), 5.50 – 5.32 (m, 1H), 5.30 – 5.11 (m, 2H), 4.68 (d, *J* = 9.4 Hz, 1H), 3.70 (s, 3H), 2.04 (s, 6H), 2.03 (s, 3H).

^{13}C NMR (101 MHz, $CDCl_3$) δ 169.75, 169.70, 169.63, 167.84, 155.08, 149.98, 136.95, 123.80, 121.22, 81.31, 70.73, 69.91, 69.55, 69.36, 52.96, 20.78 (2C), 20.68.

HRMS (ESI$^+$): calcd for $C_{18}H_{21}NO_9S$ $[M+Na]^+$ 450.0829, found 450.0822.

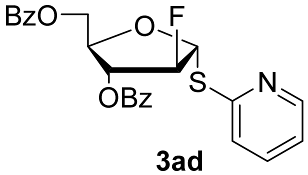

3ad

((2*R*,3*R*,4*S*,5*R*)-3-(benzoyloxy)-4-fluoro-5-(pyridin-2-ylthio)tetrahydrofuran-2-yl)methyl benzoate (3ad)

Following the **general procedure B** using **2q** as the sulfur electrophile, the desired product **3ad** was prepared as the colorless oil (26 mg, 58%) after purification by flash column chromatography on silica gel eluting with DCM/ethyl acetate (10:1 to 5:1).

^{1}H NMR (400 MHz, $CDCl_3$) δ 9.05 – 8.42 (m, 1H), 8.31 – 7.93 (m, 4H), 7.63 (t, *J* = 7.5 Hz, 1H), 7.56 (tt, *J* = 7.4, 1.8 Hz, 2H), 7.50 (t, *J* = 7.7 Hz, 2H), 7.42 (t, *J* = 7.7 Hz, 2H), 7.30 (d, *J* = 8.0 Hz, 1H), 7.09 (dd, *J* = 7.4, 4.9 Hz, 1H), 6.78 (d, *J* = 19.0 Hz, 1H), 5.64 (dd, *J* = 18.5, 3.3 Hz, 1H), 5.48 (d, *J* = 49.9 Hz, 1H), 4.71 (d, *J* = 5.2 Hz, 3H).

^{13}C NMR (101 MHz, $CDCl_3$) δ 166.37, 165.49, 156.16, 150.04, 136.84, 133.93, 133.25, 130.15, 130.00, 129.81, 128.92, 128.78, 128.49, 123.44, 120.91, 99.28 (d, *J* = 191.0 Hz), 87.78 (d, *J* = 29.4 Hz), 82.65, 77.71 (d, *J* = 31.3 Hz), 63.61.

HRMS (ESI$^+$): calcd for $C_{24}H_{20}FNO_5S$ $[M+H]^+$ 454.1119, found 454.1115.

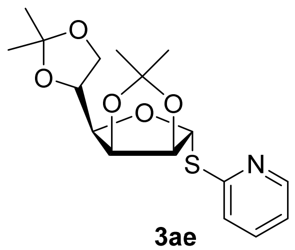

3ae

2-(((3a*S*,4*R*,6*R*,6a*S*)-6-((2,2-dimethyl-1,3-dioxolan-4-yl)methyl)-2,2-dimethyltetrahydrofuro[3,4-*d*][1,3]dioxol-4-yl)thio)pyridine (3ae)

Following the **general procedure B** using **2q** as the sulfur electrophile, the desired product **3ae** was prepared as the colorless oil (19 mg, 53%) after purification by flash column chromatography on silica gel eluting with DCM/ethyl acetate (10:1 to 5:1).

^{1}H NMR (400 MHz, $CDCl_3$) δ 8.48 (dd, *J* = 4.9, 1.8 Hz, 1H), 7.53 (td, *J* = 7.8, 1.9 Hz, 1H), 7.28 (d, *J* = 8.0 Hz, 1H), 7.05 (dd, *J* = 7.5, 4.9 Hz, 1H), 6.23 (s, 1H), 5.16 – 4.73 (m, 2H), 4.46 (ddd, *J* = 8.1, 6.2, 4.0 Hz, 1H), 4.07 (ddd, *J* = 14.9, 8.5, 4.7 Hz, 2H), 3.93 (dd, *J* = 8.8, 4.1 Hz, 1H), 1.52 (s, 3H), 1.44 (s, 3H), 1.36 (s, 3H), 1.35 (s, 3H).

^{13}C NMR (101 MHz, $CDCl_3$) δ 157.13, 149.91, 136.62, 123.54, 120.65, 113.30, 109.55, 89.20, 86.22, 81.36, 79.99, 72.90, 67.18, 27.14, 26.19, 25.34, 24.91.

HRMS (ESI^+): calcd for $C_{17}H_{23}NO_5S$ $[M+Na]^+$ 376.1189, found 376.1184.

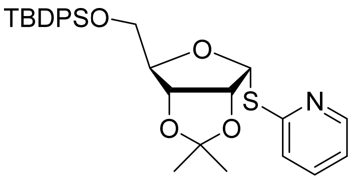

3af

2-(((3a*R*,4*R*,6*R*,6a*R*)-6-(((tert-butyldiphenylsilyl)oxy)methyl)-2,2-dimethyltetrahydrofuro[3,4-*d*][1,3]dioxol-4-yl)thio)pyridine (3af)

Following the **general procedure B** using **2q** as the sulfur electrophile, the desired product **3af** was prepared as the colorless oil (17 mg, 32%) after purification by flash column chromatography on silica gel eluting with DCM/ethyl acetate (10:1 to 5:1).

^{1}H NMR (400 MHz, $CDCl_3$) δ 8.44 (d, *J* = 4.9 Hz, 1H), 8.05 (s, 1H), 7.65 (dd, *J* = 7.4, 1.8 Hz, 4H), 7.52 (td, *J* = 7.7, 1.9 Hz, 1H), 7.45 – 7.29 (m, 6H), 7.23 (d, *J* = 8.1 Hz, 1H), 7.04 (dd, *J* = 7.5, 5.0 Hz, 1H), 6.34 (d, *J* = 5.8 Hz, 1H), 5.35 (q, *J* = 5.2 Hz, 1H), 4.50 (t, *J* = 5.5 Hz, 1H), 4.23 – 3.72 (m, 2H), 1.52 (s, 3H), 1.44 (s, 3H), 1.03 (s, 9H).

^{13}C NMR (101 MHz, $CDCl_3$) δ 160.28, 157.44, 149.95, 136.55, 135.74 (d, *J* = 4.6 Hz), 133.08 (d, *J* = 4.3 Hz), 129.93 (d, *J* = 2.6 Hz), 127.86, 121.72 (d, *J* = 246.3 Hz), 112.24, 81.77, 79.58, 73.41, 62.26, 27.37, 26.86 (2C), 25.74, 19.32.

HRMS (ESI^+): calcd for $C_{29}H_{35}NO_4SSi$ $[M+H]^+$ 522.2129, found 522.2130.

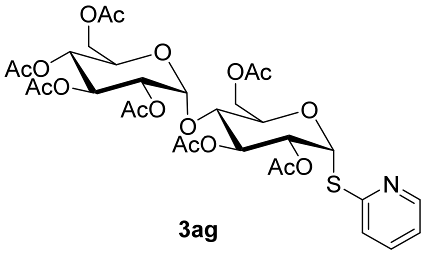

3ag

(2*R*,3*R*,4*S*,5*R*,6*R*)-2-(acetoxymethyl)-6-(((2*R*,3*R*,4*S*,5*R*,6*R*)-4,5-diacetoxy-2-(acetoxymethyl)-6-(pyridin-2-ylthio)tetrahydro-2*H*-pyran-3-yl)oxy)tetrahydro-2*H*-pyran-3,4,5-triyl triacetate (3ag)

Following the **general procedure B** uisng **2q** as the sulfur electrophile, the desired product **3ag** was prepared as the white solid (43 mg, 59%) after purification by flash column chromatography on silica gel eluting with DCM/ethyl acetate (10:1 to 2:1).

^{1}H NMR (400 MHz, $CDCl_3$) δ 8.47 (d, *J* = 3.2 Hz, 1H), 7.56 (td, *J* = 7.7, 1.9 Hz, 1H), 7.31 (d, *J* = 8.0 Hz, 1H), 7.08 (dd, *J* = 7.4, 4.7 Hz, 1H), 6.60 (d, *J* = 5.6 Hz, 1H), 5.78 – 5.32 (m, 3H), 5.16 (dd, *J* = 9.3, 5.6 Hz, 1H), 5.06 (t, *J* = 9.8 Hz, 1H), 4.89 (dd, *J* = 10.5, 3.9 Hz, 1H), 4.34 (dd, *J* = 9.7, 3.3 Hz, 2H), 4.22 (dt, *J* = 12.6, 4.9 Hz, 2H), 4.04 (dd, *J* = 12.4, 2.3 Hz, 1H), 4.01 – 3.87 (m, 2H), 2.08 (s, 6H), 2.06 – 1.97 (m, 15H).

^{13}C NMR (101 MHz, $CDCl_3$) δ 170.73, 170.67, 170.57, 170.04, 169.94, 169.82, 169.57, 155.55, 149.98, 136.81, 124.11, 121.10, 96.03, 81.22, 73.43, 72.94, 70.35, 70.13, 70.11, 69.52, 68.66, 68.14, 63.01, 61.62, 21.05, 20.88, 20.82, 20.79, 20.77, 20.75, 20.73.

HRMS (ESI$^+$): calcd for $C_{31}H_{39}NO_{17}S$ $[M+Na]^+$ 752.1831, found 752.1823.

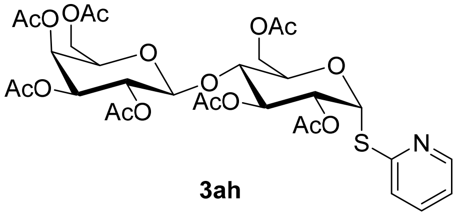

3ah

(2*R*,3*S*,4*S*,5*R*,6*S*)-2-(acetoxymethyl)-6-(((2*R*,3*R*,4*S*,5*R*,6*R*)-4,5-diacetoxy-2-(acetoxymethyl)-6-(pyridin-2-ylthio)tetrahydro-2*H*-pyran-3-yl)oxy)tetrahydro-2*H*-pyran-3,4,5-triyl triacetate (3ah)

Following the **general procedure B** using **2q** as the sulfur electrophile, the desired product **3ah** was prepared as the white solid (40 mg, 55%) after purification by flash column chromatography on silica gel eluting with DCM/ethyl acetate (5:1 to 2:1).

^{1}H NMR (400 MHz, $CDCl_3$) δ 8.45 (dd, *J* = 5.1, 1.9 Hz, 1H), 7.53 (td, *J* = 7.7, 2.0 Hz, 1H), 7.43 – 7.23 (m, 1H), 7.17 – 6.97 (m, 1H), 6.60 (d, *J* = 5.7 Hz, 1H), 5.51 – 5.30 (m, 2H), 5.18 (dd, *J* = 10.0, 5.7 Hz, 1H), 5.11 (dd, *J* = 10.4, 7.9 Hz, 1H), 4.94 (dd, *J* = 10.4, 3.4 Hz, 1H), 4.49 (d, *J* = 7.9 Hz, 1H), 4.33 (dd, *J* = 12.0, 2.1 Hz, 1H), 4.26 (ddd, *J* = 10.0, 4.9, 2.1 Hz, 1H), 4.20 – 4.03 (m, 3H),

3.88 (t, J = 6.9 Hz, 1H), 3.84 – 3.69 (m, 1H), 2.15 (s, 3H), 2.06 (s, 3H), 2.05 (s, 3H), 2.03 (s, 3H), 2.01 (s, 3H), 1.99 (s, 3H), 1.95 (s, 3H).

^{13}C NMR (101 MHz, $CDCl_3$) δ 170.47, 170.40, 170.29, 170.19, 170.01, 169.50, 169.15, 155.73, 149.92, 136.74, 123.86, 120.97, 101.18, 81.43, 76.47, 71.20, 70.86, 70.63, 70.51, 70.21, 69.21, 66.79, 62.13, 61.01, 20.98, 20.89, 20.85, 20.78 (2C), 20.64.

HRMS (ESI$^+$): calcd for $C_{31}H_{39}NO_{17}S$ $[M+H]^+$ 730.2011, found 730.2004.

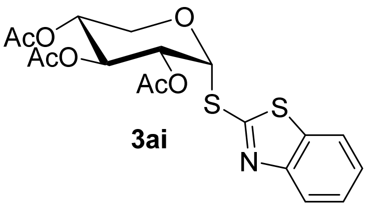

3ai

(2*R*,3*R*,4*S*,5*R*)-2-(benzo[*d*]oxazol-2-ylthio)tetrahydro-2H-pyran-3,4,5-triyl triacetate (3ai)

Following the **general procedure B** using **2r** as the sulfur electrophile, the desired product **3ai** was prepared as the light yellow oil (30 mg, 74%) after purification by flash column chromatography on silica gel eluting with DCM/ethyl acetate (20:1 to 5:1).

^{1}H NMR (400 MHz, $CDCl_3$) δ 7.93 (d, J = 8.1 Hz, 1H), 7.77 (d, J = 1.4 Hz, 1H), 7.44 (t, J = 7.7 Hz, 1H), 7.34 (t, J = 7.6 Hz, 1H), 6.52 (d, J = 5.1 Hz, 0.2H), 5.85 (d, J = 6.9 Hz, 0.8H), 5.37 (t, J = 9.2 Hz, 0.2H), 5.25 (t, J = 7.0 Hz, 0.8H), 5.24 – 5.16 (m, 0.2H), 5.13 (t, J = 6.9 Hz, 0.8H), 5.00 – 4.93 (m, 1H), 4.36 (dd, J = 12.2, 4.3 Hz, 0.8H), 4.00 (d, J = 7.8 Hz, 0.4H), 3.67 (dd, J = 12.3, 7.1 Hz, 0.8H), 2.10 (t, 7.2H), 2.07 (t, 1.8H).

^{13}C NMR (101 MHz, $CDCl_3$) δ 169.94 (α), 169.86 (β), 169.80 (α), 169.78 (α), 169.55 (β), 169.48 (β), 162.62 (β), 162.15 (α), 152.94 (α), 152.91 (β), 135.86 (α&β), 129.86 (α&β), 126.44 (α&β), 125.07 (α&β), 122.49 (α&β), 121.18 (α&β), 121.14 (α&β), 84.54 (α), 84.14 (β), 70.33 (β), 70.04 (α), 69.66 (α), 69.26 (β), 68.64 (α), 67.93 (β), 64.50 (β), 61.55 (α), 20.91 (α&β), 20.85 (α&β), 20.80 (α&β).

HRMS (ESI$^+$): calcd for $C_{18}H_{19}NO_7S_2$ $[M+Na]^+$ 448.0495, found 448.0490.

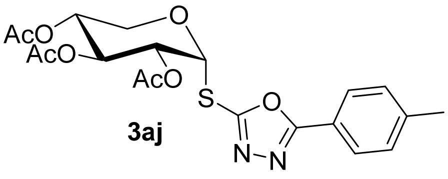

3aj

(2*R*,3*R*,4*S*,5*R*)-2-((5-(p-tolyl)-1,3,4-oxadiazol-2-yl)thio)tetrahydro-2*H*-pyran-3,4,5-triyl triacetate (3aj)

Following the **general procedure B** using disulfide **2y** as the sulfur electrophile, the desired product **3aj** was prepared as the white solid (30 mg, 67%) after purification by flash column chromatography on silica gel eluting with DCM/ethyl acetate (10:1 to 5:1).

^{1}H NMR (400 MHz, $CDCl_3$) δ 7.89 (d, *J* = 7.9 Hz, 2H), 7.29 (d, *J* = 7.9 Hz, 2H), 6.38 (d, *J* = 5.0 Hz, 0.2H), 5.74 (d, *J* = 6.7 Hz, 0.8H), 5.38 (t, *J* = 9.1 Hz, 0.2H), 5.22 (t, *J* = 7.0 Hz, 0.8H), 5.18 (t, 0.2H), 5.11 (t, *J* = 6.8 Hz, 0.8H), 5.07 – 4.88 (m, 1H), 4.33 (dd, *J* = 12.4, 4.3 Hz, 0.8H), 4.13 – 3.91 (m, 0.4H), 3.64 (dd, *J* = 12.4, 7.0 Hz, 0.8H), 2.42 (s, 3H), 2.11 (s, 6H), 2.08 (s, 3H).

^{13}C NMR (101 MHz, $CDCl_3$) δ 169.88 (α), 169.82 (β), 169.76 (α), 169.63 (α), 169.46 (β), 169.41 (β), 167.09 (α), 166.82 (β), 160.68 (β), 160.02 (α), 142.79 (α), 142.72 (β), 129.92 (α&β), 126.95 (α), 126.92 (β), 120.69 (α&β), 84.61 (α), 83.83 (β), 70.05 (β), 69.93 (α), 69.29 (α), 69.20 (β), 68.37 (α), 67.66 (β), 64.50 (β), 61.62 (α), 21.79 (α&β), 20.87, 20.81, 20.79, 20.74 (α).

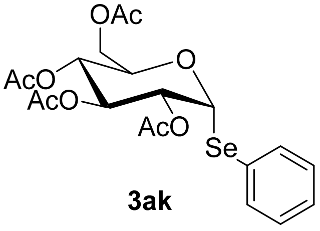

3ak

(2*R*,3*R*,4*S*,5*R*,6*R*)-2-(acetoxymethyl)-6-(phenylselanyl)tetrahydro-2*H*-pyran-3,4,5-triyl triacetate (3ak)

Following the **general procedure C**, the desired product **3ak** was prepared using the hexane/ethyl acetate (10:1 to 5:1) as eluent giving the light yellow oil. (34 mg, 69%).

^{1}H NMR (400 MHz, $CDCl_3$) δ 7.65 – 7.44 (m, 2H), 7.27 (d, *J* = 9.0 Hz, 3H), 6.20 (d, *J* = 5.6 Hz, 1H), 5.40 (t, *J* = 9.7 Hz, 1H), 5.19 – 4.93 (m, 2H), 4.69 – 4.44 (m, 1H), 4.27 (dd, *J* = 12.4, 5.0 Hz, 1H), 3.98 (dd, *J* = 12.4, 2.2 Hz, 1H), 2.09 (s, 3H), 2.05 (s, 3H), 2.04 (s, 3H), 2.02 (s, 3H).

^{13}C NMR (101 MHz, $CDCl_3$) δ 170.69, 170.11, 169.92, 169.73, 134.45, 129.41, 128.23, 127.60, 83.14, 71.36, 71.24, 69.92, 68.45, 61.96, 20.95, 20.81 (2C), 20.76.

HRMS (ESI^+): calcd for $C_{20}H_{24}O_9Se$ $[M+Na]^+$ 511.0478, found 511.0476.

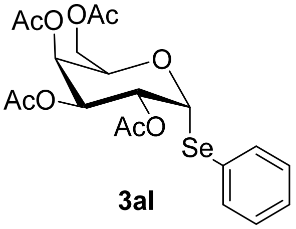

3al

(2R,3S,4S,5R,6R)-2-(acetoxymethyl)-6-(phenylselanyl)tetrahydro-2*H*-pyran-3,4,5-triyl triacetate (3al)

Following the **general procedure C**, the desired product **3al** was prepared using the hexane/ethyl acetate (10:1 to 5:1) as eluent giving the light yellow oil. (25 mg, 50%).

^{1}H NMR (400 MHz, $CDCl_3$) δ 7.88 – 7.42 (m, 2H), 7.42 – 6.93 (m, 3H), 6.27 (d, *J* = 4.8 Hz, 1H), 5.50 (dd, *J* = 3.1, 1.4 Hz, 1H), 5.37 – 5.05 (m, 2H), 4.68 (t, *J* = 6.5 Hz, 1H), 4.05 (qd, *J* = 11.4, 6.5 Hz, 2H), 2.14 (s, 3H), 2.10 (s, 3H), 2.01 (s, 3H), 1.97 (s, 3H).

^{13}C NMR (101 MHz, $CDCl_3$) δ 170.48, 170.24, 170.18, 170.03, 134.62, 129.30, 128.14, 127.58, 83.58, 69.04, 68.76, 68.65, 67.78, 61.74, 21.03, 20.78 (2C), 20.74.

HRMS (ESI^+): calcd for $C_{20}H_{24}O_9Se$ $[M+Na]^+$ 511.0478, found 511.0476.

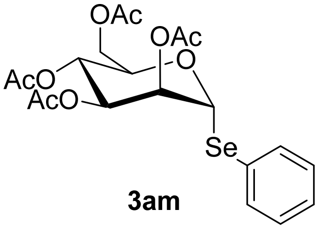

3am

(2*R*,3*R*,4*S*,5*S*,6*R*)-2-(acetoxymethyl)-6-(phenylselanyl)tetrahydro-2*H*-pyran-3,4,5-triyl triacetate (3am)

Following the **general procedure C**, the desired product **3am** was prepared using the hexane/ethyl acetate (10:1 to 5:1) as eluent giving the light yellow oil. (30 mg, 61%).

^{1}H NMR (400 MHz, $CDCl_3$) δ 7.59 (dt, *J* = 6.4, 1.9 Hz, 2H), 7.42 – 6.97 (m, 3H), 5.75 (d, *J* = 1.6 Hz, 1H), 5.56 (dd, *J* = 3.2, 1.6 Hz, 1H), 5.41 – 4.93 (m, 2H), 4.46 (ddd, *J* = 9.6, 5.8, 2.3 Hz, 1H), 4.31 (dd, *J* = 12.3, 5.7 Hz, 1H), 4.10 (dd, *J* = 12.3, 2.3 Hz, 1H), 2.13 (s, 3H), 2.07 (s, 3H), 2.06 (s, 3H), 2.01 (s, 3H).

^{13}C NMR (101 MHz, $CDCl_3$) δ 170.66, 170.01, 169.95, 169.82, 134.38, 129.47, 128.47, 128.35, 82.63, 71.62, 71.31, 69.90, 66.39, 62.49, 21.00, 20.83 (2C), 20.76.

HRMS (ESI^+): calcd for $C_{20}H_{24}O_9Se$ $[M+Na]^+$ 511.0478, found 511.0474.

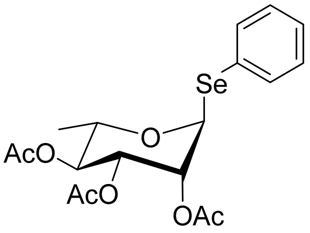

3an

(2*S*,3*S*,4*R*,5*R*,6*S*)-2-methyl-6-(phenylselanyl)tetrahydro-2*H*-pyran-3,4,5-triyl triacetate (3an)

Following the **general procedure C**, the desired product **3an** was prepared using the hexane/ethyl acetate (10:1 to 5:1) as eluent giving the light yellow oil. (22 mg, 45%).

^{1}H NMR (400 MHz, $CDCl_3$) δ 7.74 – 7.48 (m, 2H), 7.46 – 6.96 (m, 3H), 5.65 (d, *J* = 1.6 Hz, 1H), 5.56 (dd, *J* = 3.4, 1.6 Hz, 1H), 5.28 (dd, *J* = 10.2, 3.3 Hz, 1H), 5.15 (t, *J* = 9.9 Hz, 1H), 4.51 – 3.92 (m, 1H), 2.12 (s, 3H), 2.08 (s, 3H), 2.01 (s, 3H), 1.25 (d, *J* = 6.1 Hz, 4H).

^{13}C NMR (101 MHz, $CDCl_3$) δ 170.11, 170.09, 170.06, 134.34, 129.46, 128.81, 128.31, 82.82, 72.11, 71.16, 69.86, 69.77, 21.04, 20.94, 20.81, 17.44.

HRMS (ESI^+): calcd for $C_{18}H_{23}O_7Se$ $[M+Na]^+$ 453.0423, found 453.0415.

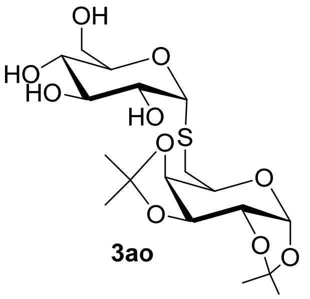

3ao

(2*R*,3*S*,4*S*,5*R*,6*R*)-2-(hydroxymethyl)-6-((((3a*R*,5*S*,5a*R*,8a*S*,8b*R*)-2,2,7,7-tetramethyltetrahydro-5*H*-bis([1,3]dioxolo)[4,5-b:4',5'-d]pyran-5-yl)methyl)thio)tetrahydro-2*H*-pyran-3,4,5-triol (3ao)

Following the **general procedure B** using **2ab** as the sulfur electrophile, the impure product was hydrolyzed using catalytic sodium methoxide in anhydrous methanol. The desired product **3ao** was prepared as the white solid (34 mg, 78%) after purification by flash column chromatography on silica gel eluting with ethyl acetate/MeOH (5:1).

^{1}H NMR (400 MHz, $CDCl_3$) δ 5.51 (d, *J* = 4.9 Hz, 1H), 5.39 (d, *J* = 5.2 Hz, 1H), 4.96 (s, 1H), 4.62 (dd, *J* = 7.7, 2.4 Hz, 1H), 4.58 (s, 1H), 4.38 (d, *J* = 8.0 Hz, 1H), 4.30 (dd, *J* = 5.0, 2.4 Hz, 1H), 4.17 (s, 1H), 3.98 (d, *J* = 8.0 Hz, 2H), 3.90 – 3.75 (m, 3H), 3.55 (dt, *J* = 23.2, 8.9 Hz, 2H), 3.34 (s, 1H), 2.85 (ddt, *J* = 20.0, 13.4, 7.3 Hz, 2H), 1.54 (s, 3H), 1.43 (s, 3H), 1.35 (s, 3H), 1.32 (s, 3H).

^{13}C NMR (101 MHz, $CDCl_3$) δ 109.58, 108.98, 96.78, 88.34, 75.07, 73.16, 71.70, 71.29, 70.87, 70.71 (d, *J* = 16.16), 69.97, 67.31, 61.87, 53.57, 32.16, 26.23, 26.16, 25.05, 24.72.

HRMS (ESI$^+$): calcd for $C_{18}H_{30}O_{10}S$ $[M+H]^+$ 439.1632, found 439.1627.

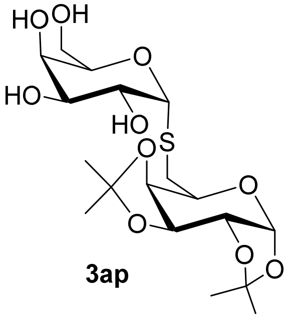

3ap

(2*R*,3*R*,4*S*,5*R*,6*R*)-2-(hydroxymethyl)-6-((((3a*R*,5*S*,5a*R*,8a*S*,8b*R*)-2,2,7,7-tetramethyltetrahydro-5*H*-bis([1,3]dioxolo)[4,5-b:4',5'-d]pyran-5-yl)methyl)thio)tetrahydro-2*H*-pyran-3,4,5-triol (3ap)

Following the **general procedure B** using **2ab** as the sulfur electrohphile, the impure product was hydrolyzed using catalytic sodium methoxide in anhydrous methanol. The desired product **3ap** was prepared using ethyl acetate/MeOH (5:1) as the eluent giving the white solid (36 mg, 83%).

^{1}H NMR (400 MHz, $CDCl_3$) δ 5.52 (d, *J* = 4.9 Hz, 1H), 5.47 (d, *J* = 5.3 Hz, 1H), 4.63 (dd, *J* = 7.9, 2.4 Hz, 1H), 4.50 – 4.38 (m, 1H), 4.31 (dd, *J* = 5.0, 2.4 Hz, 1H), 4.21 (d, *J* = 5.2 Hz, 1H), 4.15 (s,

1H), 4.04 (d, J = 7.2 Hz, 2H), 3.97 (s, 1H), 3.89 (dd, J = 12.1, 6.3 Hz, 1H), 3.77 (d, J = 12.1 Hz, 1H), 3.70 (s, 1H), 3.60 (d, J = 10.0 Hz, 1H), 3.41 (s, 1H), 3.12 (s, 1H), 2.87 (qd, J = 13.5, 7.2 Hz, 2H), 1.55 (s, 3H), 1.44 (s, 3H), 1.35 (s, 3H), 1.32 (s, 3H).

^{13}C NMR (101 MHz, $CDCl_3$) δ 109.63, 109.03, 96.84, 88.97, 71.94, 71.61, 71.19, 70.83, 70.72, 70.15, 68.81, 67.24, 62.89, 32.24, 26.21, 26.14, 25.02, 24.71.

HRMS (ESI$^+$): calcd for $C_{18}H_{30}O_{10}S$ [M+H]$^+$ 439.1632, found 439.1624.

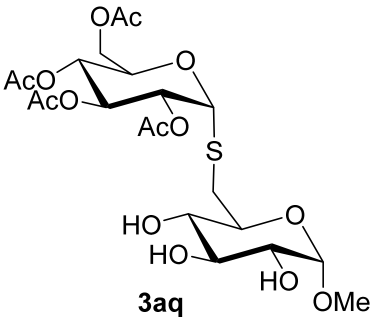

3aq

(2*R*,3*R*,4*S*,5*R*,6*R*)-2-(acetoxymethyl)-6-((((2*S*,3*S*,4*S*,5*R*,6*S*)-3,4,5-trihydroxy-6-methoxytetrahydro-2*H*-pyran-2-yl)methyl)thio)tetrahydro-2*H*-pyran-3,4,5-triyl triacetate (3aq)

Following the **general procedure B** using **2ai** as the sulfur electrophile, the desired product **3aq** was prepared using DCM/MeOH (50:1 to 10:1) as eluent giving the white solid. (39 mg, 71%).

^{1}H NMR (400 MHz, $CDCl_3$) δ 5.77 (d, J = 5.7 Hz, 1H), 5.37 (t, J = 9.8 Hz, 1H), 5.17 – 4.93 (m, 2H), 4.72 (d, J = 3.9 Hz, 1H), 4.61 – 4.40 (m, 1H), 4.30 (dd, J = 12.4, 4.3 Hz, 1H), 4.11 (dd, J = 12.6, 2.5 Hz, 1H), 3.86 – 3.64 (m, 2H), 3.47 (dd, J = 10.4, 3.3 Hz, 2H), 4.42 (s, 3H), 3.05 (dd, J = 13.7, 2.7 Hz, 1H), 2.73 (dd, J = 13.7, 6.7 Hz, 1H), 2.08 (s, 3H), 2.05 (s, 3H), 2.03 (s, 4H), 2.01 (s, 3H).

^{13}C NMR (101 MHz, $CDCl_3$) δ 171.01, 170.07, 170.00, 169.76, 99.39, 81.94, 74.65, 72.47, 72.37, 70.95, 70.54, 70.33, 68.71, 67.73, 62.06, 55.53, 30.89, 20.88, 20.86, 20.79, 20.74.

HRMS (ESI$^+$): calcd for $C_{21}H_{32}O_{14}S$ [M+H]$^+$ 541.1586, found 541.1583.

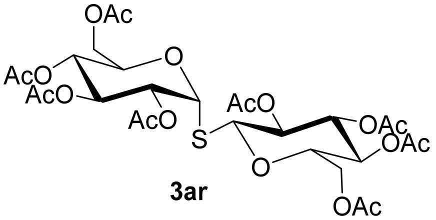

3ar

(2*S*,3*S*,4*R*,5*S*,6*R*)-2-(acetoxymethyl)-6-(((2*R*,3*R*,4*S*,5*R*,6*R*)-3,4,5-triacetoxy-6-(acetoxymethyl)tetrahydro-2H-pyran-2-yl)thio)tetrahydro-2*H*-pyran-3,4,5-triyl triacetate (3ar)

Following the **general procedure B** using **2aa** as the sulfur electrophile, the desired product **3ar** was prepared using DCM/acetone (20:1) as eluent giving the white solid (60 mg, 86%).

^{1}H NMR (400 MHz, $CDCl_3$) δ 5.93 (d, *J* = 5.7 Hz, 1H), 5.29 (t, *J* = 9.8 Hz, 1H), 5.20 – 4.90 (m, 5H), 4.56 (d, *J* = 10.1 Hz, 1H), 4.38 (d, *J* = 10.2 Hz, 2H), 4.21 – 4.05 (m, 3H), 3.71 (ddd, *J* = 10.1, 4.6, 2.4 Hz, 1H), 2.09 (s, 3H), 2.09 (s, 3H), 2.01 (s, 12H), 2.00 (s, 3H), 1.99 (s, 3H).

^{13}C NMR (101 MHz, $CDCl_3$) δ 170.73, 170.71, 170.25, 169.96, 169.76, 169.65, 169.46, 169.21, 82.83, 82.25, 76.47, 73.86, 71.10, 70.69, 70.43, 68.65, 67.98, 67.95, 62.10, 61.30, 20.84 (3C), 20.76, 20.71 (4C).

HRMS (ESI$^+$): calcd for $C_{28}H_{38}O_{18}S$ $[M+H]^+$ 695.1852, found 695.1852.

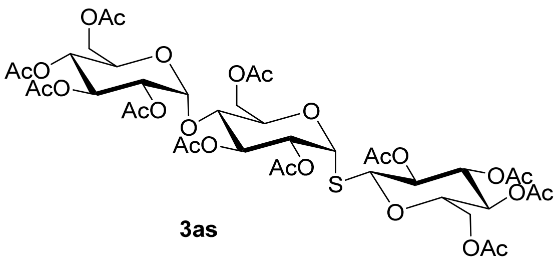

3as

(2*R*,3*R*,4*S*,5*R*,6*S*)-2-(acetoxymethyl)-6-(((2*R*,3*R*,4*S*,5*R*,6*R*)-3,4-diacetoxy-6-(acetoxymethyl)-5-(((2*R*,3*R*,4*S*,5*R*,6*R*)-3,4,5-triacetoxy-6-(acetoxymethyl)tetrahydro-2*H*-pyran-2-yl)oxy)tetrahydro-2*H*-pyran-2-yl)thio)tetrahydro-2*H*-pyran-3,4,5-triyl triacetate (3as)

Following the **general procedure B** using **2aa** as the sulfur electrophile, the desired product **3as** was prepared using DCM/acetone (20:1) as eluent giving the white solid. (72 mg, 73%).

^{1}H NMR (400 MHz, $CDCl_3$) δ 5.79 (d, *J* = 5.6 Hz, 1H), 5.38 (d, *J* = 4.0 Hz, 1H), 5.33 (q, *J* = 9.3, 8.6 Hz, 2H), 5.16 (t, *J* = 9.3 Hz, 1H), 5.10 – 4.98 (m, 3H), 4.84 (ddd, *J* = 10.6, 4.8, 2.9 Hz, 2H), 4.56 (d, *J* = 10.0 Hz, 1H), 4.45 (dd, *J* = 12.5, 2.5 Hz, 1H), 4.38 – 4.13 (m, 5H), 4.09 – 3.96 (m, 2H), 3.92 (dd, *J* = 10.2, 2.8 Hz, 1H), 3.72 (dd, *J* = 9.7, 3.4 Hz, 1H), 2.13 (s, 3H), 2.09 (s, 3H), 2.07 (s, 3H), 2.04 (s, 3H), 2.01 (s, 3H), 2.00 (s, 9H), 1.98 (s, 9H).

^{13}C NMR (101 MHz, $CDCl_3$) δ 170.77, 170.73, 170.61, 170.49, 170.24, 169.95, 169.83, 169.58, 169.53, 169.44, 169.15, 95.72, 82.86, 81.85, 76.44, 73.88, 72.37, 72.30, 71.09 (2C), 70.12, 69.42, 69.15, 68.54, 68.00, 67.85, 62.33, 61.92, 61.35, 20.97, 20.96, 20.83, 20.78, 20.74, 20.72, 20.69, 20.66, 20.62.

HRMS (ESI$^+$): calcd for $C_{40}H_{54}O_{26}S$ $[M+H]^+$ 983.2697, found 983.2693.

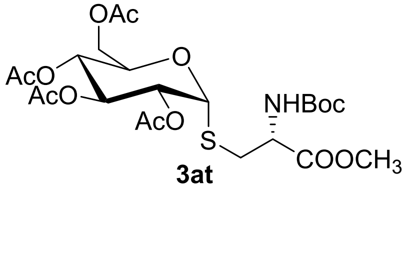

3at

(2*R*,3*R*,4*S*,5*R*,6*R*)-2-(acetoxymethyl)-6-(((*R*)-2-((*tert*-butoxycarbonyl)amino)-3-methoxy-3-oxopropyl)thio)tetrahydro-2*H*-pyran-3,4,5-triyl triacetate (3at)

Following the **general procedure B** uisng **2ac** as the sulfur electrophile, the desired product **3at** was prepared using DCM/ethyl acetate (2:1) as eluent giving the colorless oil. (45 mg, 80%).

^{1}H NMR (400 MHz, $CDCl_3$) δ 5.66 (d, *J* = 8.4 Hz, 1H), 5.58 (d, *J* = 5.8 Hz, 1H), 5.28 (t, *J* = 9.9 Hz, 1H), 5.15 – 4.94 (m, 2H), 4.80 – 4.51 (m, 1H), 4.45 – 4.27 (m, 2H), 4.15 (dd, *J* = 12.3, 1.9 Hz, 1H), 3.76 (s, 4H), 3.17 (dd, *J* = 14.3, 5.0 Hz, 1H), 2.98 (dd, *J* = 14.2, 3.8 Hz, 1H), 2.11 (s, 4H), 2.06 (s, 4H), 2.03 (s, 5H), 2.01 (s, 3H), 1.44 (s, 11H).

^{13}C NMR (101 MHz, $CDCl_3$) δ 171.05, 170.74, 170.01, 169.98, 169.68, 155.32, 84.02, 80.39, 77.36, 70.71, 70.25, 68.38, 62.00, 53.80, 52.77, 34.72, 28.41 (3C), 20.86, 20.81, 20.78, 20.73.

HRMS (ESI^+): calcd for $C_{23}H_{35}NO_{13}S$ $[M+H]^+$ 566.1902, found 566.1896.

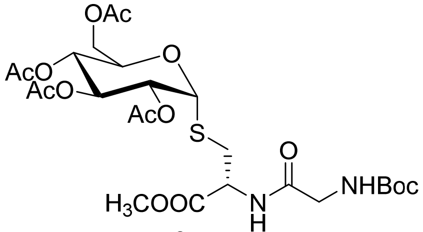

3au

(2*R*,3*R*,4*S*,5*R*,6*R*)-2-(acetoxymethyl)-6-(((*R*)-2-(2-((*tert*-butoxycarbonyl)amino)acetamido)-3-methoxy-3-oxopropyl)thio)tetrahydro-2*H*-pyran-3,4,5-triyl triacetate (3au)

Following the **general procedure B** using **2ad** as the sulfur electrophile, the desired product **3au** was prepared using DCM/MeOH (50:1) as eluent giving the colorless oil. (41 mg, 72%).

^{1}H NMR (400 MHz, $CDCl_3$) δ 7.09 (d, *J* = 8.1 Hz, 1H), 5.54 (d, *J* = 5.8 Hz, 1H), 5.45 – 5.18 (m, 2H), 5.07 – 4.84 (m, 3H), 4.62 – 4.17 (m, 3H), 3.83 (t, *J* = 5.9 Hz, 2H), 3.75 (s, 3H), 3.14 (dd, *J* = 14.5, 5.3 Hz, 1H), 3.03 (dd, *J* = 14.5, 3.8 Hz, 1H), 2.11 (s, 3H), 2.05 (s, 3H), 2.03 (s, 3H), 2.00 (s, 3H), 1.46 (s, 9H).

^{13}C NMR (101 MHz, $CDCl_3$) δ 170.76, 170.39, 169.98, 169.94, 169.68, 169.52, 156.03, 84.04, 80.40, 70.66, 70.12, 68.67, 68.42, 61.83, 52.91, 52.46, 44.33, 34.31, 28.44 (3C), 20.89, 20.81, 20.74, 20.71.

HRMS (ESI^+): calcd for $C_{25}H_{38}N_2O_{14}S$ $[M+H]^+$ 623.2117, found 623.2114.

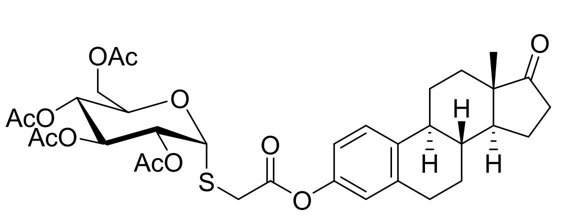

3av

(2*R*,3*R*,4*S*,5*R*,6*R*)-2-(acetoxymethyl)-6-((2-(((8*R*,9*S*,13*S*,14*S*)-13-methyl-17-oxo-7,8,9,11,12,13,14,15,16,17-decahydro-6*H*-cyclopenta[a]phenanthren-3-yl)oxy)-2-oxoethyl)thio)tetrahydro-2*H*-pyran-3,4,5-triyl triacetate (3av)

Following the **general procedure B** using the **2af** as the sulfur electrophile, the desired product **3av** was prepared using hexane/ethy acetate as eluent first, then dichloromethane/ethyl acetate (20:1) as eluent giving the white solid. (31 mg, 47%).

^{1}H NMR (400 MHz, $CDCl_3$) δ 7.28 (d, *J* = 8.8 Hz, 1H), 6.85 (dd, *J* = 8.4, 2.5 Hz, 1H), 6.80 (d, *J* = 2.5 Hz, 1H), 5.87 (d, *J* = 5.8 Hz, 1H), 5.39 (t, *J* = 9.8 Hz, 1H), 5.19 – 5.04 (m, 2H), 4.46 (ddd, *J* = 10.4, 4.3, 2.2 Hz, 1H), 4.29 (dd, *J* = 12.6, 4.2 Hz, 1H), 4.02 (dd, *J* = 12.5, 2.2 Hz, 1H), 3.60 (d, *J* = 15.5 Hz, 1H), 3.43 (d, *J* = 15.6 Hz, 1H), 2.90 (dd, *J* = 9.2, 4.2 Hz, 2H), 2.50 (dd, *J* = 18.8, 8.7 Hz, 1H), 2.39 (dd, *J* = 10.5, 4.7 Hz, 1H), 2.37 – 2.21 (m, 1H), 2.23 – 2.11 (m, 1H), 2.07 (s, 6H), 2.02 (d, *J* = 2.4 Hz, 6H), 1.99 – 1.91 (m, 1H), 1.74 – 1.37 (m, 8H), 0.91 (s, 3H).

^{13}C NMR (101 MHz, $CDCl_3$) δ 220.84, 170.70, 170.05, 169.85, 169.69, 168.57, 148.51, 138.33, 137.97, 126.65, 121.37, 118.53, 82.59, 70.56, 70.47, 68.39, 68.35, 61.75, 50.57, 48.07, 44.28, 38.09, 35.98, 31.81, 31.68, 29.50, 26.43, 25.88, 21.72, 20.84, 20.82, 20.79, 20.73, 13.97.

HRMS (ESI$^+$): calcd for $C_{34}H_{42}O_{12}S$ [M+Na]$^+$ 697.2289, found 697.2277.

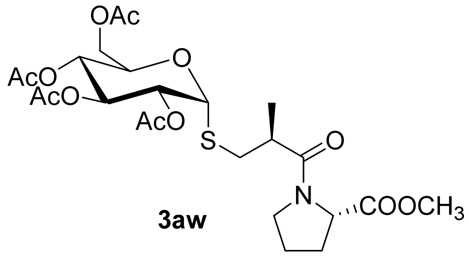

3aw

(2*R*,3*R*,4*S*,5*R*,6*R*)-2-(acetoxymethyl)-6-(((*S*)-3-((*S*)-2-(methoxycarbonyl)pyrrolidin-1-yl)-2-methyl-3-oxopropyl)thio)tetrahydro-2*H*-pyran-3,4,5-triyl triacetate (3aw)

Following the **general procedure B** using **2ae** as the sulfur electrophile, the desired product **3aw** was prepared using dichloromethane/ethyl acetate (5:1) as eluent giving the colorless oil. (42 mg, 75%).

^{1}H NMR (400 MHz, $CDCl_3$) δ 5.65 (d, *J* = 5.8 Hz, 1H), 5.34 (t, *J* = 9.8 Hz, 1H), 5.13 – 4.83 (m, 2H), 4.50 (dd, *J* = 8.6, 3.8 Hz, 1H), 4.37 (dq, *J* = 8.0, 2.4 Hz, 1H), 4.27 (dd, *J* = 12.3, 4.8 Hz, 1H), 4.06 (dd, *J* = 12.2, 2.4 Hz, 1H), 3.70 (s, 3H), 3.69 – 3.42 (m, 2H), 2.92 (dd, *J* = 12.9, 7.0 Hz, 1H), 2.72 (q, *J* = 7.0 Hz, 1H), 2.50 (dd, *J* = 13.1, 7.2 Hz, 1H), 2.20 (ddd, *J* = 11.4, 8.0, 5.4 Hz, 1H), 2.13 – 2.09 (m, 1H), 2.07 (s, 3H), 2.05 (s, 1H), 2.04 (s, 3H), 2.02 (s, 3H), 2.00 (s, 3H), 1.97 – 1.63 (m, 1H), 1.22 (d, *J* = 7.0 Hz, 3H).

^{13}C NMR (101 MHz, $CDCl_3$) δ 173.40, 172.79, 170.70, 170.05, 169.92, 169.70, 81.84, 70.88, 70.55, 68.68, 67.79, 62.02, 58.78, 52.29, 47.01, 38.43, 32.67, 29.16, 24.94, 20.86, 20.85, 20.79, 20.73, 16.91.

HRMS (ESI$^+$): calcd for $C_{24}H_{35}NO_{12}S$ [M+H]$^+$ 562.1953, found 562.1948.

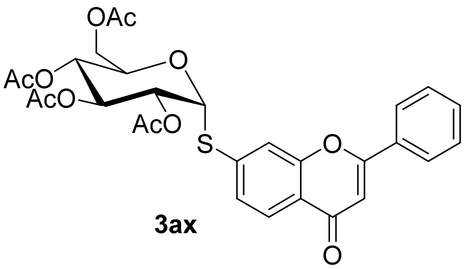

3ax

(2*R*,3*R*,4*S*,5*R*,6*R*)-2-(acetoxymethyl)-6-((4-oxo-2-phenyl-4*H*-chromen-7-yl)thio)tetrahydro-2*H*-pyran-3,4,5-triyl triacetate (3ax)

Following the **general procedure A** using **2ag** as the sulfur electrophile, the desired product **3ax** was prepared using the hexane/ethyl acetate (10:1 to 5:1) as eluent giving the white solid. (29 mg, 50%).

^{1}H NMR (400 MHz, $CDCl_3$) δ 8.13 (d, *J* = 8.4 Hz, 1H), 7.99 – 7.88 (m, 2H), 7.67 (d, *J* = 1.7 Hz, 1H), 7.60 – 7.49 (m, 3H), 7.42 (dd, *J* = 8.4, 1.8 Hz, 1H), 6.80 (s, 1H), 6.18 (d, *J* = 5.7 Hz, 1H), 5.46 (t, *J* = 9.8 Hz, 1H), 5.18 (dd, *J* = 10.4, 5.7 Hz, 1H), 5.11 (t, *J* = 9.8 Hz, 1H), 4.50 (ddd, *J* = 10.3, 5.4, 2.2 Hz, 1H), 4.31 (dd, *J* = 12.4, 5.3 Hz, 1H), 4.05 (dd, *J* = 12.4, 2.2 Hz, 1H), 2.11 (s, 3H), 2.06 (s, 6H), 1.97 (s, 3H).

^{13}C NMR (101 MHz, $CDCl_3$) δ 177.81, 170.55, 170.03, 169.92, 169.69, 163.52, 156.43, 141.03, 131.92, 131.62, 129.25, 126.52, 126.45, 126.28, 122.68, 118.20, 107.96, 83.78, 70.58, 70.41, 68.97, 68.49, 61.93, 20.85, 20.79, 20.73 (2C).

HRMS (ESI$^+$): calcd for $C_{29}H_{28}O_{11}S$ [M+Na]$^+$ 607.1245, found 607.1233.

10. Spectral Data

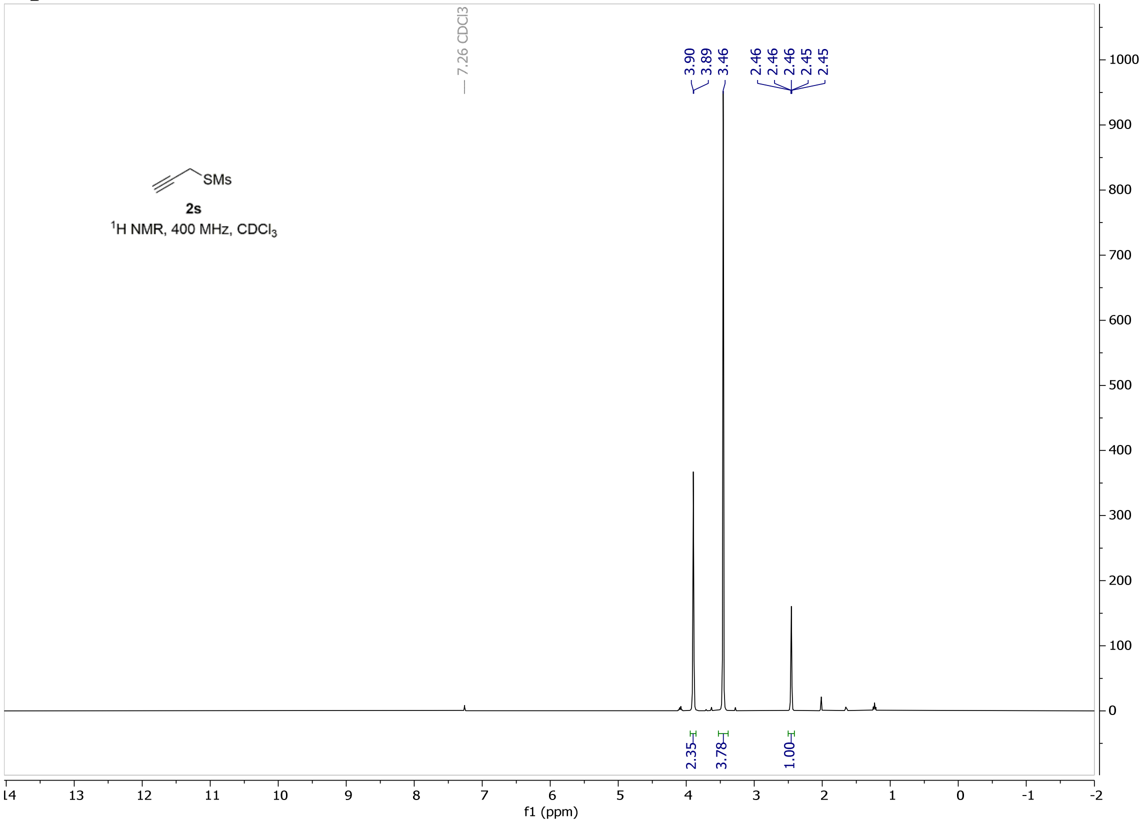

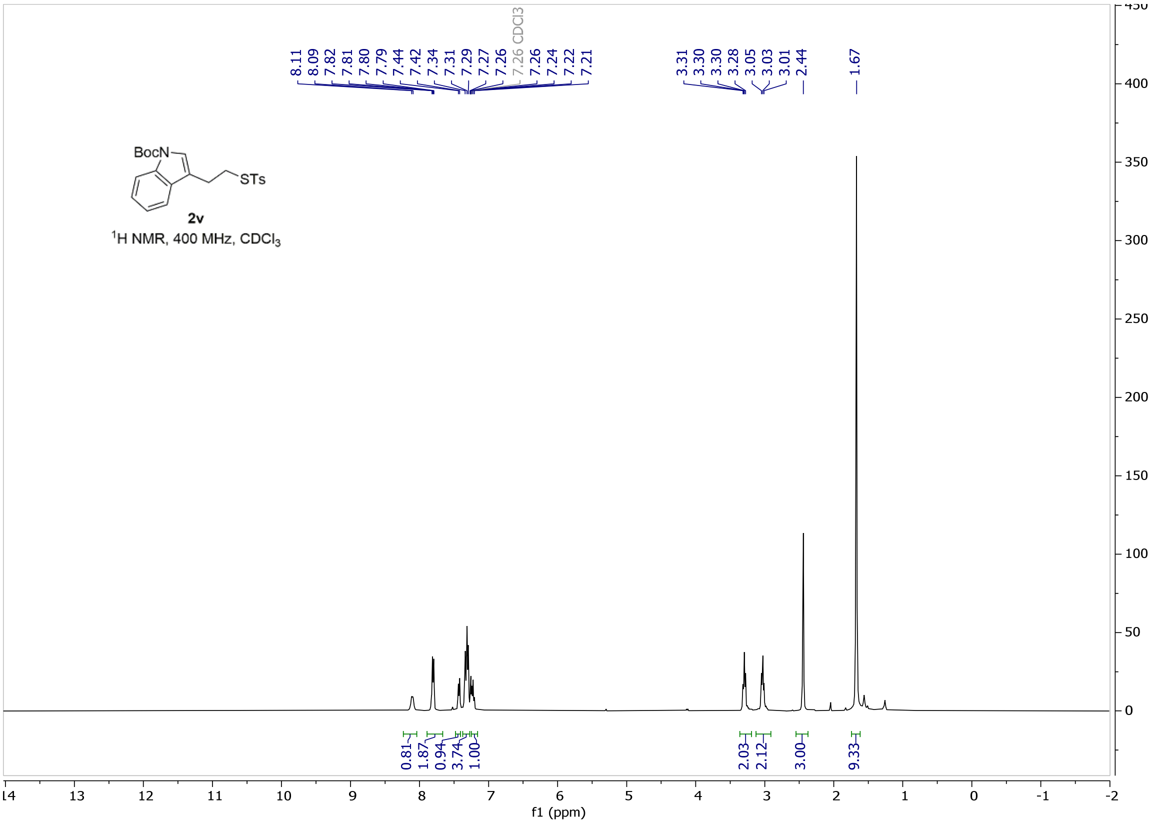

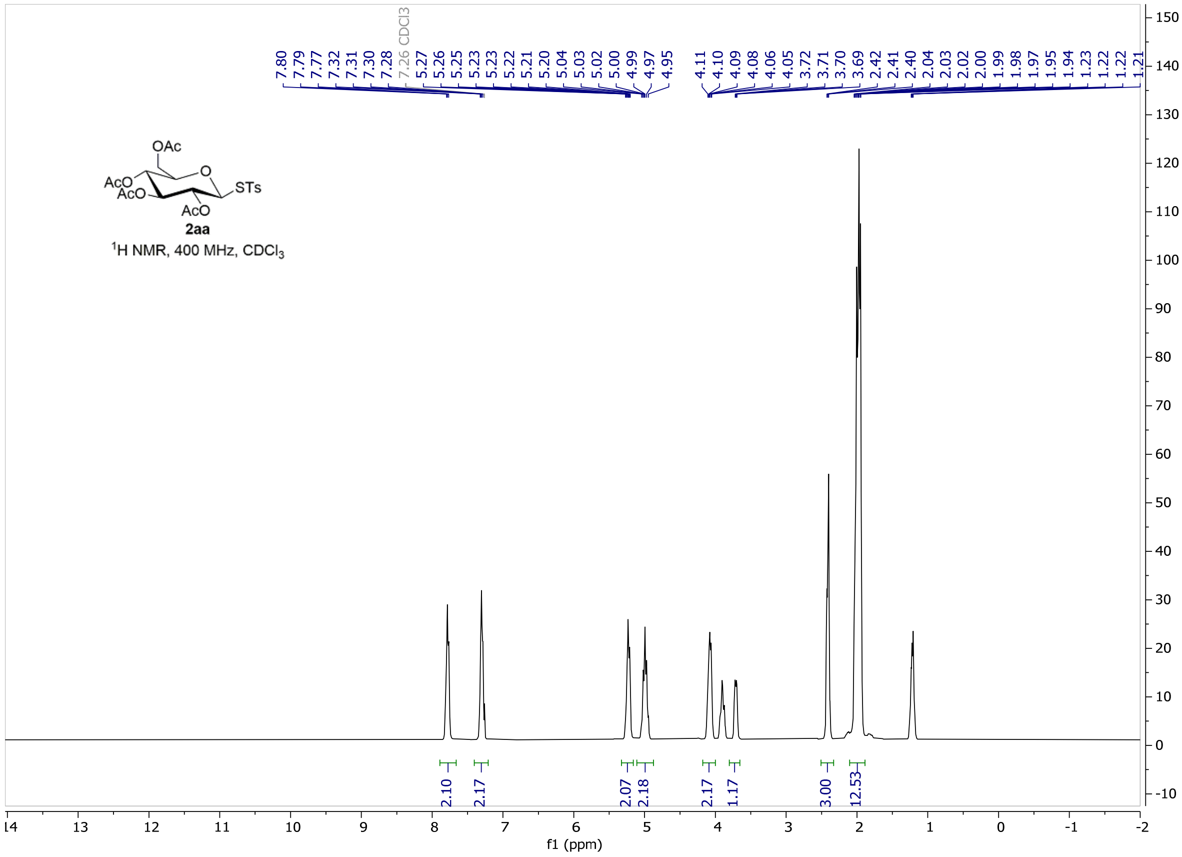

2aa
^{1}H NMR, 400 MHz, $CDCl_3$
f1 (ppm)

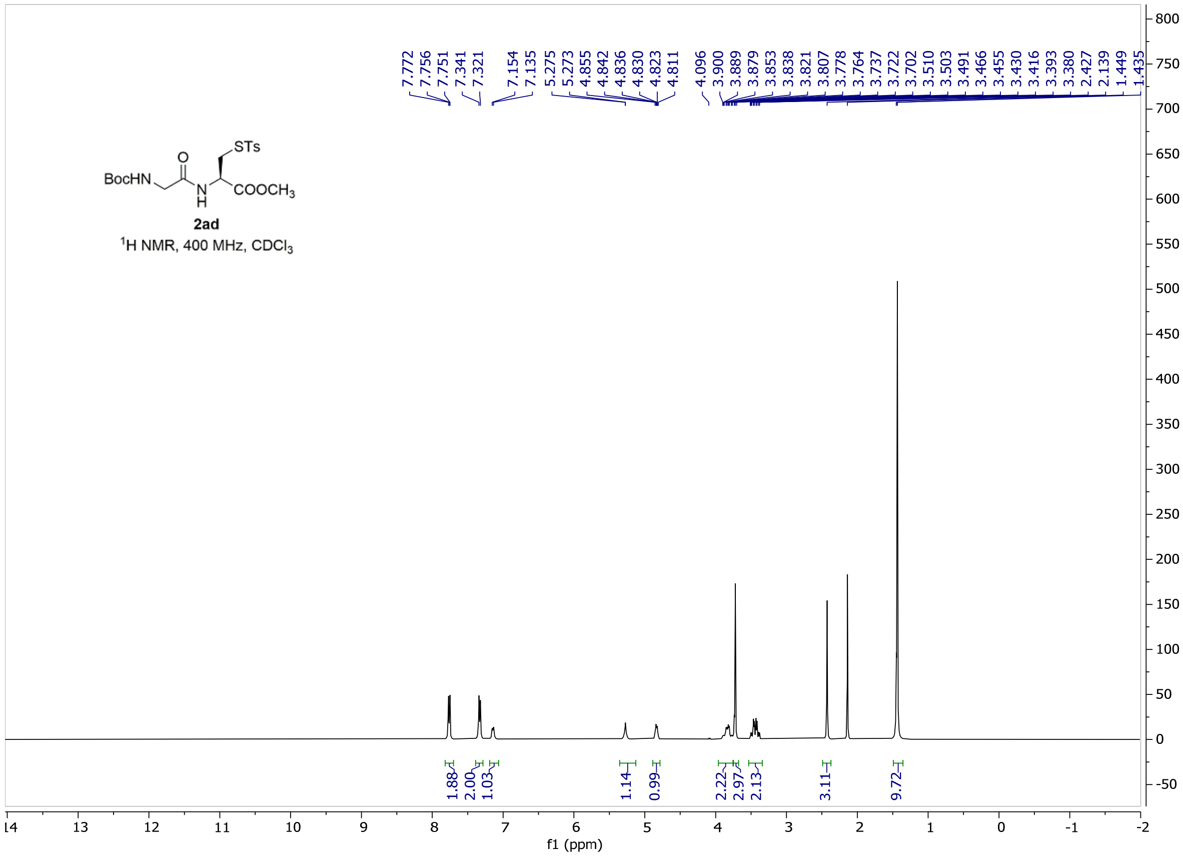

2ad
^{1}H NMR, 400 MHz, $CDCl_3$
f1 (ppm)

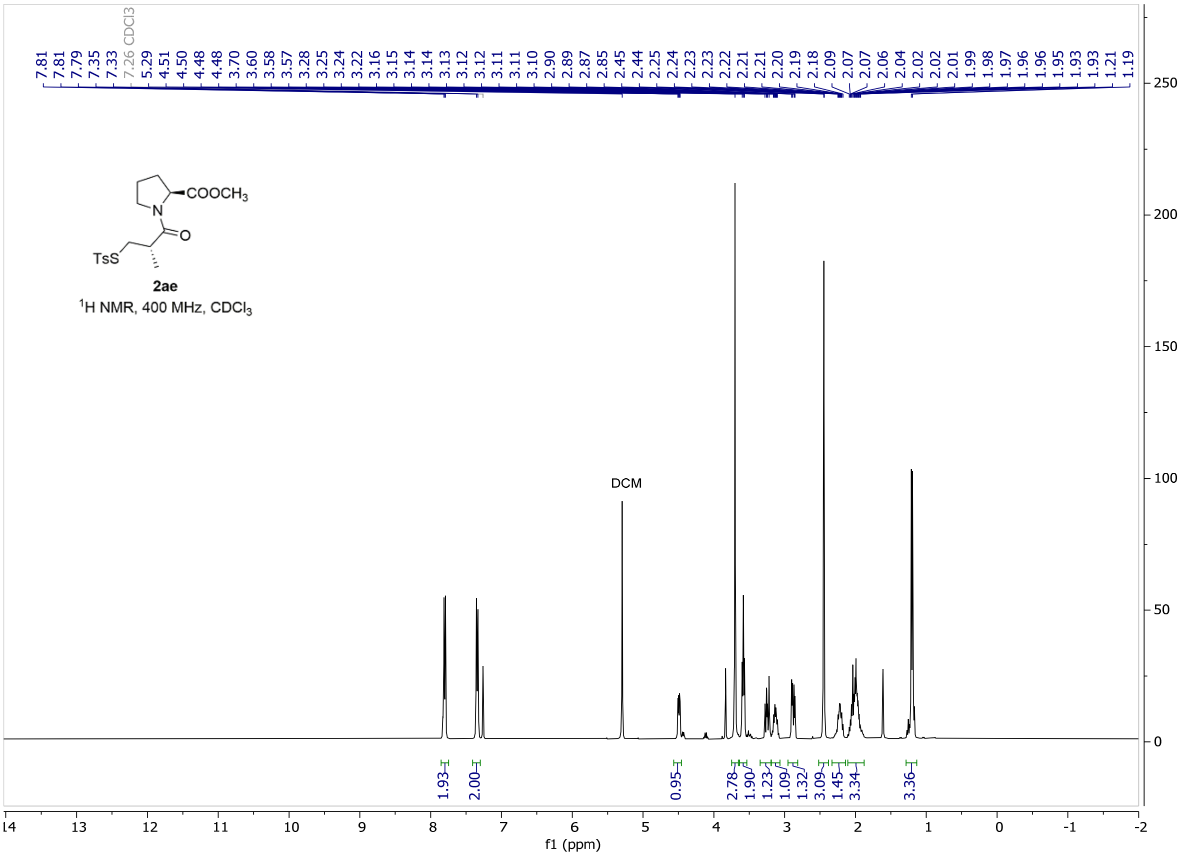

TsS
COOCH3
2ae
1H NMR, 400 MHz, CDCl3
DCM
7.26 CDCl3
f1 (ppm)

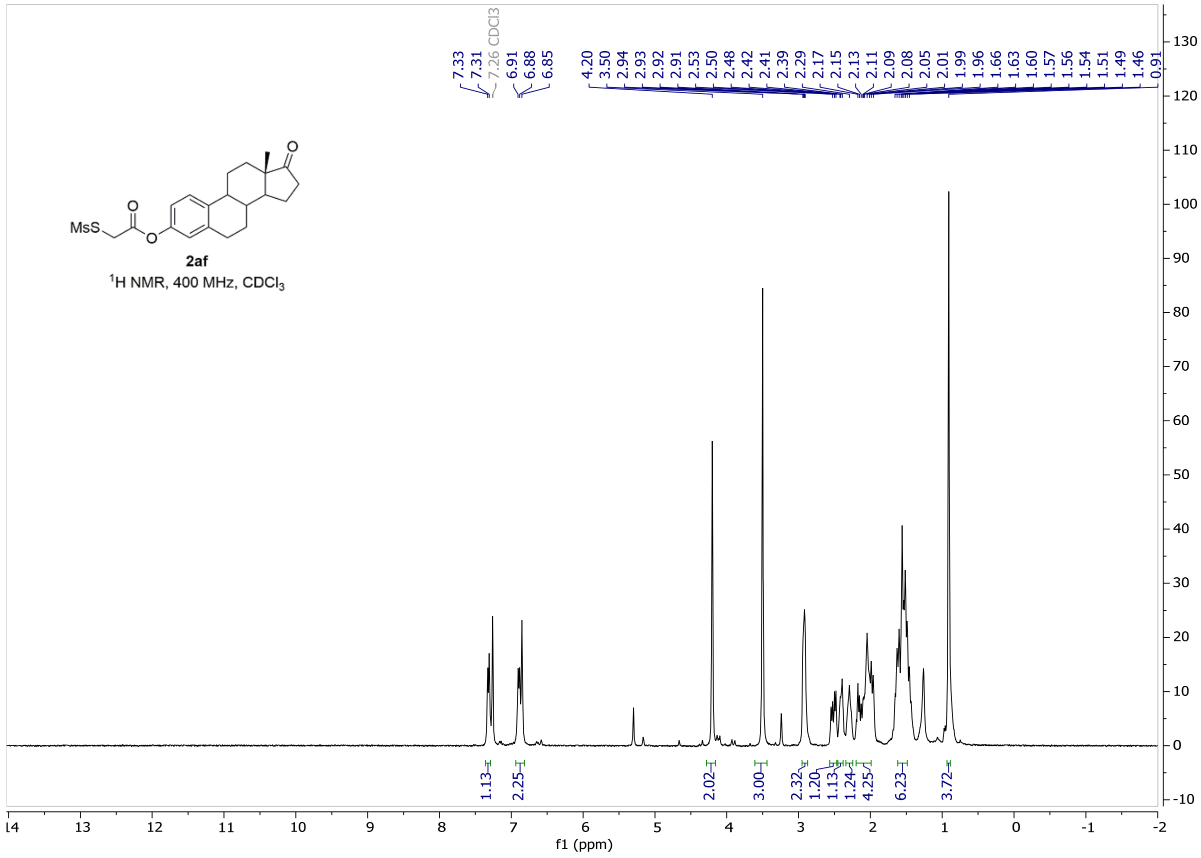

MsS
2af
1H NMR, 400 MHz, CDCl3
7.26 CDCl3
f1 (ppm)

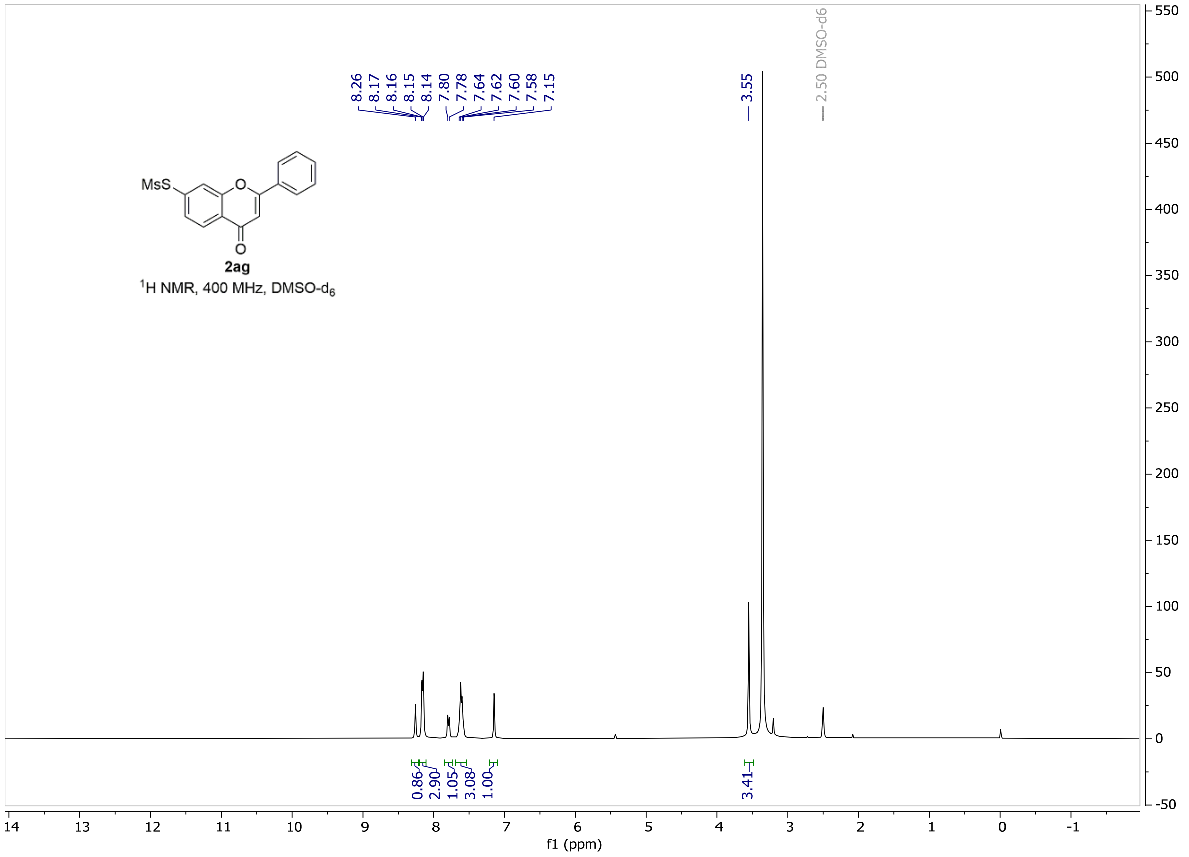

8.26
8.17
8.16
8.15
8.14
7.80
7.78
7.64
7.62
7.60
7.58
7.15
3.55
2.50 DMSO-d6
MsS
O
O
2ag
1H NMR, 400 MHz, DMSO-d6
0.86
2.90
1.05
3.08
1.00
3.41
f1 (ppm)

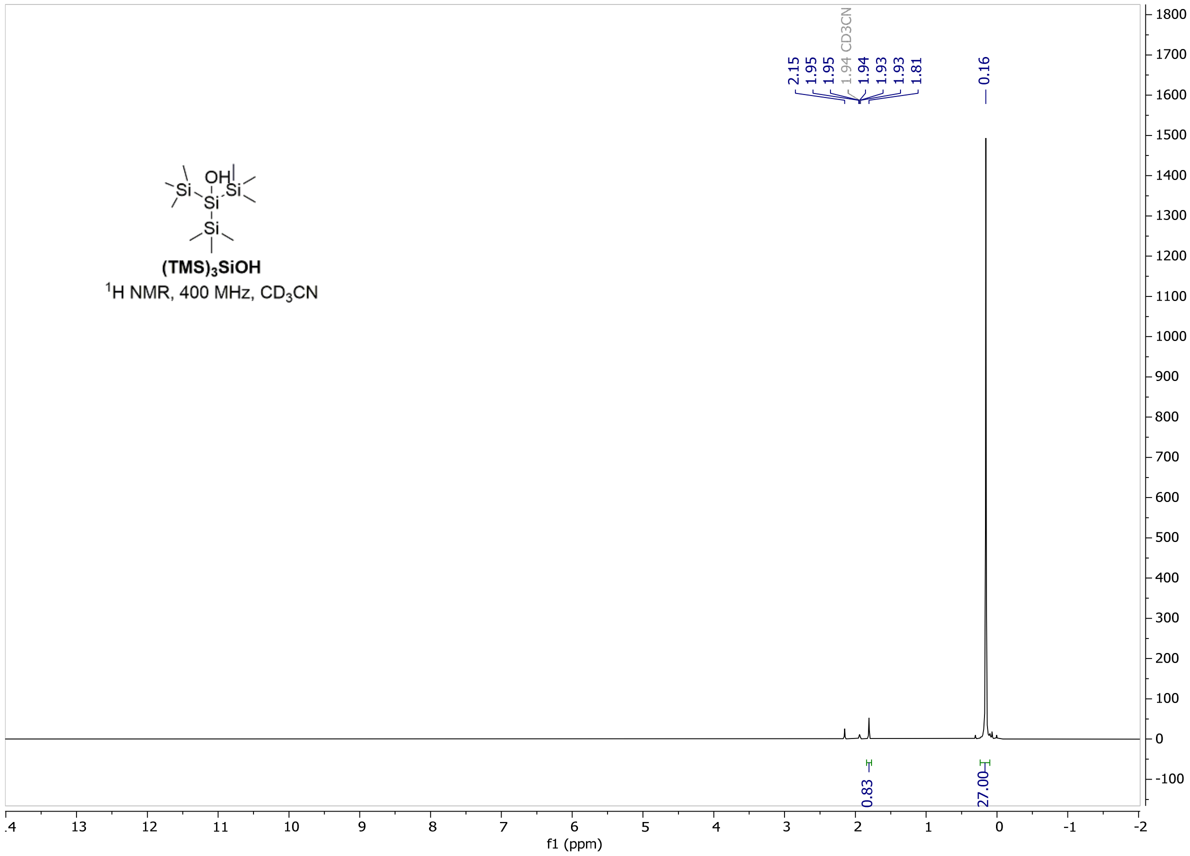

2.15
1.95
1.95
1.94 CD3CN
1.94
1.93
1.93
1.81
0.16
OH
Si
Si
Si
Si
(TMS)3SiOH
1H NMR, 400 MHz, CD3CN
0.83
27.00
f1 (ppm)

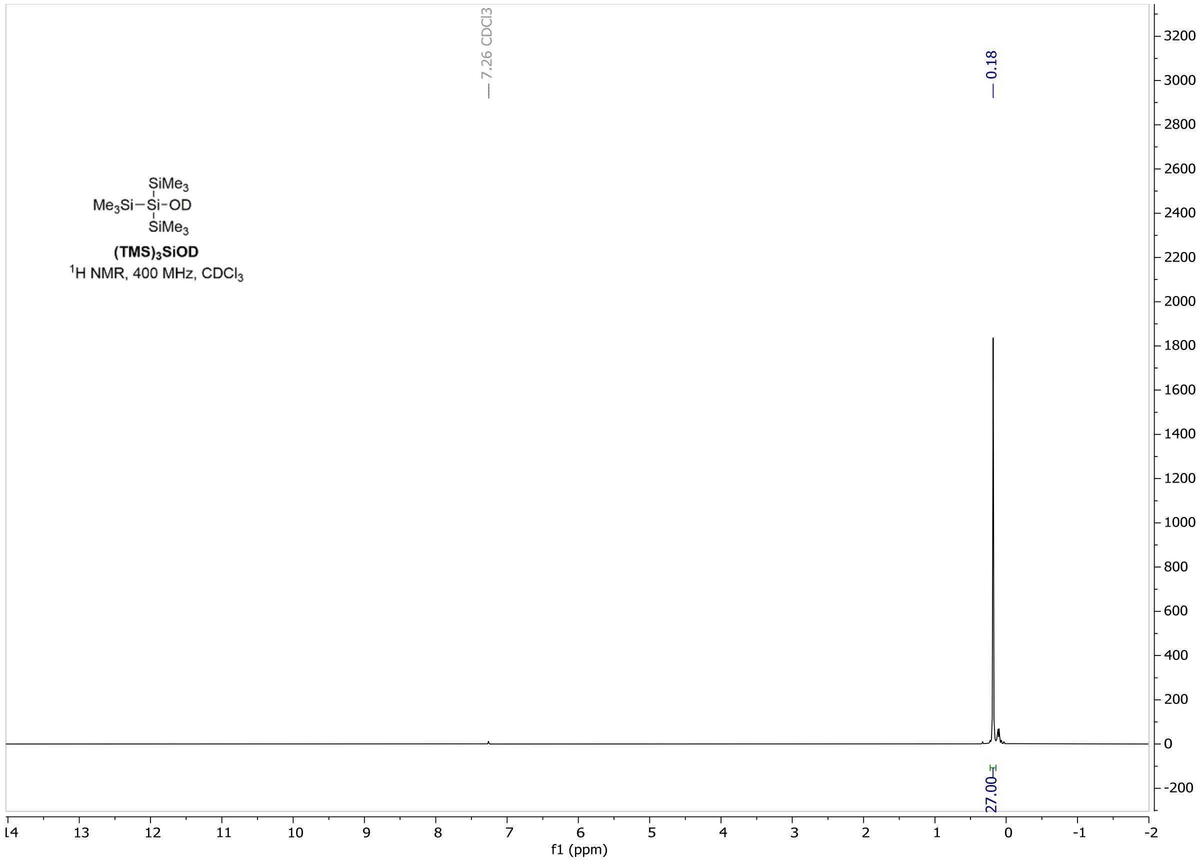
SiMe3
Me3Si–Si–OD
SiMe3
(TMS)3SiOD
1H NMR, 400 MHz, CDCl3
7.26 CDCl3
0.18
27.00
f1 (ppm)

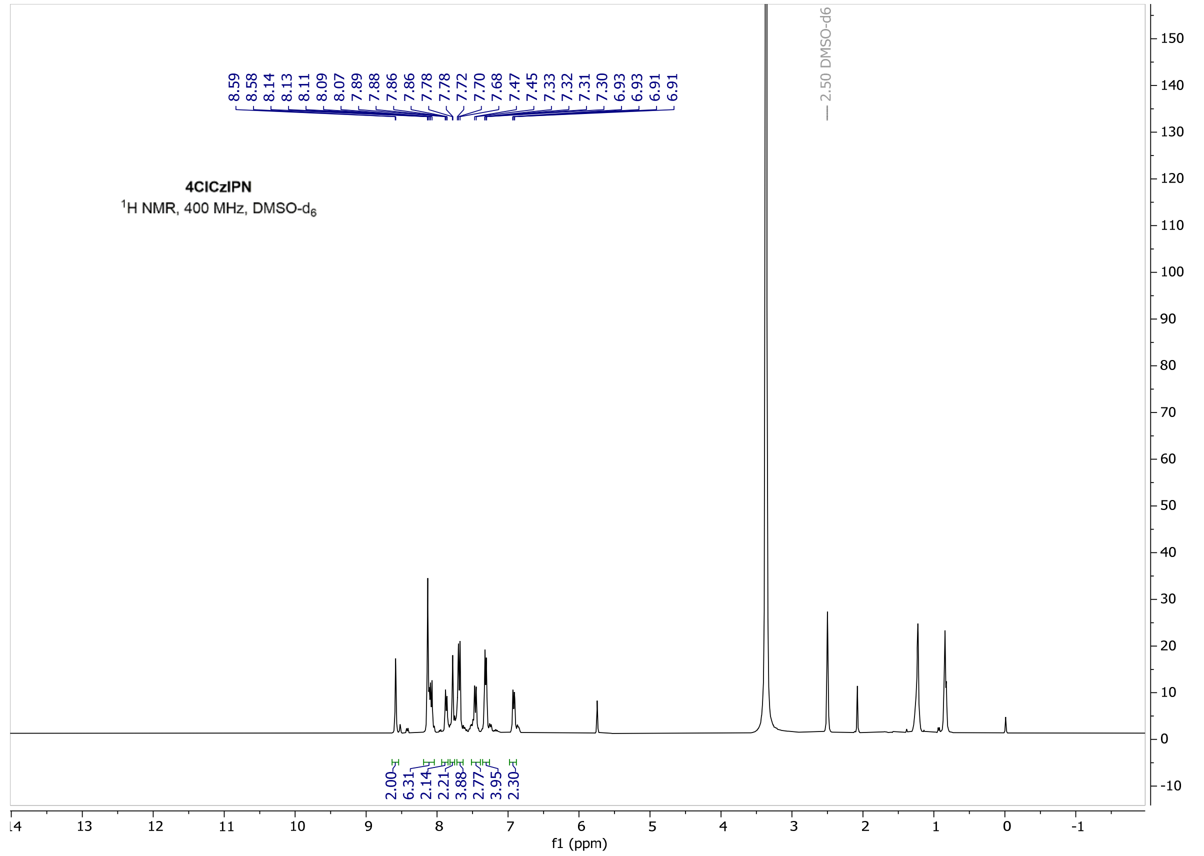
4ClCzIPN
1H NMR, 400 MHz, DMSO-d6
8.59
8.58
8.14
8.13
8.11
8.09
8.07
7.89
7.88
7.86
7.78
7.78
7.72
7.70
7.68
7.47
7.45
7.33
7.32
7.31
7.30
6.93
6.93
6.91
6.91
2.50 DMSO-d6
2.00
6.31
2.14
2.21
3.88
2.77
3.95
2.30
f1 (ppm)

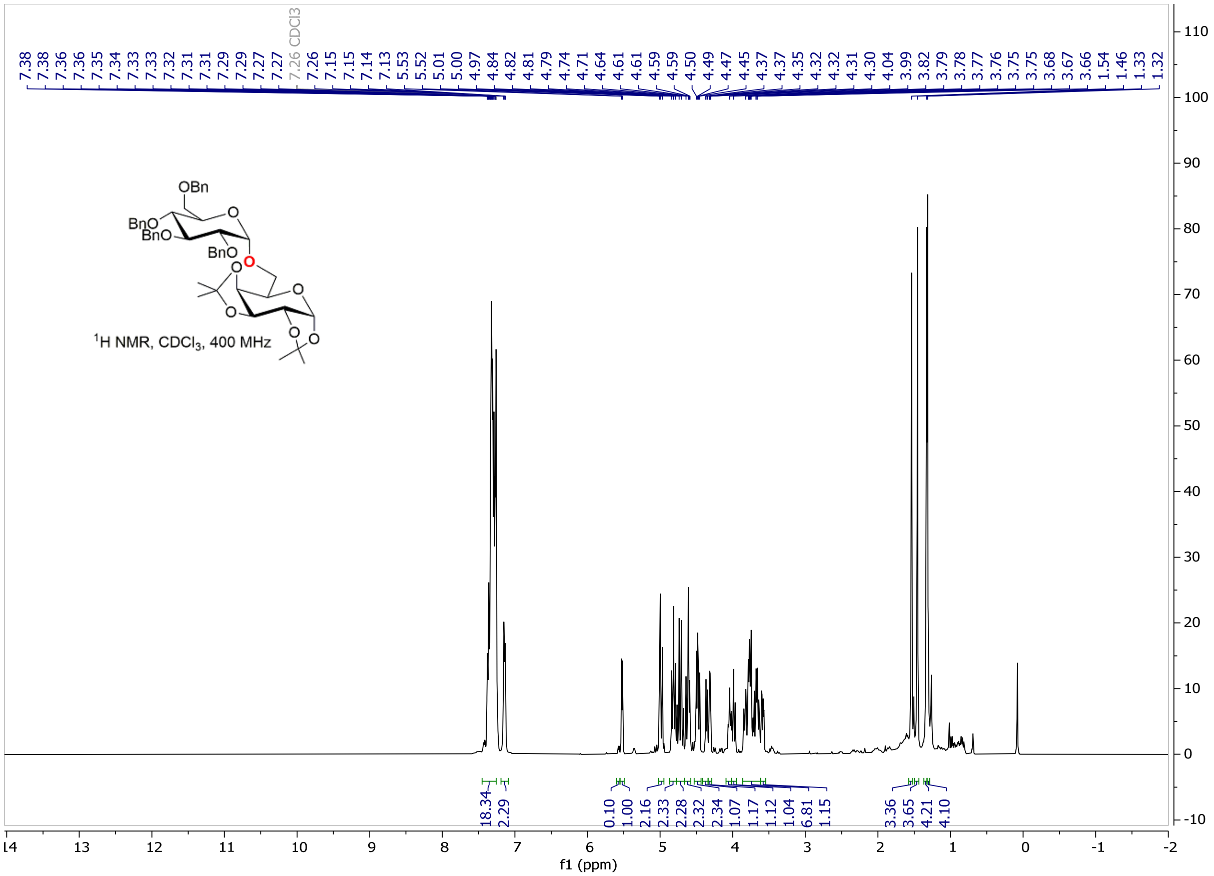

OBn
BnO
BnO
BnO
O
O
O
O
O
O
O
O
^{1}H NMR, CDCl3, 400 MHz
7.26 CDCl3
f1 (ppm)

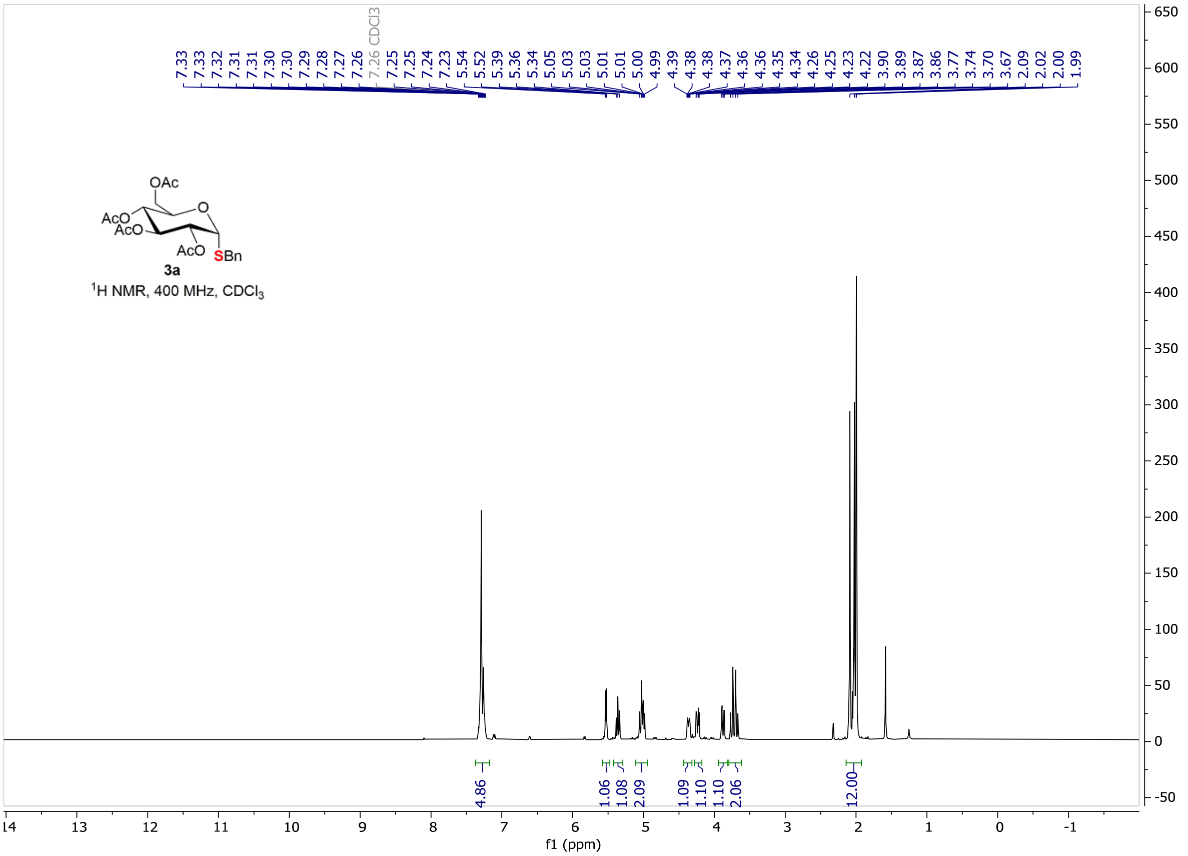

7.33 7.33 7.32 7.31 7.31 7.30 7.30 7.29 7.28 7.27 7.26 7.26 CDCl3 7.25 7.25 7.24 7.23 5.54 5.52 5.39 5.36 5.34 5.05 5.03 5.03 5.01 5.01 5.00 4.99 4.39 4.38 4.38 4.37 4.36 4.36 4.35 4.34 4.26 4.25 4.23 4.22 3.90 3.89 3.87 3.86 3.77 3.74 3.70 3.67 2.09 2.02 2.00 1.99
OAc
AcO
AcO
O
AcO
SBn
3a
^{1}H NMR, 400 MHz, CDCl$_3$
4.86
1.06
1.08
2.09
1.09
1.10
1.10
2.06
12.00
f1 (ppm)

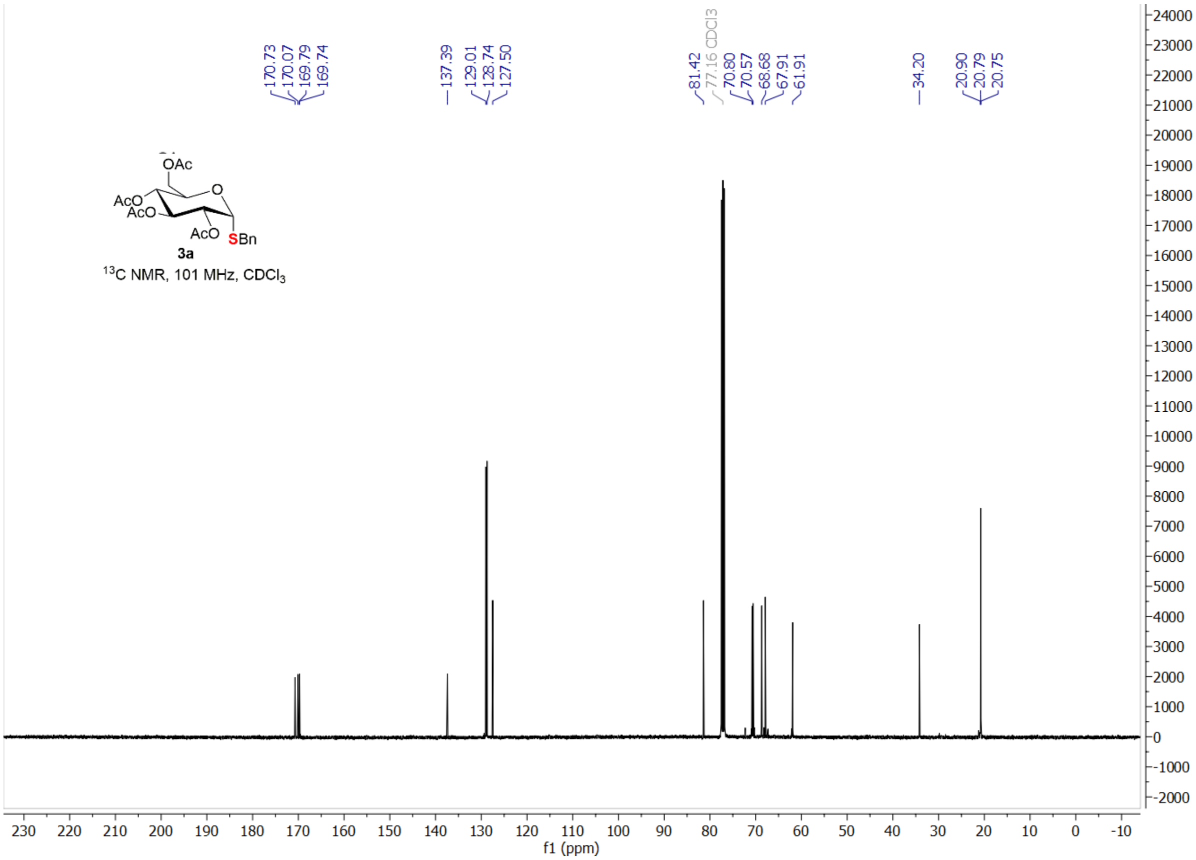

170.73 170.07 169.79 169.74
137.39
129.01 128.74 127.50
81.42
77.16 CDCl3
70.80 70.57 68.68 67.91 61.91
34.20
20.90 20.79 20.75
OAc
AcO
AcO
O
AcO
SBn
3a
^{13}C NMR, 101 MHz, CDCl$_3$
f1 (ppm)

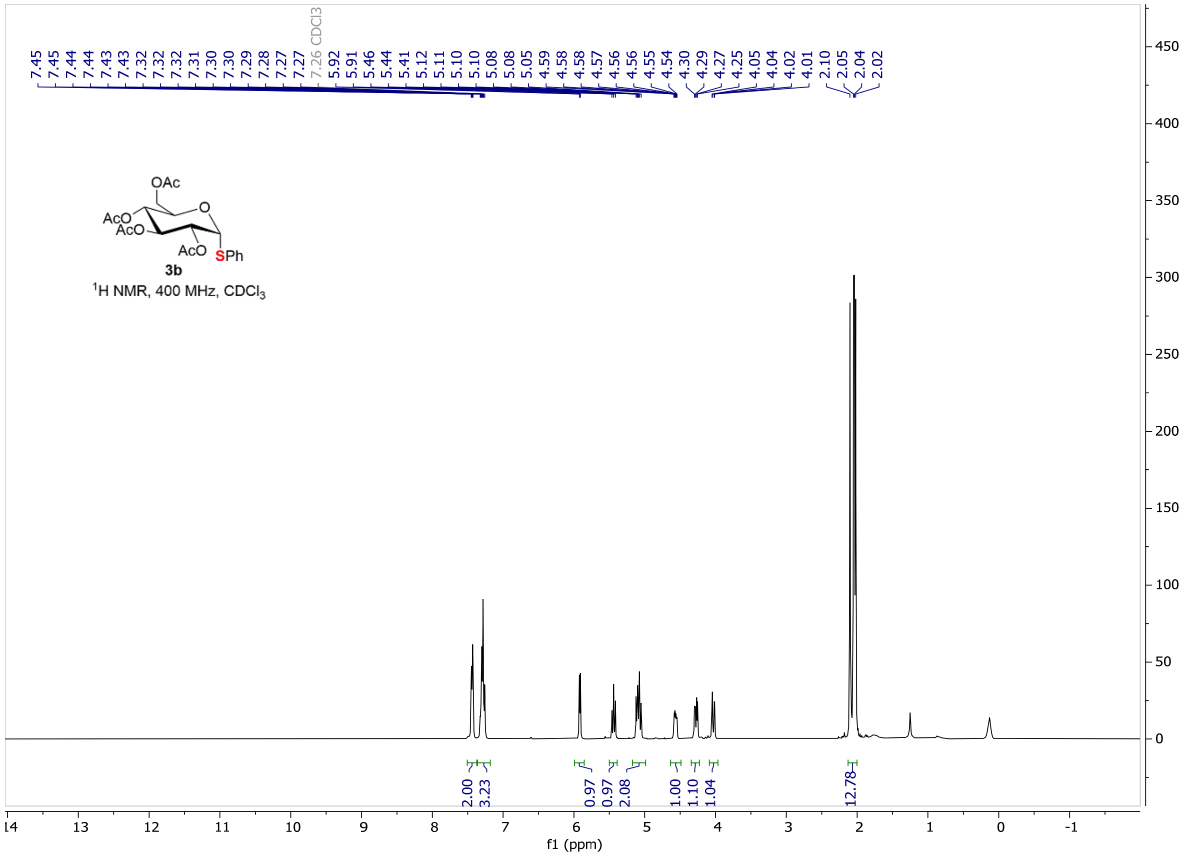
7.45 7.45 7.44 7.44 7.43 7.43 7.32 7.32 7.32 7.31 7.30 7.30 7.29 7.28 7.27 7.27 7.26 CDCl3 5.92 5.91 5.46 5.44 5.41 5.12 5.11 5.10 5.10 5.08 5.08 5.05 4.59 4.58 4.58 4.57 4.56 4.56 4.55 4.54 4.30 4.29 4.27 4.25 4.05 4.04 4.02 4.01 2.10 2.05 2.04 2.02
OAc
AcO
AcO
O
AcO
SPh
3b
^{1}H NMR, 400 MHz, CDCl$_3$
2.00 3.23 0.97 0.97 2.08 1.00 1.10 1.04 12.78
f1 (ppm)

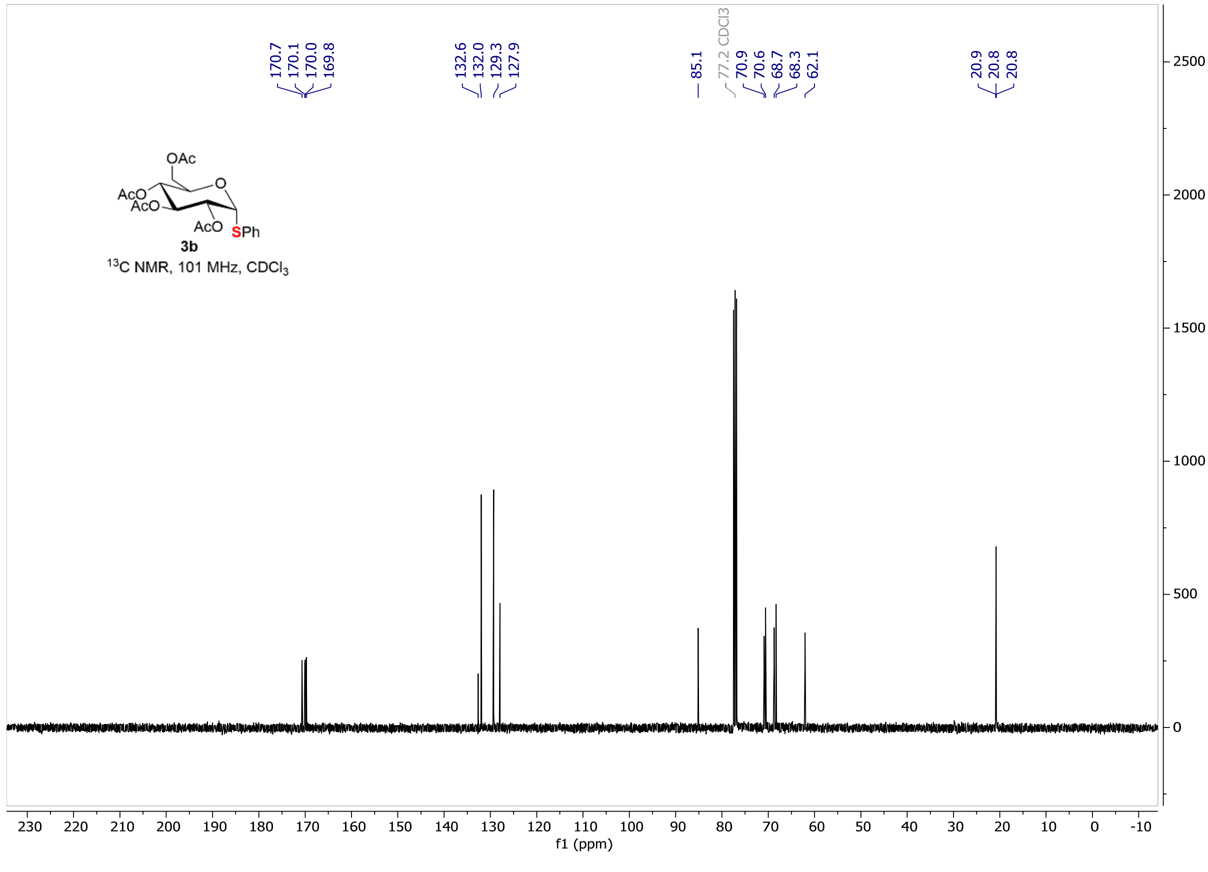
170.7 170.1 170.0 169.8 132.6 132.0 129.3 127.9 85.1 77.2 CDCl3 70.9 70.6 68.7 68.3 62.1 20.9 20.8 20.8
OAc
AcO
AcO
O
AcO
SPh
3b
^{13}C NMR, 101 MHz, CDCl$_3$
f1 (ppm)

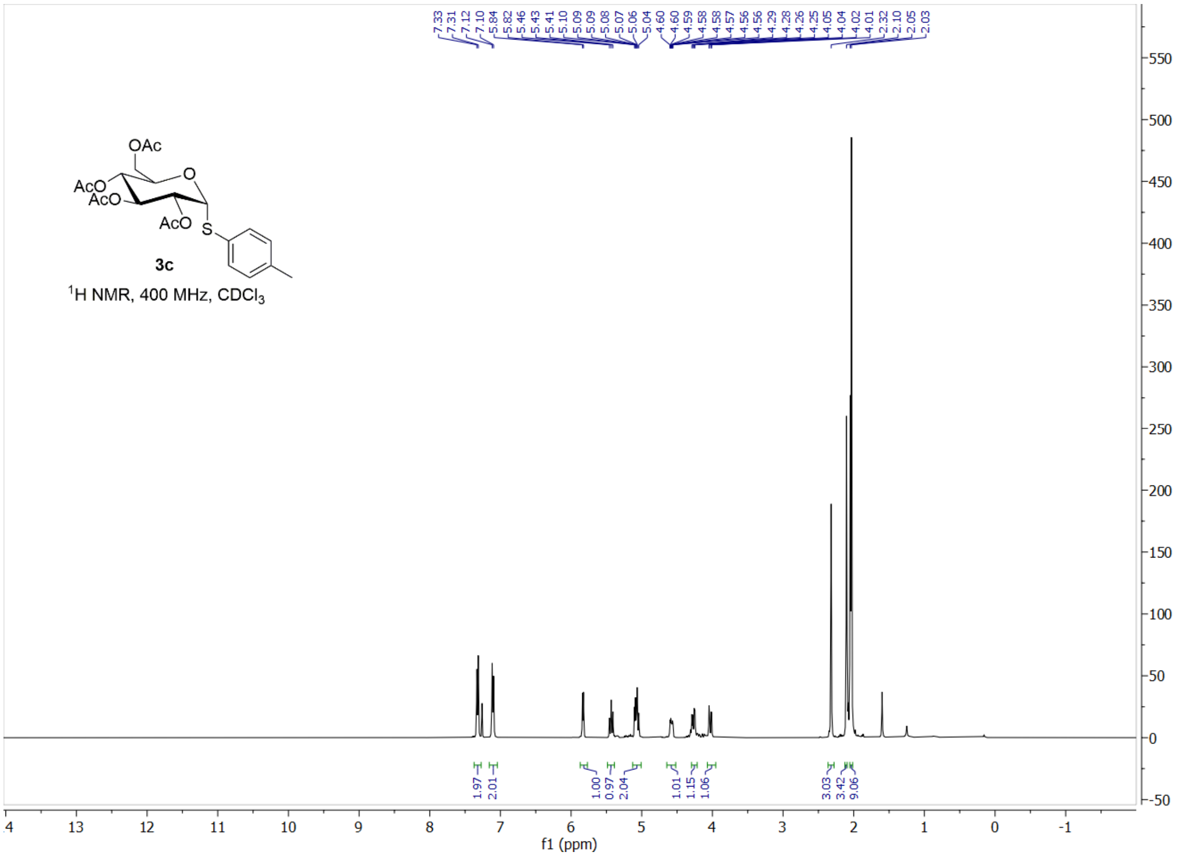
7.33
7.31
7.12
7.10
5.84
5.82
5.46
5.43
5.41
5.10
5.09
5.09
5.08
5.07
5.06
5.04
4.60
4.59
4.58
4.58
4.57
4.56
4.56
4.29
4.28
4.26
4.25
4.05
4.04
4.02
4.01
2.32
2.10
2.05
2.03
OAc
AcO
AcO
O
AcO
S
3c
^{1}H NMR, 400 MHz, CDCl3
1.97
2.01
1.00
0.97
2.04
1.01
1.15
1.06
3.03
3.42
9.06
f1 (ppm)

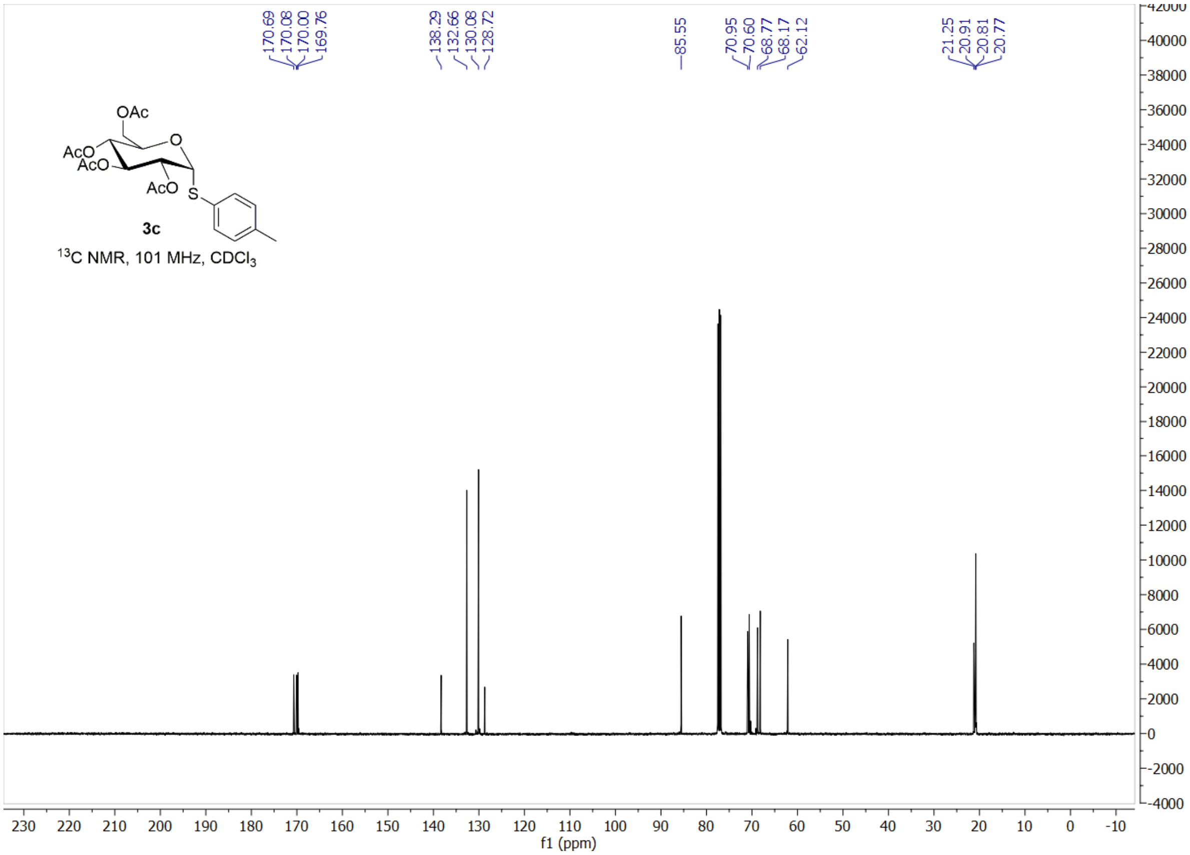
170.69
170.08
170.00
169.76
138.29
132.66
130.08
128.72
85.55
70.95
70.60
68.77
68.17
62.12
21.25
20.91
20.81
20.77
OAc
AcO
AcO
O
AcO
S
3c
^{13}C NMR, 101 MHz, CDCl3
f1 (ppm)

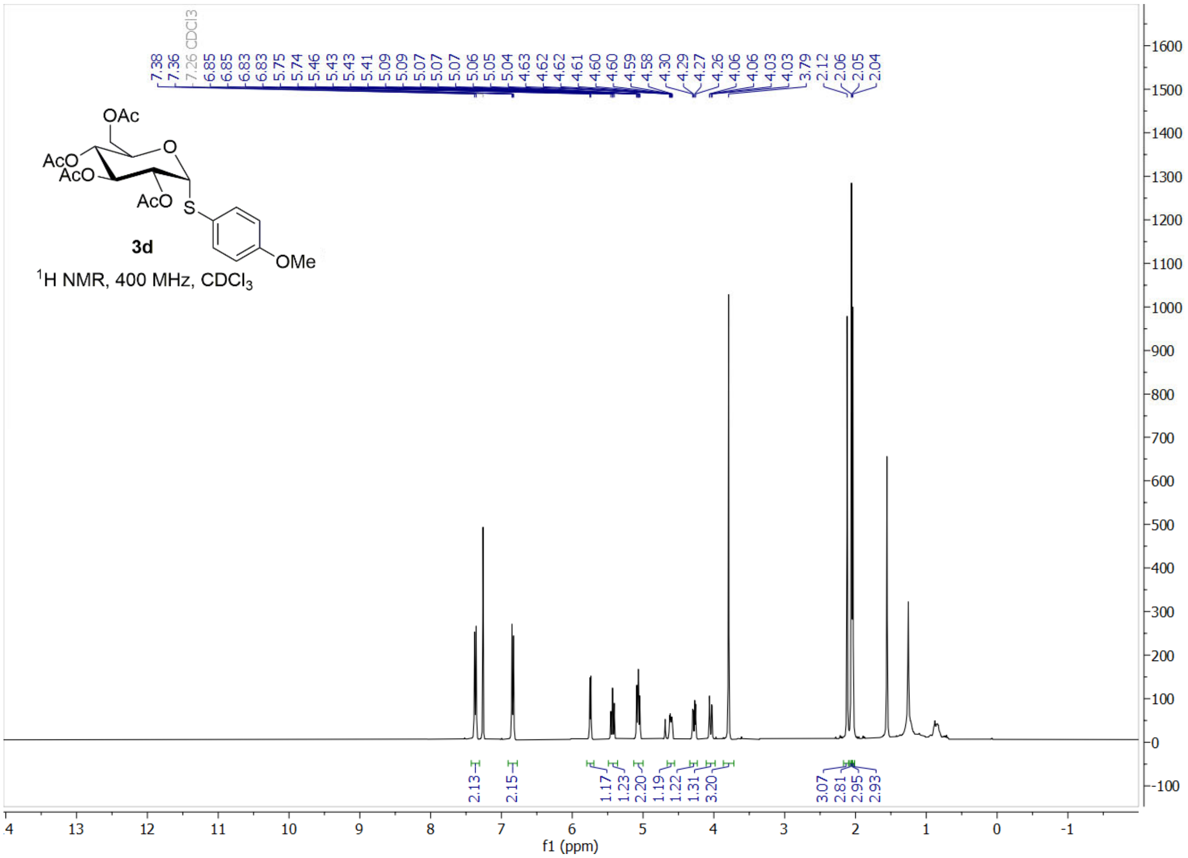

7.38
7.36
7.26 CDCl3
6.85
6.85
6.83
6.83
5.75
5.74
5.46
5.43
5.43
5.41
5.09
5.09
5.07
5.07
5.07
5.06
5.05
5.04
4.63
4.62
4.62
4.61
4.60
4.60
4.59
4.58
4.30
4.29
4.27
4.26
4.06
4.06
4.03
4.03
3.79
2.12
2.06
2.05
2.04
OAc
AcO
AcO
AcO
O
S
OMe
3d
^{1}H NMR, 400 MHz, $CDCl_3$
2.13
2.15
1.17
1.23
2.20
1.19
1.22
1.31
3.20
3.07
2.81
2.95
2.93
f1 (ppm)

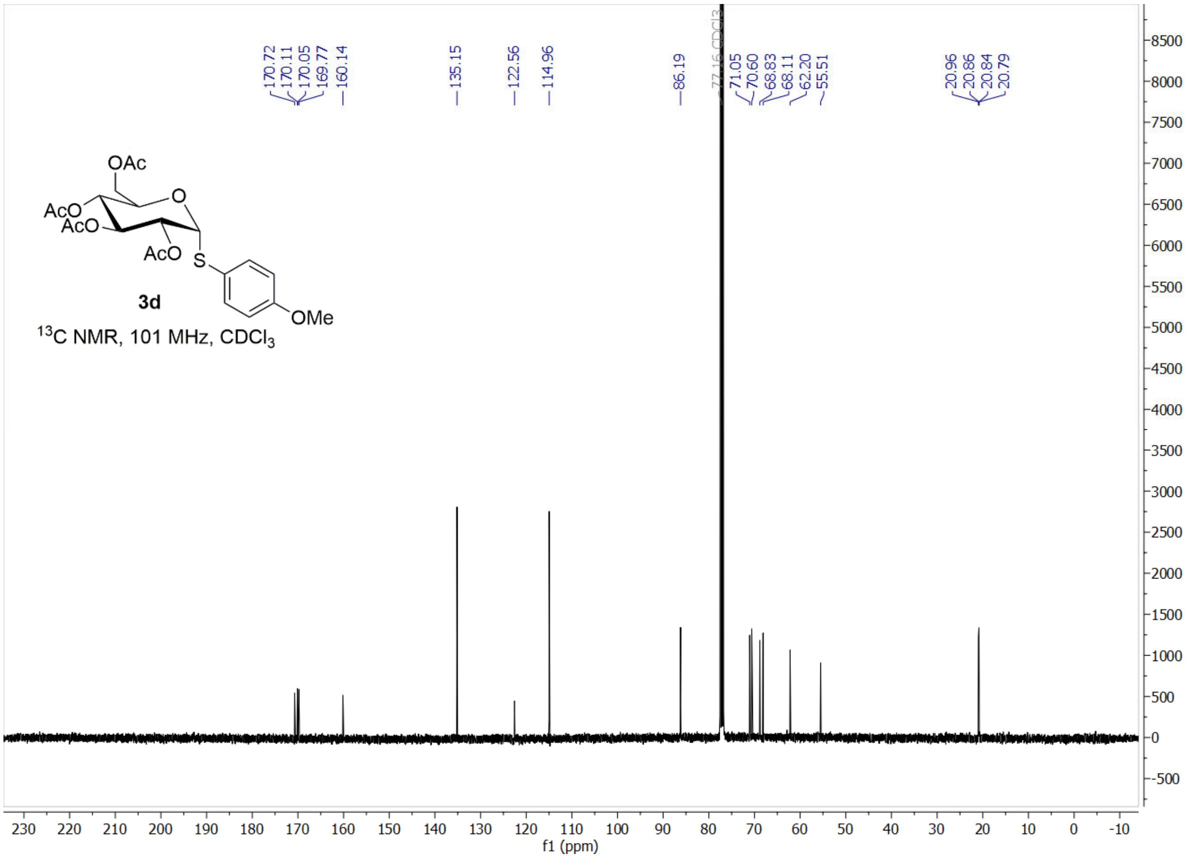

170.72
170.11
170.05
169.77
160.14
135.15
122.56
114.96
86.19
77.16 CDCl3
71.05
70.60
68.83
68.11
62.20
55.51
20.96
20.86
20.84
20.79
OAc
AcO
AcO
AcO
O
S
OMe
3d
^{13}C NMR, 101 MHz, $CDCl_3$
f1 (ppm)

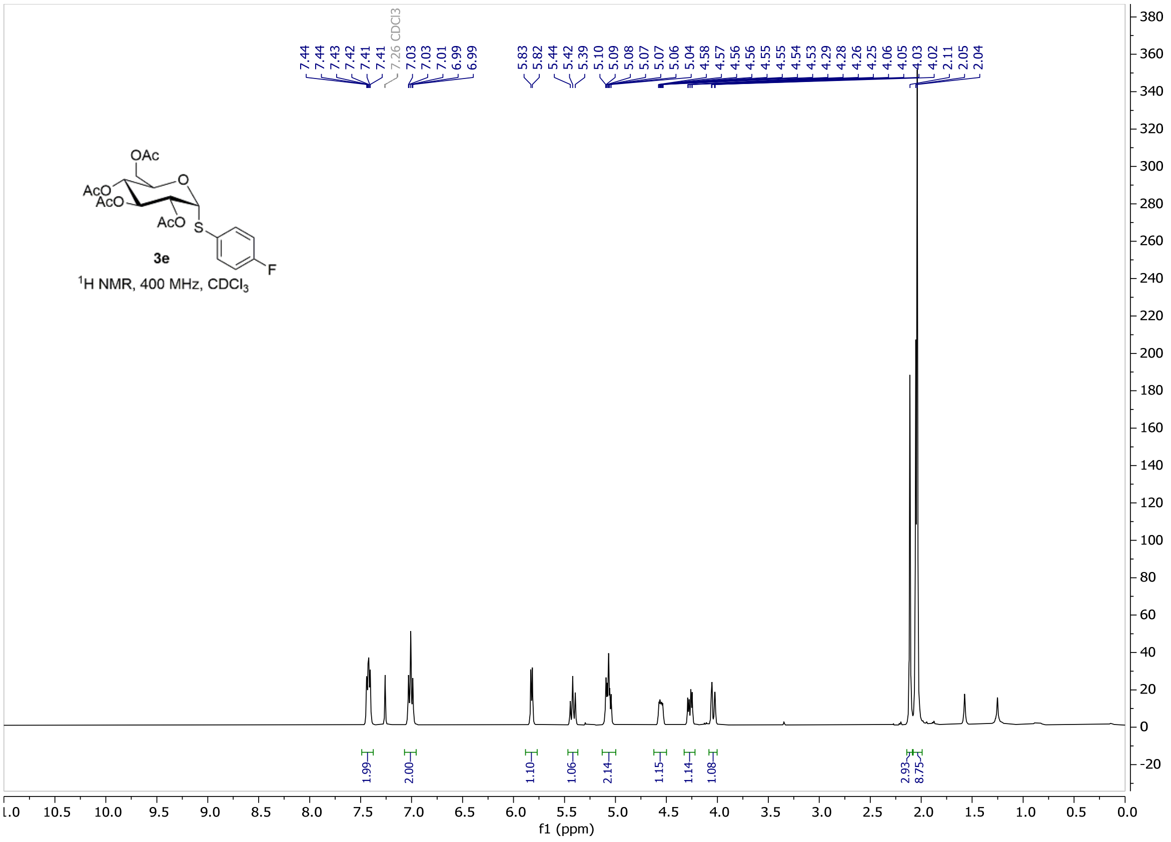
OAc
AcO
AcO
O
AcO
S
F
3e
1H NMR, 400 MHz, CDCl3
7.44 7.44 7.43 7.42 7.41 7.41 7.26 CDCl3 7.03 7.03 7.01 6.99 6.99 5.83 5.82 5.44 5.42 5.39 5.10 5.09 5.08 5.07 5.07 5.06 5.04 4.58 4.57 4.56 4.56 4.55 4.55 4.54 4.53 4.29 4.28 4.26 4.25 4.06 4.05 4.03 4.02 2.11 2.05 2.04
1.99 2.00 1.10 1.06 2.14 1.15 1.14 1.08 2.93 8.75
f1 (ppm)

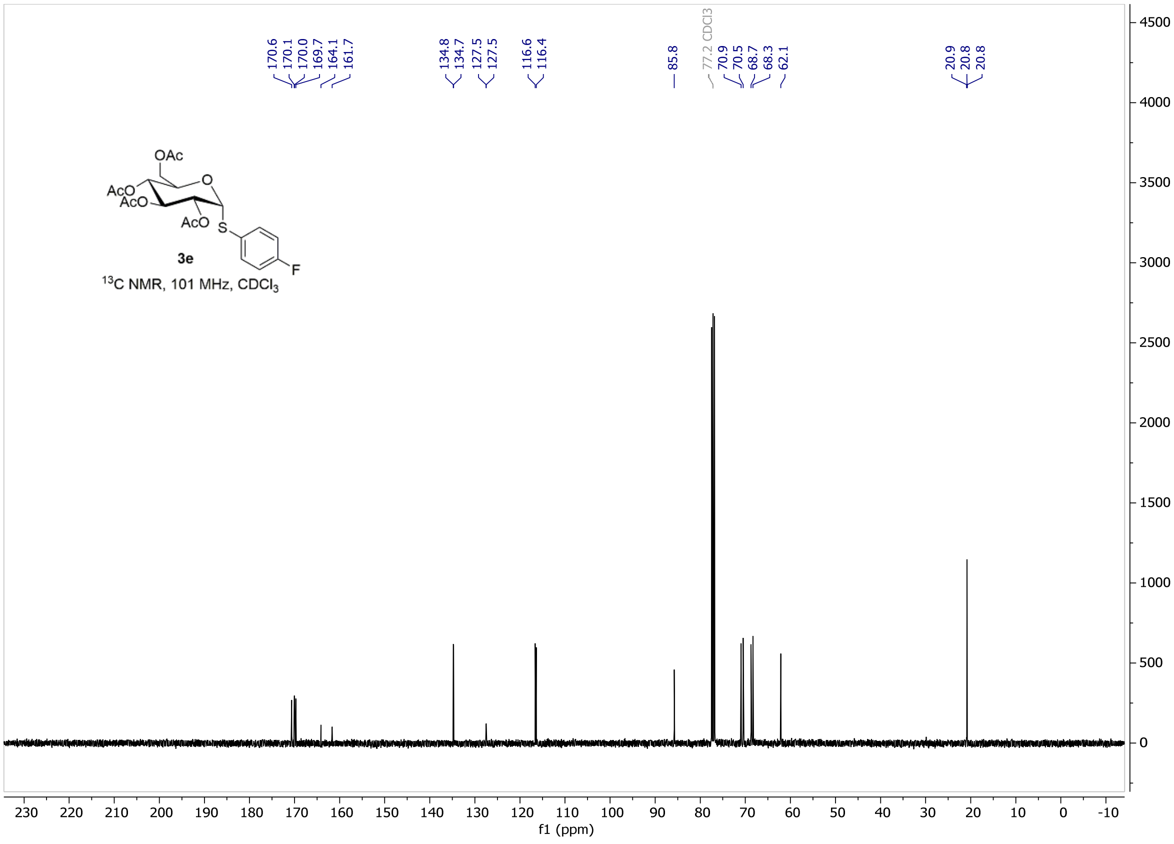
OAc
AcO
AcO
O
AcO
S
F
3e
13C NMR, 101 MHz, CDCl3
170.6 170.1 170.0 169.7 164.1 161.7 134.8 134.7 127.5 127.5 116.6 116.4 85.8 77.2 CDCl3 70.9 70.5 68.7 68.3 62.1 20.9 20.8 20.8
f1 (ppm)

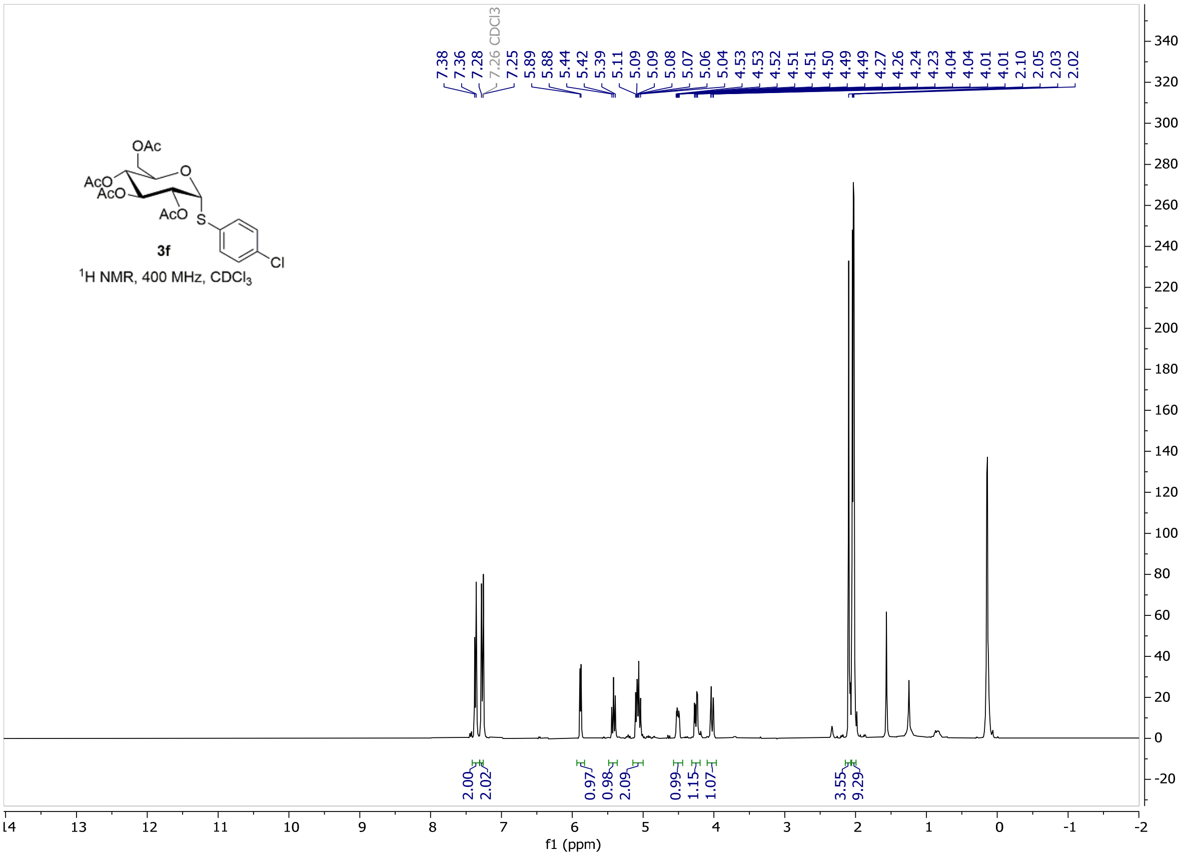
7.38
7.36
7.28
7.26 CDCl3
7.25
5.89
5.88
5.44
5.42
5.39
5.11
5.09
5.09
5.08
5.07
5.06
5.04
4.53
4.53
4.52
4.51
4.51
4.50
4.49
4.49
4.27
4.26
4.24
4.23
4.04
4.04
4.01
2.10
2.05
2.03
2.02
OAc
AcO
AcO
O
AcO
S
Cl
3f
1H NMR, 400 MHz, CDCl3
2.00
2.02
0.97
0.98
2.09
0.99
1.15
1.07
3.55
9.29
f1 (ppm)

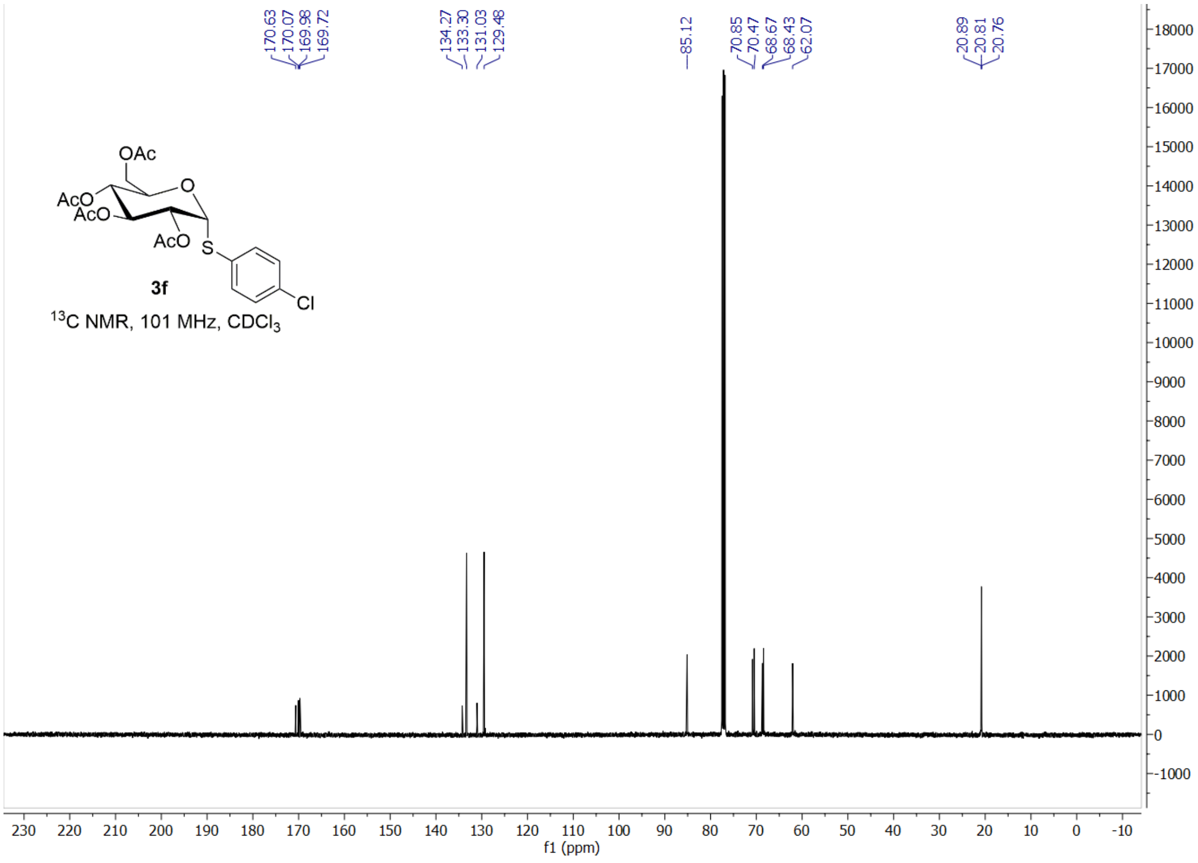
170.63
170.07
169.98
169.72
134.27
133.30
131.03
129.48
85.12
70.85
70.47
68.67
68.43
62.07
20.89
20.81
20.76
OAc
AcO
AcO
O
AcO
S
Cl
3f
13C NMR, 101 MHz, CDCl3
f1 (ppm)

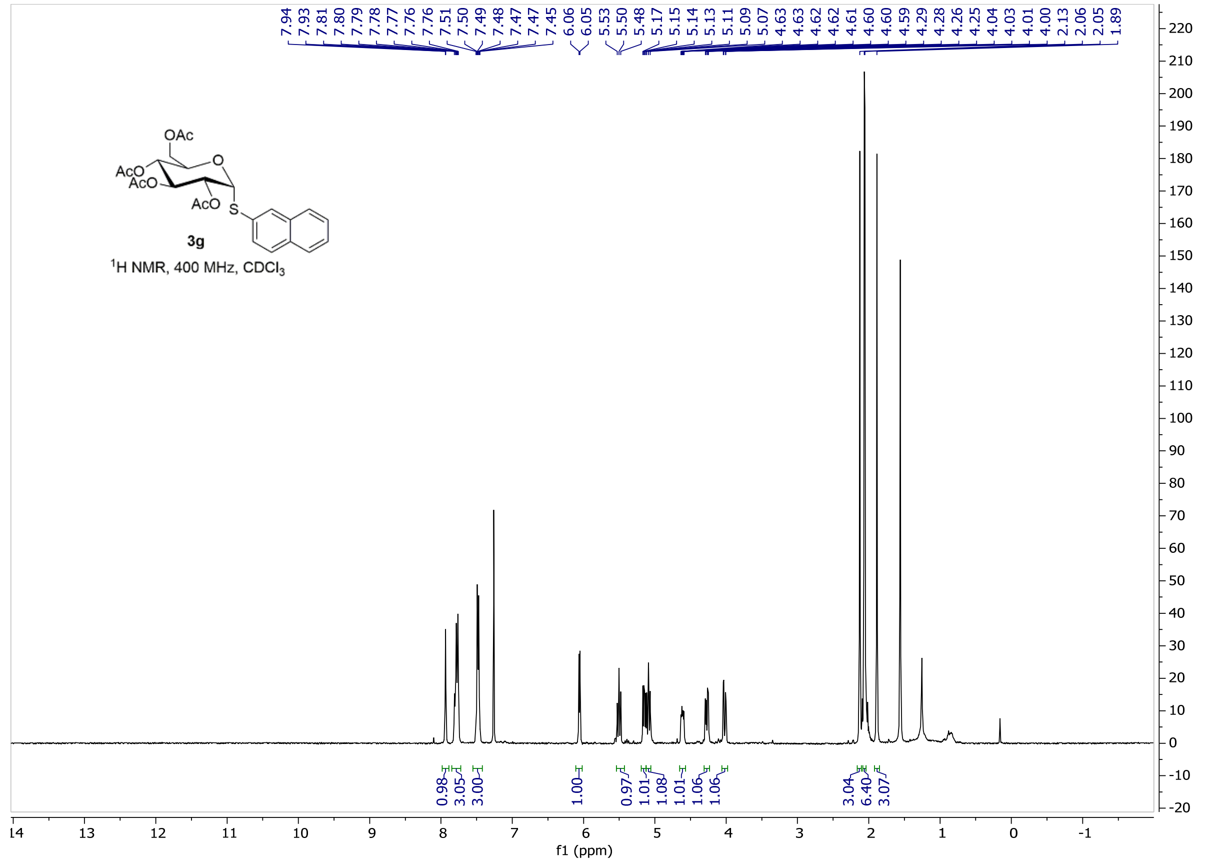
3g
1H NMR, 400 MHz, CDCl3
f1 (ppm)

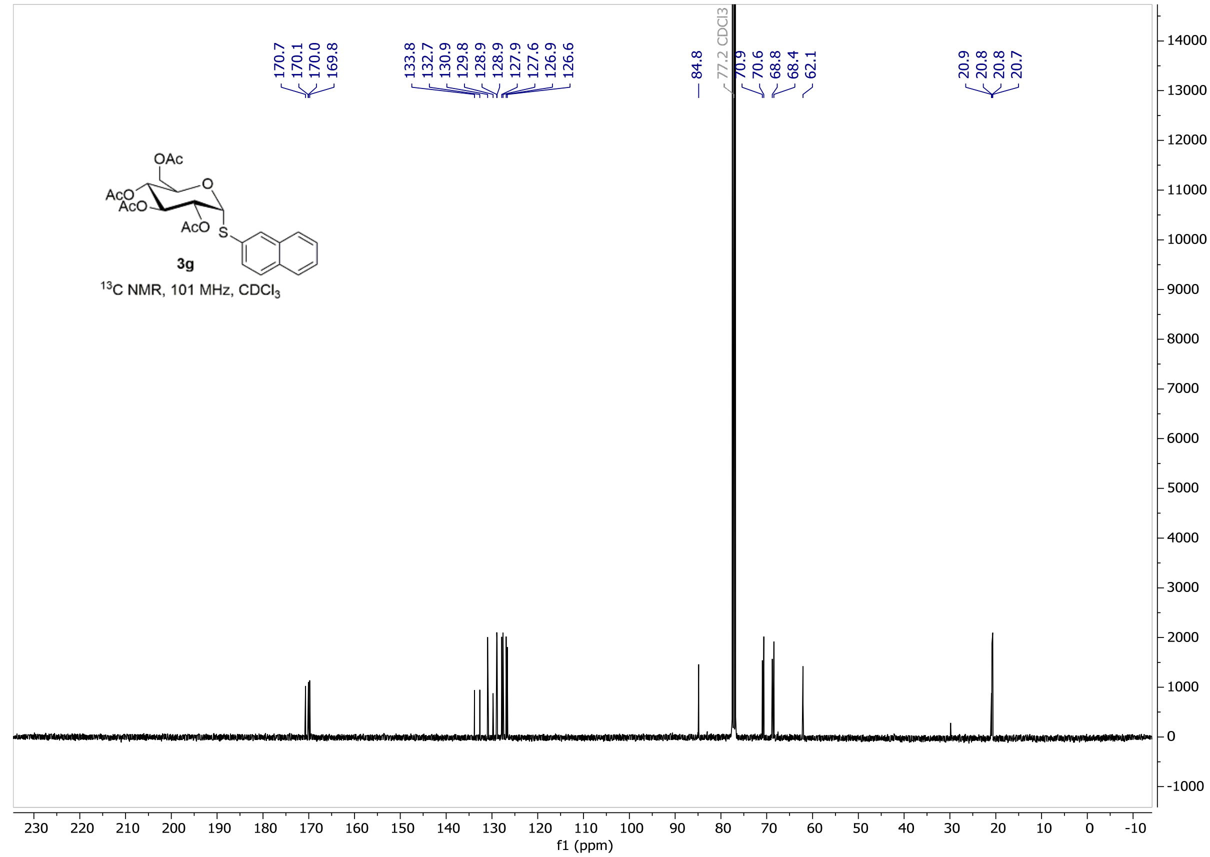
3g
13C NMR, 101 MHz, CDCl3
77.2 CDCl3
f1 (ppm)

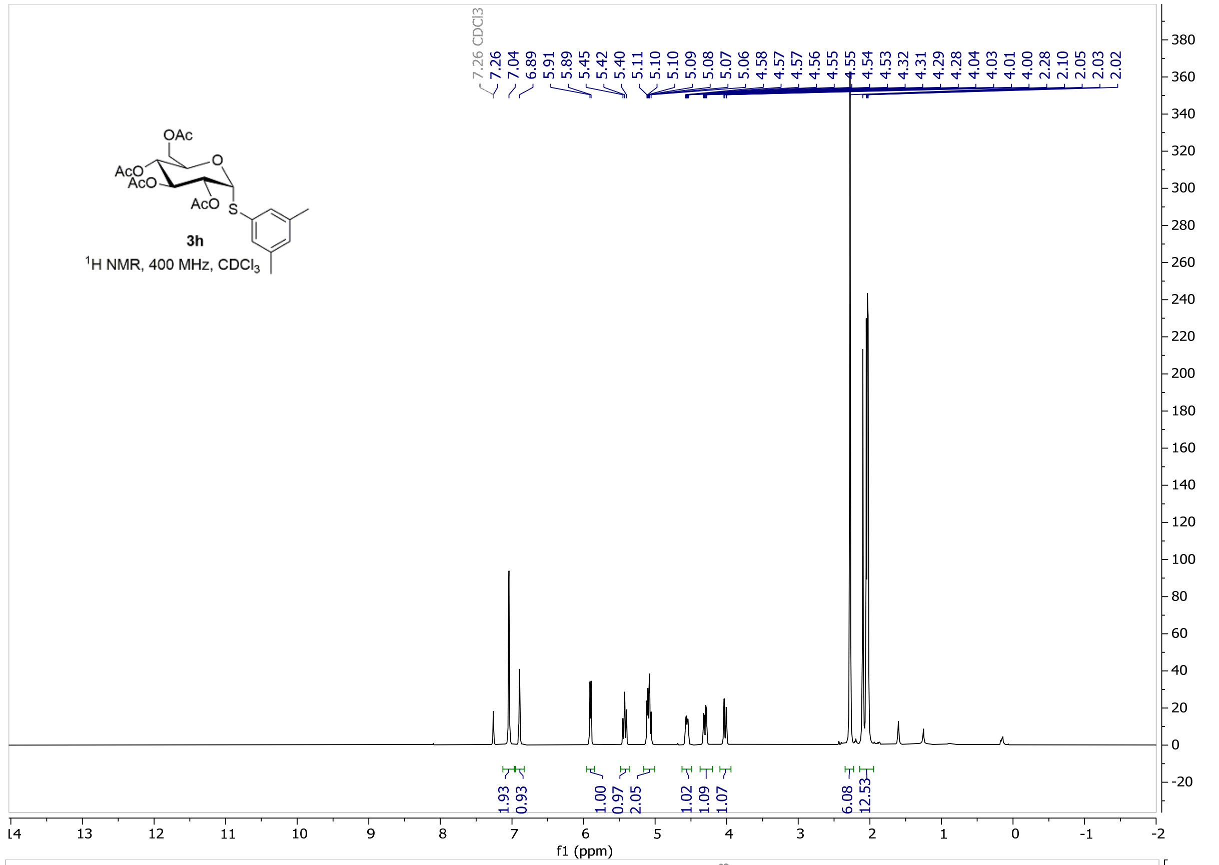

7.26 CDCl3
7.26
7.04
6.89
5.91
5.89
5.45
5.42
5.40
5.11
5.10
5.10
5.09
5.08
5.07
5.06
4.58
4.57
4.57
4.56
4.55
4.55
4.54
4.53
4.32
4.31
4.29
4.28
4.04
4.03
4.01
4.00
2.28
2.10
2.05
2.03
2.02
OAc
AcO
AcO
AcO
O
S
3h
1H NMR, 400 MHz, CDCl3
1.93
0.93
1.00
0.97
2.05
1.02
1.09
1.07
6.08
12.53
f1 (ppm)

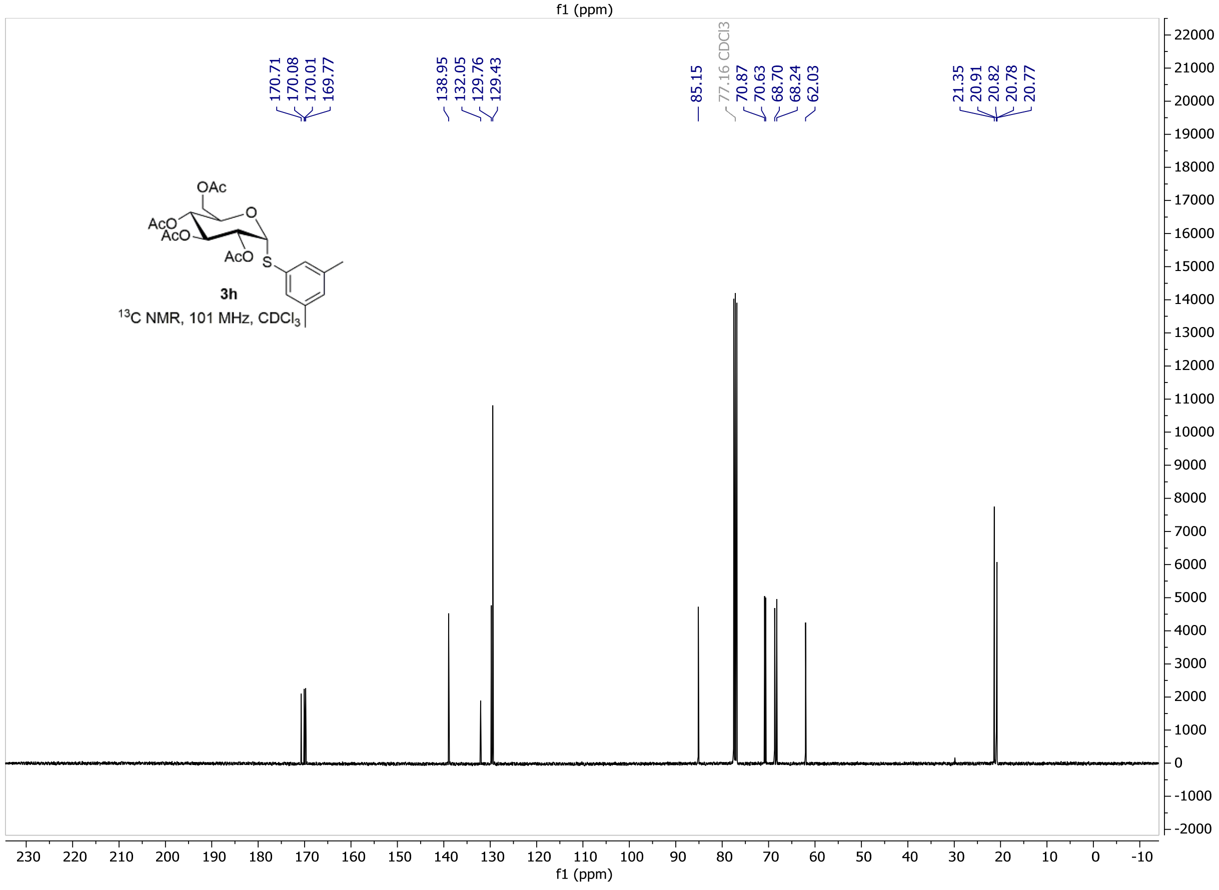

170.71
170.08
170.01
169.77
138.95
132.05
129.76
129.43
85.15
77.16 CDCl3
70.87
70.63
68.70
68.24
62.03
21.35
20.91
20.82
20.78
20.77
OAc
AcO
AcO
AcO
O
S
3h
13C NMR, 101 MHz, CDCl3
f1 (ppm)

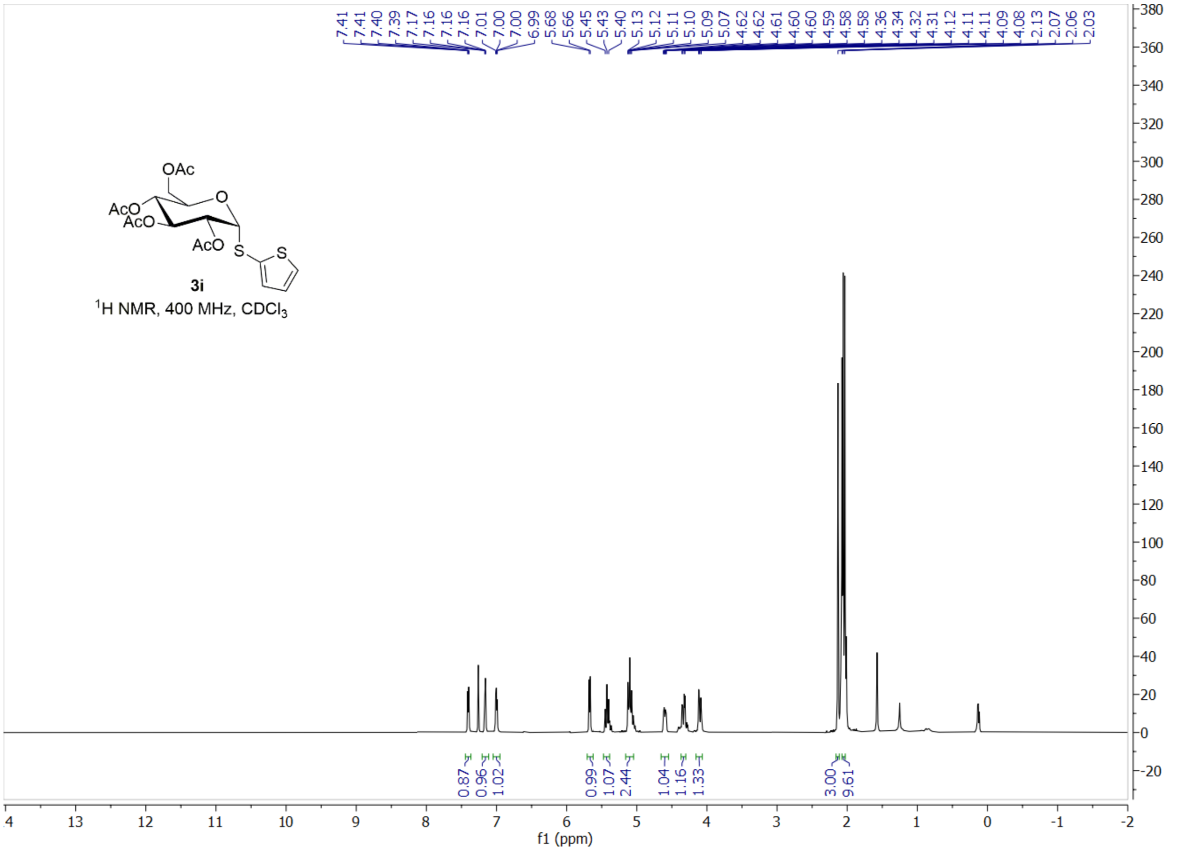
7.41
7.41
7.40
7.39
7.17
7.16
7.16
7.01
7.00
7.00
6.99
5.68
5.66
5.45
5.43
5.40
5.13
5.12
5.11
5.10
5.09
5.07
4.62
4.62
4.61
4.60
4.60
4.59
4.58
4.58
4.36
4.34
4.32
4.31
4.12
4.11
4.11
4.09
4.08
2.13
2.07
2.06
2.03
OAc
AcO
AcO
O
AcO
S
S
3i
^{1}H NMR, 400 MHz, CDCl$_3$
0.87
0.96
1.02
0.99
1.07
2.44
1.04
1.16
1.33
3.00
9.61
f1 (ppm)

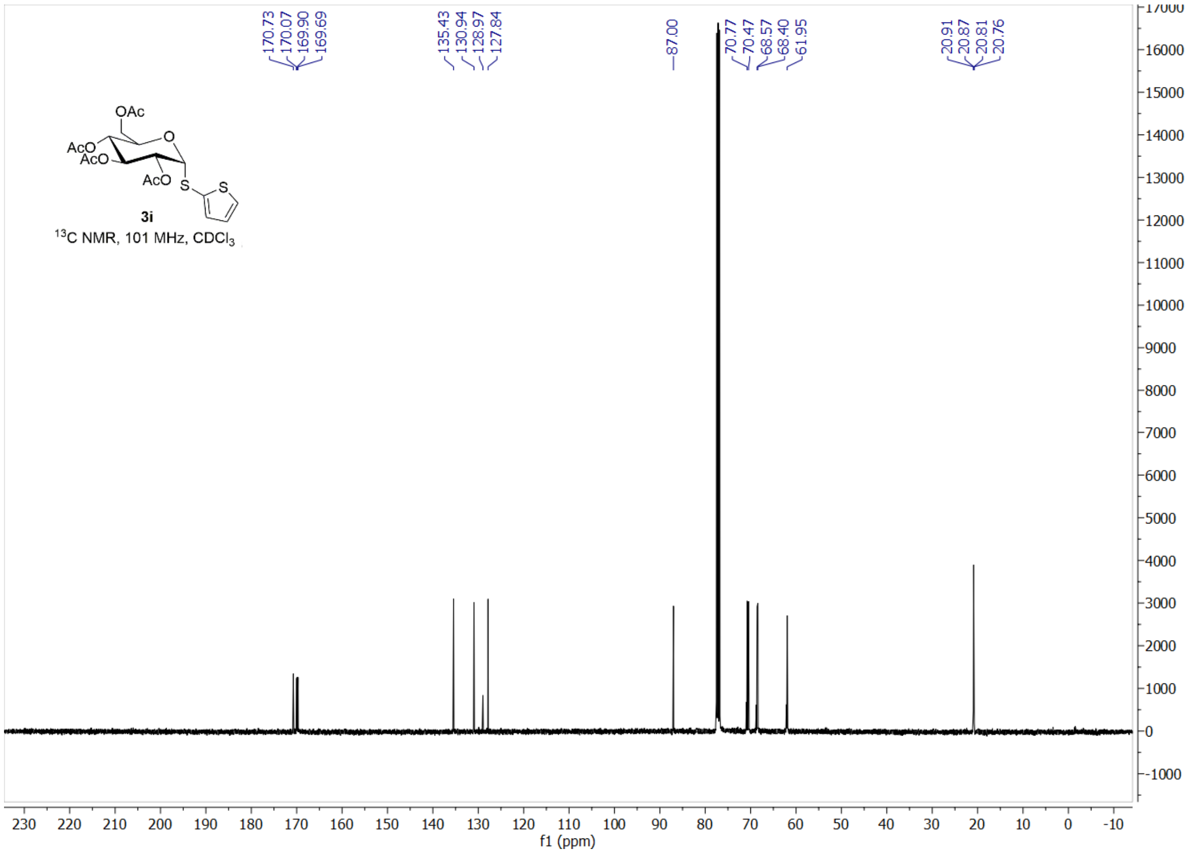
170.73
170.07
169.90
169.69
135.43
130.94
128.97
127.84
87.00
70.77
70.47
68.57
68.40
61.95
20.91
20.87
20.81
20.76
OAc
AcO
AcO
O
AcO
S
S
3i
^{13}C NMR, 101 MHz, CDCl$_3$
f1 (ppm)

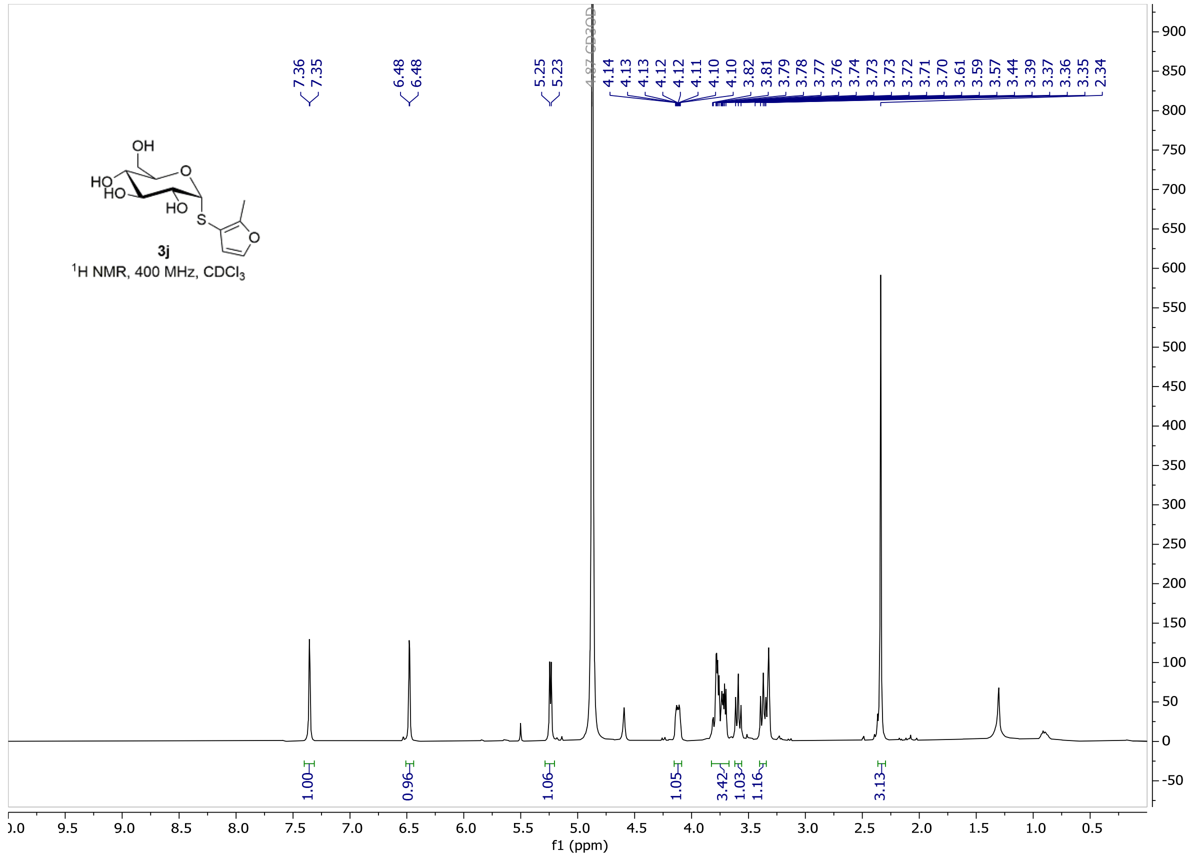
3j
^{1}H NMR, 400 MHz, CDCl$_3$
7.36 7.35 6.48 6.48 5.25 5.23 4.87 CD3OD 4.14 4.13 4.13 4.12 4.12 4.11 4.10 4.10 3.82 3.81 3.79 3.78 3.77 3.76 3.74 3.73 3.73 3.72 3.71 3.70 3.61 3.59 3.57 3.44 3.39 3.37 3.36 3.35 2.34
1.00 0.96 1.06 1.05 3.42 1.03 1.16 3.13
f1 (ppm)

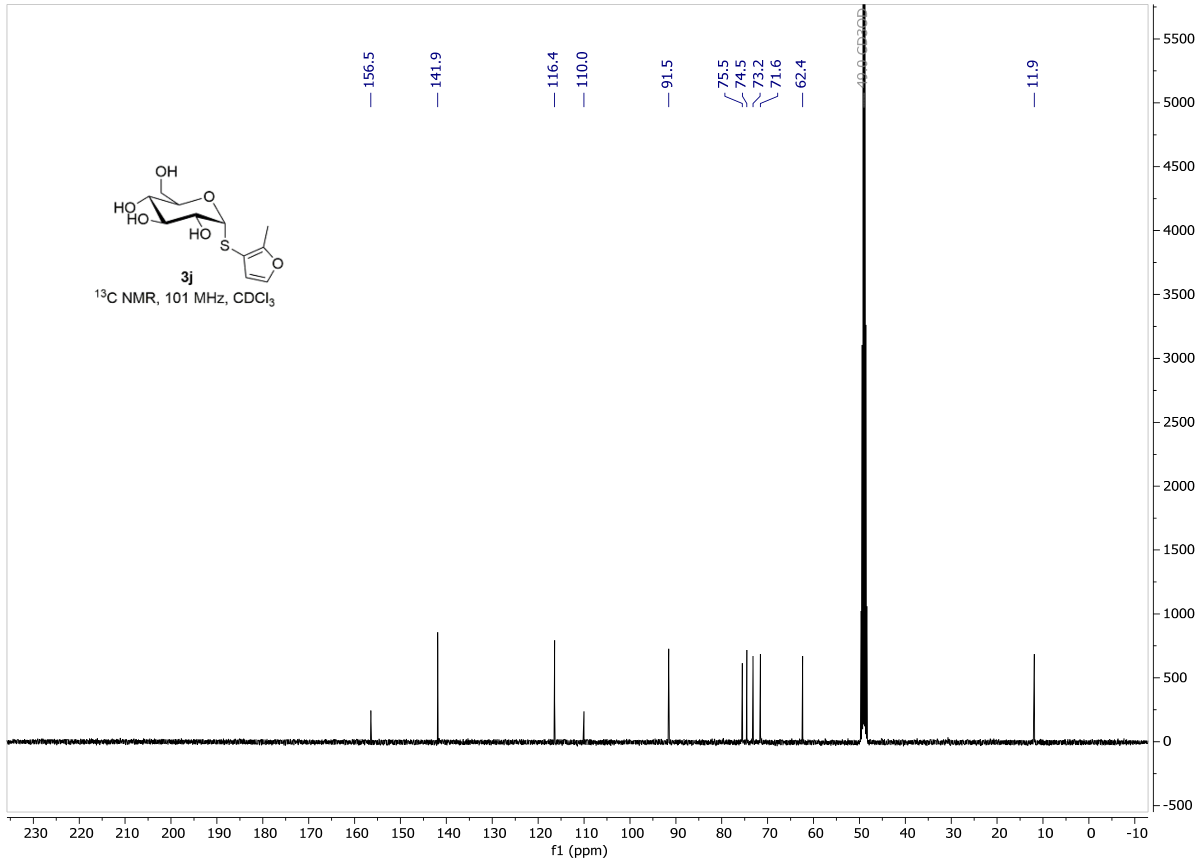
3j
^{13}C NMR, 101 MHz, CDCl$_3$
156.5 141.9 116.4 110.0 91.5 75.5 74.5 73.2 71.6 62.4 49.0 CD3OD 11.9
f1 (ppm)

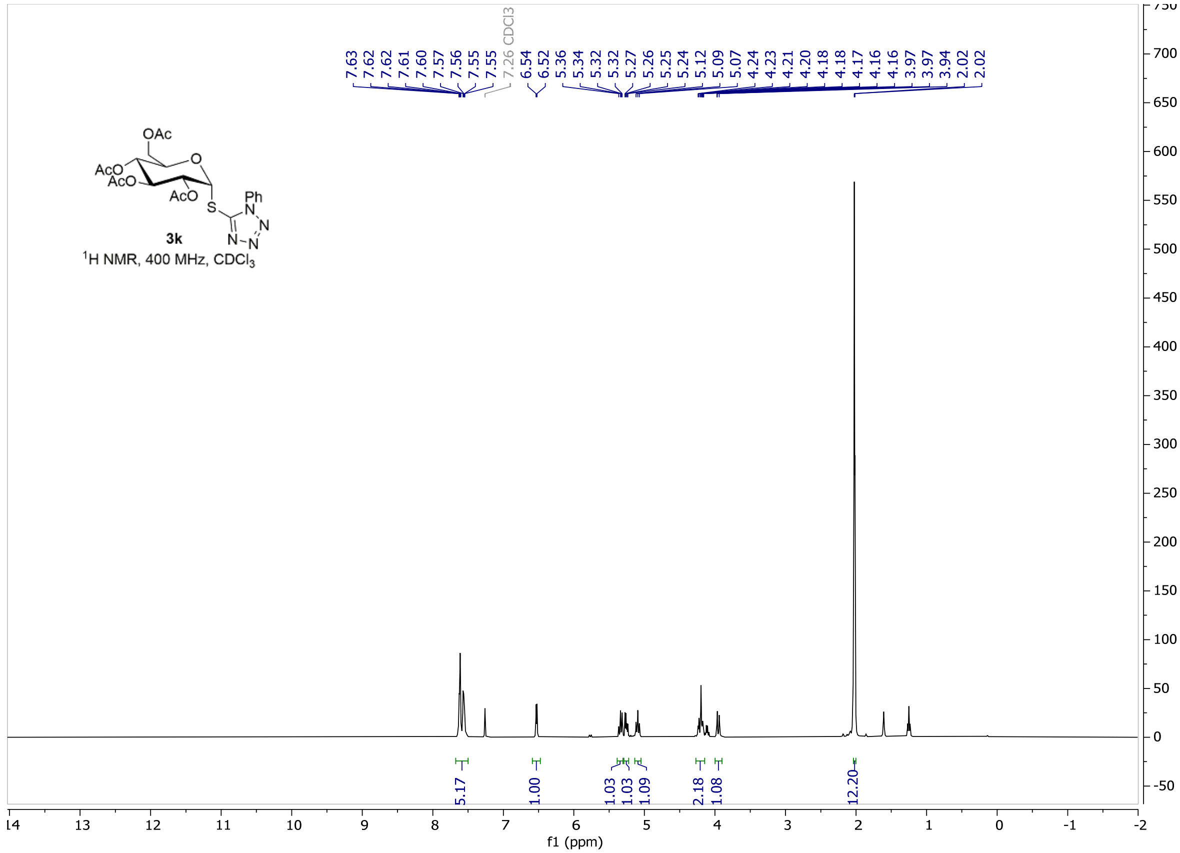
7.63
7.62
7.61
7.60
7.57
7.56
7.55
7.55
7.26 CDCl3
6.54
6.52
5.36
5.34
5.32
5.32
5.27
5.26
5.25
5.24
5.12
5.09
5.07
4.24
4.23
4.21
4.20
4.18
4.18
4.17
4.16
3.97
3.94
2.02
2.02
OAc
AcO
AcO
AcO
O
S
Ph
N
N
N
N
3k
^{1}H NMR, 400 MHz, $CDCl_3$
5.17
1.00
1.03
1.03
1.09
2.18
1.08
12.20
f1 (ppm)

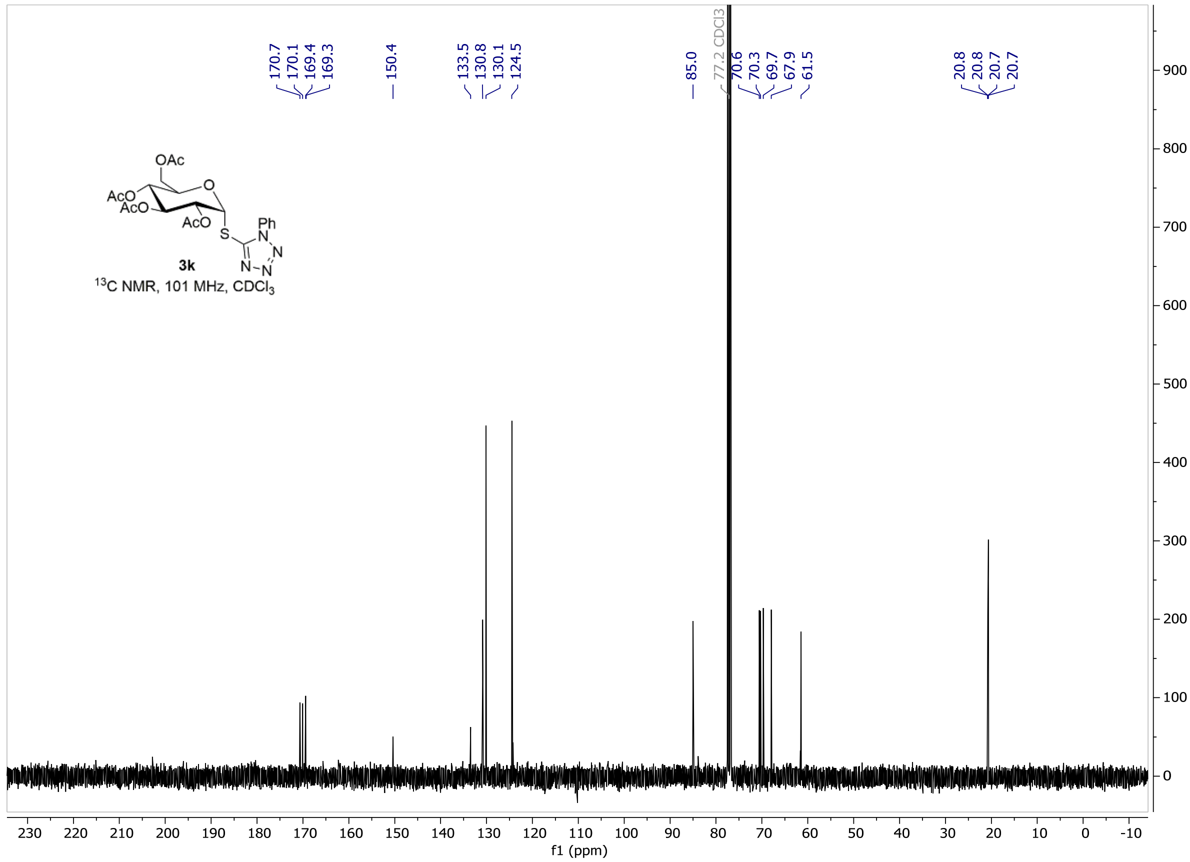
170.7
170.1
169.4
169.3
150.4
133.5
130.8
130.1
124.5
85.0
77.2 CDCl3
70.6
70.3
69.7
67.9
61.5
20.8
20.8
20.7
20.7
OAc
AcO
AcO
AcO
O
S
Ph
N
N
N
N
3k
^{13}C NMR, 101 MHz, $CDCl_3$
f1 (ppm)

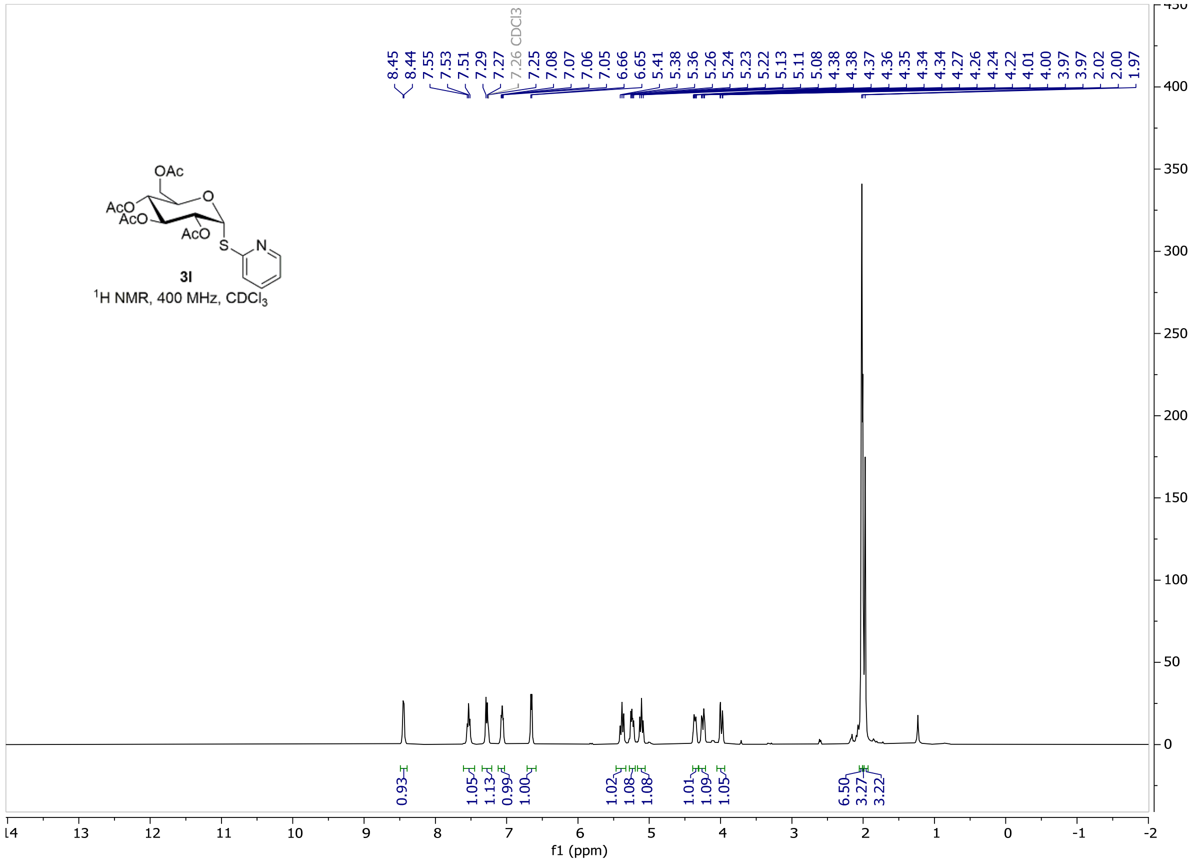
3l
¹H NMR, 400 MHz, CDCl₃
f1 (ppm)

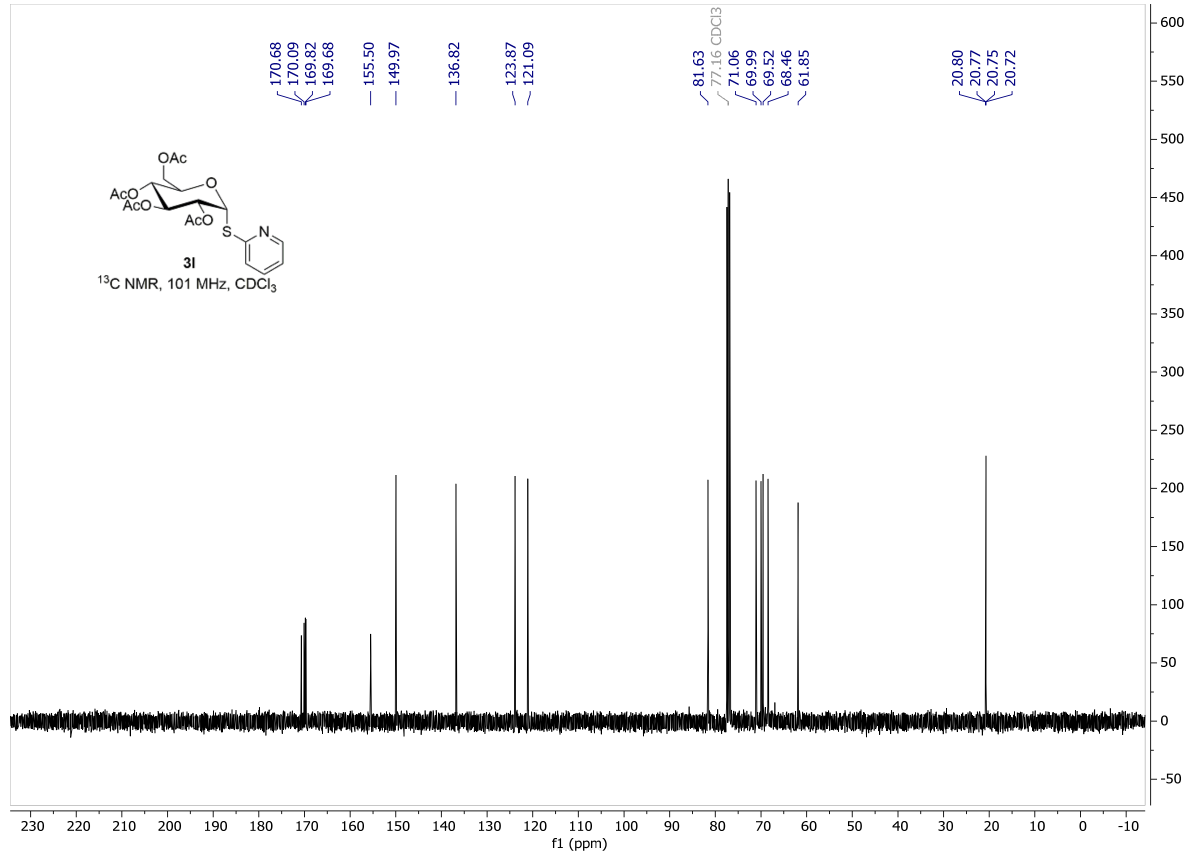
3l
¹³C NMR, 101 MHz, CDCl₃
f1 (ppm)

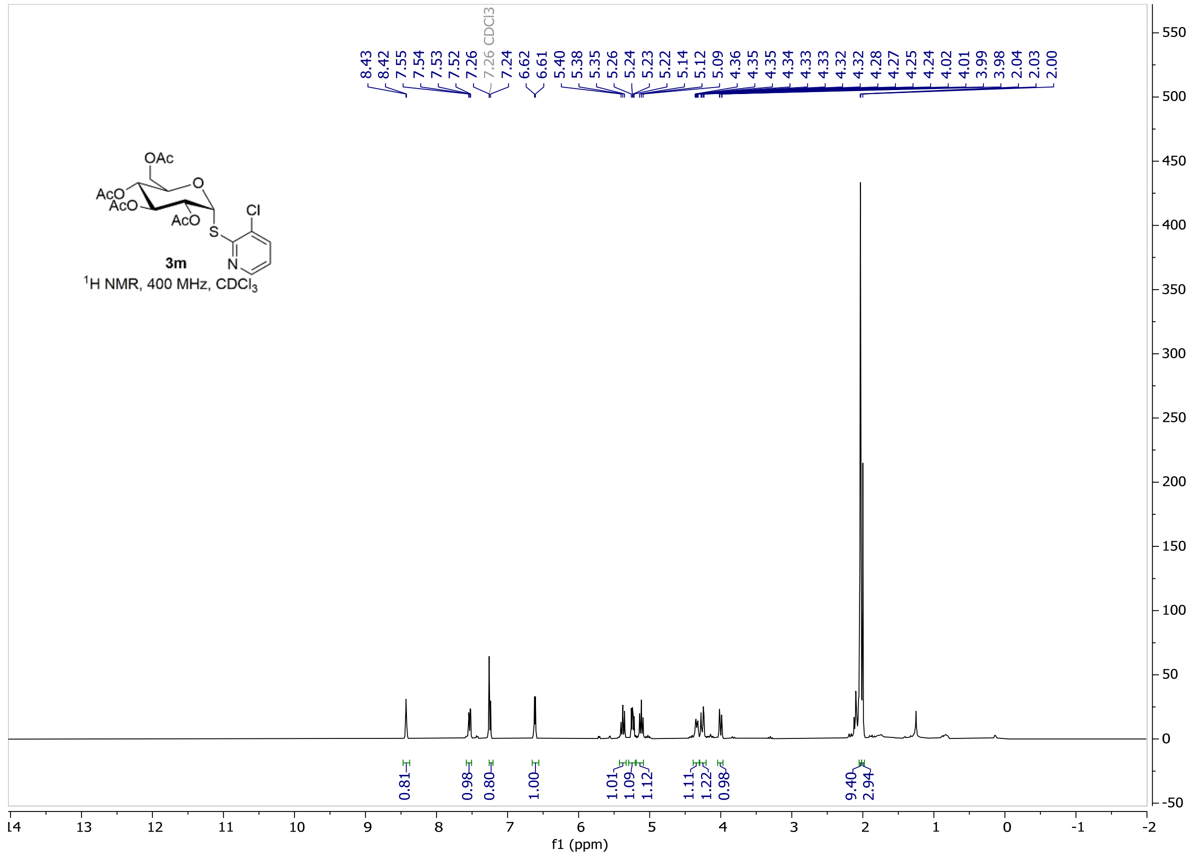

8.43
8.42
7.55
7.54
7.53
7.52
7.26
7.26 CDCl3
7.24
6.62
6.61
5.40
5.38
5.35
5.26
5.24
5.23
5.22
5.14
5.12
5.09
4.36
4.35
4.35
4.34
4.33
4.33
4.32
4.32
4.28
4.27
4.25
4.24
4.02
4.01
3.99
3.98
2.04
2.03
2.00
OAc
AcO
AcO
O
AcO
S
Cl
N
3m
^{1}H NMR, 400 MHz, CDCl$_3$
0.81
0.98
0.80
1.00
1.01
1.09
1.12
1.11
1.22
0.98
9.40
2.94
f1 (ppm)

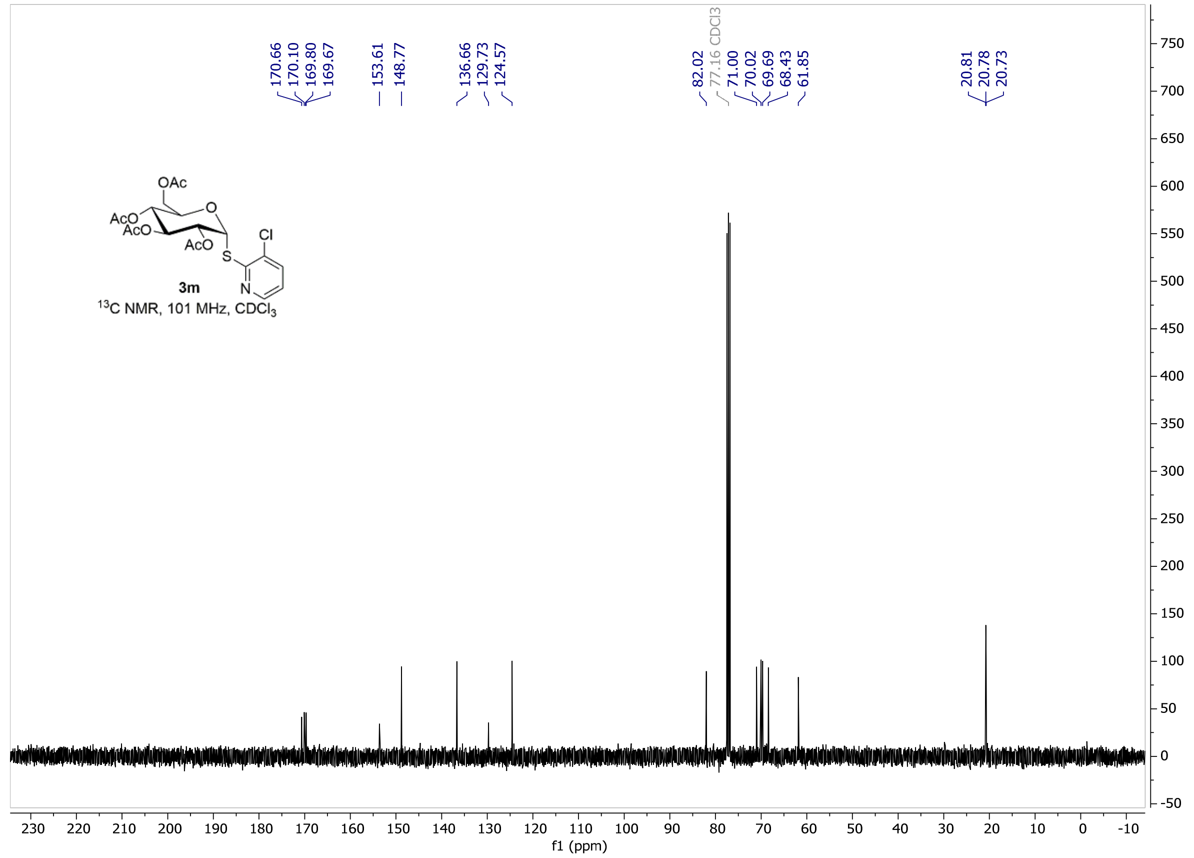

170.66
170.10
169.80
169.67
153.61
148.77
136.66
129.73
124.57
82.02
77.16 CDCl3
71.00
70.02
69.69
68.43
61.85
20.81
20.78
20.73
OAc
AcO
AcO
O
AcO
S
Cl
N
3m
^{13}C NMR, 101 MHz, CDCl$_3$
f1 (ppm)

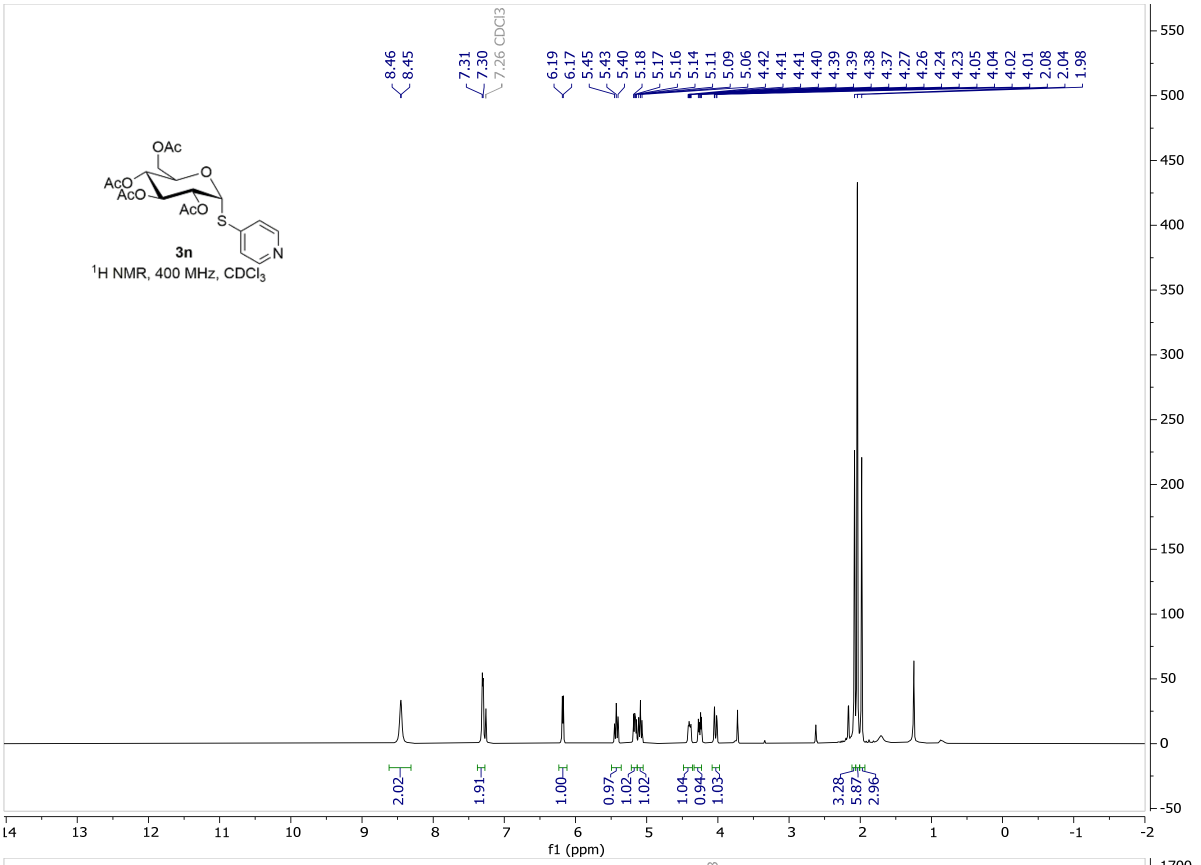
OAc
AcO
AcO
AcO
O
S
N
3n
^{1}H NMR, 400 MHz, CDCl$_3$
8.46
8.45
7.31
7.30
7.26 CDCl3
6.19
6.17
5.45
5.43
5.40
5.18
5.17
5.16
5.14
5.11
5.09
5.06
4.42
4.41
4.41
4.40
4.39
4.39
4.38
4.37
4.27
4.26
4.24
4.23
4.05
4.04
4.02
4.01
2.08
2.04
1.98
2.02
1.91
1.00
0.97
1.02
1.02
1.04
0.94
1.03
3.28
5.87
2.96
f1 (ppm)

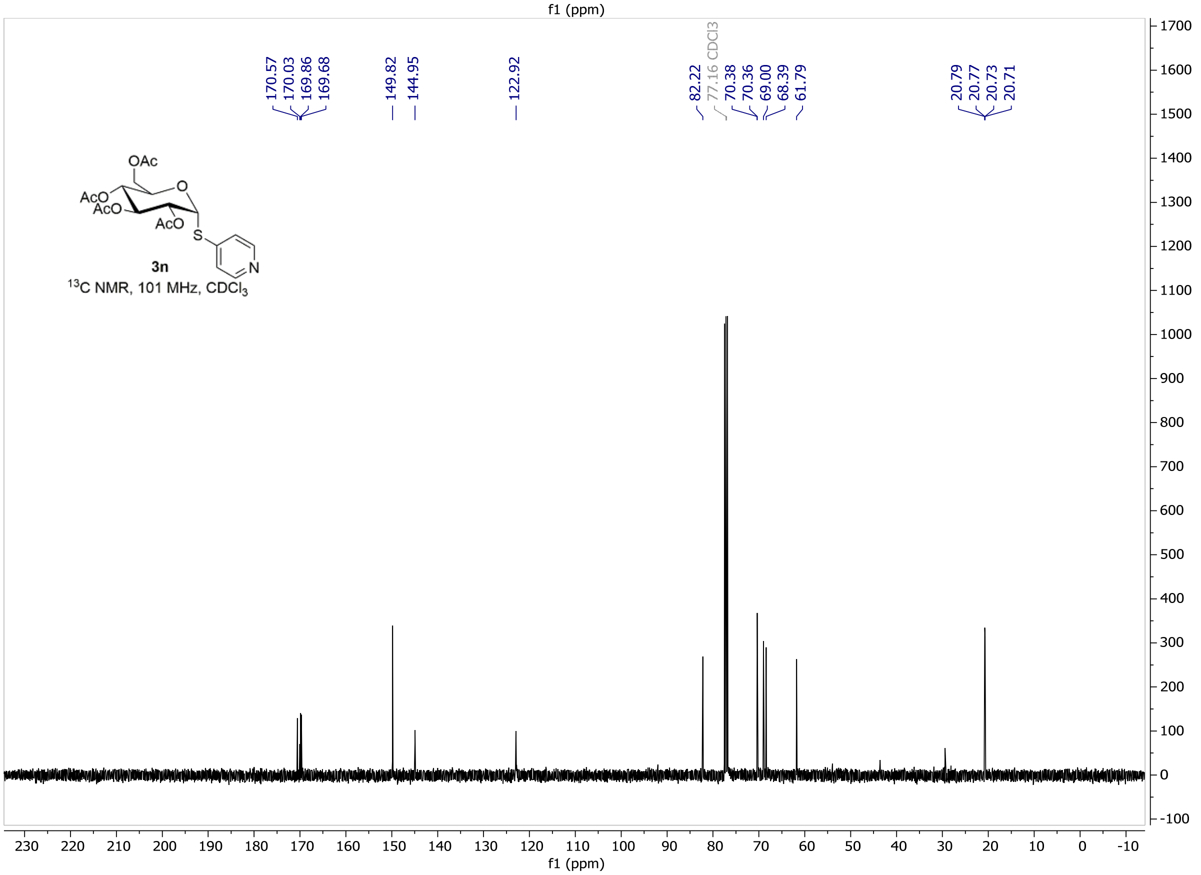
OAc
AcO
AcO
AcO
O
S
N
3n
^{13}C NMR, 101 MHz, CDCl$_3$
170.57
170.03
169.86
169.68
149.82
144.95
122.92
82.22
77.16 CDCl3
70.38
70.36
69.00
68.39
61.79
20.79
20.77
20.73
20.71
f1 (ppm)

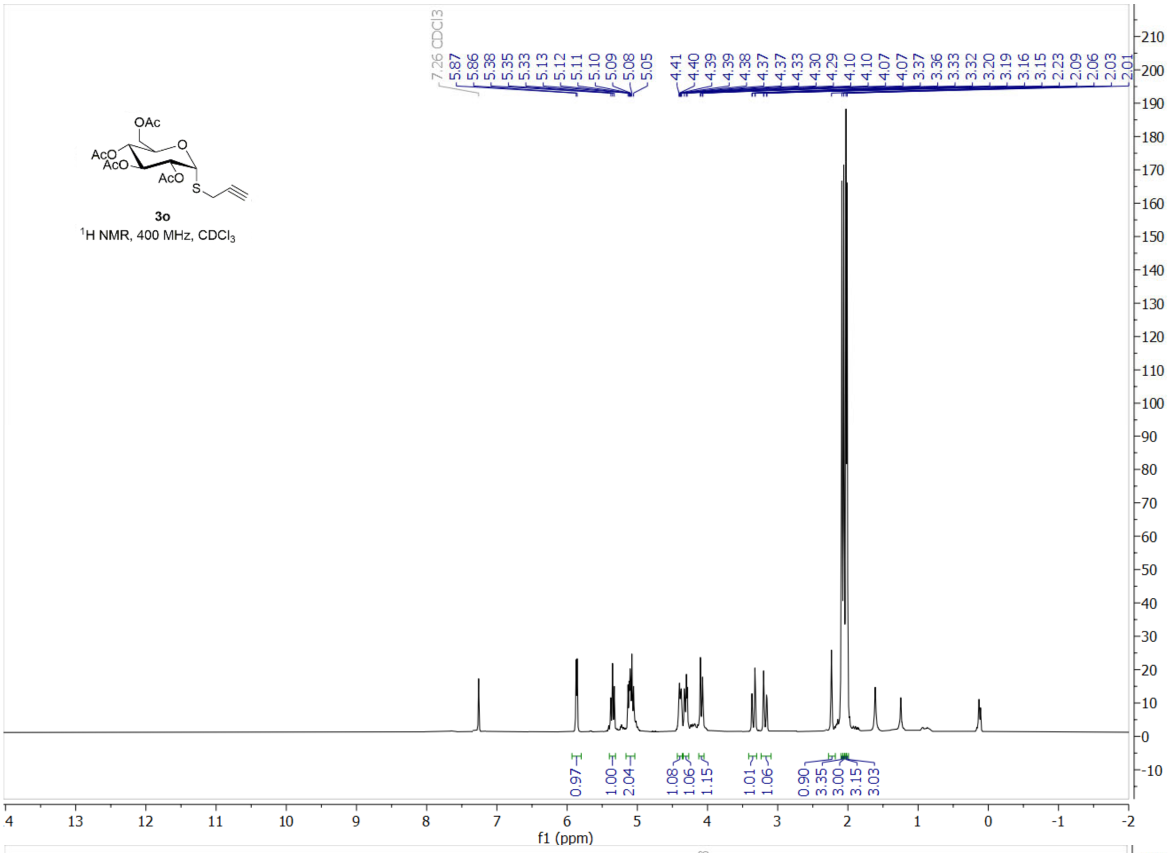

7.26 CDCl3
5.87
5.86
5.38
5.35
5.33
5.13
5.12
5.11
5.10
5.09
5.08
5.05
4.41
4.40
4.39
4.39
4.38
4.37
4.37
4.33
4.30
4.29
4.10
4.10
4.07
4.07
3.37
3.36
3.33
3.32
3.20
3.19
3.16
3.15
2.23
2.09
2.06
2.03
2.01
3o
^{1}H NMR, 400 MHz, CDCl$_3$
0.97
1.00
2.04
1.08
1.06
1.15
1.01
1.06
0.90
3.35
3.00
3.15
3.03
f1 (ppm)

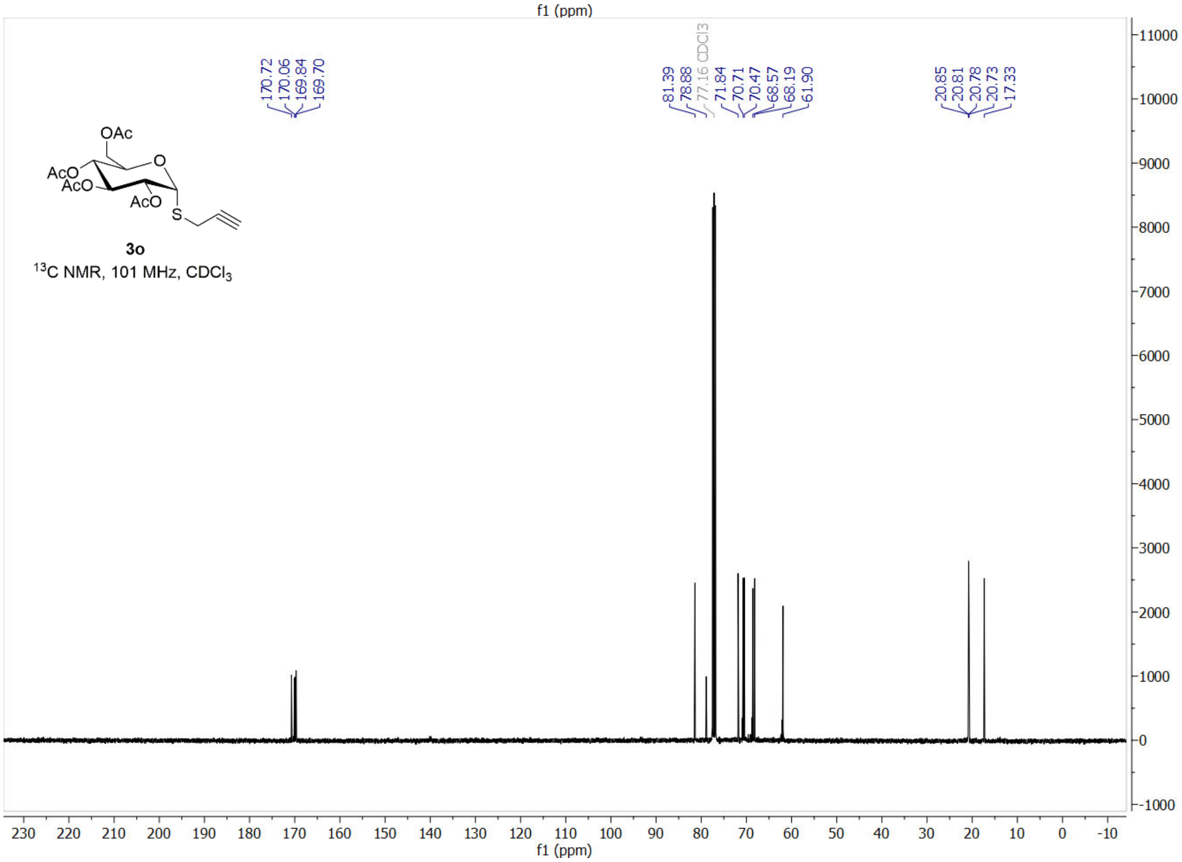

170.72
170.06
169.84
169.70
81.39
78.88
77.16 CDCl3
71.84
70.71
70.47
68.57
68.19
61.90
20.85
20.81
20.78
20.73
17.33
3o
^{13}C NMR, 101 MHz, CDCl$_3$
f1 (ppm)

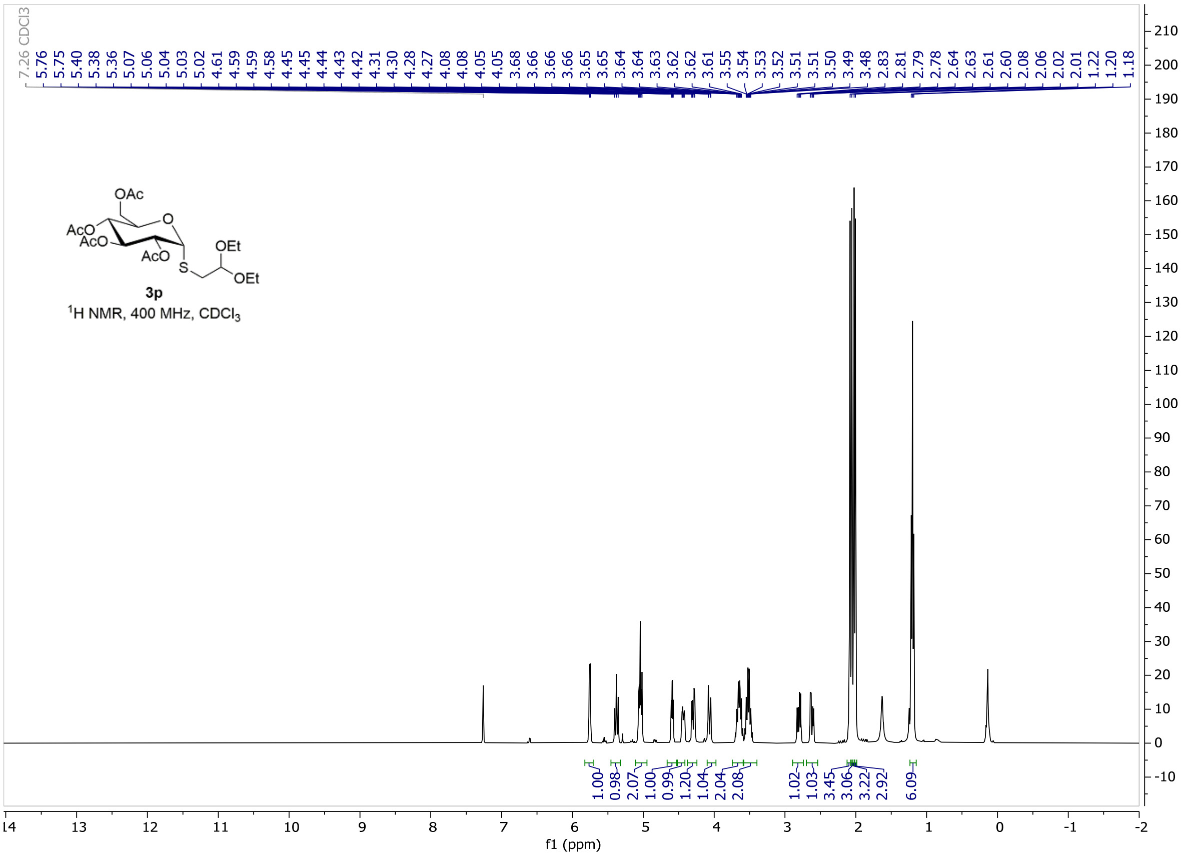
3p
^{1}H NMR, 400 MHz, $CDCl_3$
f1 (ppm)

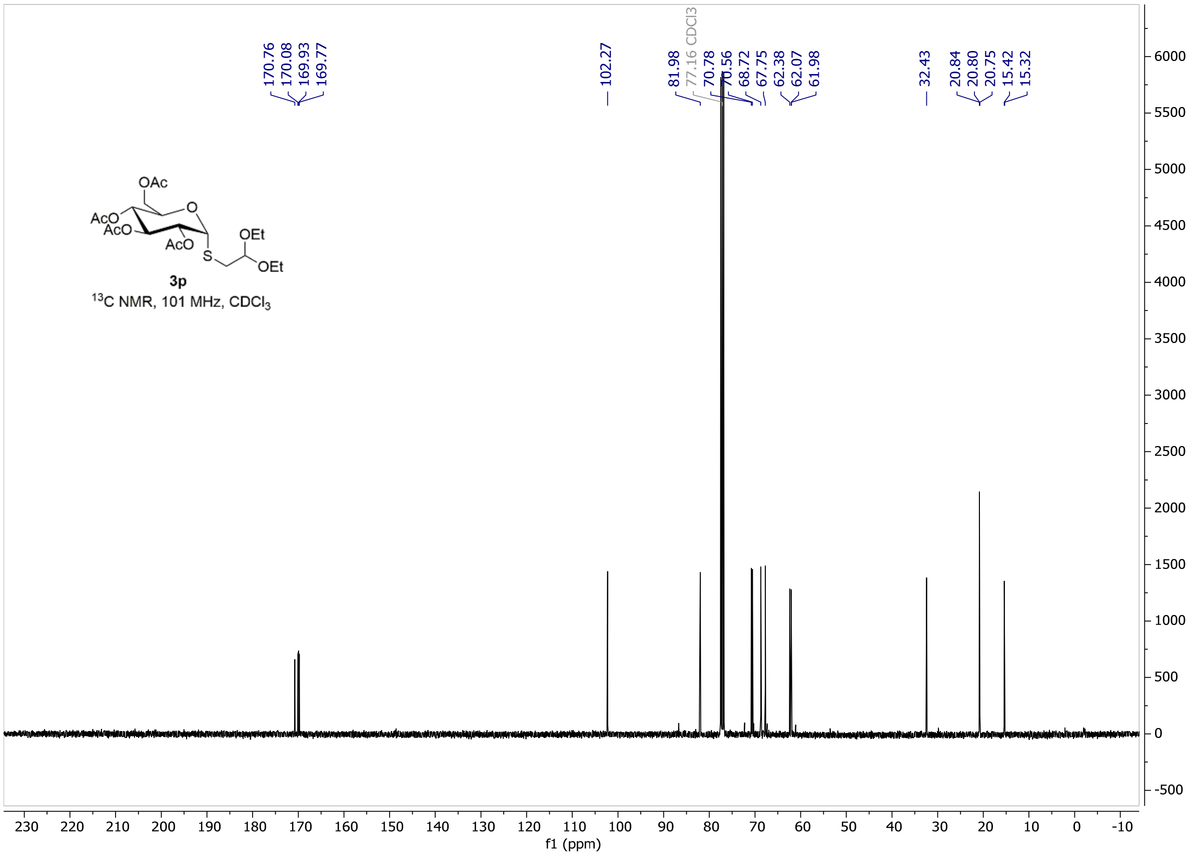
3p
^{13}C NMR, 101 MHz, $CDCl_3$
f1 (ppm)

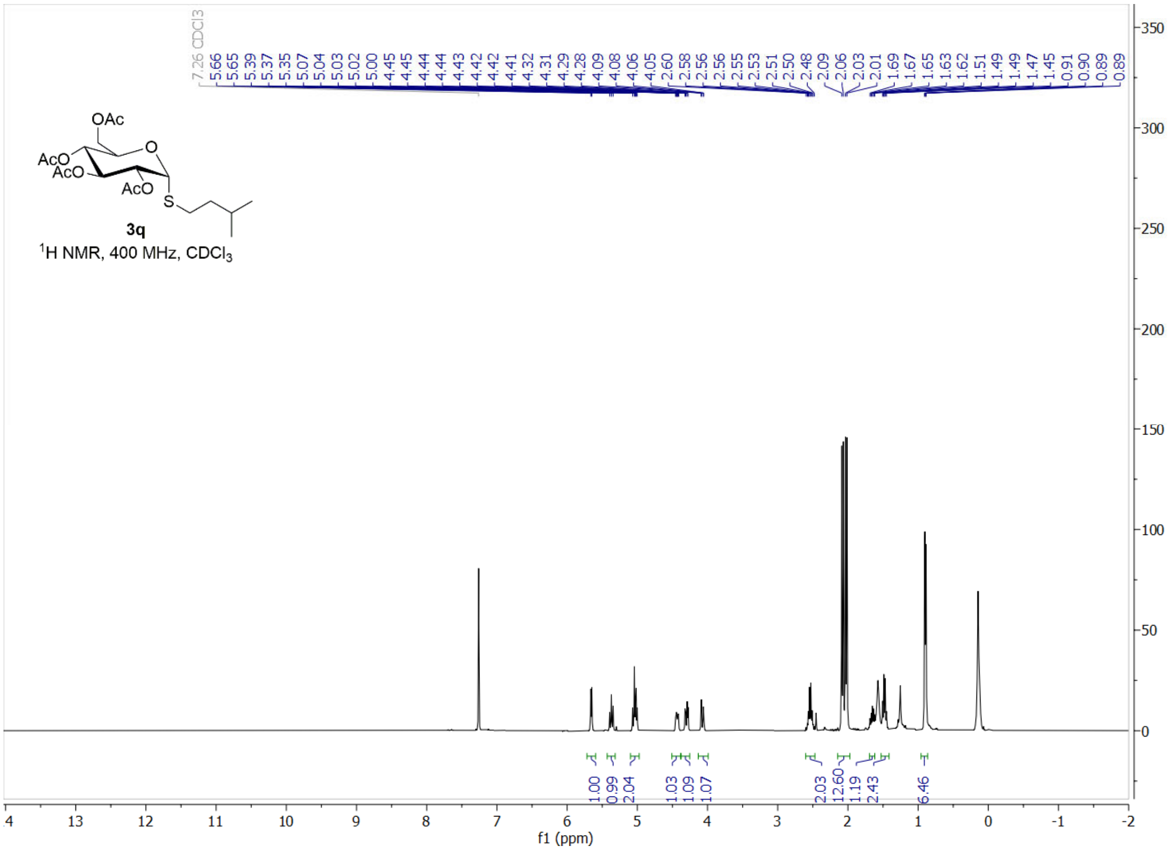

3q
^{1}H NMR, 400 MHz, CDCl$_3$
f1 (ppm)

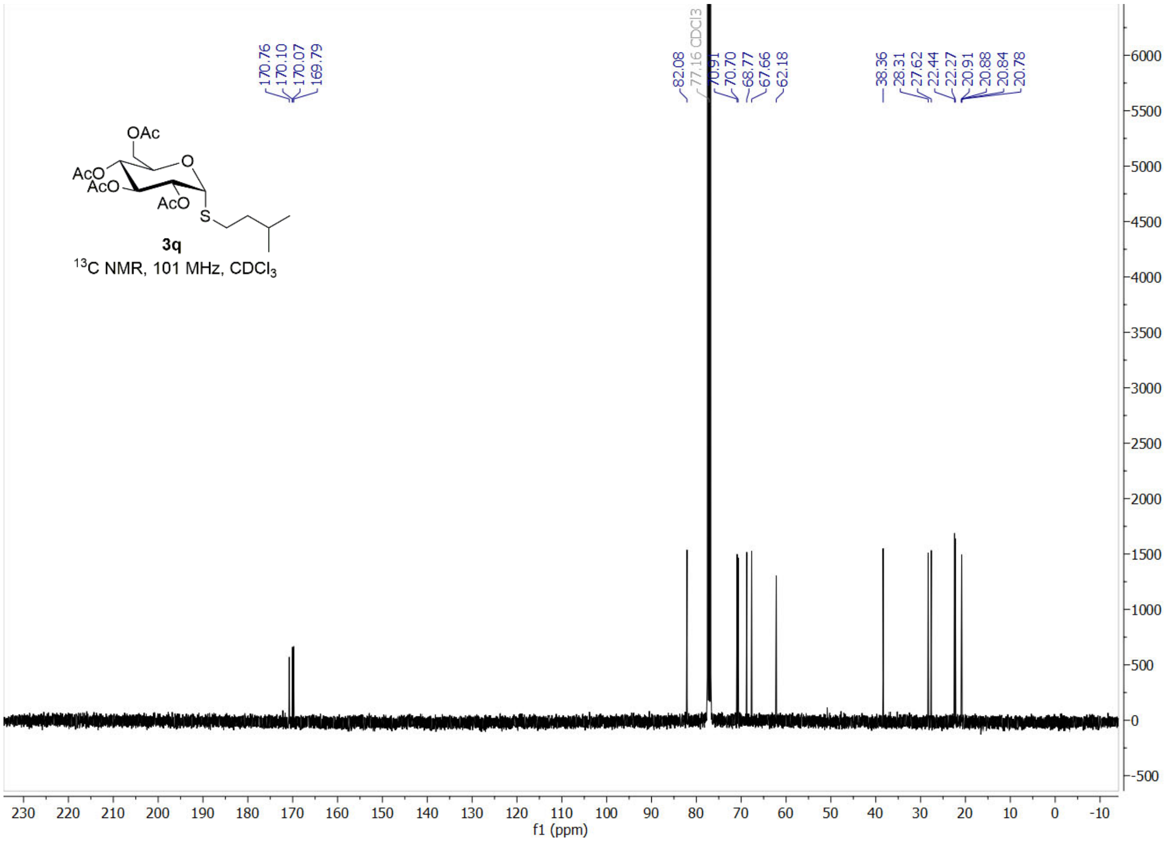

3q
^{13}C NMR, 101 MHz, CDCl$_3$
f1 (ppm)

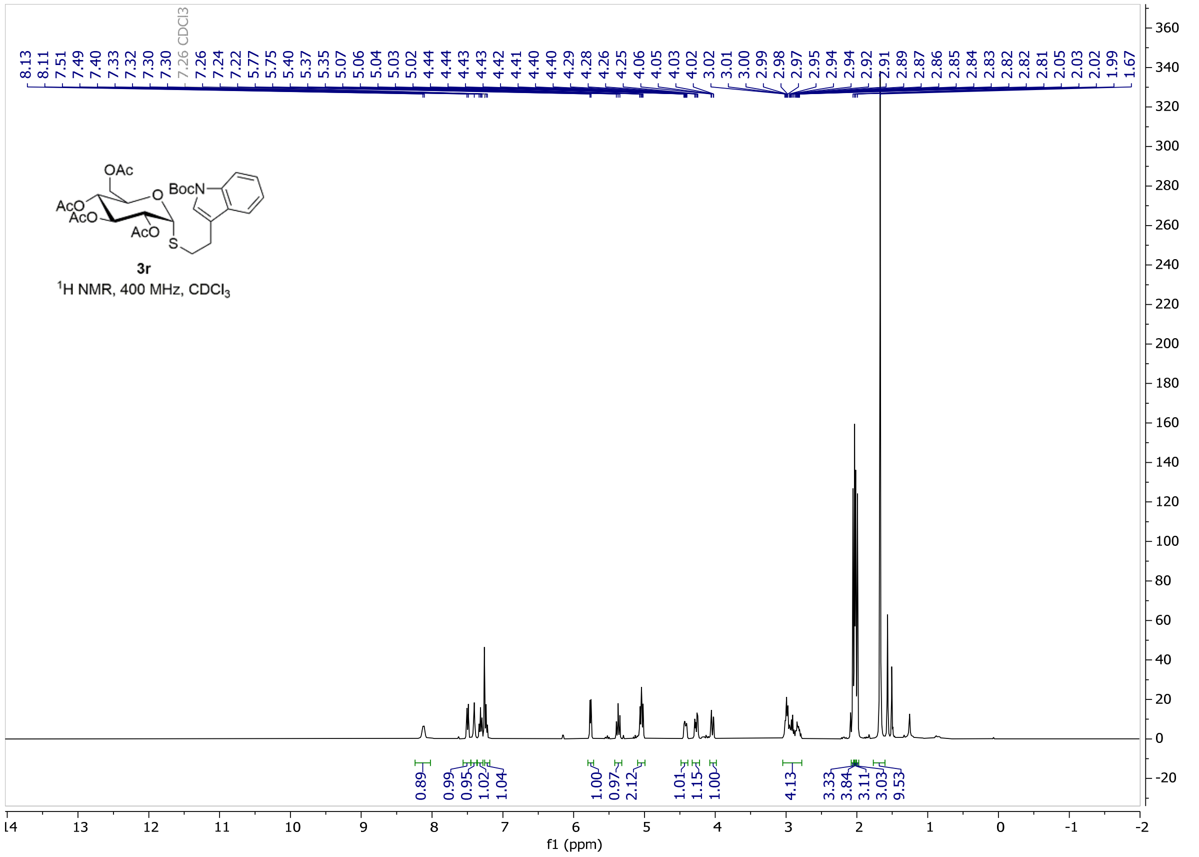
3r
^{1}H NMR, 400 MHz, CDCl$_3$
8.13 8.11 7.51 7.49 7.40 7.33 7.32 7.30 7.30 7.26 CDCl3 7.26 7.24 7.22 5.77 5.75 5.40 5.37 5.35 5.07 5.06 5.04 5.03 5.02 4.44 4.44 4.43 4.43 4.42 4.41 4.40 4.29 4.28 4.26 4.25 4.06 4.05 4.03 4.02 3.02 3.01 3.00 2.99 2.98 2.97 2.95 2.94 2.94 2.92 2.91 2.89 2.87 2.86 2.85 2.84 2.83 2.82 2.82 2.81 2.05 2.03 2.02 1.99 1.67
0.89 0.99 0.95 1.02 1.04 1.00 0.97 2.12 1.01 1.15 1.00 4.13 3.33 3.84 3.11 3.03 9.53
f1 (ppm)

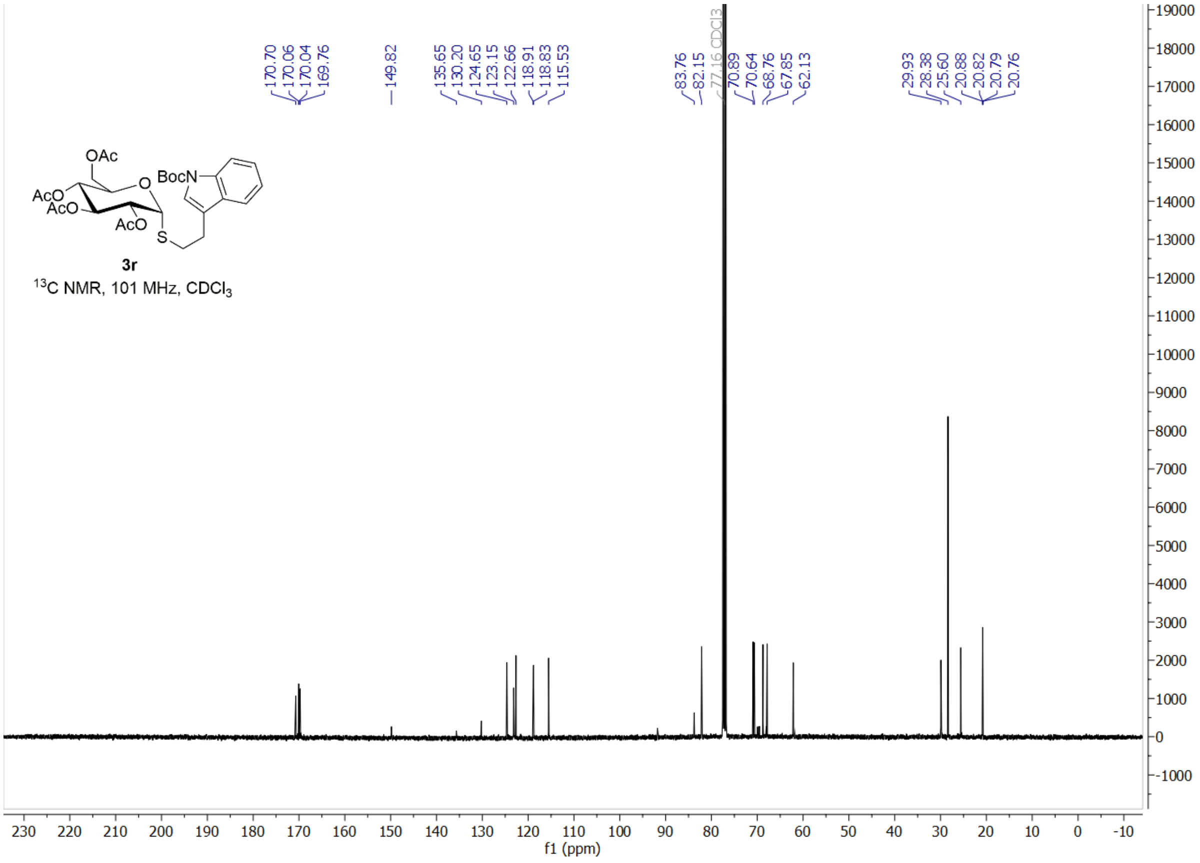
3r
^{13}C NMR, 101 MHz, CDCl$_3$
170.70 170.06 170.04 169.76 149.82 135.65 130.20 124.65 123.15 122.66 118.91 118.83 115.53 83.76 82.15 77.16 CDCl3 70.89 70.64 68.76 67.85 62.13 29.93 28.38 25.60 20.88 20.82 20.79 20.76
f1 (ppm)

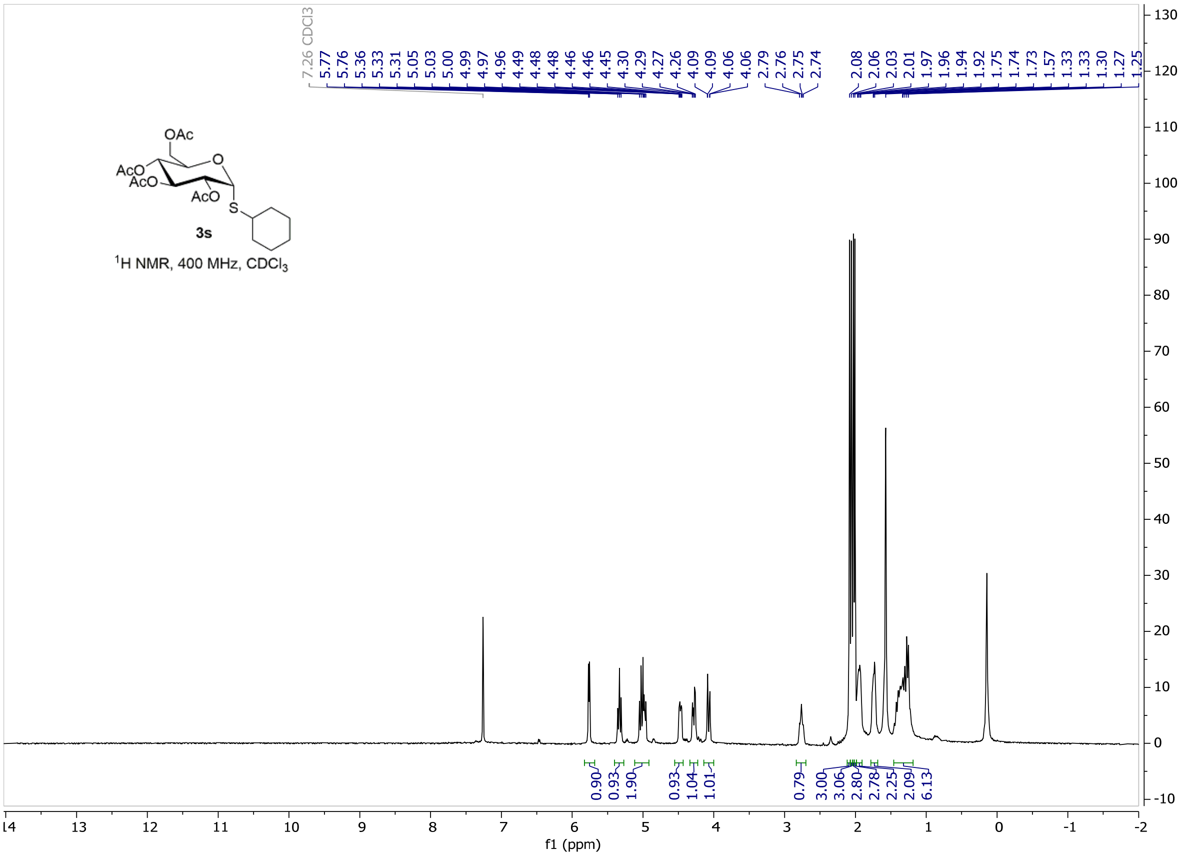
OAc
AcO
AcO
AcO
S
3s
^{1}H NMR, 400 MHz, CDCl$_3$
7.26 CDCl3
5.77 5.76 5.36 5.33 5.31 5.05 5.03 5.00 4.99 4.97 4.96 4.49 4.48 4.46 4.46 4.45 4.30 4.29 4.27 4.26 4.09 4.09 4.06 4.06 2.79 2.76 2.75 2.74 2.08 2.06 2.03 2.01 1.97 1.96 1.94 1.92 1.75 1.74 1.73 1.57 1.33 1.30 1.27 1.25
0.90 0.93 1.90 0.93 1.04 1.01 0.79 3.00 3.06 2.80 2.78 2.25 2.09 6.13
f1 (ppm)

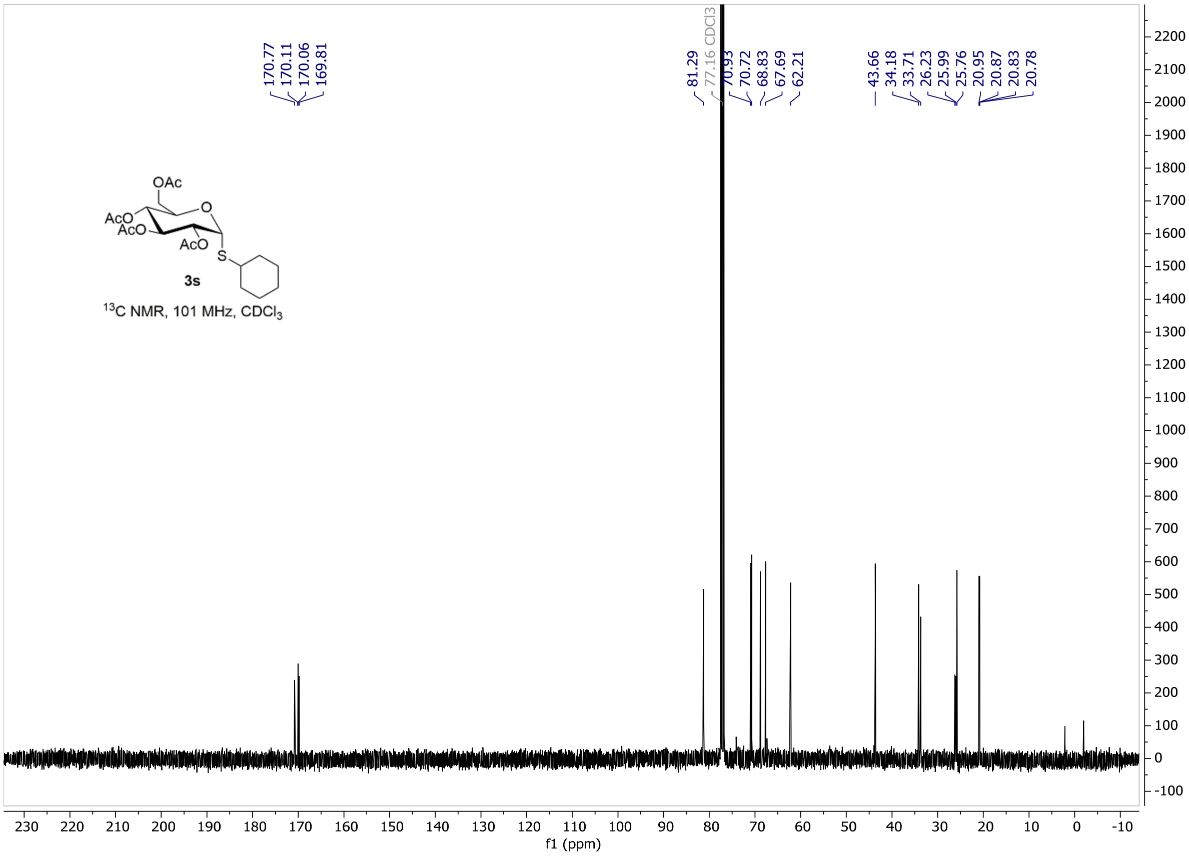
OAc
AcO
AcO
AcO
S
3s
^{13}C NMR, 101 MHz, CDCl$_3$
170.77 170.11 170.06 169.81
81.29 77.16 CDCl3 70.93 70.72 68.83 67.69 62.21
43.66 34.18 33.71 26.23 25.99 25.76 20.95 20.87 20.83 20.78
f1 (ppm)

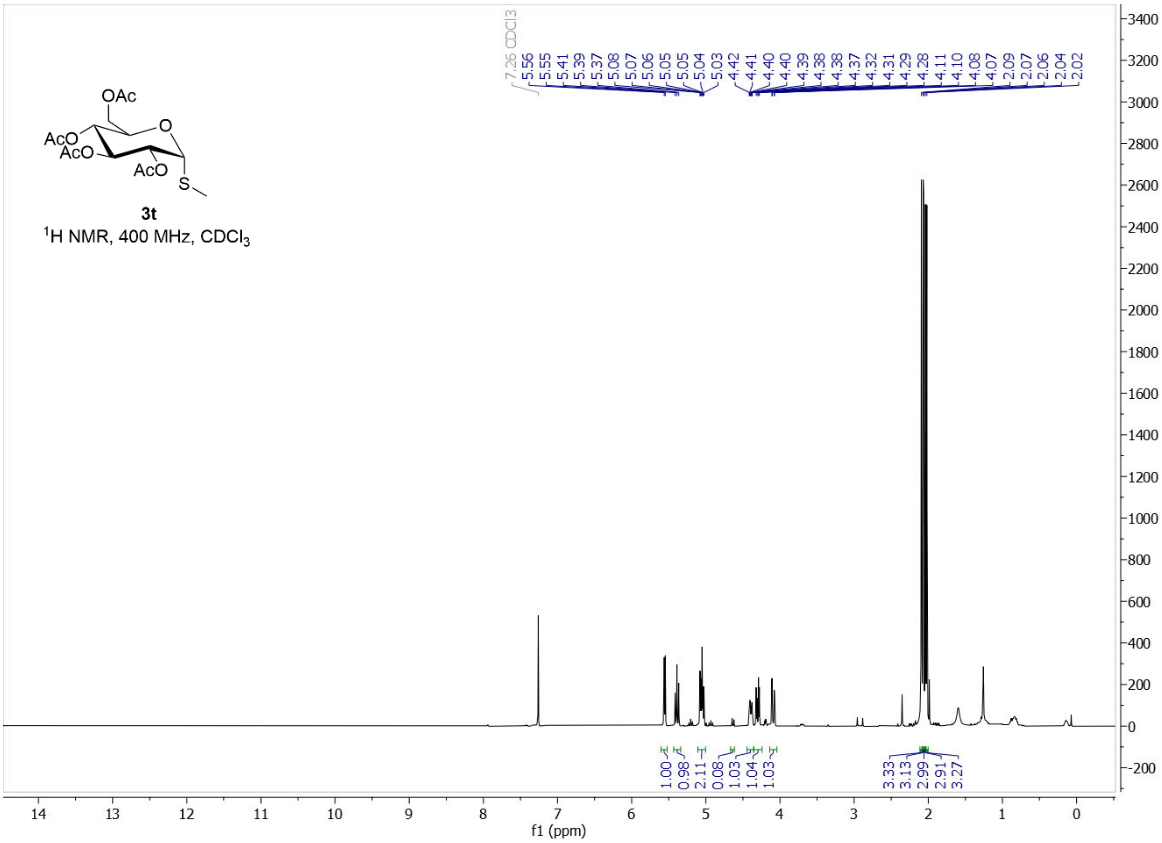

OAc
AcO
AcO
AcO
O
S
3t
^{1}H NMR, 400 MHz, CDCl$_3$
7.26 CDCl3
5.56
5.55
5.41
5.39
5.37
5.08
5.07
5.06
5.05
5.05
5.04
5.03
4.42
4.41
4.40
4.39
4.38
4.38
4.37
4.32
4.31
4.29
4.28
4.11
4.10
4.08
4.07
2.09
2.07
2.06
2.04
2.02
1.00
0.98
2.11
0.08
1.03
1.04
1.03
3.33
3.13
2.99
2.91
3.27
f1 (ppm)

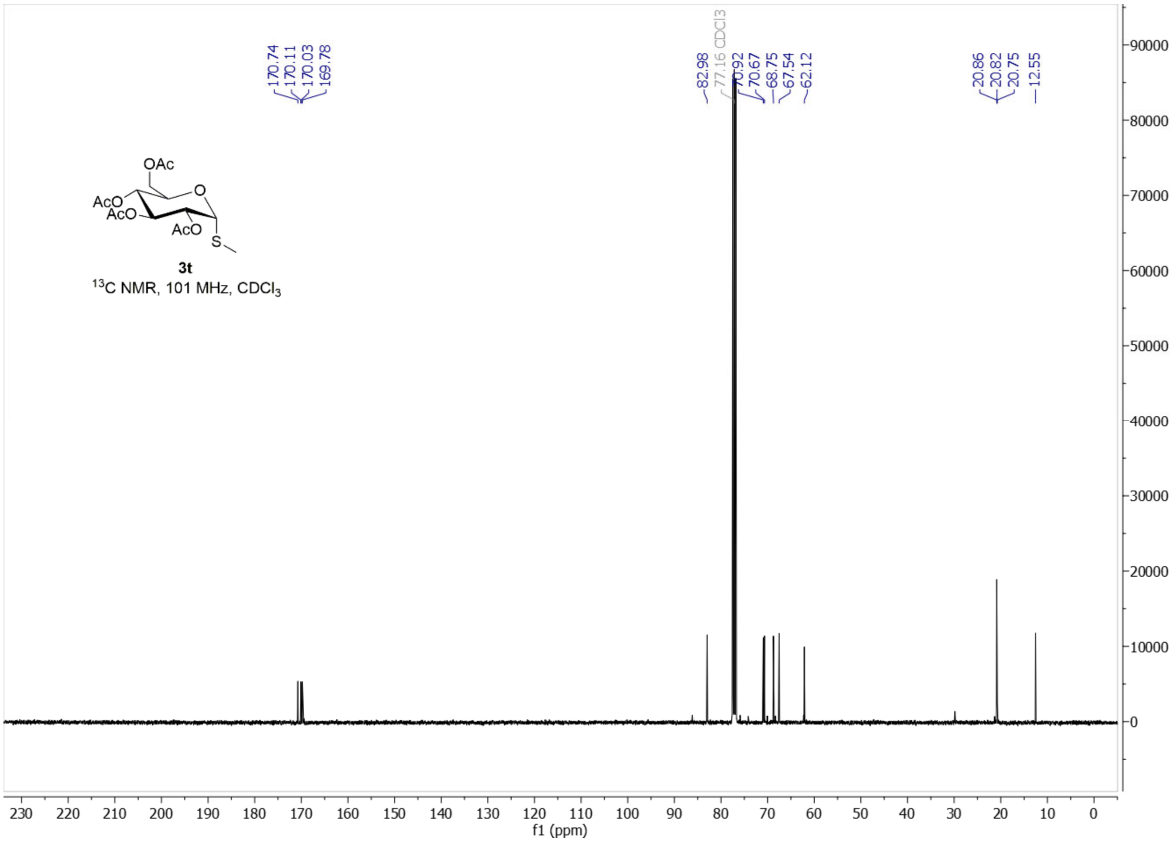

OAc
AcO
AcO
AcO
O
S
3t
^{13}C NMR, 101 MHz, CDCl$_3$
170.74
170.11
170.03
169.78
82.98
77.16 CDCl3
70.92
70.67
68.75
67.54
62.12
20.86
20.82
20.75
12.55
f1 (ppm)

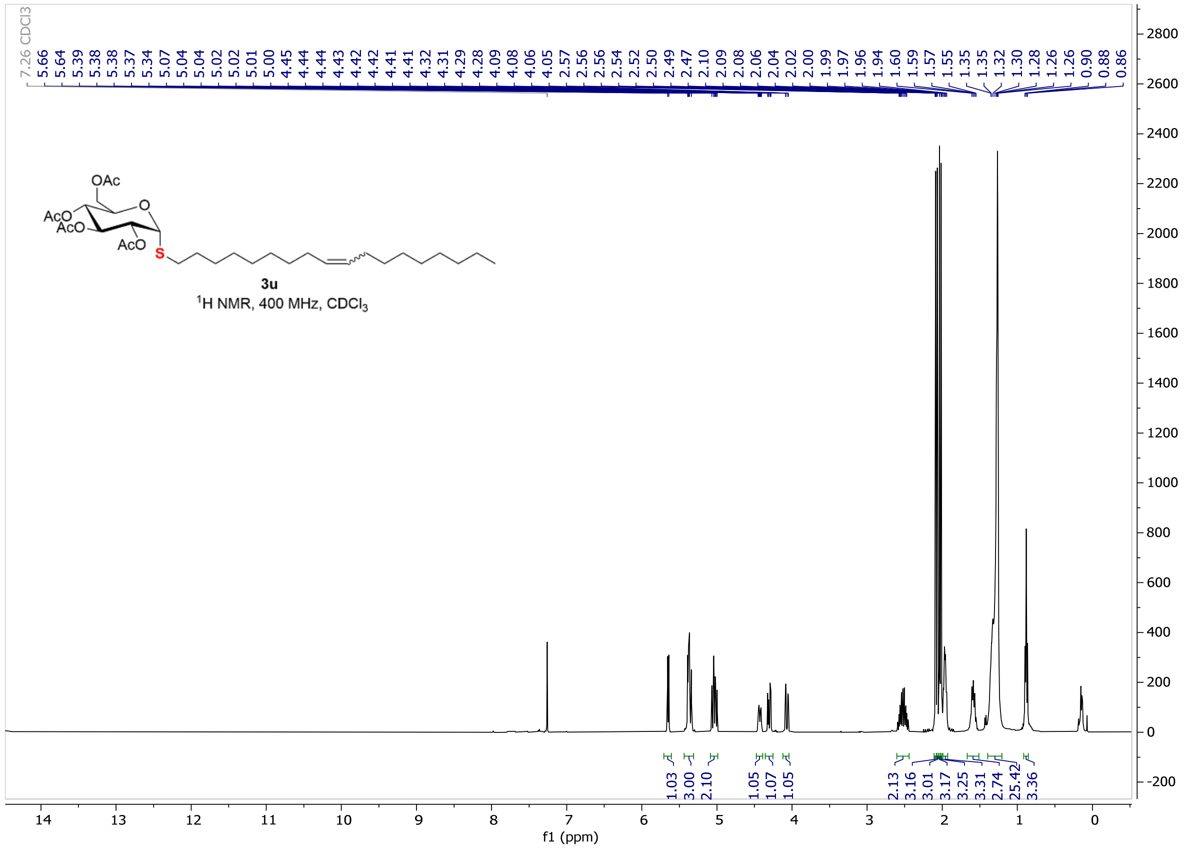

3u
^{1}H NMR, 400 MHz, CDCl$_3$
f1 (ppm)

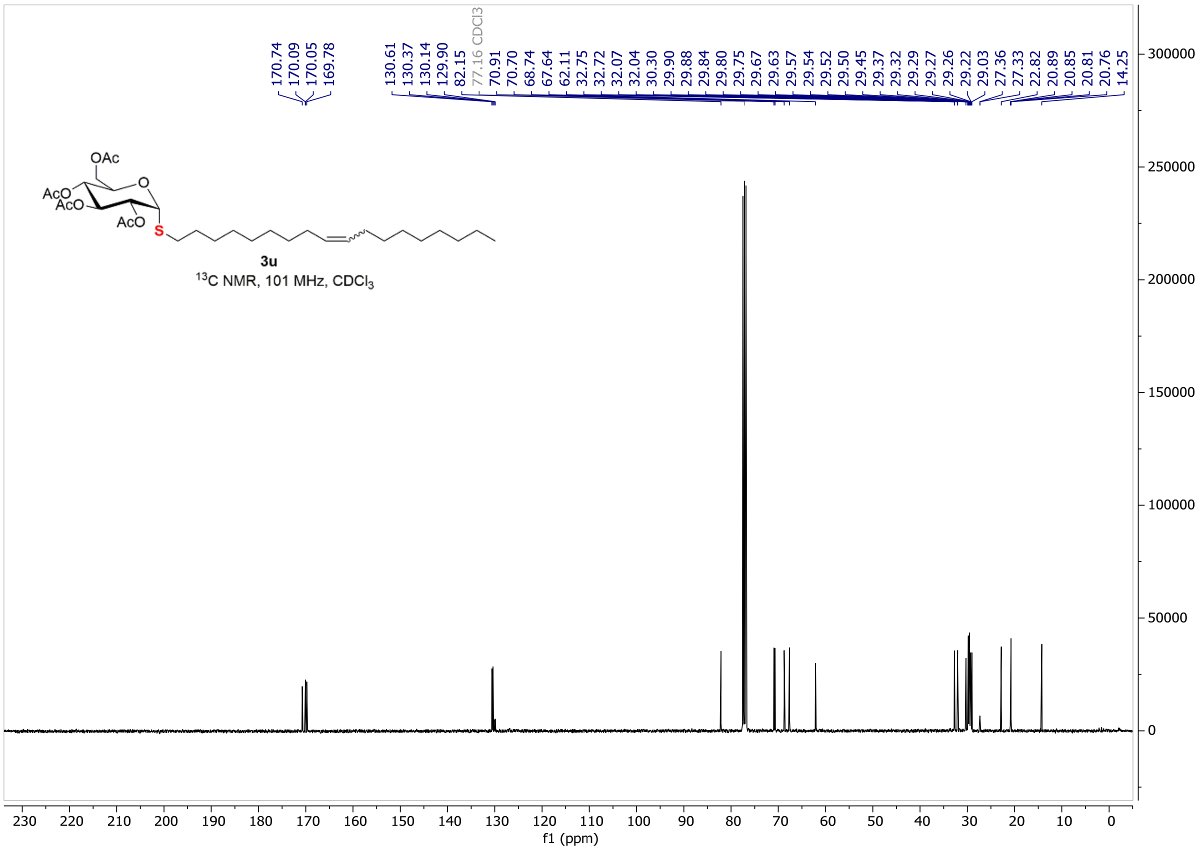

3u
^{13}C NMR, 101 MHz, CDCl$_3$
f1 (ppm)

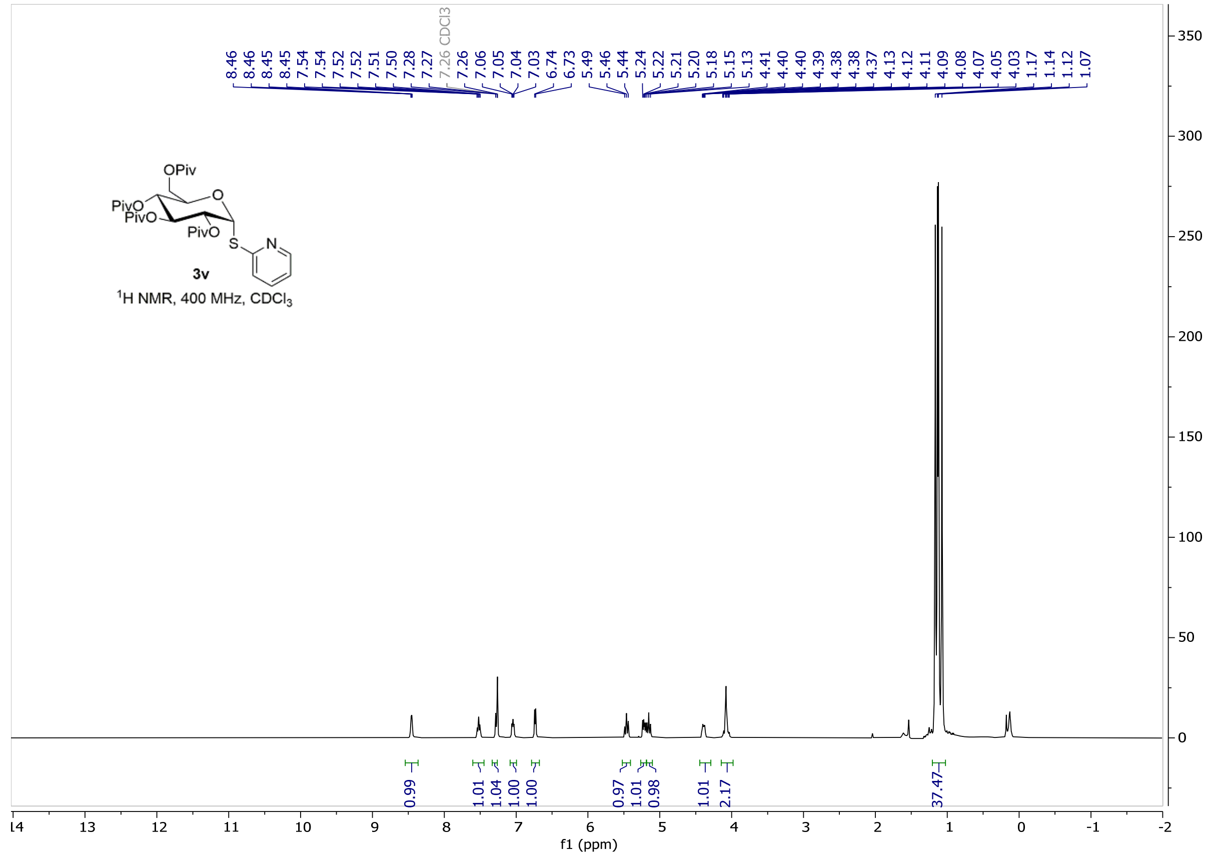

3v
^{1}H NMR, 400 MHz, CDCl3
f1 (ppm)

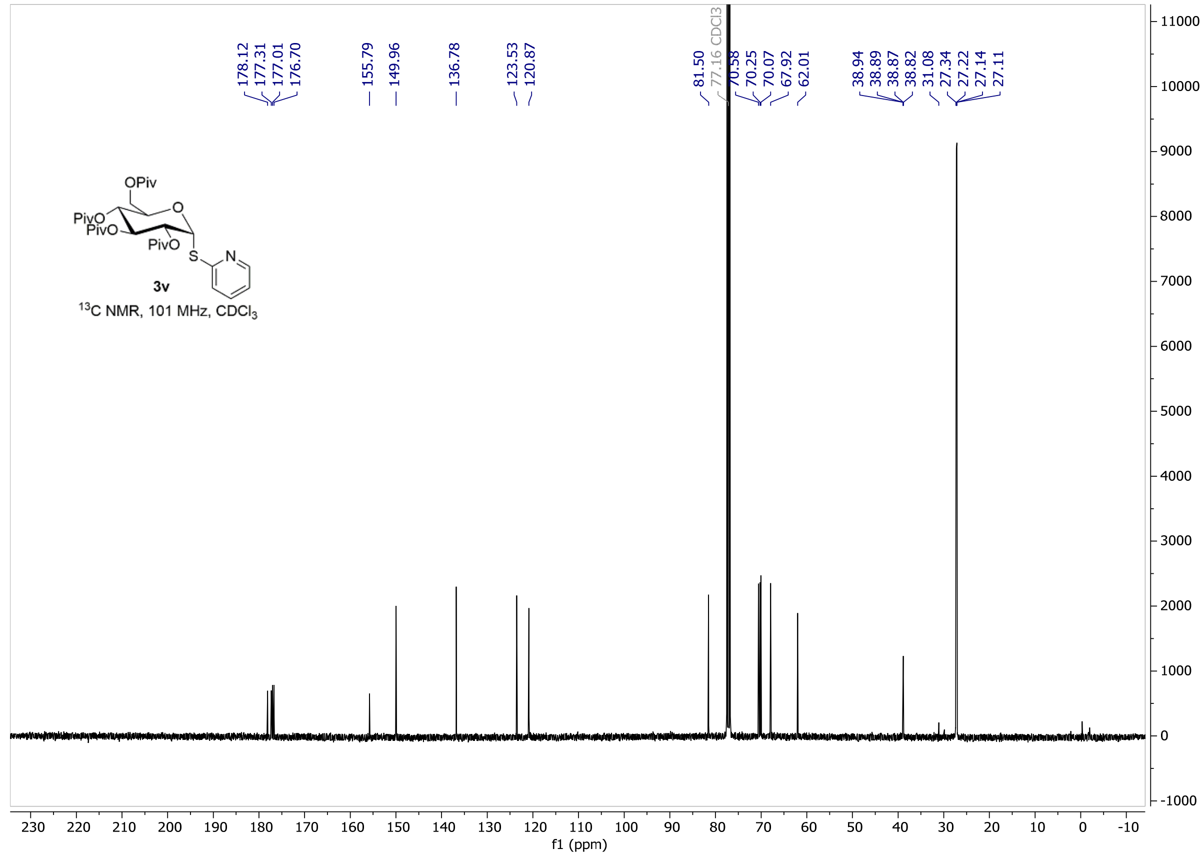

3v
^{13}C NMR, 101 MHz, CDCl3
f1 (ppm)

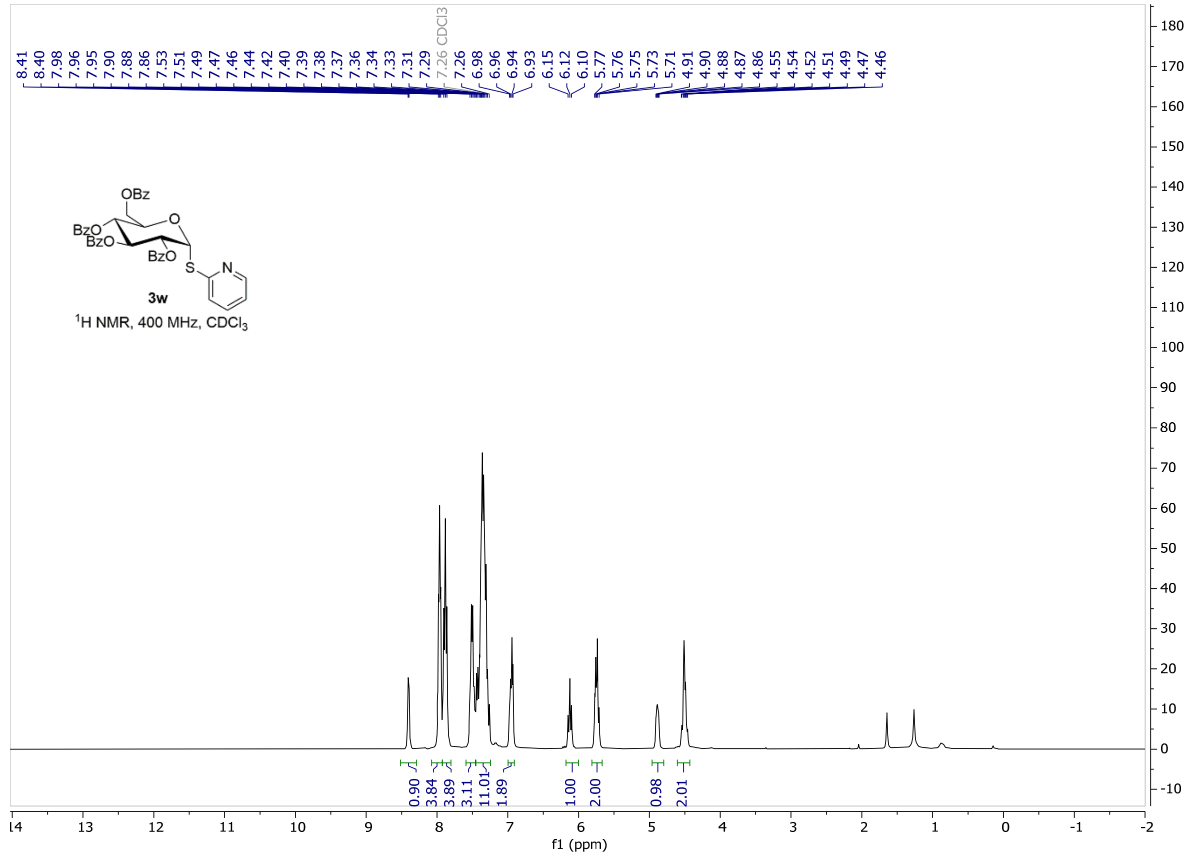

8.41
8.40
7.98
7.96
7.95
7.90
7.88
7.86
7.53
7.51
7.49
7.47
7.46
7.44
7.42
7.40
7.39
7.38
7.37
7.36
7.34
7.33
7.31
7.29
7.26 CDCl3
7.26
6.98
6.96
6.94
6.93
6.15
6.12
6.10
5.77
5.76
5.75
5.73
5.71
4.91
4.90
4.88
4.87
4.86
4.55
4.54
4.52
4.51
4.49
4.47
4.46
OBz
BzO
BzO
BzO
O
S
N
3w
1H NMR, 400 MHz, CDCl3
0.90
3.84
3.89
3.11
11.01
1.89
1.00
2.00
0.98
2.01
f1 (ppm)

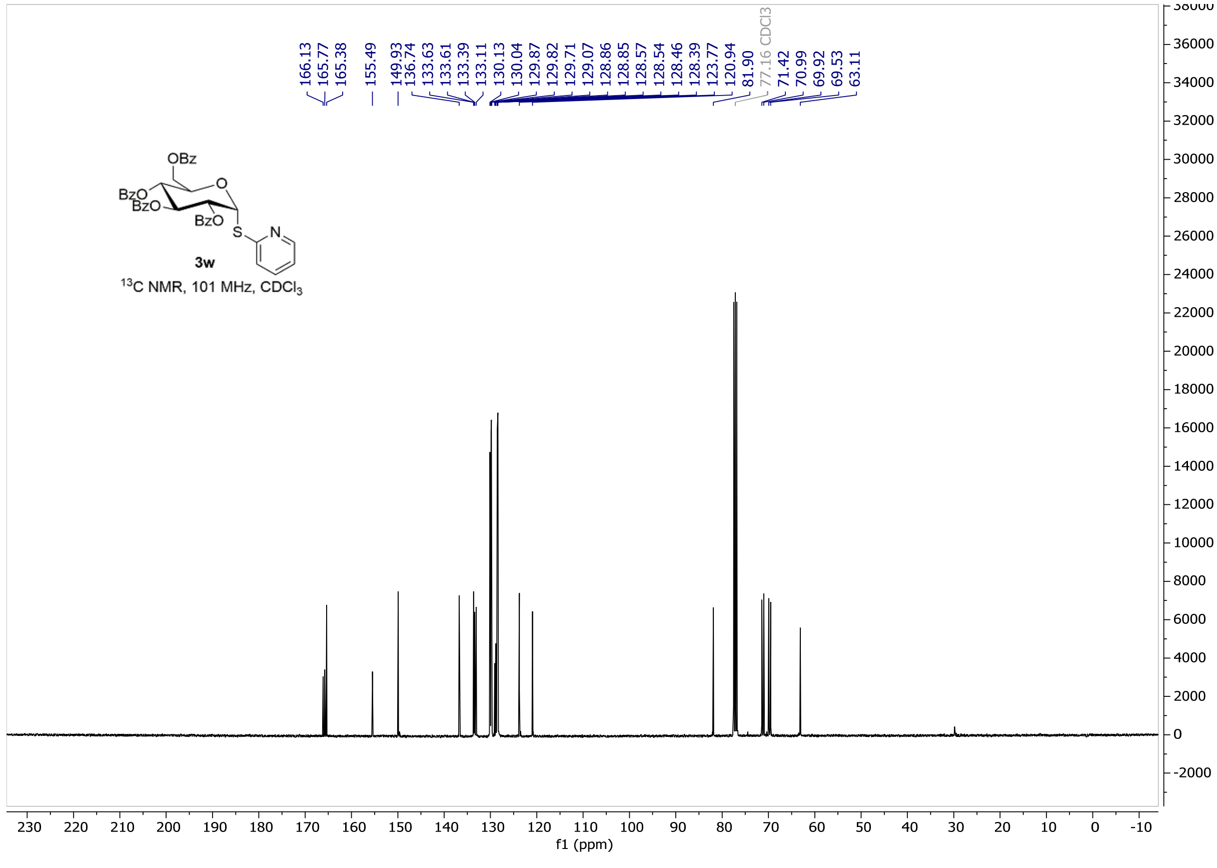

166.13
165.77
165.38
155.49
149.93
136.74
133.63
133.61
133.39
133.11
130.13
130.04
129.87
129.82
129.71
129.07
128.86
128.85
128.57
128.54
128.46
128.39
123.77
120.94
81.90
77.16 CDCl3
71.42
70.99
69.92
69.53
63.11
OBz
BzO
BzO
BzO
O
S
N
3w
13C NMR, 101 MHz, CDCl3
f1 (ppm)

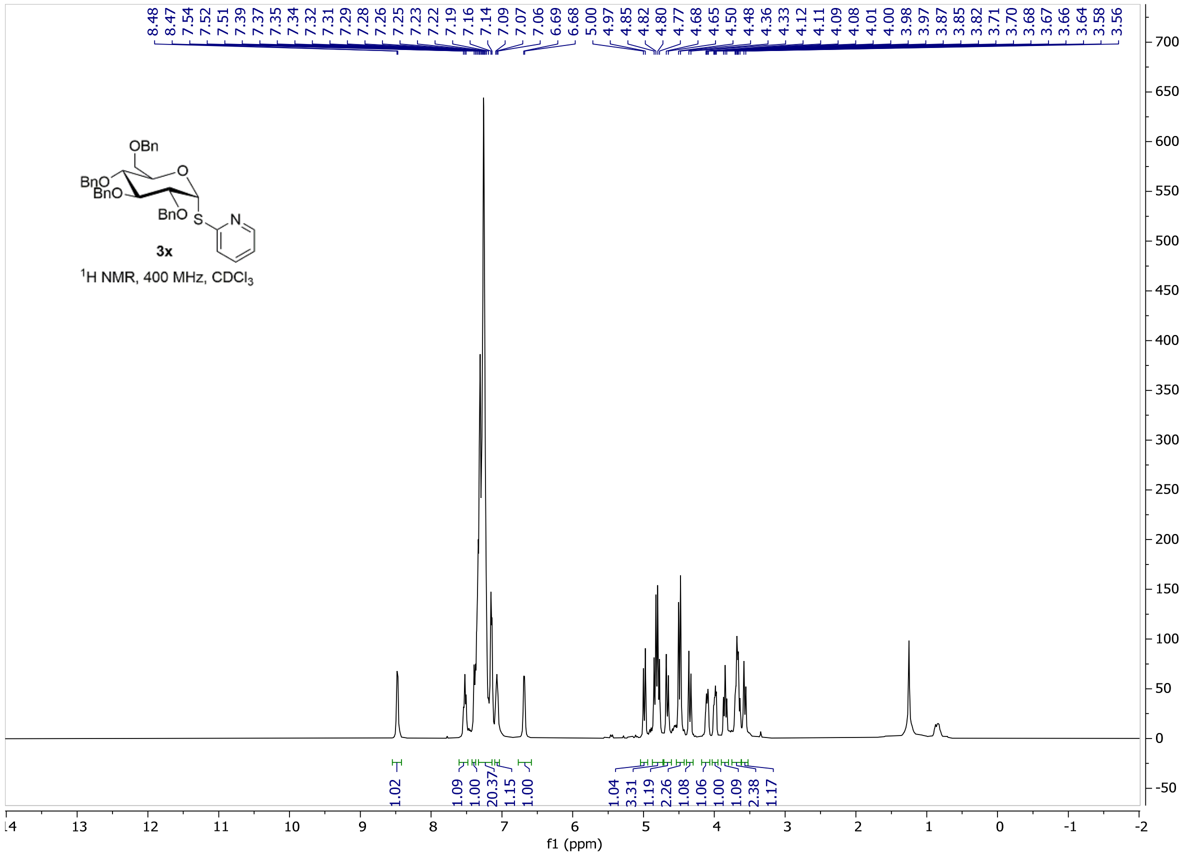

OBn
BnO
BnO
BnO
S
N
3x
^{1}H NMR, 400 MHz, CDCl$_3$
f1 (ppm)

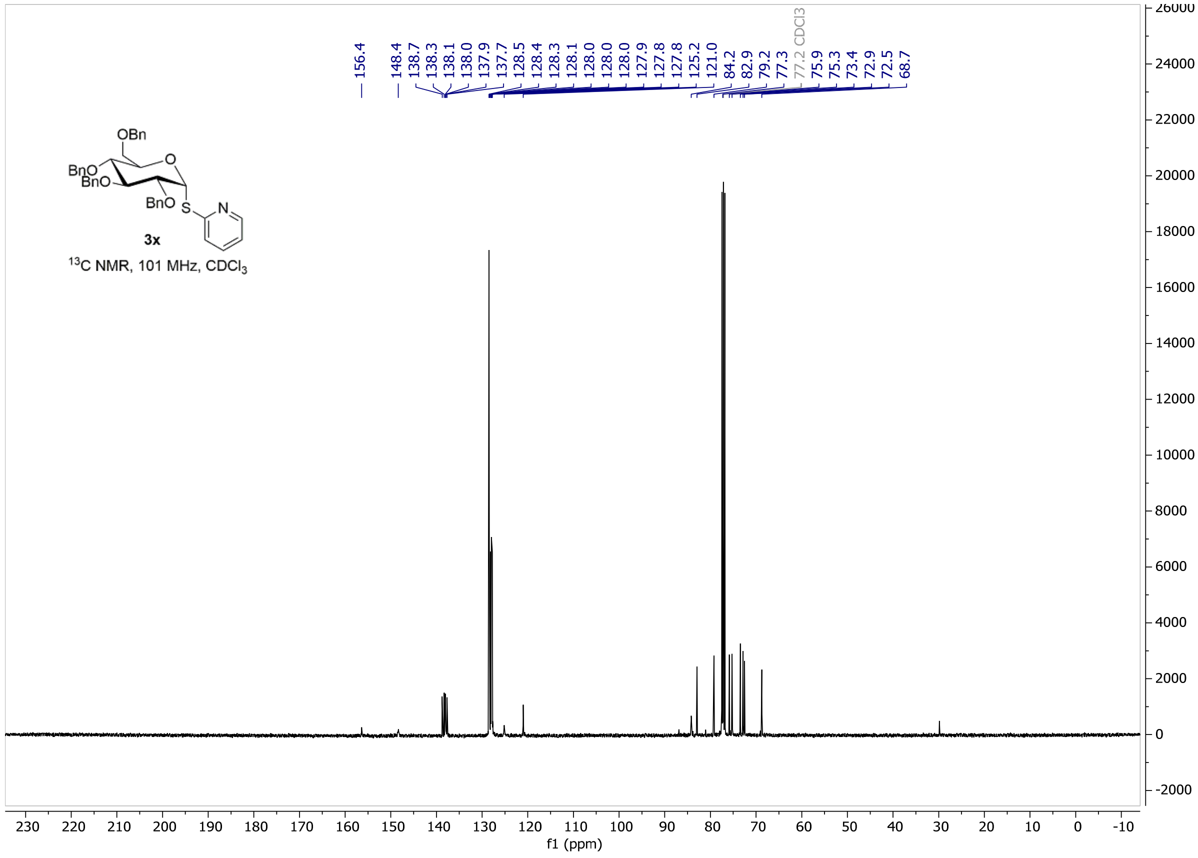

OBn
BnO
BnO
BnO
S
N
3x
^{13}C NMR, 101 MHz, CDCl$_3$
77.2 CDCl3
f1 (ppm)

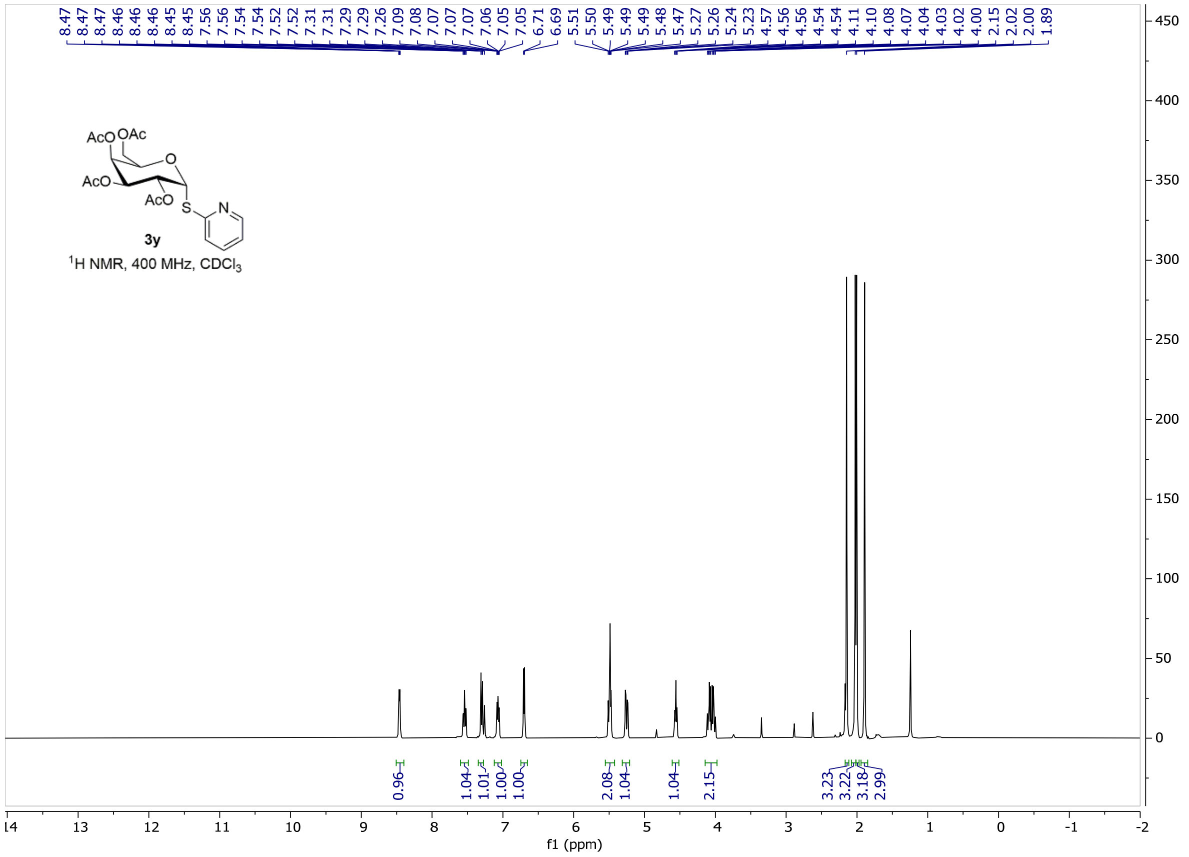
8.47 8.47 8.47 8.46 8.46 8.46 8.45 8.45 7.56 7.56 7.54 7.54 7.52 7.52 7.31 7.31 7.29 7.26 7.09 7.08 7.07 7.07 7.07 7.06 7.05 7.05 6.71 6.69 5.51 5.50 5.49 5.49 5.49 5.48 5.47 5.27 5.26 5.24 5.23 4.57 4.56 4.56 4.54 4.54 4.11 4.10 4.08 4.07 4.04 4.03 4.02 4.00 2.15 2.02 2.00 1.89
AcO OAc
O
AcO
AcO
S
N
3y
^{1}H NMR, 400 MHz, CDCl$_3$
0.96
1.04
1.01
1.00
1.00
2.08
1.04
1.04
2.15
3.23
3.22
3.18
2.99
f1 (ppm)

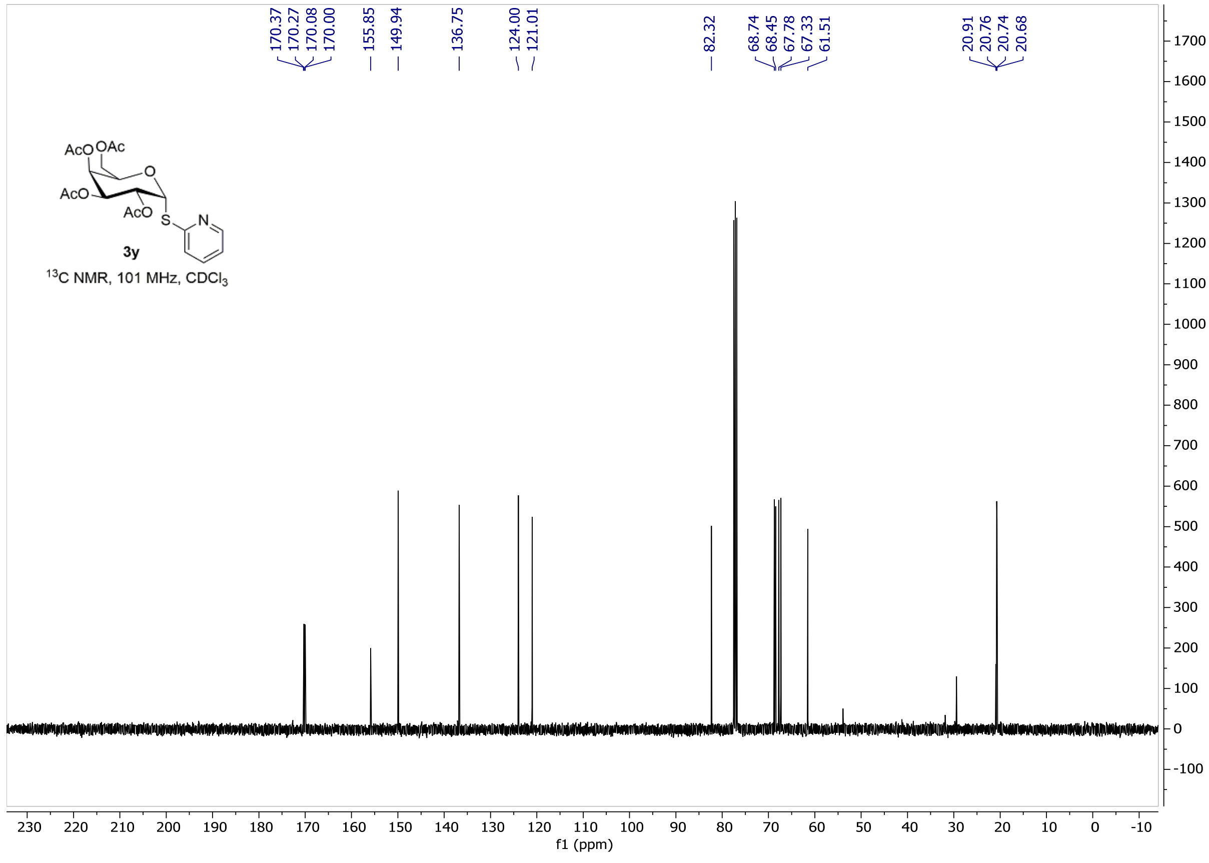
170.37 170.27 170.08 170.00
155.85
149.94
136.75
124.00 121.01
82.32
68.74 68.45 67.78 67.33 61.51
20.91 20.76 20.74 20.68
AcO OAc
O
AcO
AcO
S
N
3y
^{13}C NMR, 101 MHz, CDCl$_3$
f1 (ppm)

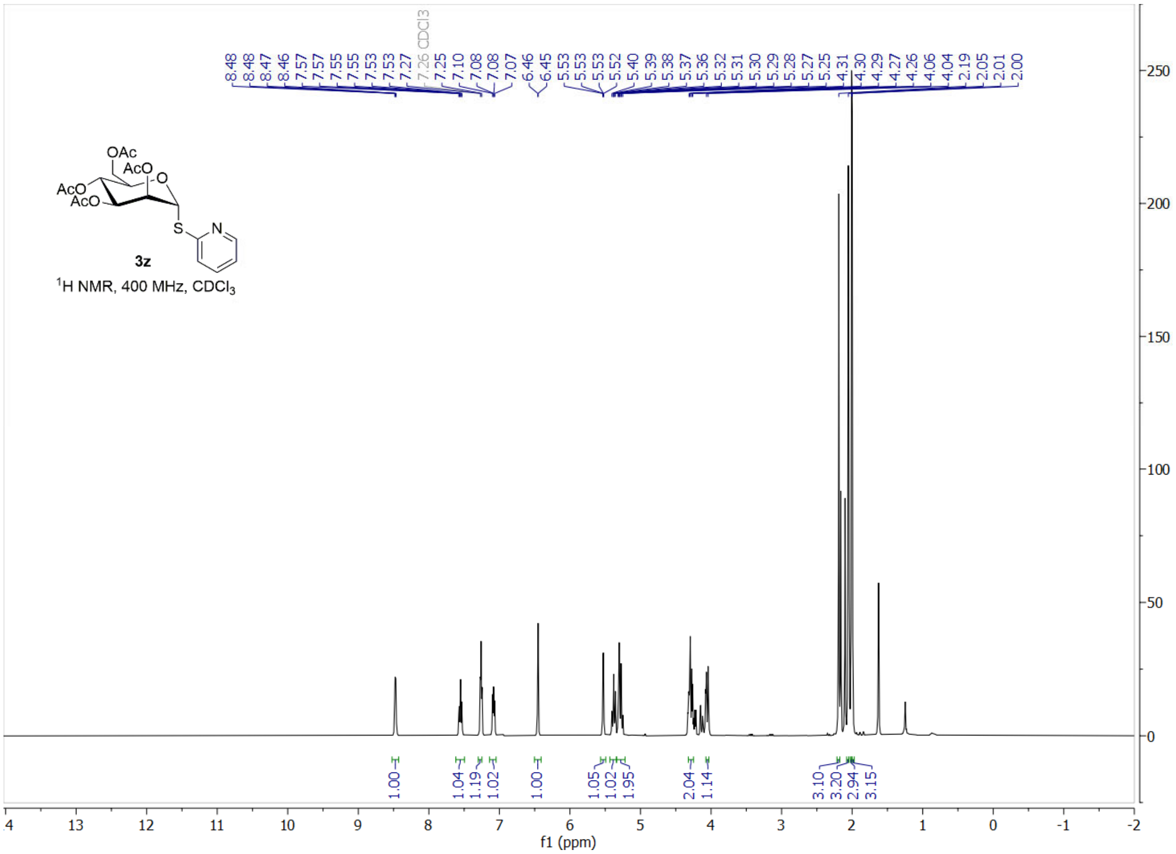

3z
^{1}H NMR, 400 MHz, CDCl$_3$
f1 (ppm)

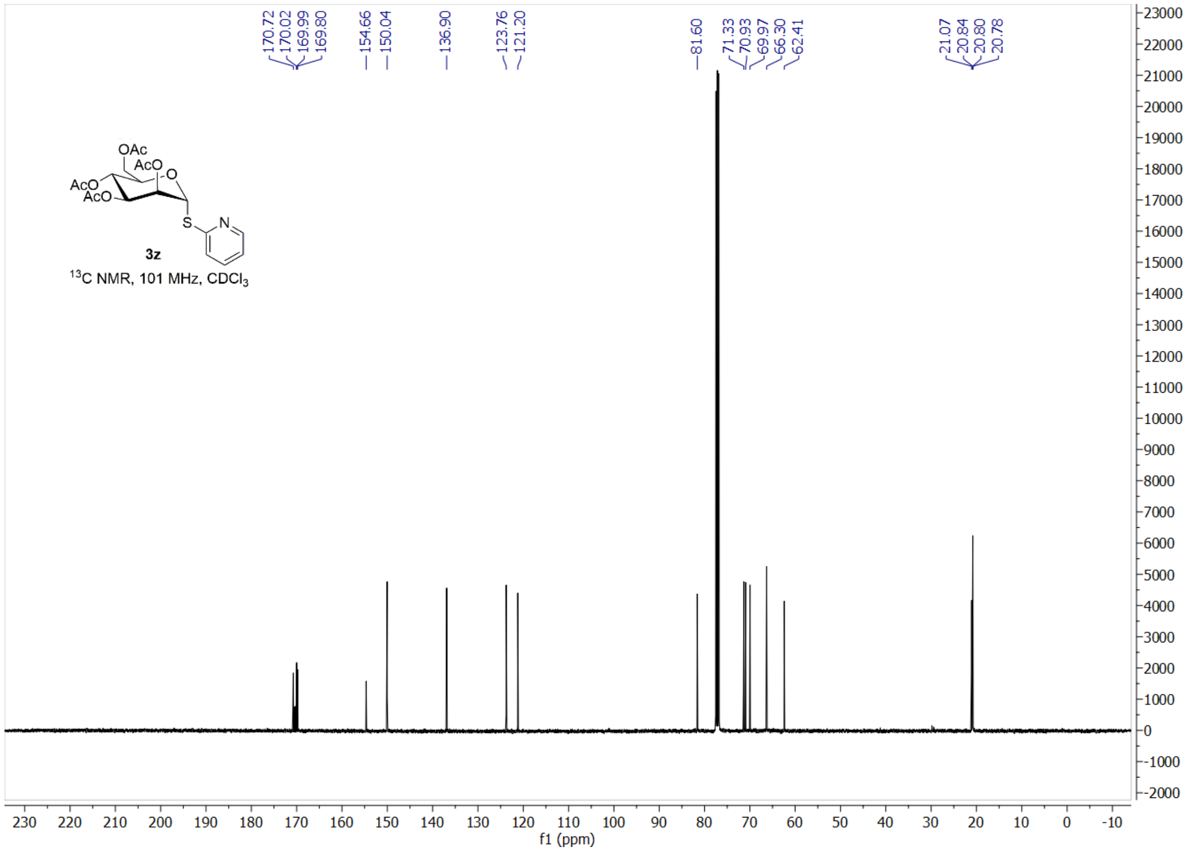

3z
^{13}C NMR, 101 MHz, CDCl$_3$
f1 (ppm)

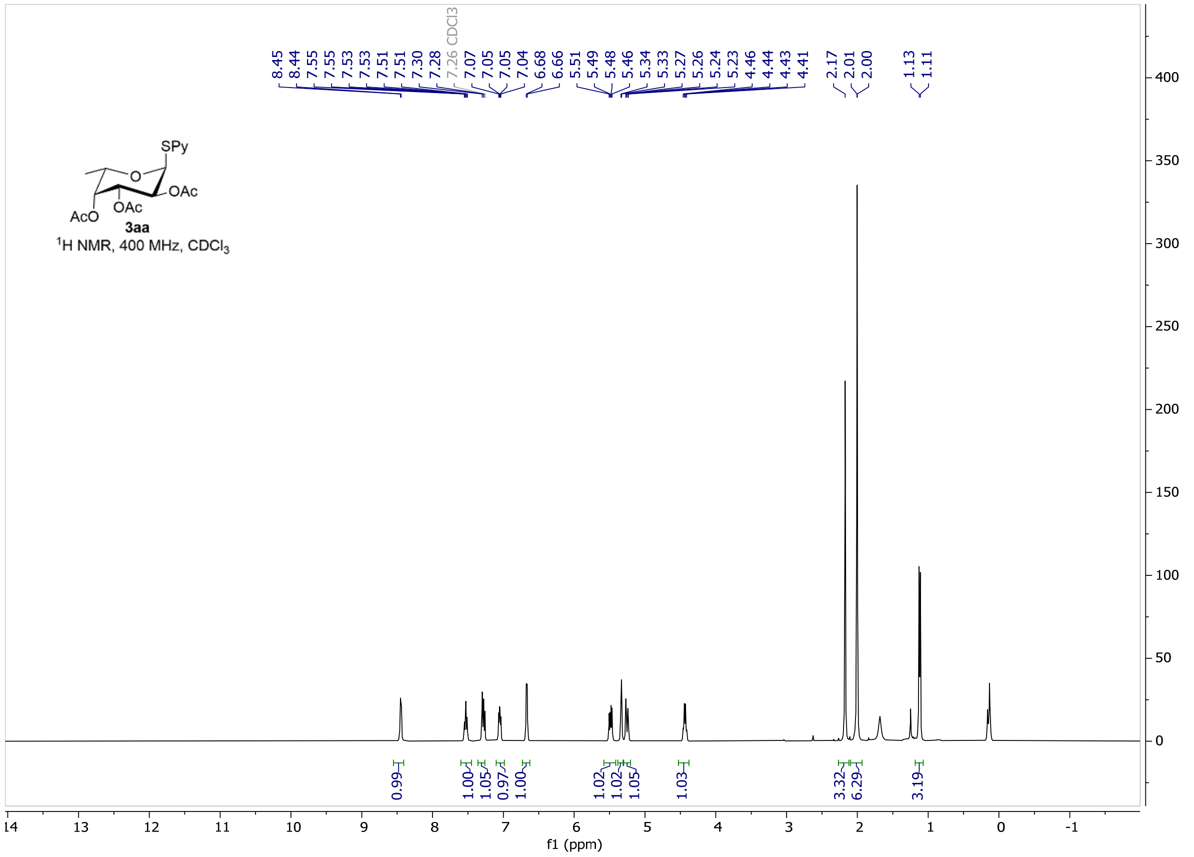

8.45
8.44
7.55
7.55
7.53
7.53
7.51
7.51
7.30
7.28
7.26 CDCl3
7.07
7.05
7.05
7.04
6.68
6.66
5.51
5.49
5.48
5.46
5.34
5.33
5.27
5.26
5.24
5.23
4.46
4.44
4.43
4.41
2.17
2.01
2.00
1.13
1.11
SPy
O
OAc
OAc
AcO
3aa
^{1}H NMR, 400 MHz, CDCl$_3$
0.99
1.00
1.05
0.97
1.00
1.02
1.02
1.05
1.03
3.32
6.29
3.19
f1 (ppm)

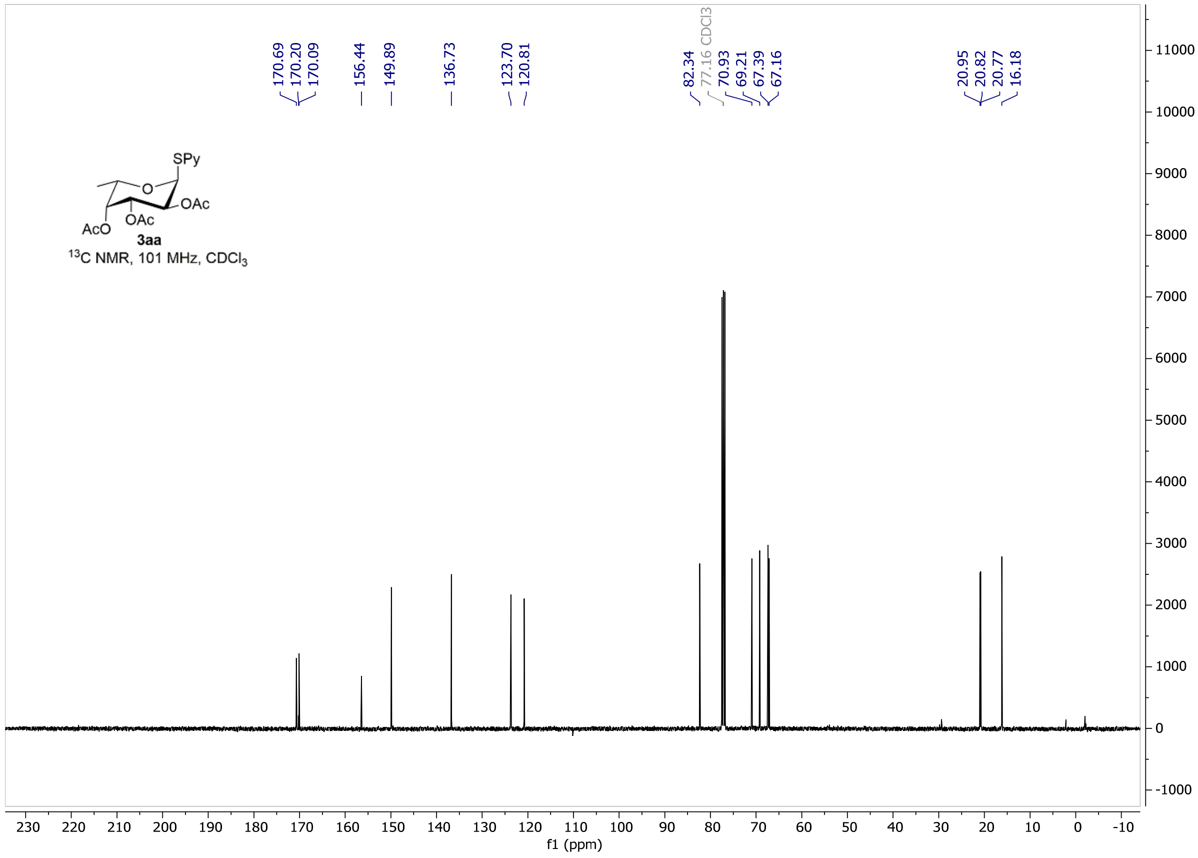

170.69
170.20
170.09
156.44
149.89
136.73
123.70
120.81
82.34
77.16 CDCl3
70.93
69.21
67.39
67.16
20.95
20.82
20.77
16.18
SPy
O
OAc
OAc
AcO
3aa
^{13}C NMR, 101 MHz, CDCl$_3$
f1 (ppm)

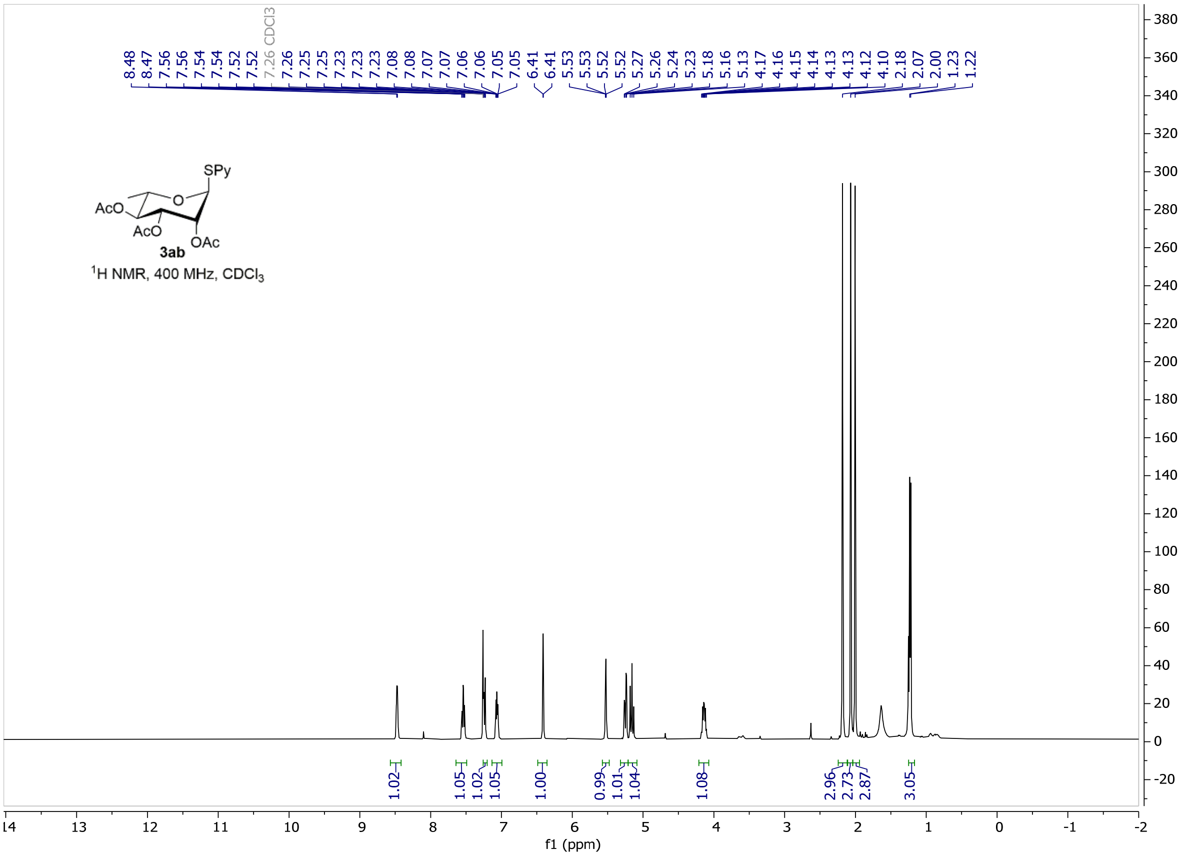

SPy
AcO
AcO
OAc
3ab
^{1}H NMR, 400 MHz, CDCl$_3$
f1 (ppm)

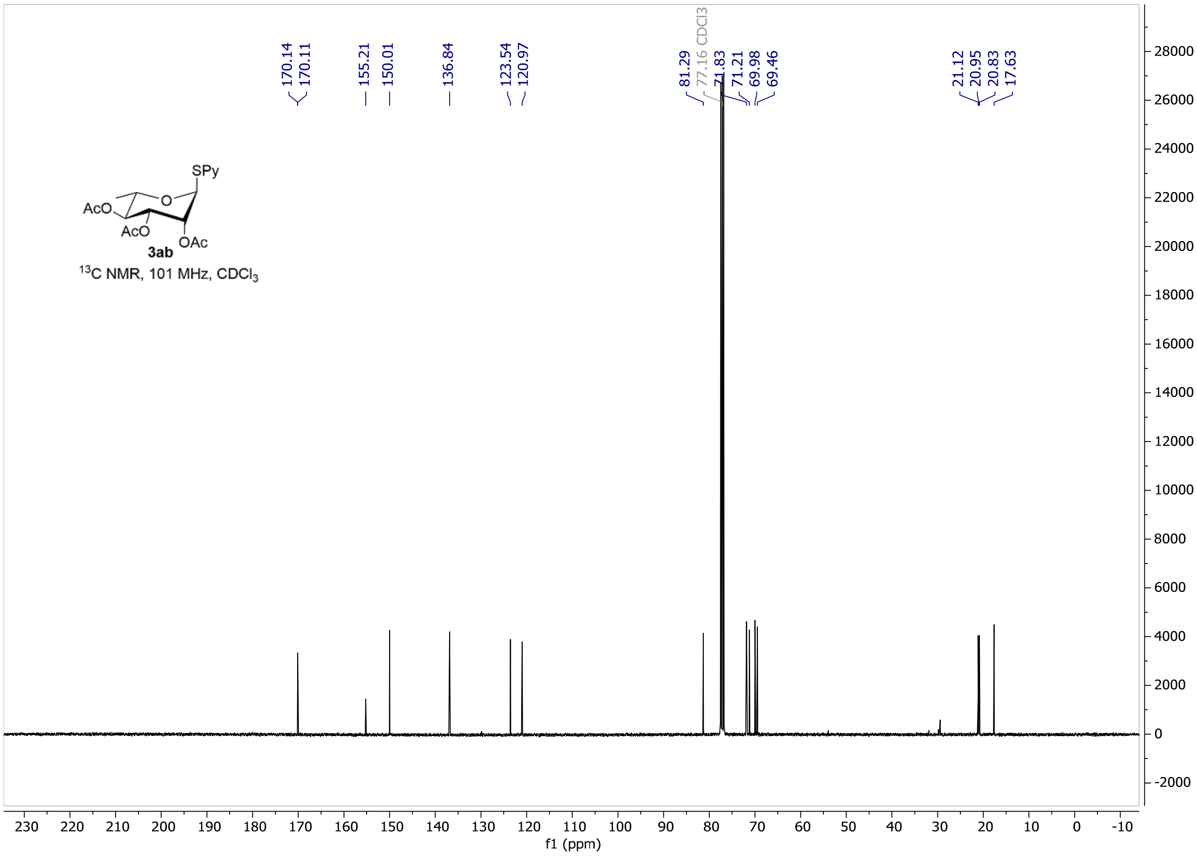

SPy
AcO
AcO
OAc
3ab
^{13}C NMR, 101 MHz, CDCl$_3$
f1 (ppm)

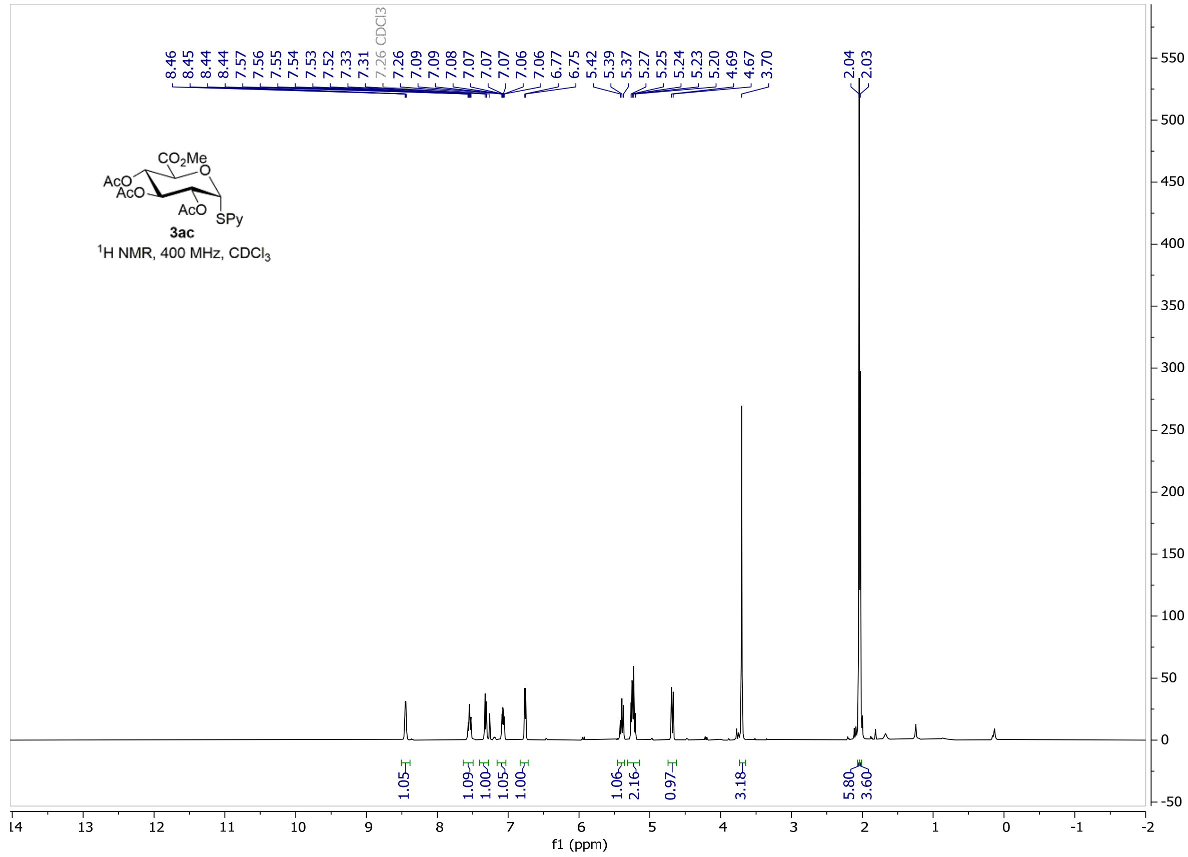
CO2Me
AcO
AcO
AcO
SPy
3ac
1H NMR, 400 MHz, CDCl3
f1 (ppm)

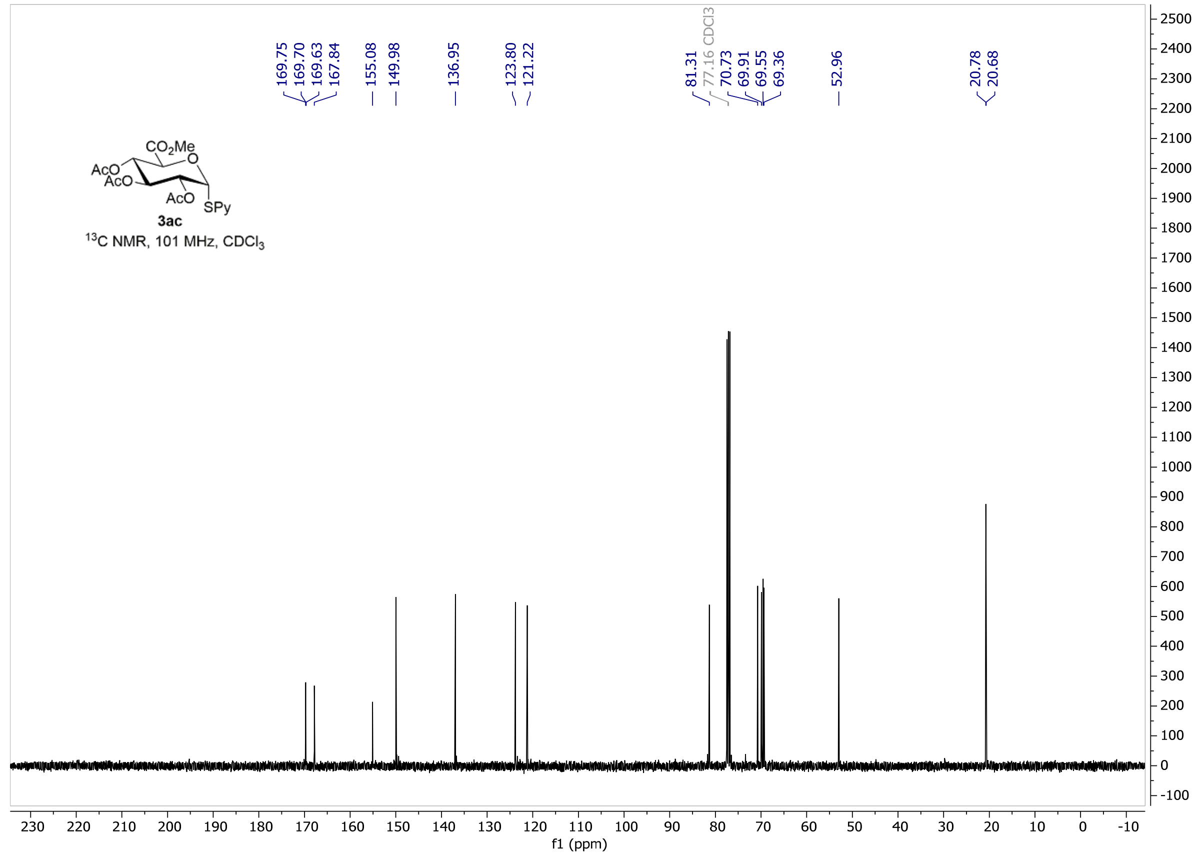
CO2Me
AcO
AcO
AcO
SPy
3ac
13C NMR, 101 MHz, CDCl3
f1 (ppm)

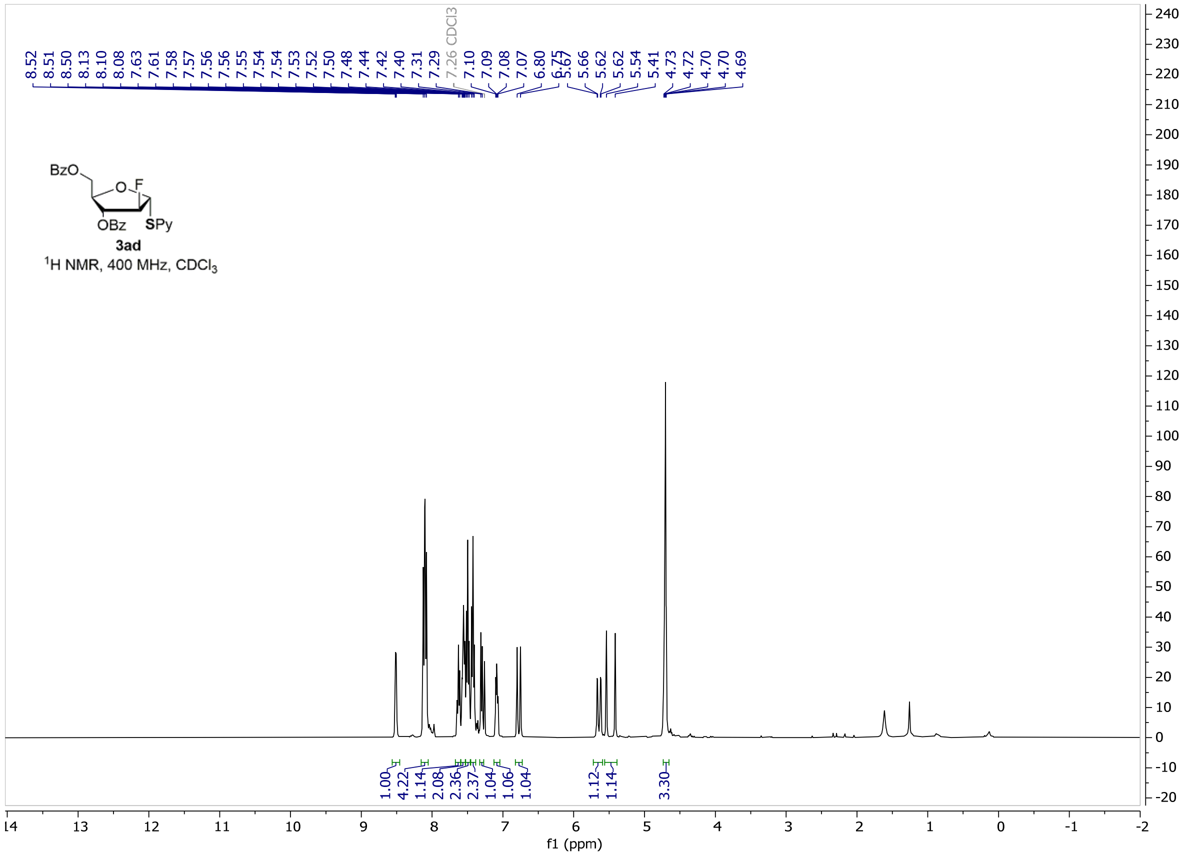

8.52
8.51
8.50
8.13
8.10
8.08
7.63
7.61
7.58
7.57
7.56
7.56
7.55
7.54
7.54
7.53
7.52
7.50
7.48
7.44
7.42
7.40
7.31
7.29
7.26 CDCl3
7.10
7.09
7.08
7.07
6.80
6.75
5.67
5.66
5.62
5.62
5.54
5.41
4.73
4.72
4.70
4.70
4.69
BzO
O
F
OBz
SPy
3ad
¹H NMR, 400 MHz, CDCl₃
1.00
4.22
1.14
2.08
2.36
2.37
1.04
1.06
1.04
1.12
1.14
3.30
f1 (ppm)

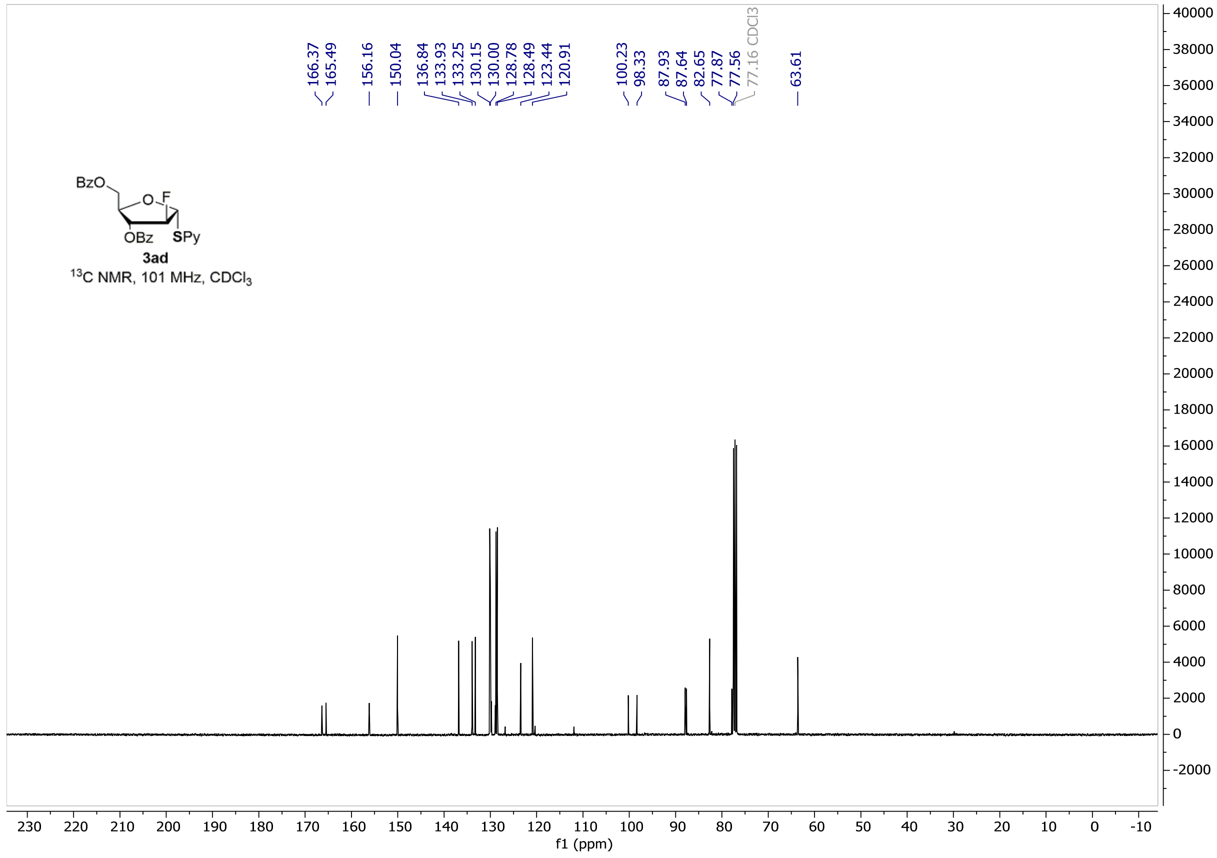

166.37
165.49
156.16
150.04
136.84
133.93
133.25
130.15
130.00
128.78
128.49
123.44
120.91
100.23
98.33
87.93
87.64
82.65
77.87
77.56
77.16 CDCl3
63.61
BzO
O
F
OBz
SPy
3ad
¹³C NMR, 101 MHz, CDCl₃
f1 (ppm)

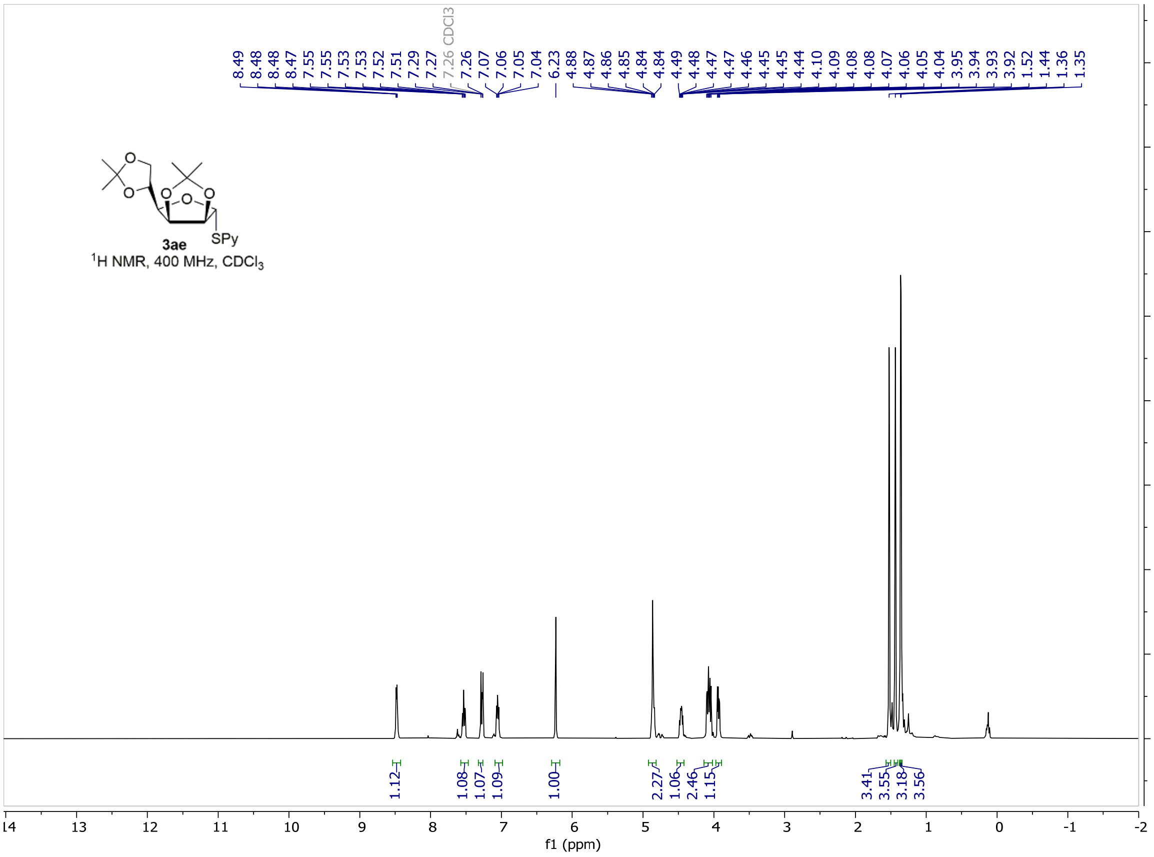

8.49 8.48 8.48 8.47 7.55 7.55 7.53 7.53 7.52 7.51 7.29 7.27 7.26 CDCl3 7.26 7.07 7.06 7.05 7.04 6.23 4.88 4.87 4.86 4.85 4.84 4.84 4.49 4.48 4.47 4.47 4.46 4.45 4.45 4.44 4.10 4.09 4.08 4.08 4.07 4.06 4.05 4.04 3.95 3.94 3.93 3.92 1.52 1.44 1.36 1.35
SPy
3ae
^{1}H NMR, 400 MHz, $CDCl_3$
1.12 1.08 1.07 1.09 1.00 2.27 1.06 2.46 1.15 3.41 3.55 3.18 3.56
f1 (ppm)

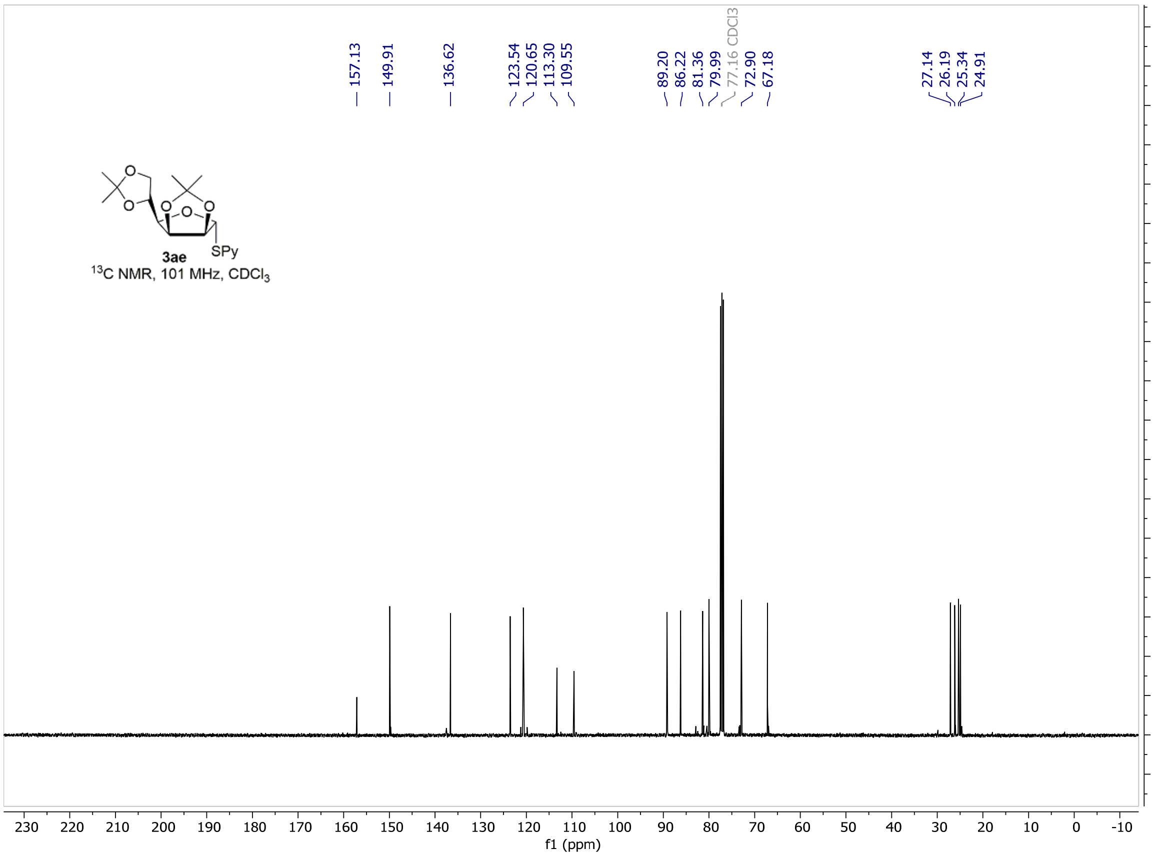

157.13 149.91 136.62 123.54 120.65 113.30 109.55 89.20 86.22 81.36 79.99 77.16 CDCl3 72.90 67.18 27.14 26.19 25.34 24.91
SPy
3ae
^{13}C NMR, 101 MHz, $CDCl_3$
f1 (ppm)

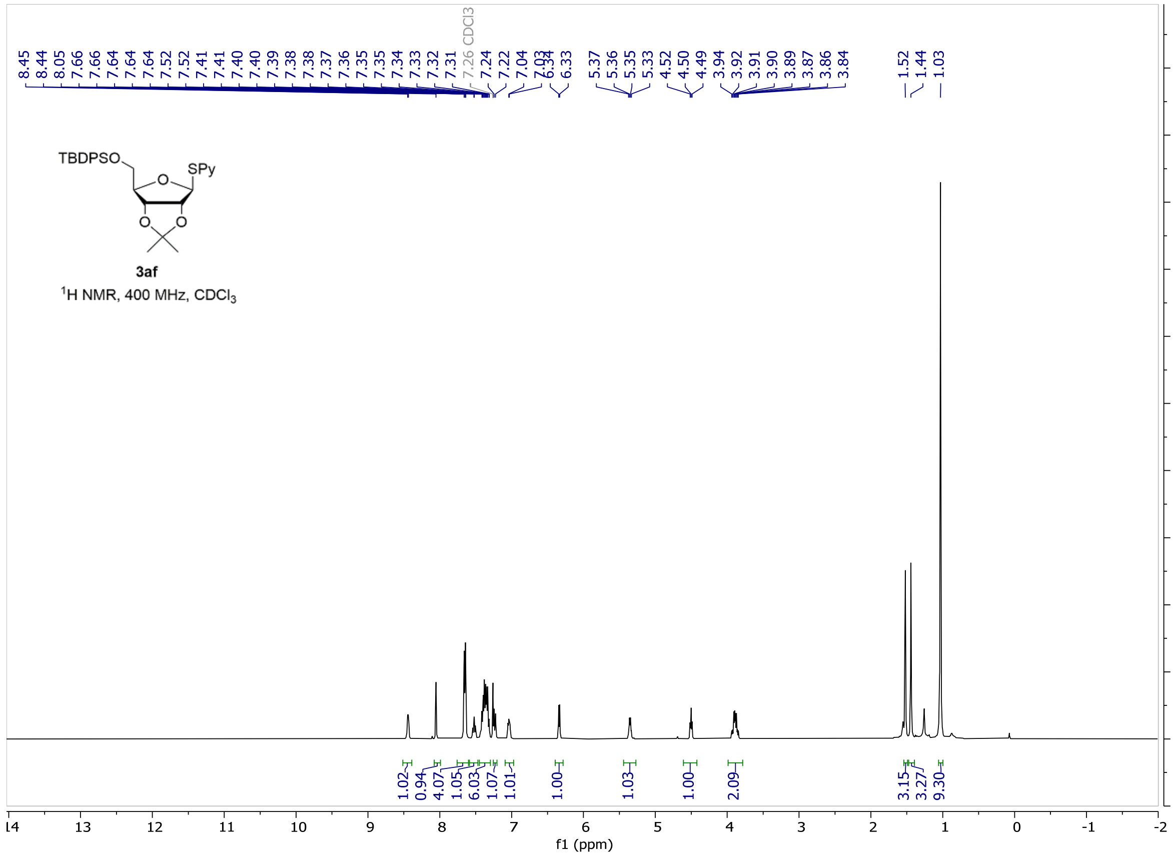
TBDPSO
SPy
3af
^{1}H NMR, 400 MHz, $CDCl_3$
f1 (ppm)

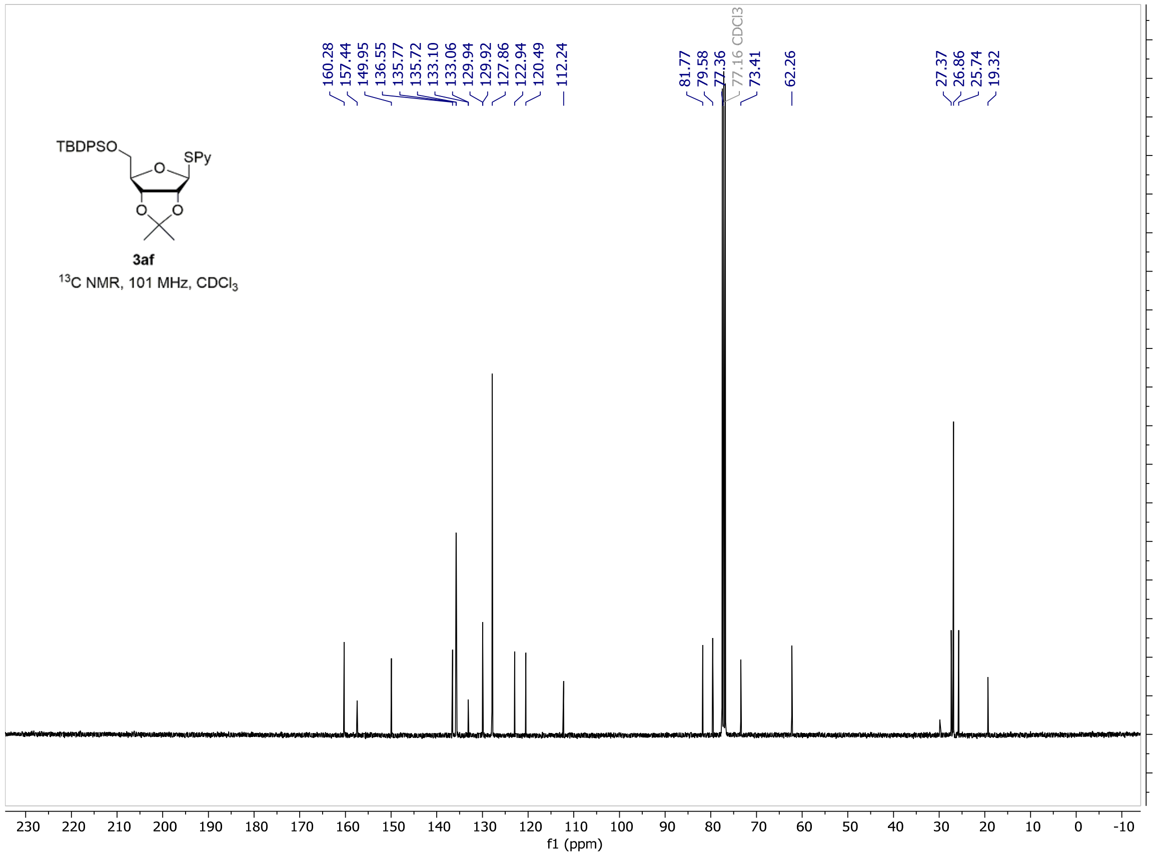
TBDPSO
SPy
3af
^{13}C NMR, 101 MHz, $CDCl_3$
f1 (ppm)

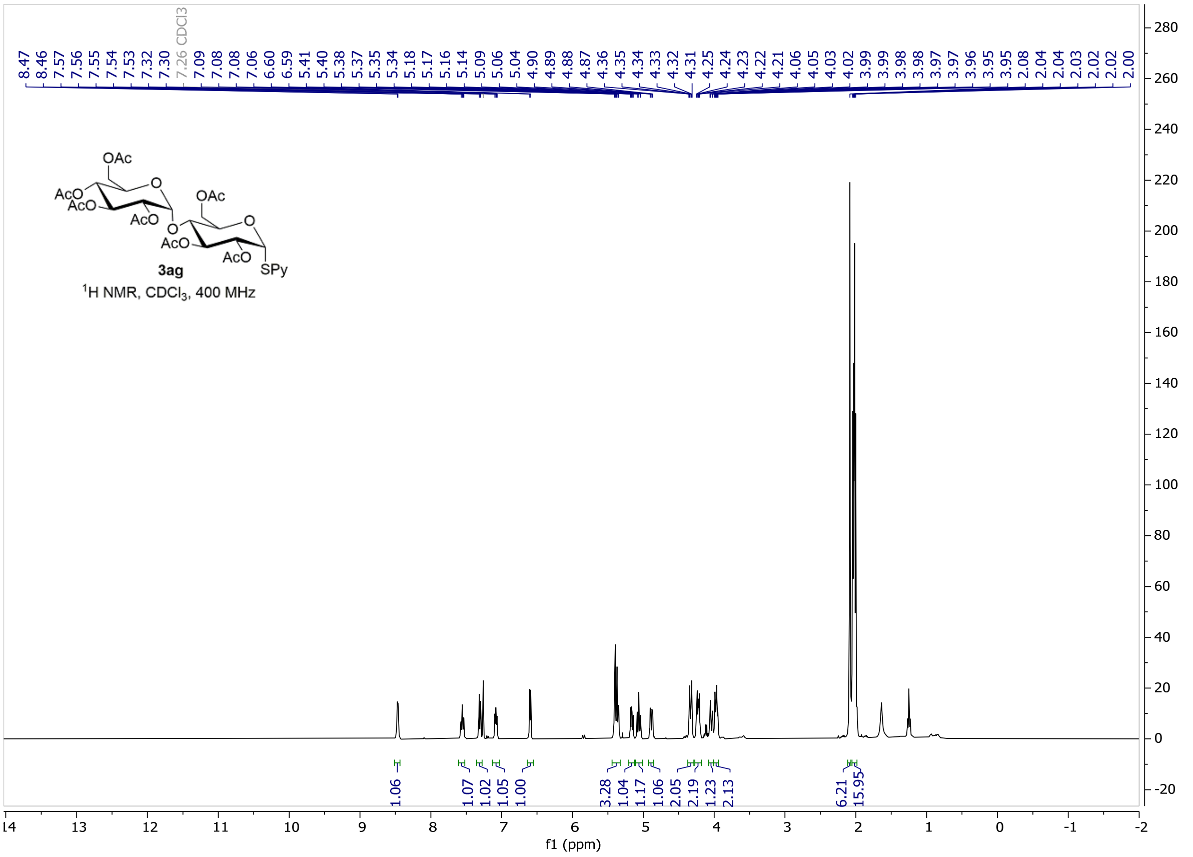

3ag
^{1}H NMR, CDCl$_3$, 400 MHz
f1 (ppm)

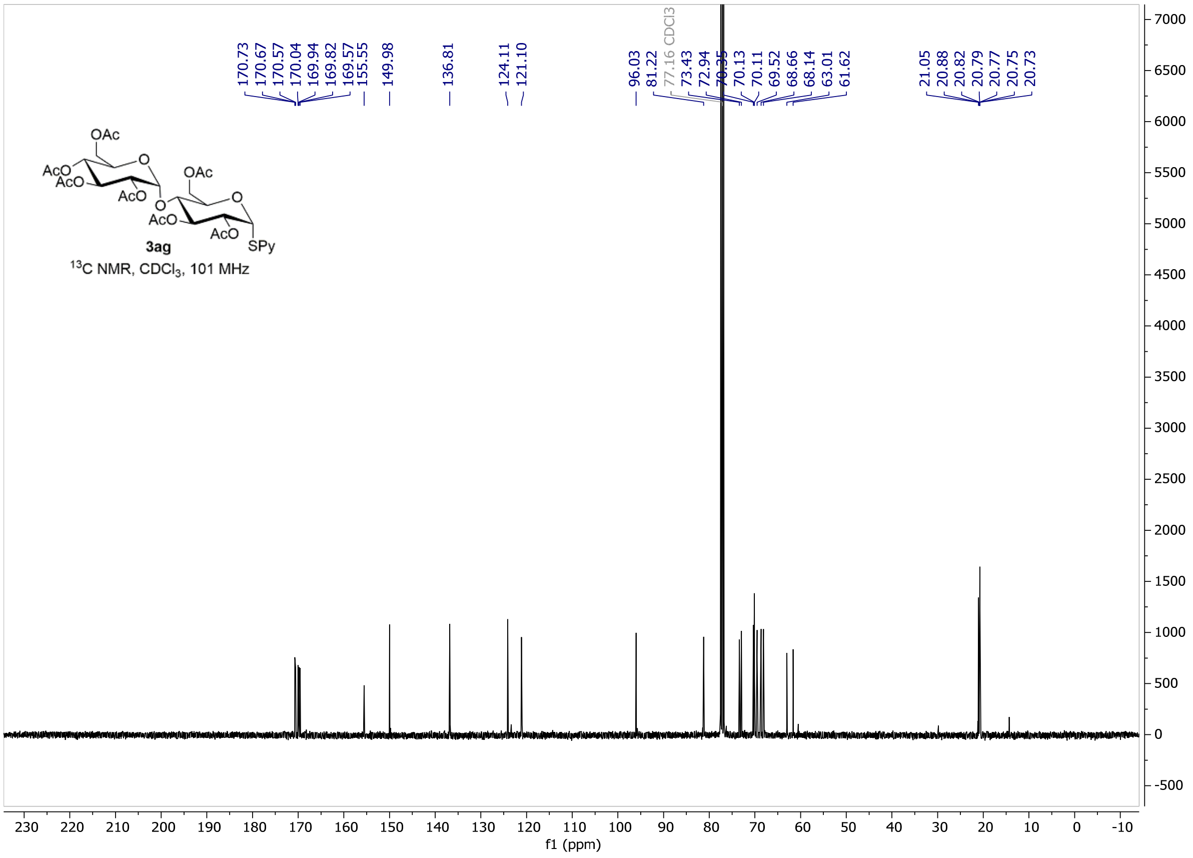

3ag
^{13}C NMR, CDCl$_3$, 101 MHz
f1 (ppm)

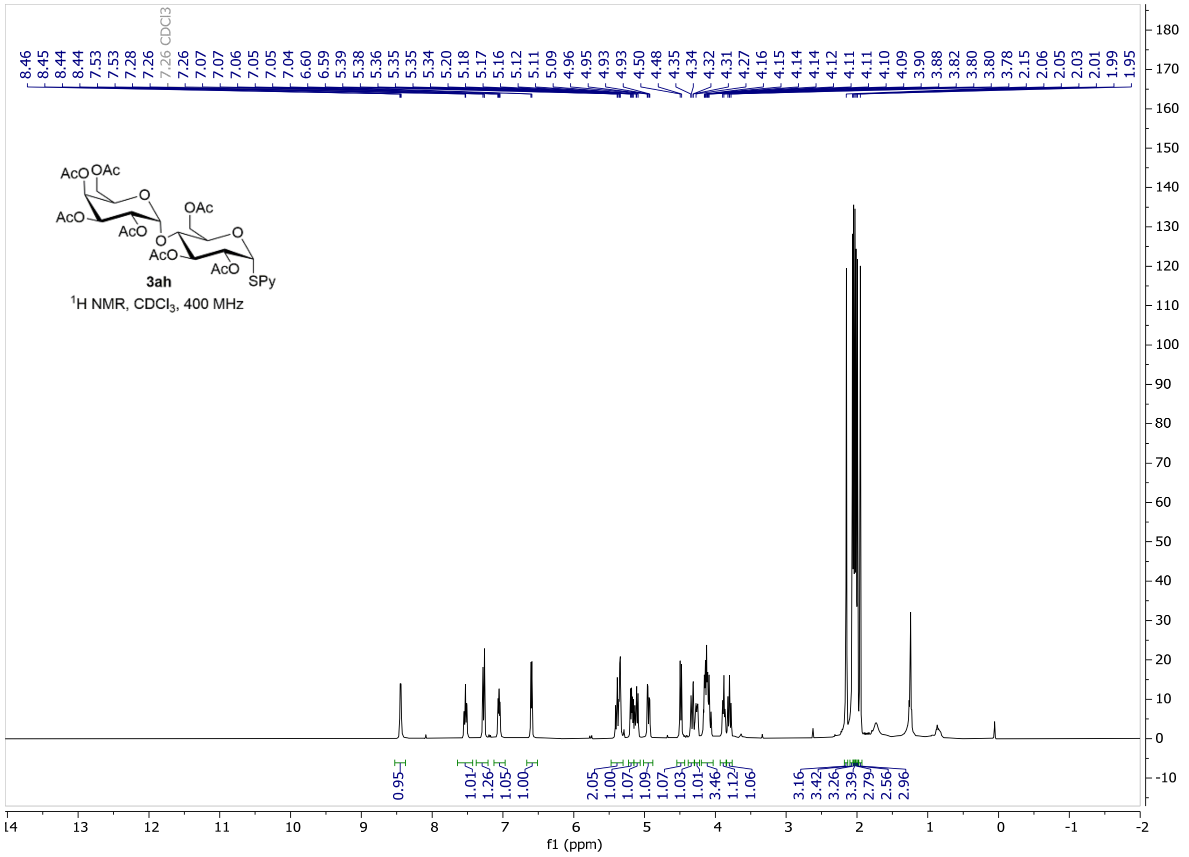
3ah
^{1}H NMR, CDCl$_3$, 400 MHz
f1 (ppm)

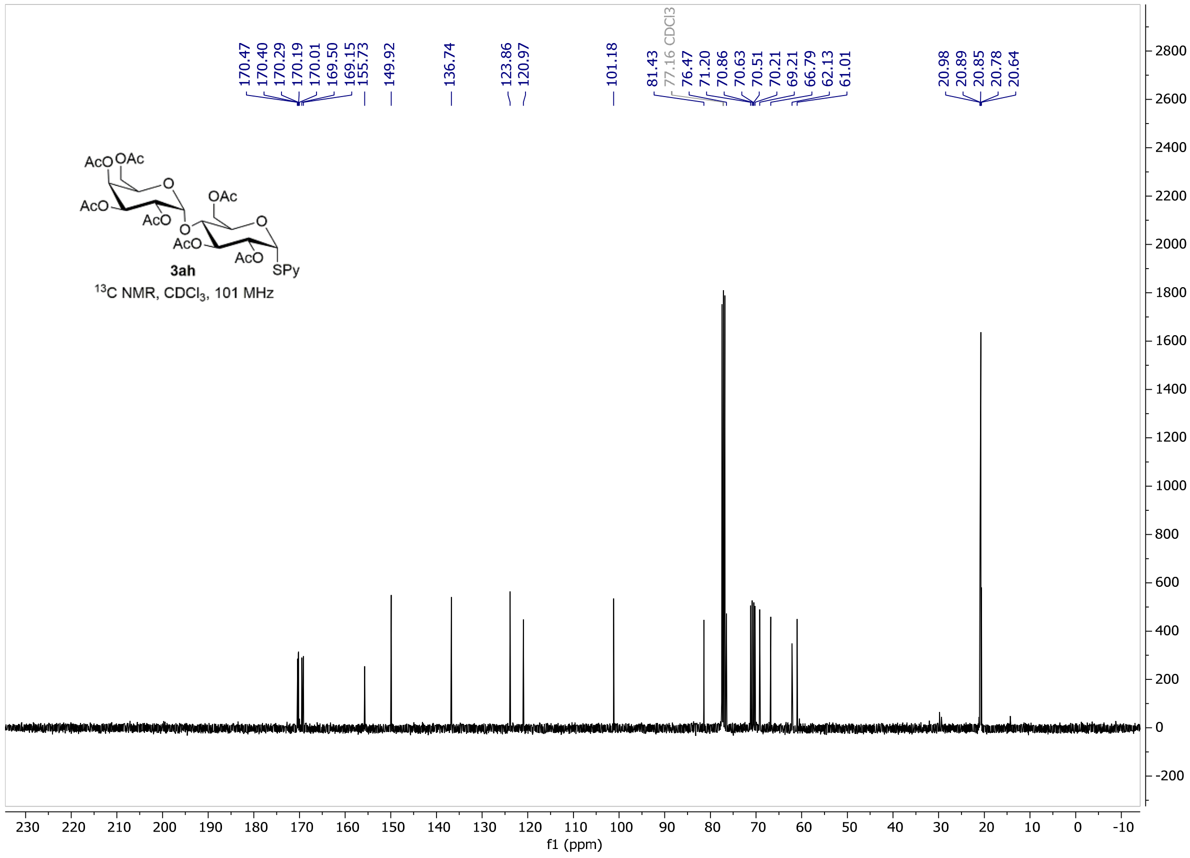
3ah
^{13}C NMR, CDCl$_3$, 101 MHz
f1 (ppm)

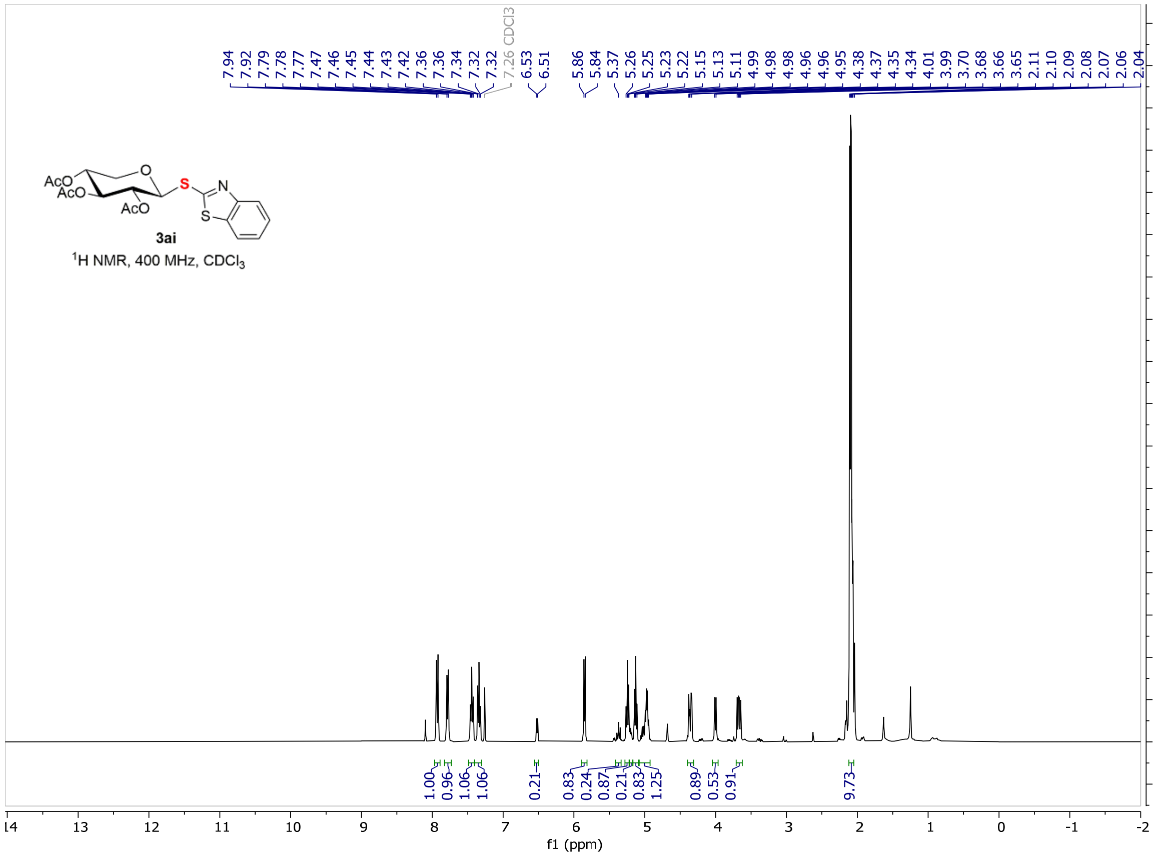

3ai
¹H NMR, 400 MHz, CDCl3
7.26 CDCl3
f1 (ppm)

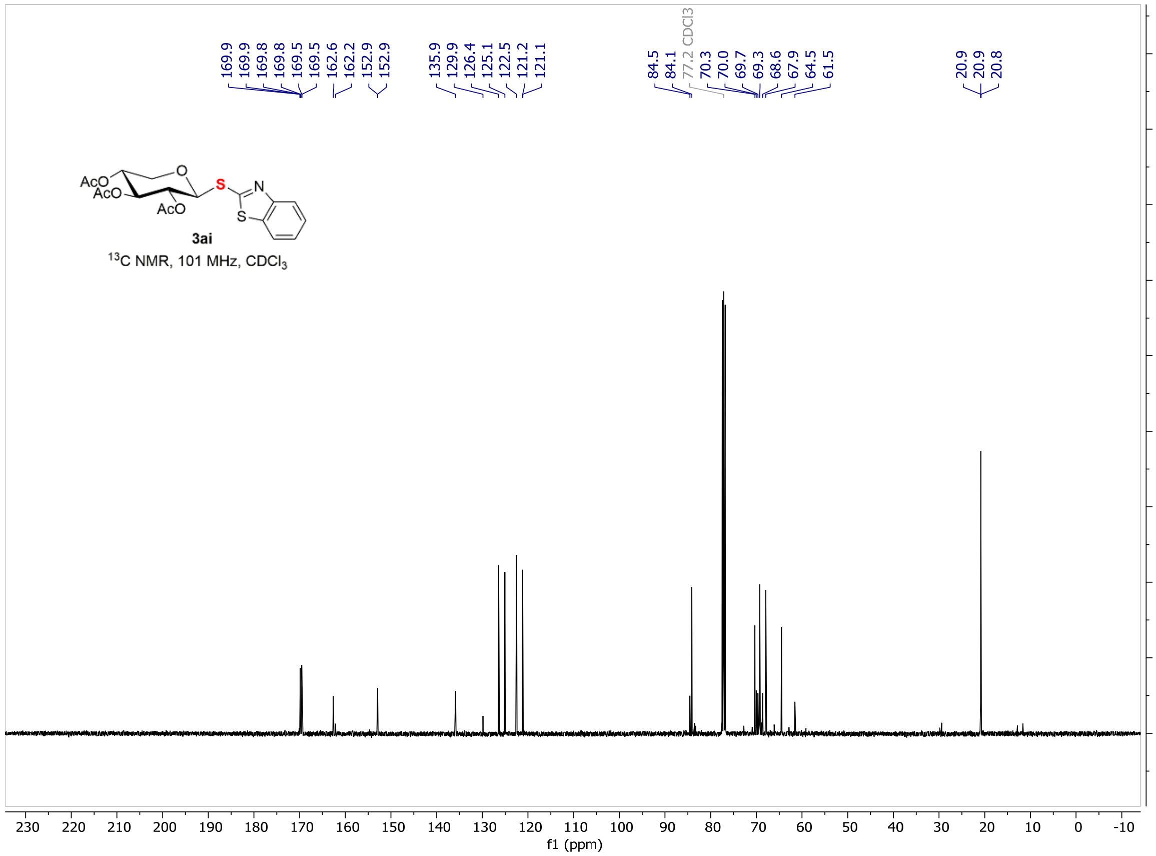

3ai
¹³C NMR, 101 MHz, CDCl3
77.2 CDCl3
f1 (ppm)

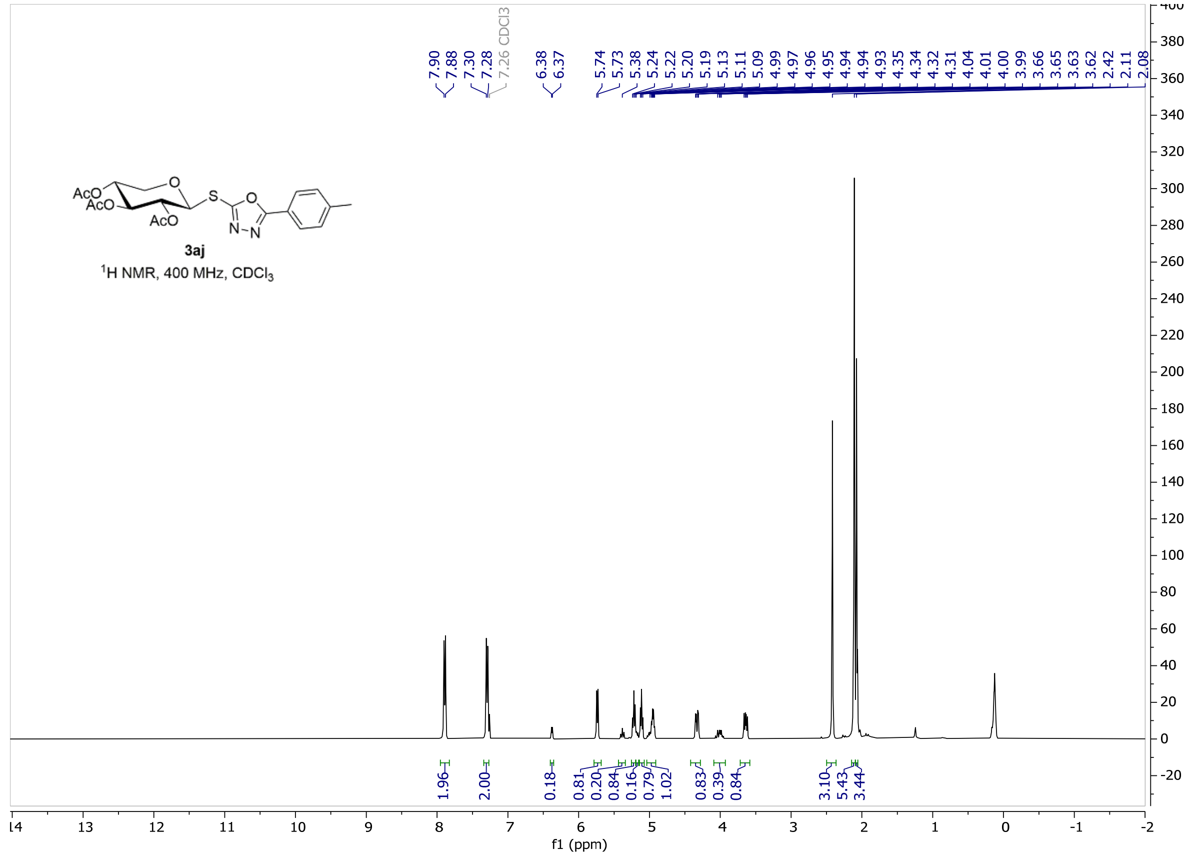

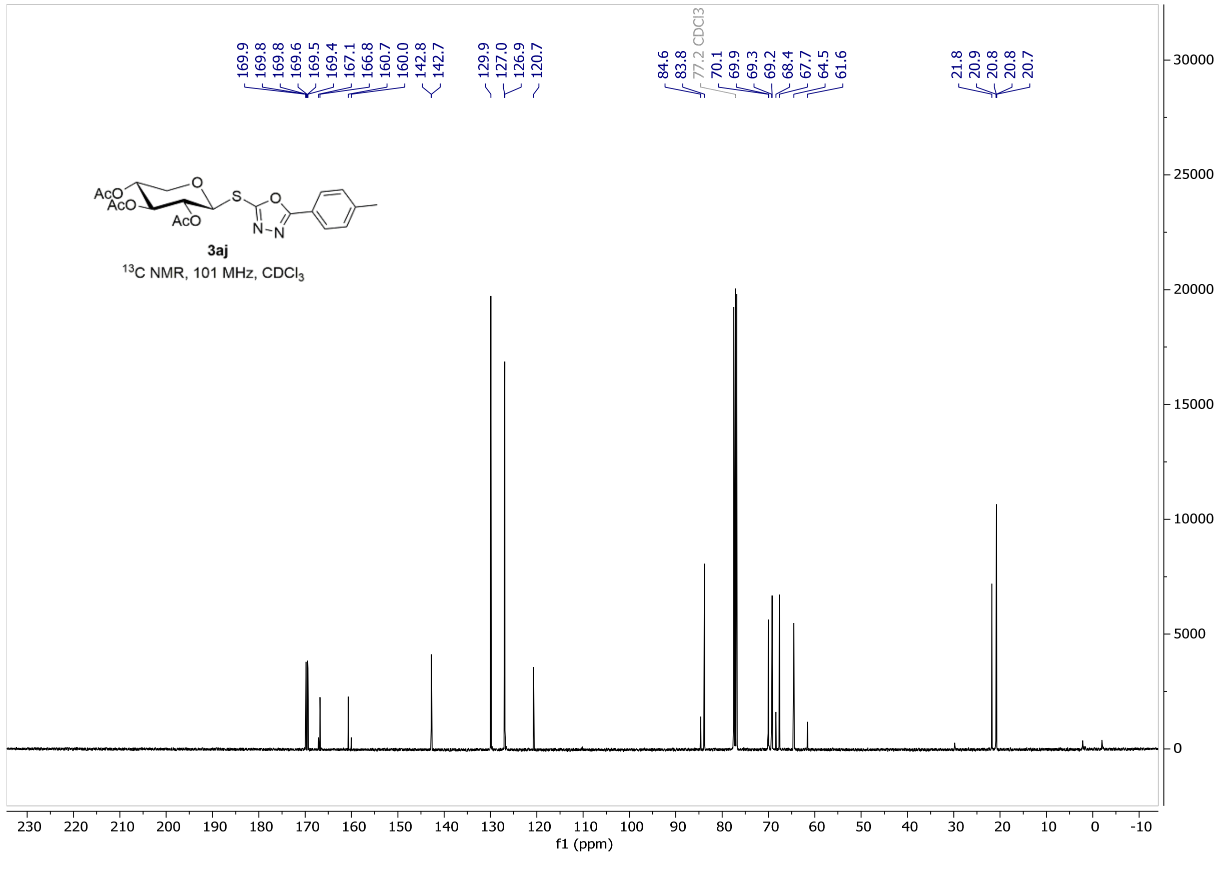

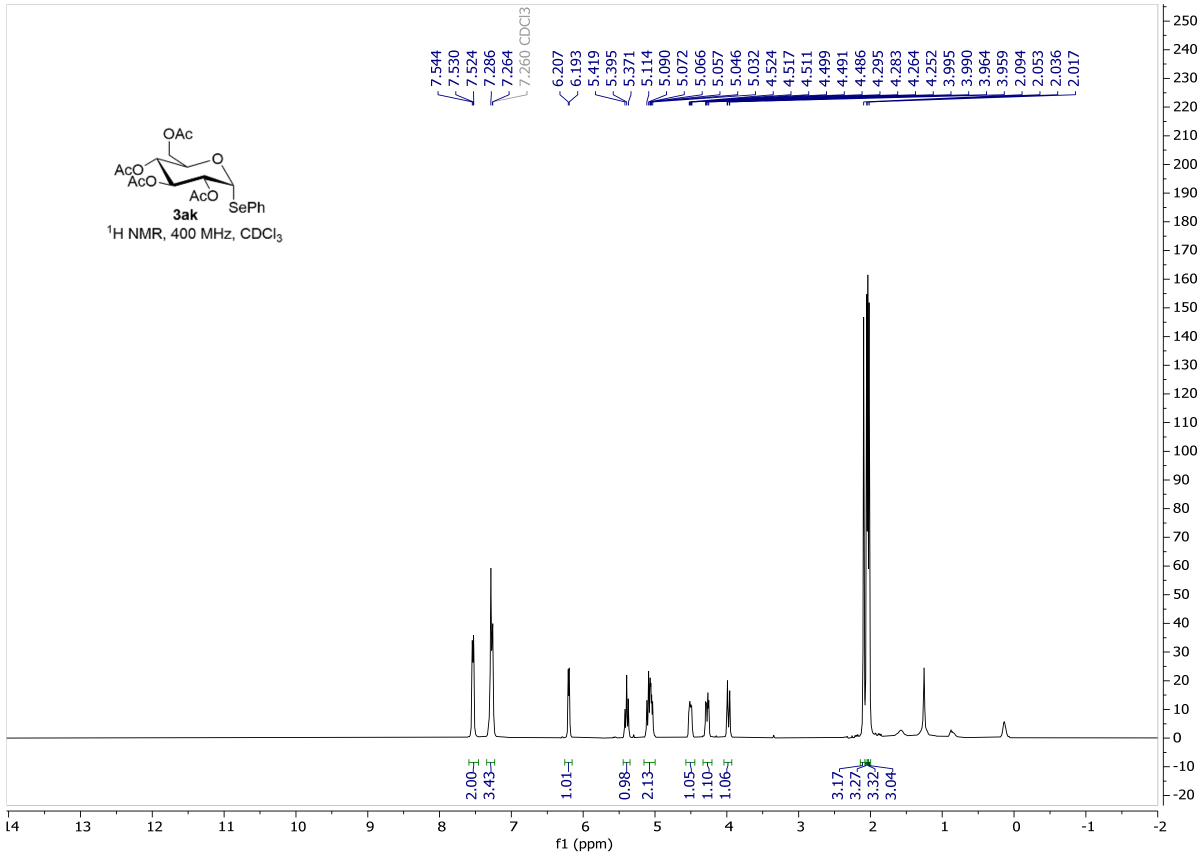

OAc
AcO
AcO
AcO
SePh
3ak
1H NMR, 400 MHz, CDCl3
7.544
7.530
7.524
7.286
7.264
7.260 CDCl3
6.207
6.193
5.419
5.395
5.371
5.114
5.090
5.072
5.066
5.057
5.046
5.032
4.524
4.517
4.511
4.499
4.491
4.486
4.295
4.283
4.264
4.252
3.995
3.990
3.964
3.959
2.094
2.053
2.036
2.017
2.00
3.43
1.01
0.98
2.13
1.05
1.10
1.06
3.17
3.27
3.32
3.04
f1 (ppm)

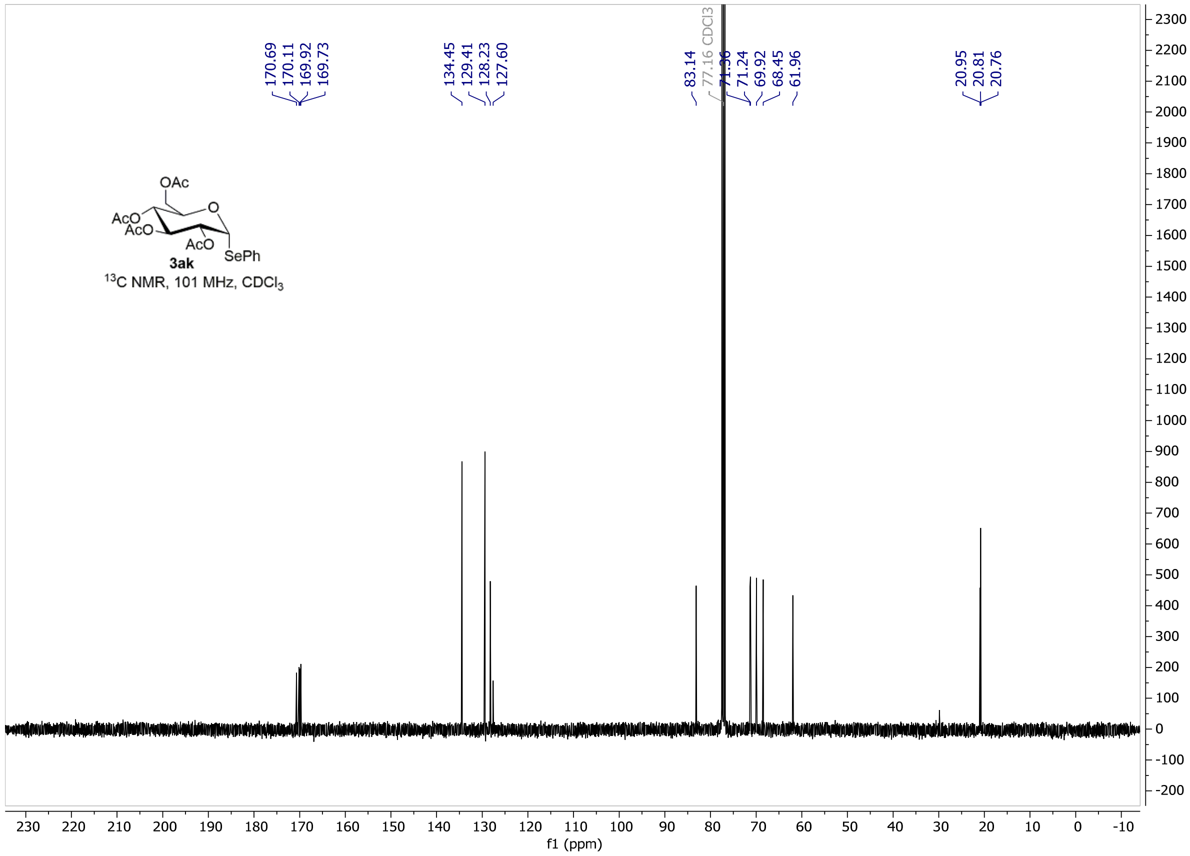

OAc
AcO
AcO
AcO
SePh
3ak
13C NMR, 101 MHz, CDCl3
170.69
170.11
169.92
169.73
134.45
129.41
128.23
127.60
83.14
77.16 CDCl3
71.36
71.24
69.92
68.45
61.96
20.95
20.81
20.76
f1 (ppm)

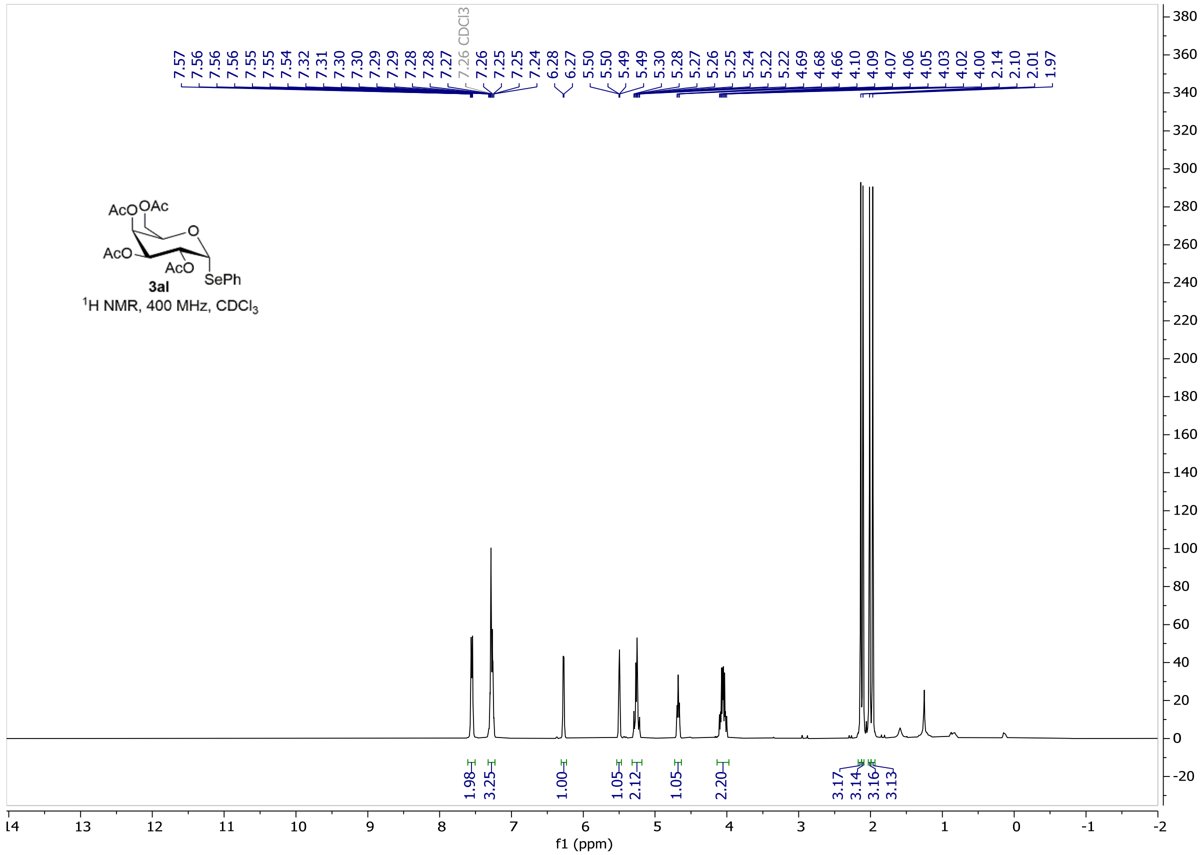
AcO OAc
AcO
AcO
SePh
O
3al
1H NMR, 400 MHz, CDCl3
7.57 7.56 7.56 7.55 7.55 7.54 7.32 7.31 7.30 7.29 7.29 7.28 7.28 7.27 7.26 CDCl3 7.26 7.25 7.25 7.24 6.28 6.27 5.50 5.49 5.49 5.30 5.28 5.27 5.26 5.25 5.24 5.22 5.22 4.69 4.68 4.66 4.10 4.09 4.07 4.06 4.05 4.03 4.02 4.00 2.14 2.10 2.01 1.97
1.98 3.25 1.00 1.05 2.12 1.05 2.20 3.17 3.14 3.16 3.13
f1 (ppm)

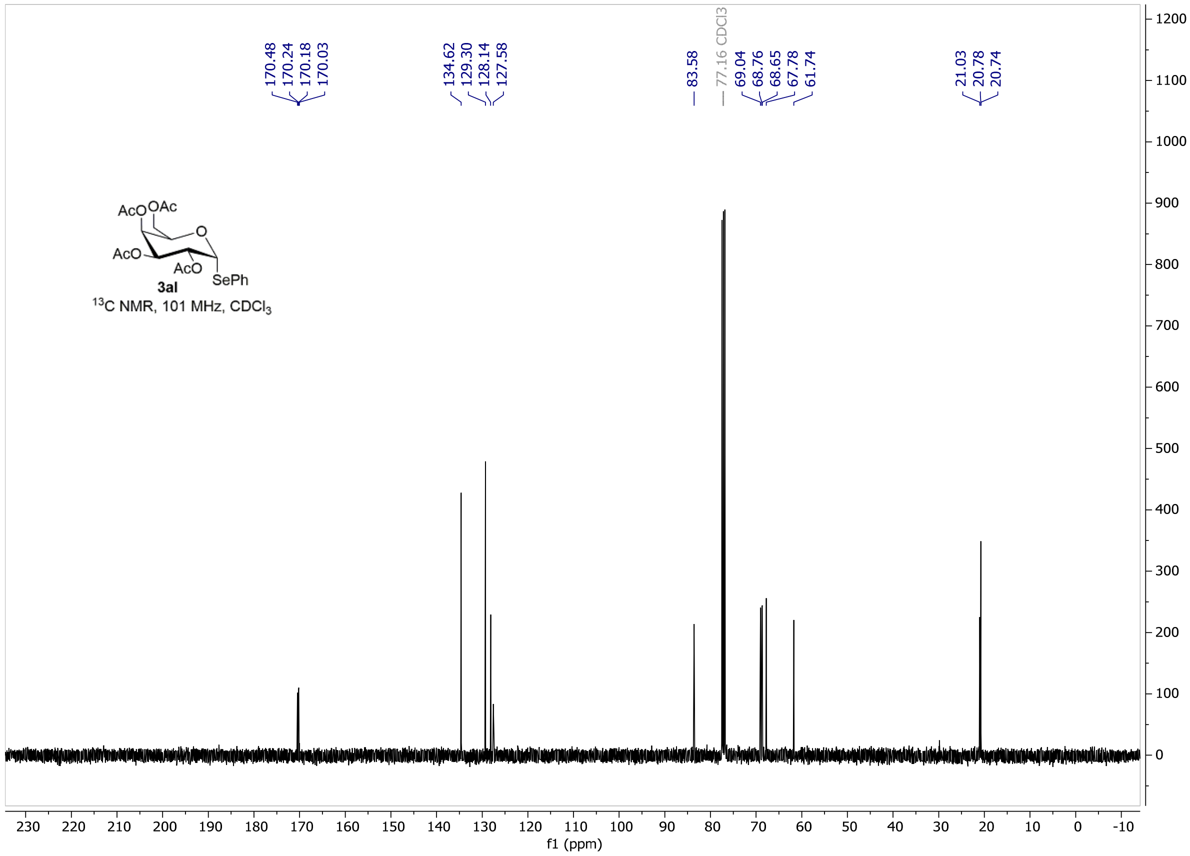
AcO OAc
AcO
AcO
SePh
O
3al
13C NMR, 101 MHz, CDCl3
170.48 170.24 170.18 170.03
134.62 129.30 128.14 127.58
83.58
77.16 CDCl3
69.04 68.76 68.65 67.78 61.74
21.03 20.78 20.74
f1 (ppm)

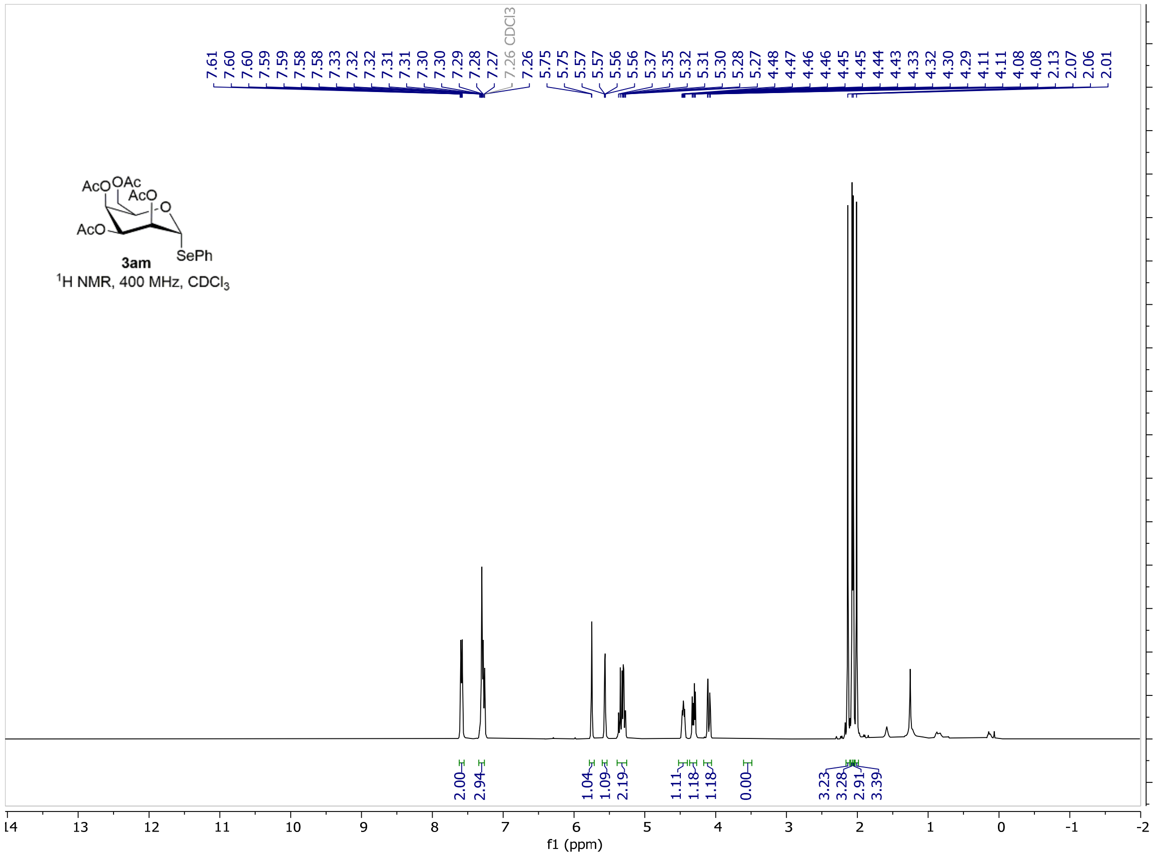

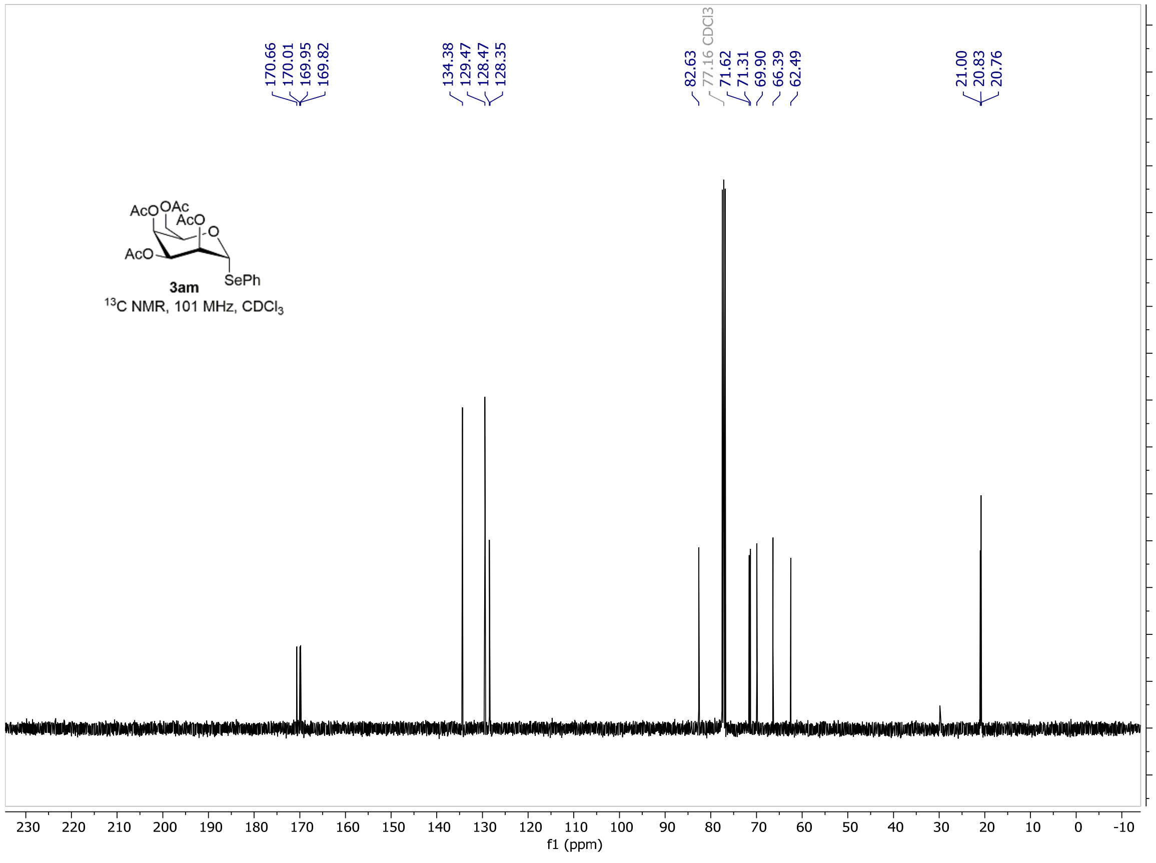

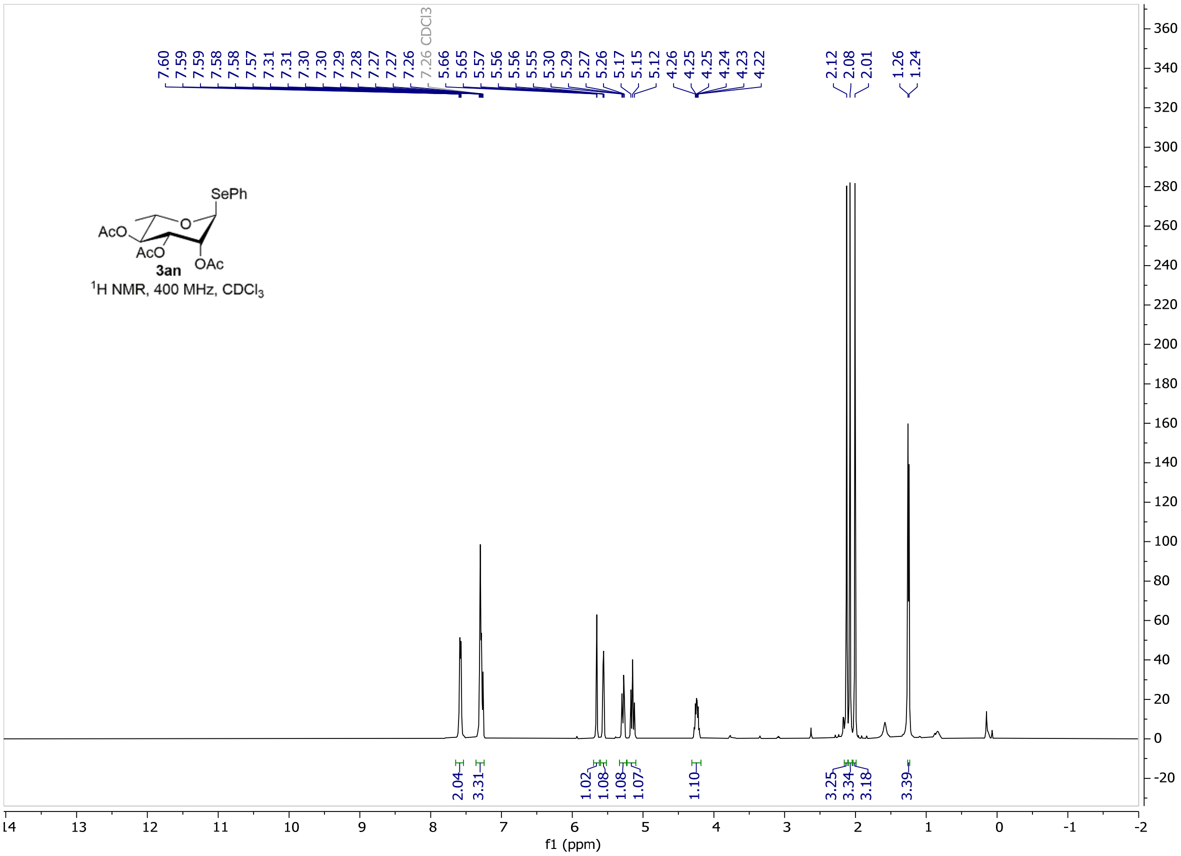
7.60
7.59
7.59
7.58
7.58
7.57
7.31
7.31
7.30
7.30
7.29
7.28
7.27
7.27
7.26
7.26 CDCl3
5.66
5.65
5.57
5.56
5.56
5.55
5.30
5.29
5.27
5.26
5.17
5.15
5.12
4.26
4.25
4.25
4.24
4.23
4.22
2.12
2.08
2.01
1.26
1.24
SePh
AcO
O
AcO
OAc
3an
^{1}H NMR, 400 MHz, CDCl$_3$
2.04
3.31
1.02
1.08
1.08
1.07
1.10
3.25
3.34
3.18
3.39
f1 (ppm)

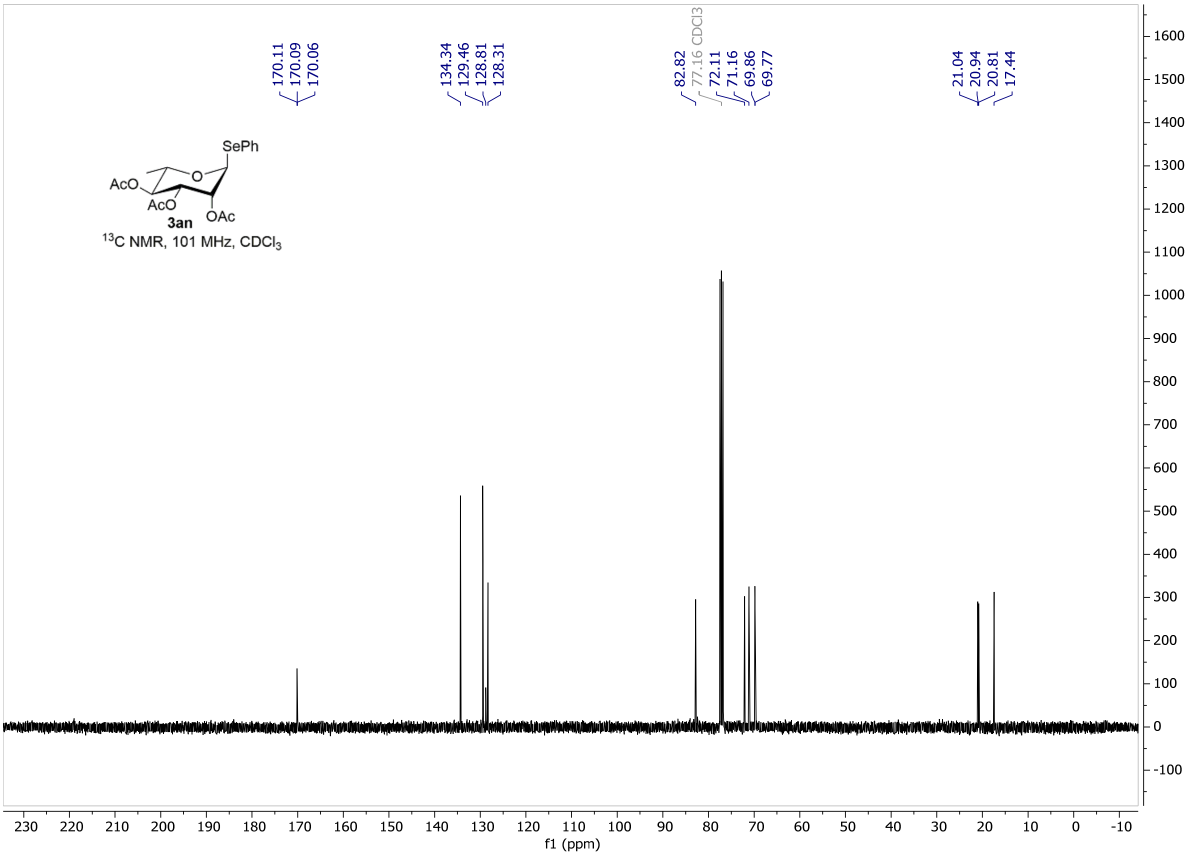
170.11
170.09
170.06
134.34
129.46
128.81
128.31
82.82
77.16 CDCl3
72.11
71.16
69.86
69.77
21.04
20.94
20.81
17.44
SePh
AcO
O
AcO
OAc
3an
^{13}C NMR, 101 MHz, CDCl$_3$
f1 (ppm)

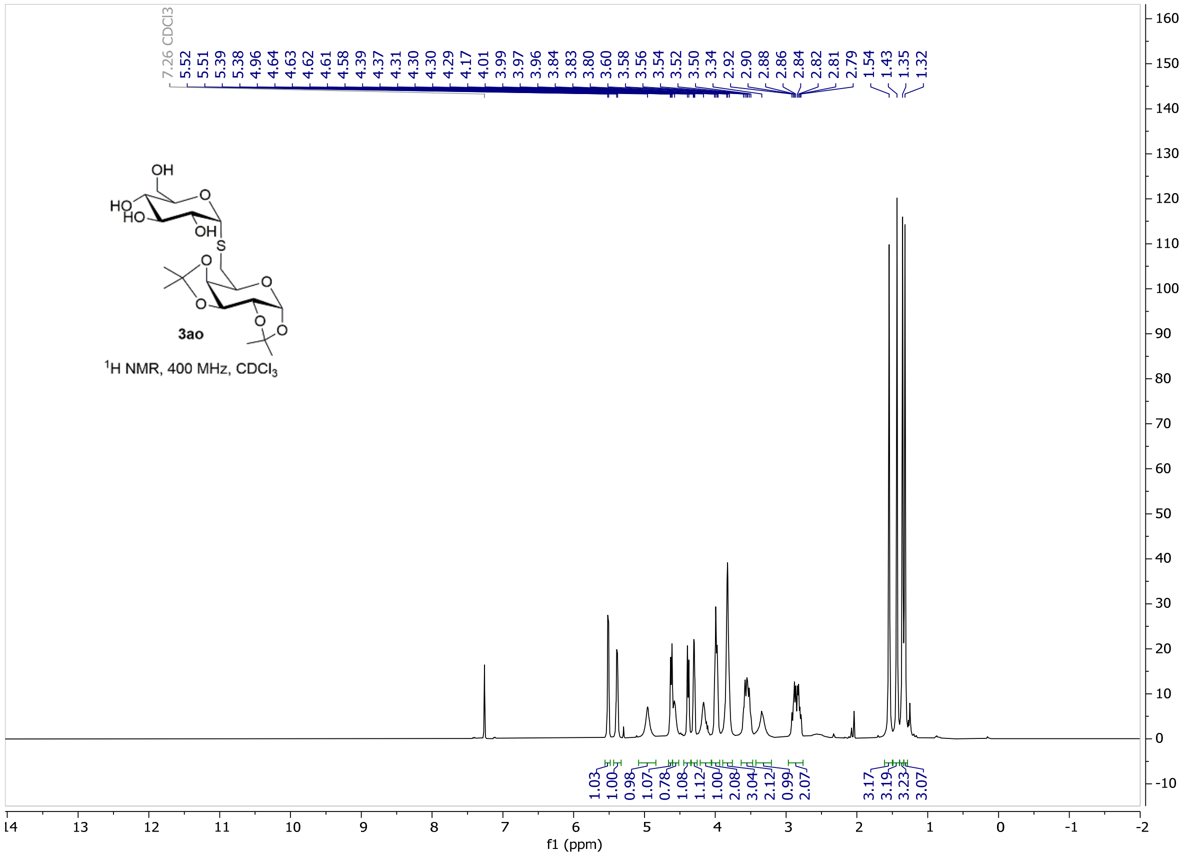

7.26 CDCl3
5.52
5.51
5.39
5.38
4.96
4.64
4.63
4.62
4.61
4.58
4.39
4.37
4.31
4.30
4.29
4.17
4.01
3.99
3.97
3.96
3.84
3.83
3.80
3.60
3.58
3.56
3.54
3.52
3.50
3.34
2.92
2.90
2.88
2.86
2.84
2.82
2.81
2.79
1.54
1.43
1.35
1.32
3ao
^{1}H NMR, 400 MHz, $CDCl_3$
1.03
1.00
0.98
1.07
0.78
1.08
1.12
1.00
2.08
3.04
2.12
0.99
2.07
3.17
3.19
3.23
3.07
f1 (ppm)

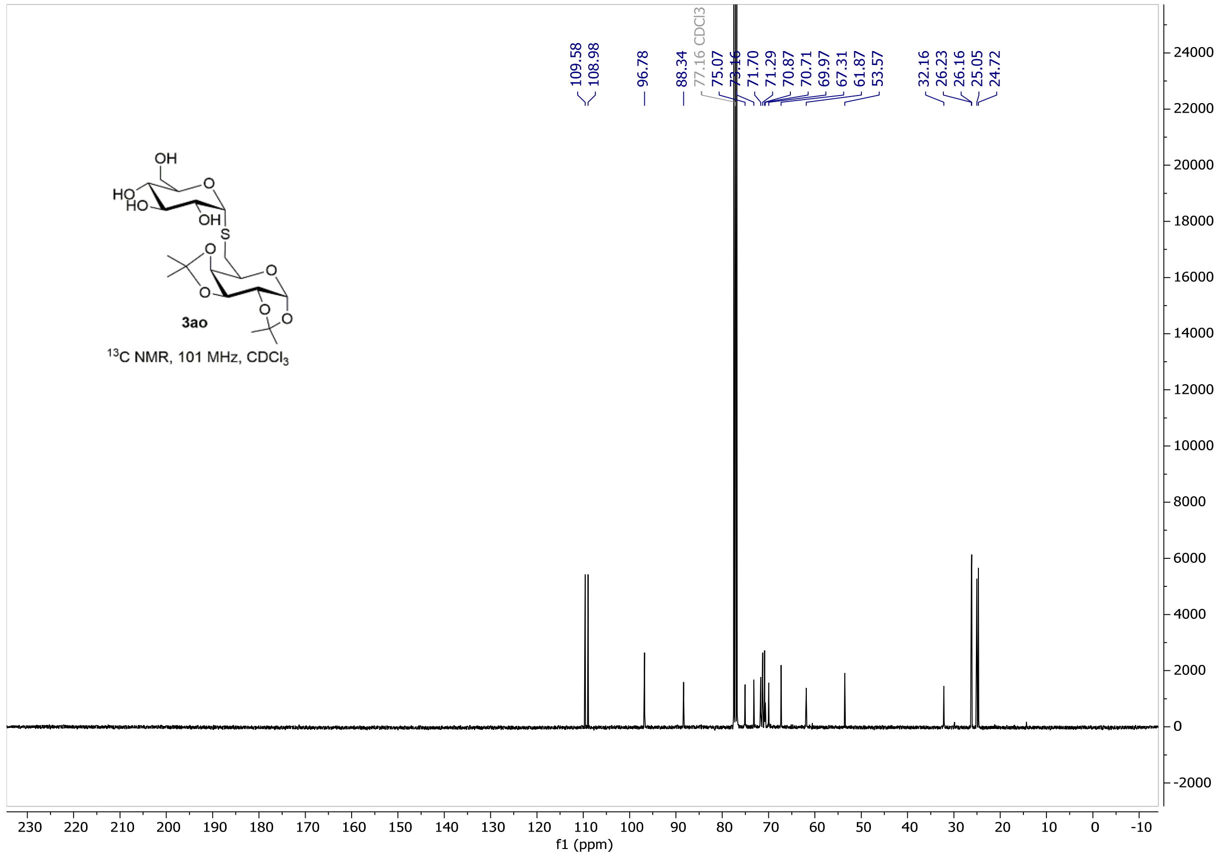

109.58
108.98
96.78
88.34
77.16 CDCl3
75.07
73.16
71.70
71.29
70.87
70.71
69.97
67.31
61.87
53.57
32.16
26.23
26.16
25.05
24.72
3ao
^{13}C NMR, 101 MHz, $CDCl_3$
f1 (ppm)

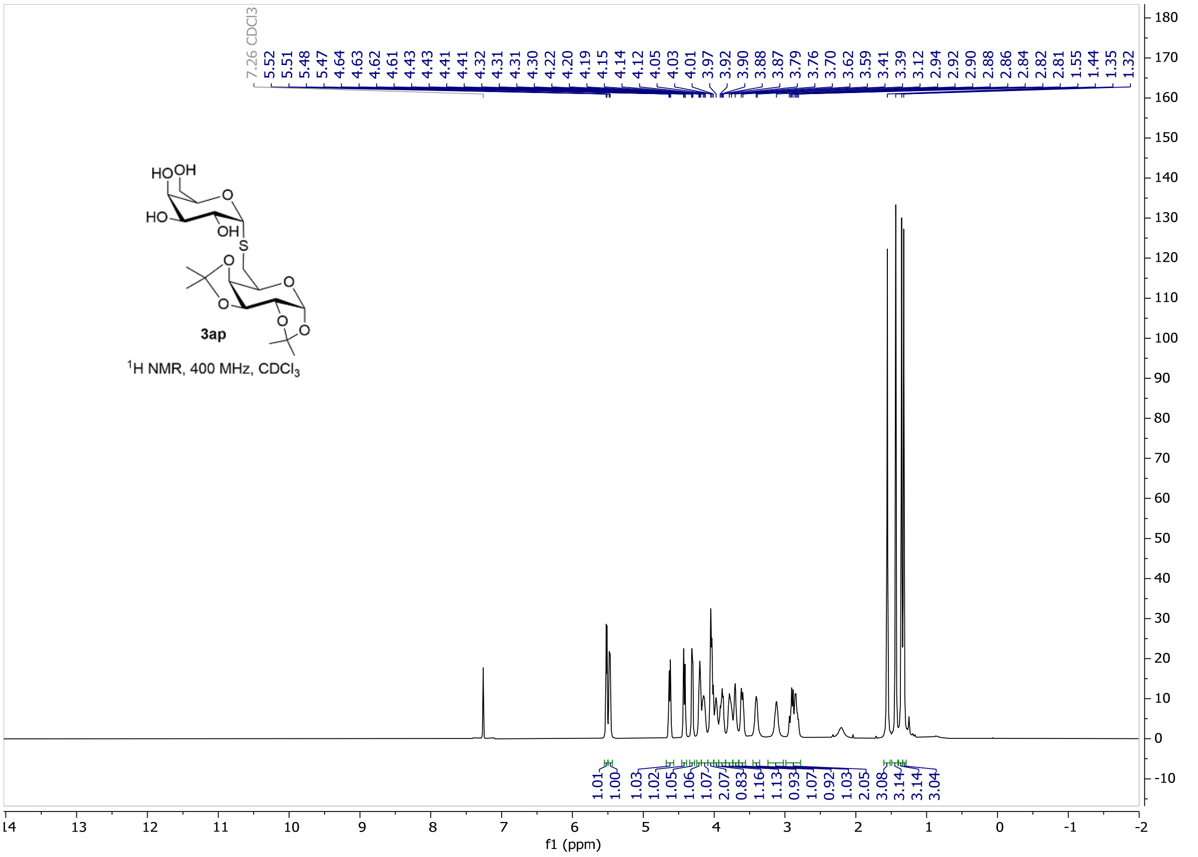
7.26 CDCl3
5.52
5.51
5.48
5.47
4.64
4.63
4.62
4.61
4.43
4.43
4.41
4.41
4.32
4.31
4.31
4.30
4.22
4.20
4.19
4.15
4.14
4.12
4.05
4.03
4.01
3.97
3.92
3.90
3.88
3.87
3.79
3.76
3.70
3.62
3.59
3.41
3.39
3.12
2.94
2.92
2.90
2.88
2.86
2.84
2.82
2.81
1.55
1.44
1.35
1.32
3ap
¹H NMR, 400 MHz, CDCl3
f1 (ppm)

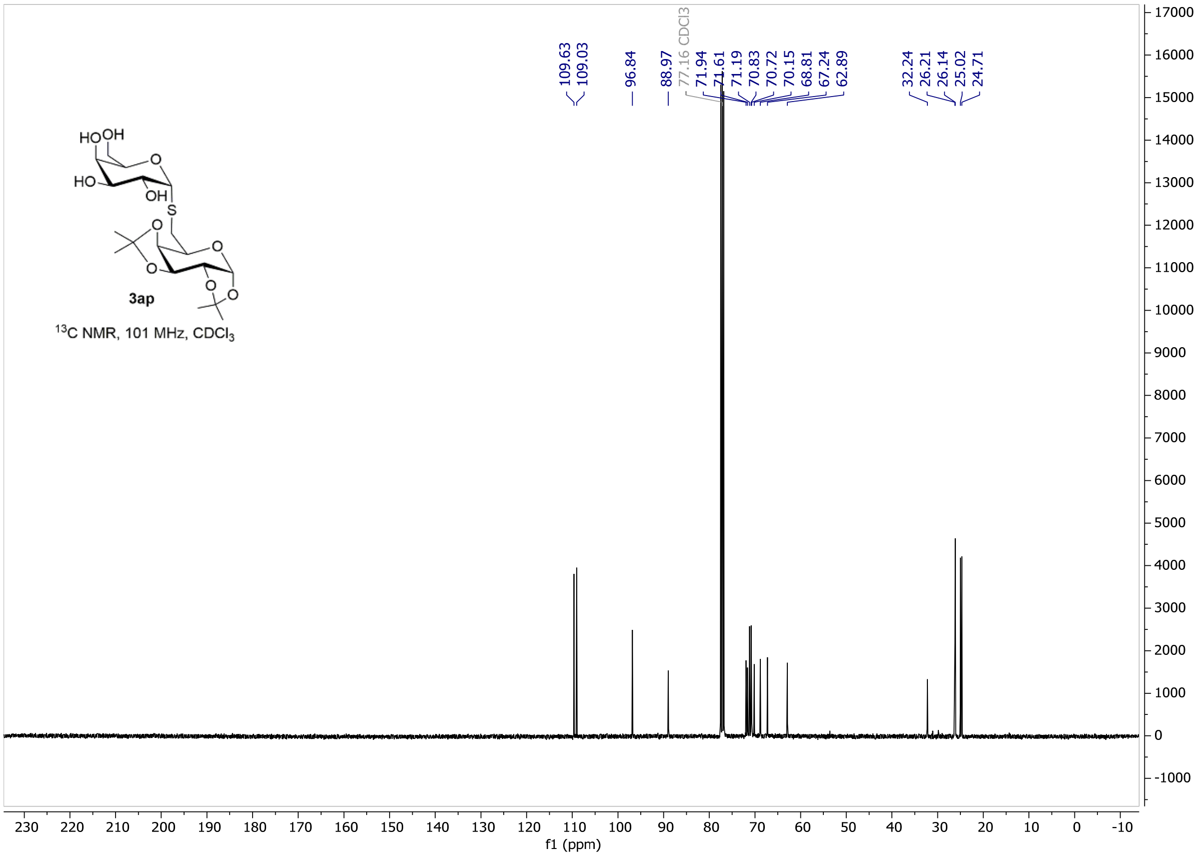
109.63
109.03
96.84
88.97
77.16 CDCl3
71.94
71.61
71.19
70.83
70.72
70.15
68.81
67.24
62.89
32.24
26.21
26.14
25.02
24.71
3ap
¹³C NMR, 101 MHz, CDCl3
f1 (ppm)

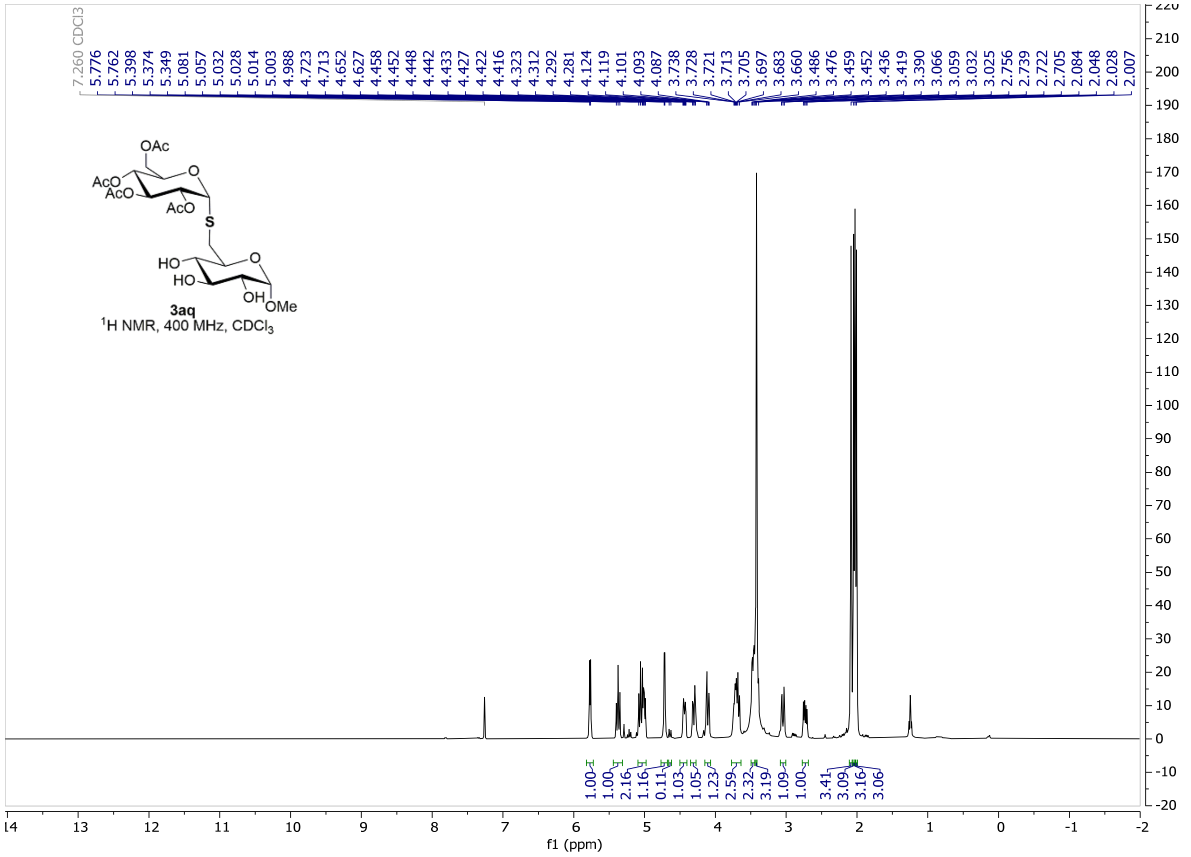
7.260 CDCl3
5.776
5.762
5.398
5.374
5.349
5.081
5.057
5.032
5.028
5.014
5.003
4.988
4.723
4.713
4.652
4.627
4.458
4.452
4.448
4.442
4.433
4.427
4.422
4.416
4.323
4.312
4.292
4.281
4.124
4.119
4.101
4.093
4.087
3.738
3.728
3.721
3.713
3.705
3.697
3.683
3.660
3.486
3.476
3.459
3.452
3.436
3.419
3.390
3.066
3.059
3.032
3.025
2.756
2.739
2.722
2.705
2.084
2.028
2.007
OAc
AcO
AcO
AcO
O
S
HO
HO
OH
O
OMe
3aq
^{1}H NMR, 400 MHz, CDCl$_3$
1.00
1.00
2.16
1.16
0.11
1.03
1.05
1.23
2.59
2.32
3.19
1.09
1.00
3.41
3.09
3.16
3.06
f1 (ppm)

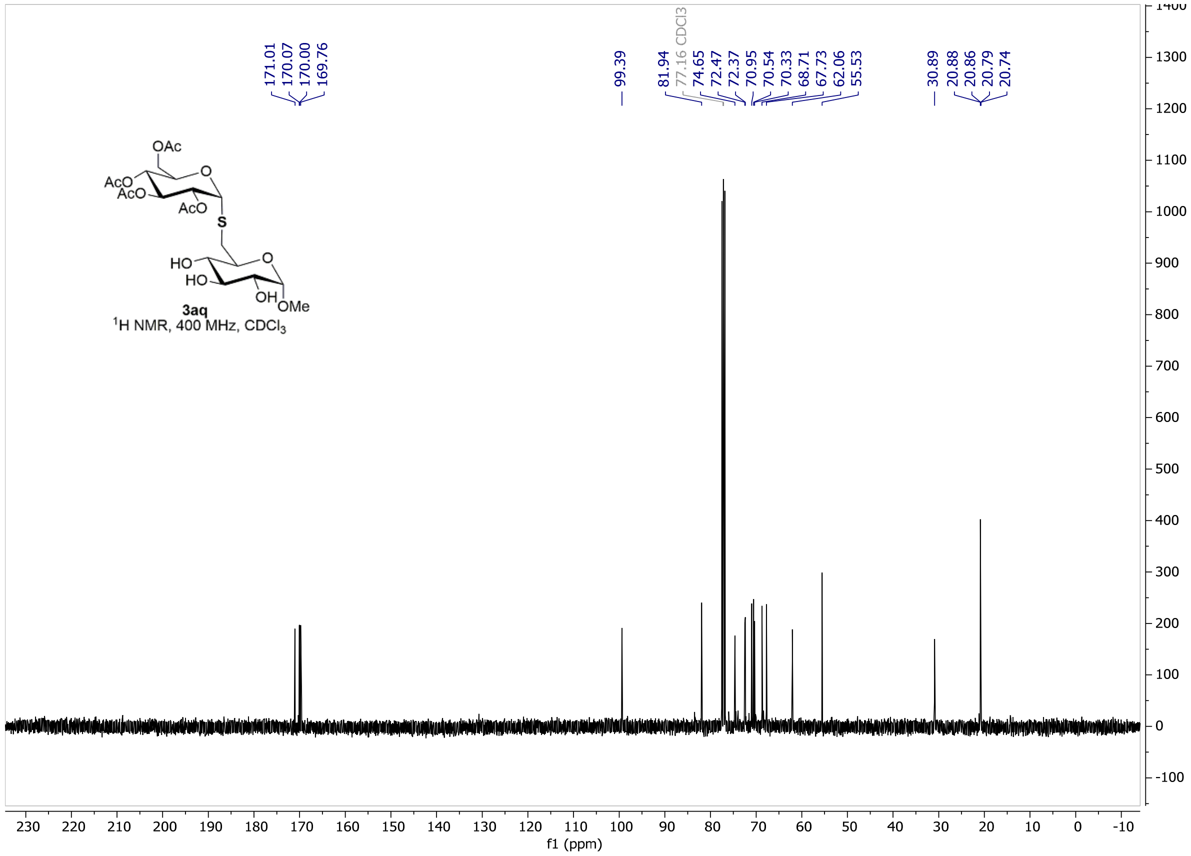
171.01
170.07
170.00
169.76
99.39
81.94
77.16 CDCl3
74.65
72.47
72.37
70.95
70.54
70.33
68.71
67.73
62.06
55.53
30.89
20.88
20.86
20.79
20.74
OAc
AcO
AcO
AcO
O
S
HO
HO
OH
O
OMe
3aq
^{1}H NMR, 400 MHz, CDCl$_3$
f1 (ppm)

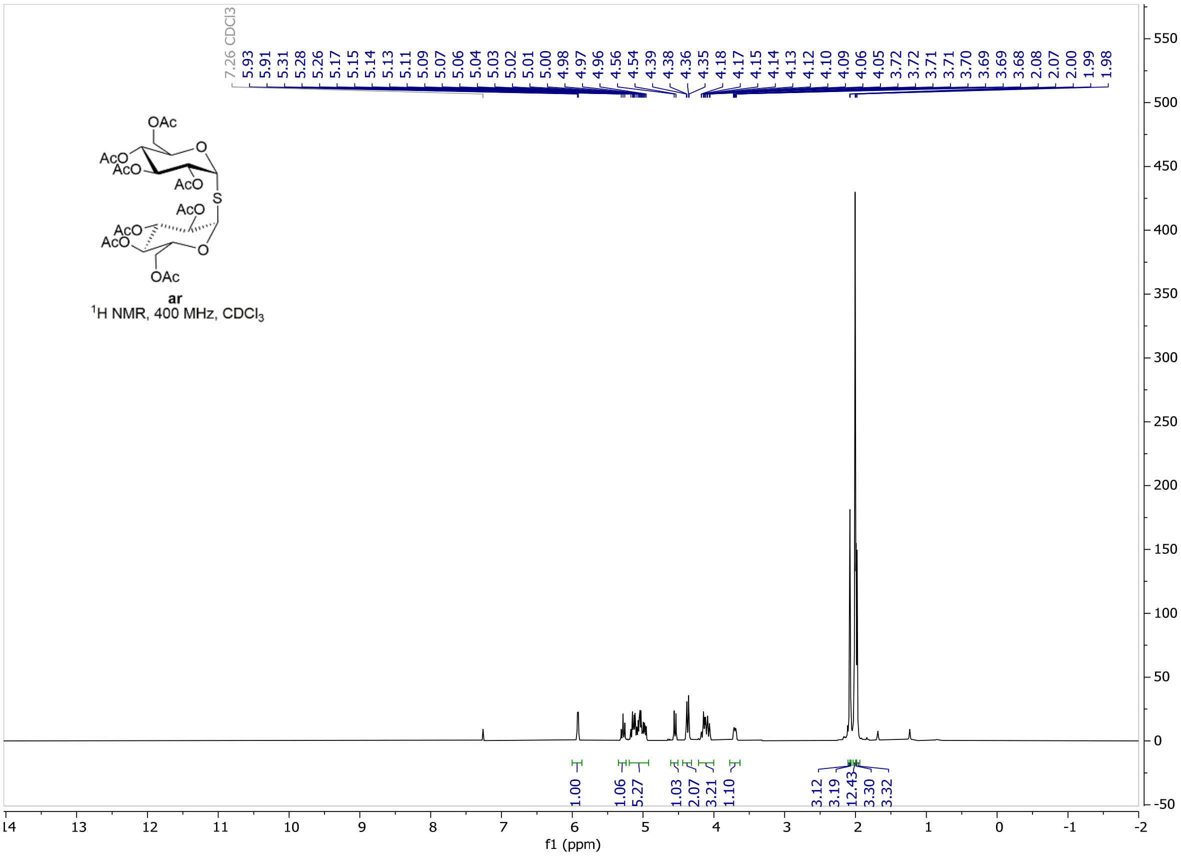
7.26 CDCl3
5.93
5.91
5.31
5.28
5.26
5.17
5.15
5.14
5.13
5.11
5.09
5.07
5.06
5.04
5.03
5.02
5.01
5.00
4.98
4.97
4.96
4.56
4.54
4.39
4.38
4.36
4.35
4.18
4.17
4.15
4.14
4.13
4.12
4.10
4.09
4.06
4.05
3.72
3.72
3.71
3.71
3.70
3.69
3.69
3.68
2.08
2.07
2.00
1.99
1.98
OAc
AcO
AcO
O
AcO
S
AcO
AcO
AcO
O
OAc
ar
1H NMR, 400 MHz, CDCl3
1.00
1.06
5.27
1.03
2.07
3.21
1.10
3.12
3.19
12.43
3.30
3.32
f1 (ppm)

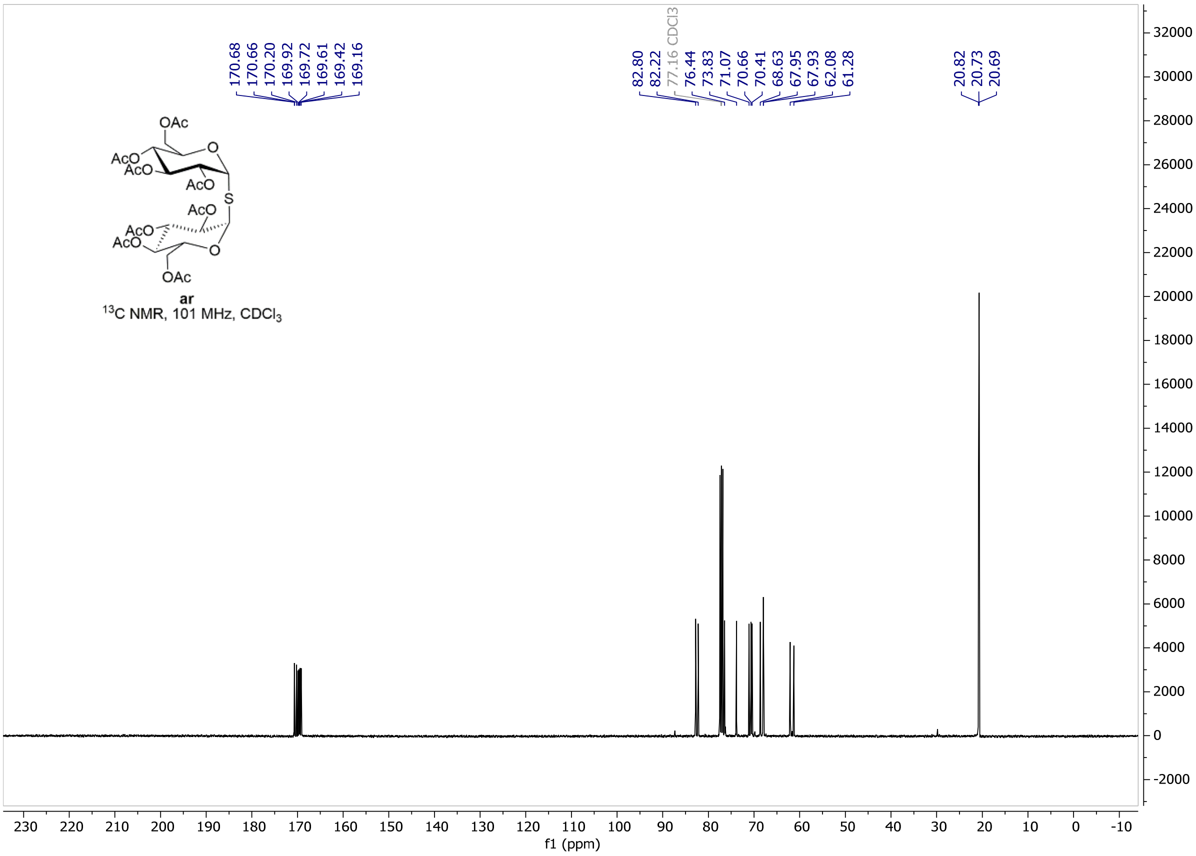
170.68
170.66
170.20
169.92
169.72
169.61
169.42
169.16
82.80
82.22
77.16 CDCl3
76.44
73.83
71.07
70.66
70.41
68.63
67.95
67.93
62.08
61.28
20.82
20.73
20.69
OAc
AcO
AcO
O
AcO
S
AcO
AcO
AcO
O
OAc
ar
13C NMR, 101 MHz, CDCl3
f1 (ppm)

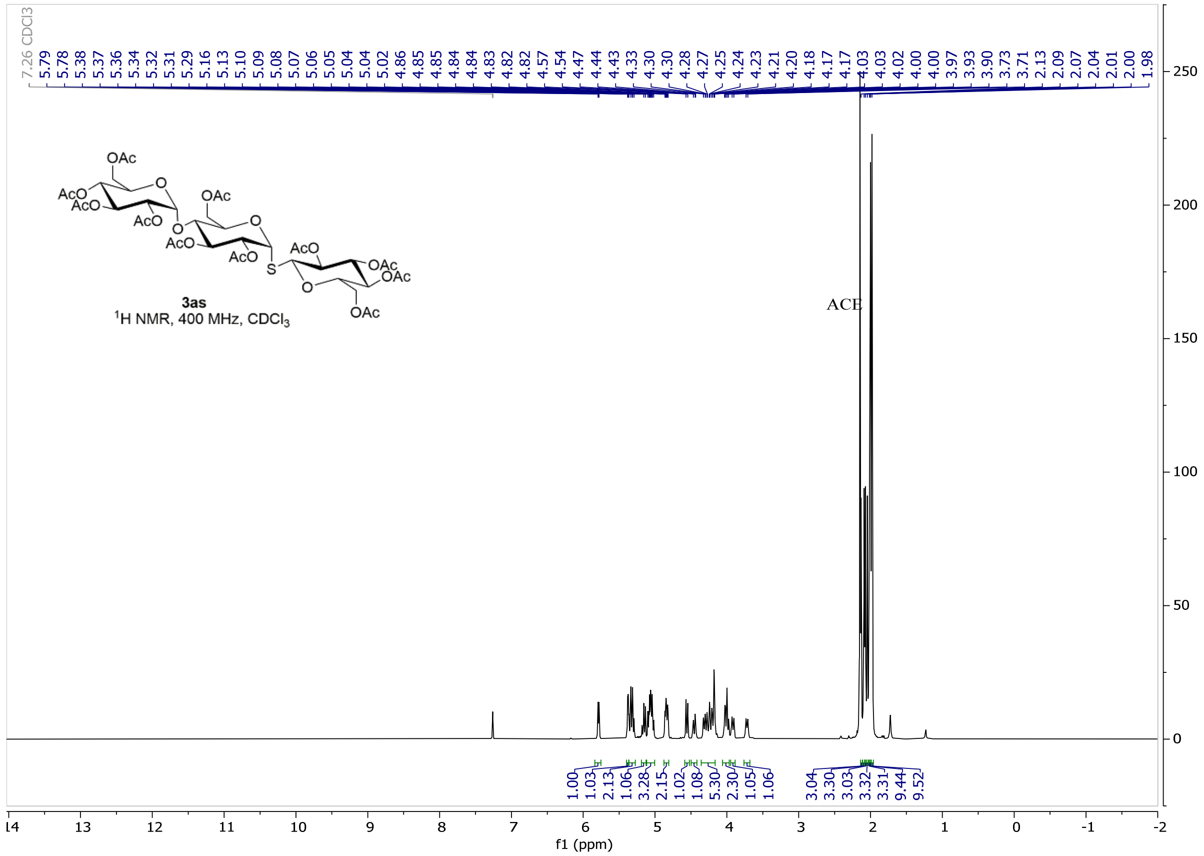
7.26 CDCl3
5.79 5.78 5.38 5.37 5.36 5.34 5.32 5.31 5.29 5.16 5.13 5.10 5.09 5.08 5.07 5.06 5.05 5.04 5.04 5.02 4.86 4.85 4.85 4.84 4.84 4.83 4.82 4.82 4.57 4.54 4.47 4.44 4.43 4.33 4.30 4.30 4.28 4.27 4.25 4.24 4.23 4.21 4.20 4.18 4.17 4.17 4.03 4.03 4.03 4.02 4.00 4.00 3.97 3.93 3.90 3.73 3.71 2.13 2.09 2.07 2.04 2.01 2.00 1.98
3as
1H NMR, 400 MHz, CDCl3
ACE
1.00 1.03 2.13 1.06 3.28 2.15 1.02 1.08 5.30 2.30 1.05 1.06 3.04 3.30 3.03 3.32 3.31 9.44 9.52
f1 (ppm)

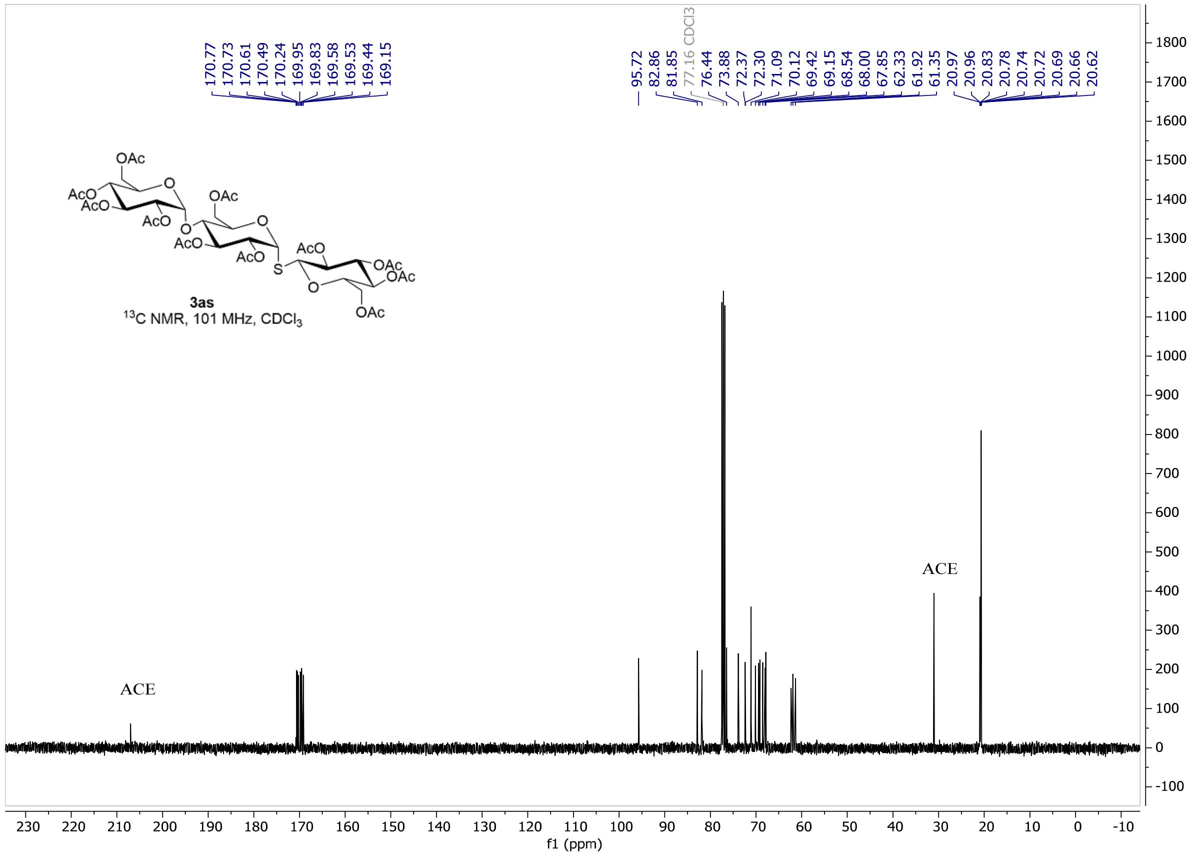
170.77 170.73 170.61 170.49 170.24 169.95 169.83 169.58 169.53 169.44 169.15
95.72 82.86 81.85 77.16 CDCl3 76.44 73.88 72.37 72.30 71.09 70.12 69.42 69.15 68.54 68.00 67.85 62.33 61.92 61.35 20.97 20.96 20.83 20.78 20.74 20.72 20.69 20.66 20.62
3as
13C NMR, 101 MHz, CDCl3
ACE
ACE
f1 (ppm)

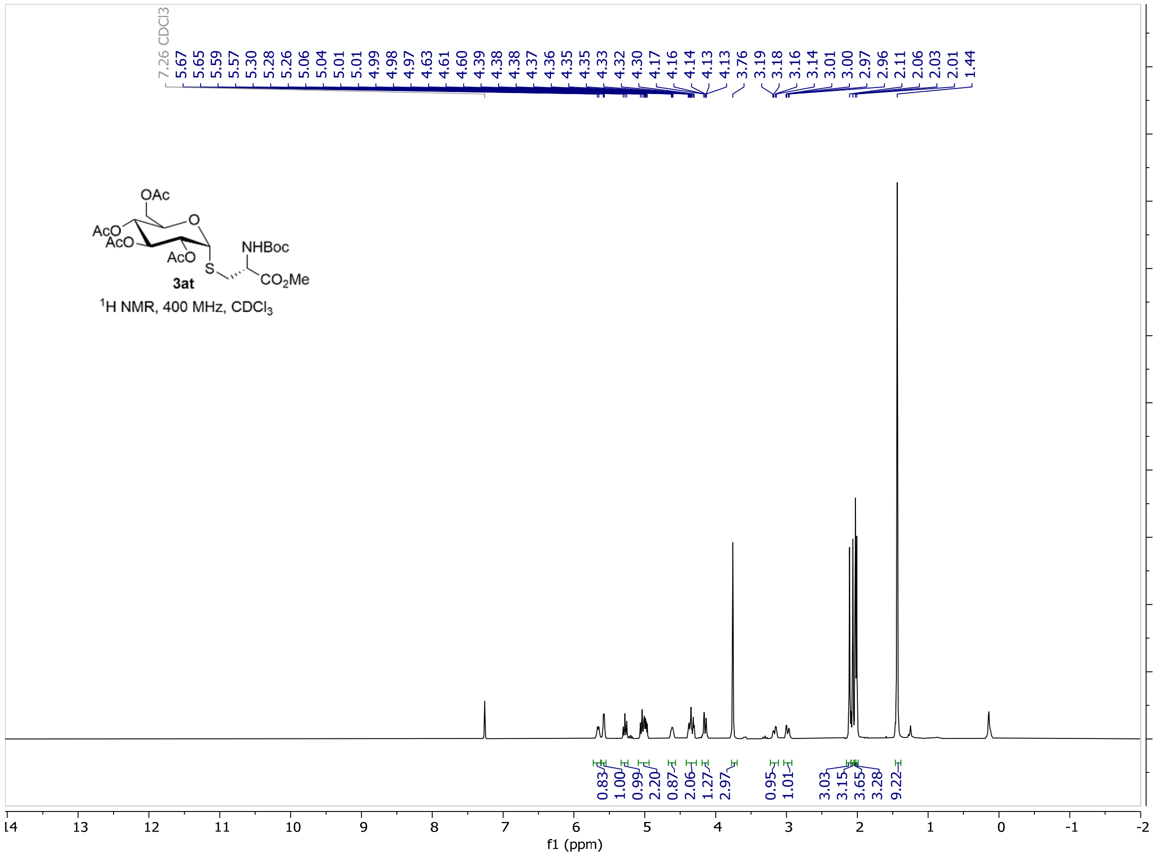

7.26 CDCl3
5.67
5.65
5.59
5.57
5.30
5.28
5.26
5.06
5.04
5.01
5.01
4.99
4.98
4.97
4.63
4.61
4.60
4.39
4.38
4.38
4.37
4.36
4.35
4.35
4.33
4.32
4.30
4.17
4.16
4.14
4.13
4.13
3.76
3.19
3.18
3.16
3.14
3.01
3.00
2.97
2.96
2.11
2.06
2.03
2.01
1.44
OAc
AcO
AcO
O
AcO
NHBoc
S
CO2Me
3at
1H NMR, 400 MHz, CDCl3
f1 (ppm)

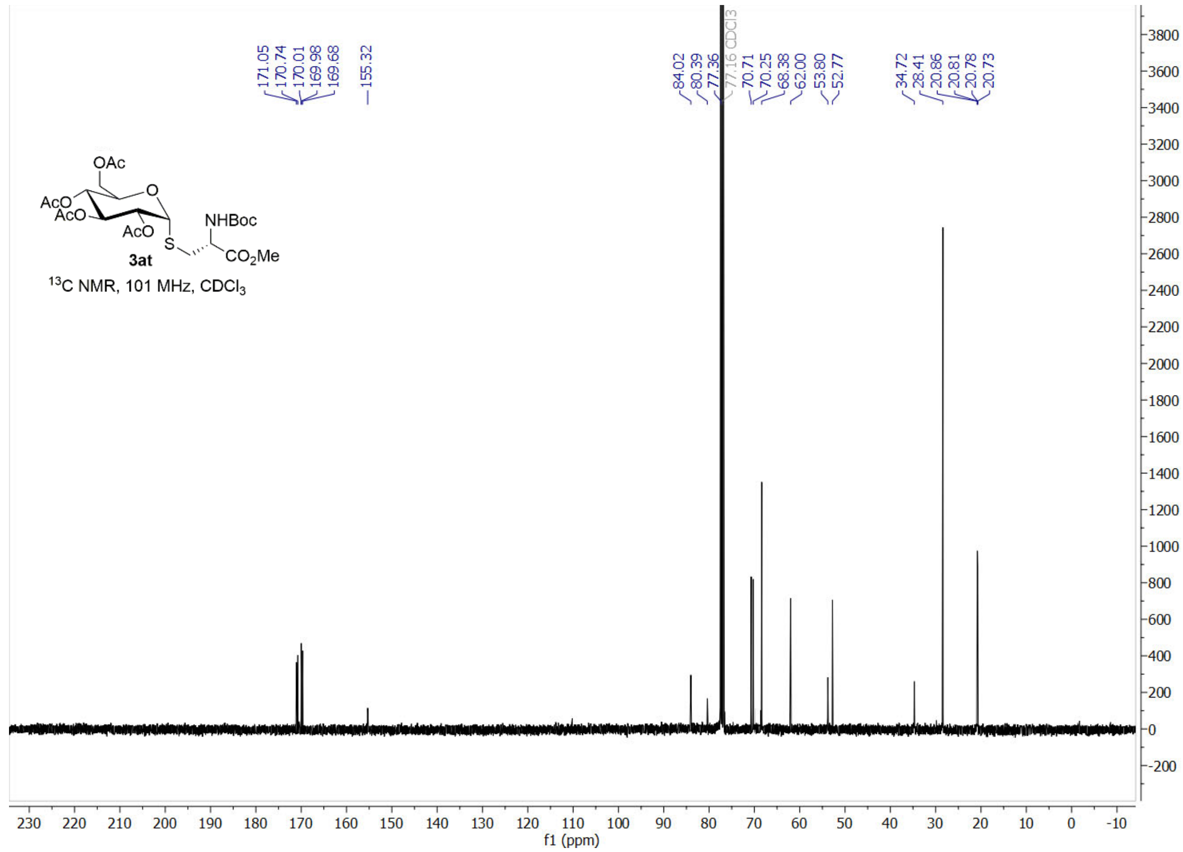

171.05
170.74
170.01
169.98
169.68
155.32
84.02
80.39
77.36
77.16 CDCl3
70.71
70.25
68.38
62.00
53.80
52.77
34.72
28.41
20.86
20.81
20.78
20.73
OAc
AcO
AcO
O
AcO
NHBoc
S
CO2Me
3at
13C NMR, 101 MHz, CDCl3
f1 (ppm)

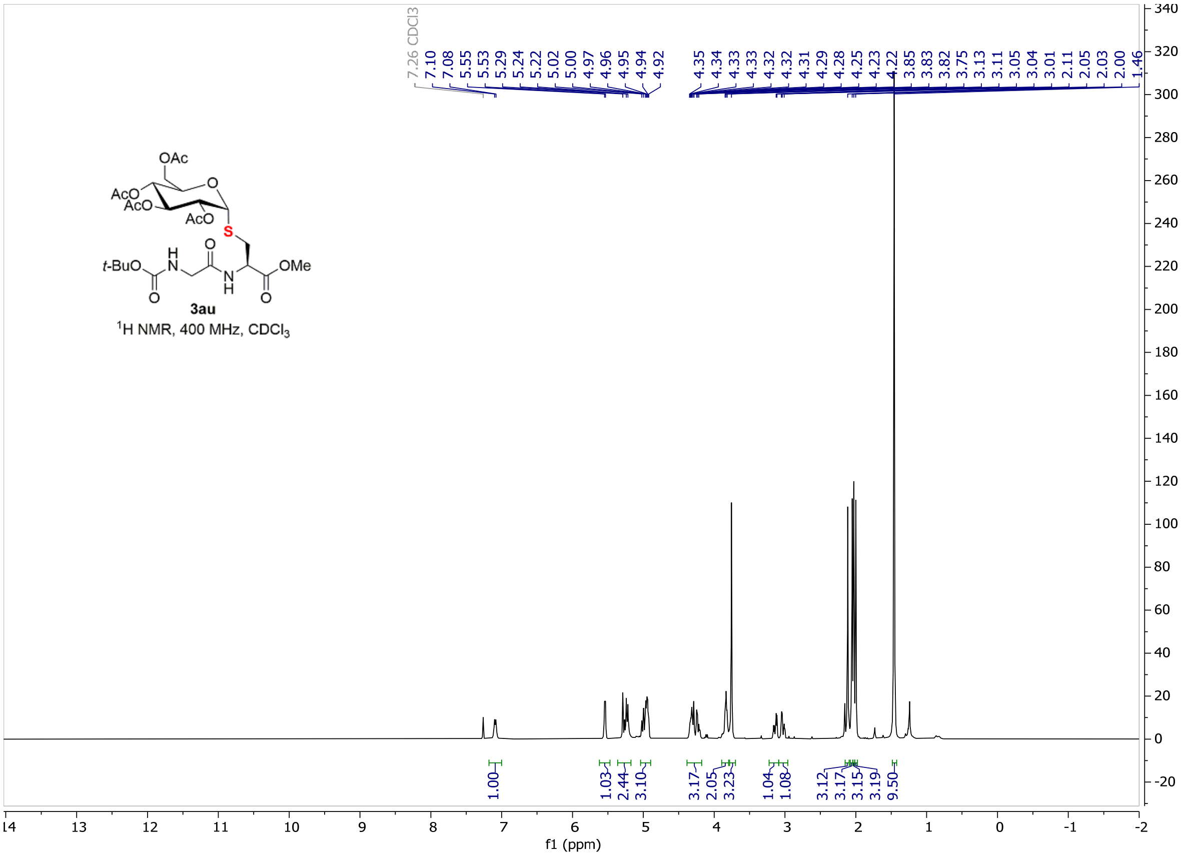
7.26 CDCl3
7.10
7.08
5.55
5.53
5.29
5.24
5.22
5.02
5.00
4.97
4.96
4.95
4.94
4.92
4.35
4.34
4.33
4.33
4.32
4.32
4.31
4.29
4.28
4.25
4.23
4.22
3.85
3.83
3.82
3.75
3.13
3.11
3.05
3.04
3.01
2.11
2.05
2.03
2.00
1.46
OAc
AcO
AcO
AcO
O
S
t-BuO
H
N
O
N
H
OMe
O
O
3au
1H NMR, 400 MHz, CDCl3
1.00
1.03
2.44
3.10
3.17
2.05
3.23
1.04
1.08
3.12
3.17
3.15
3.19
9.50
f1 (ppm)

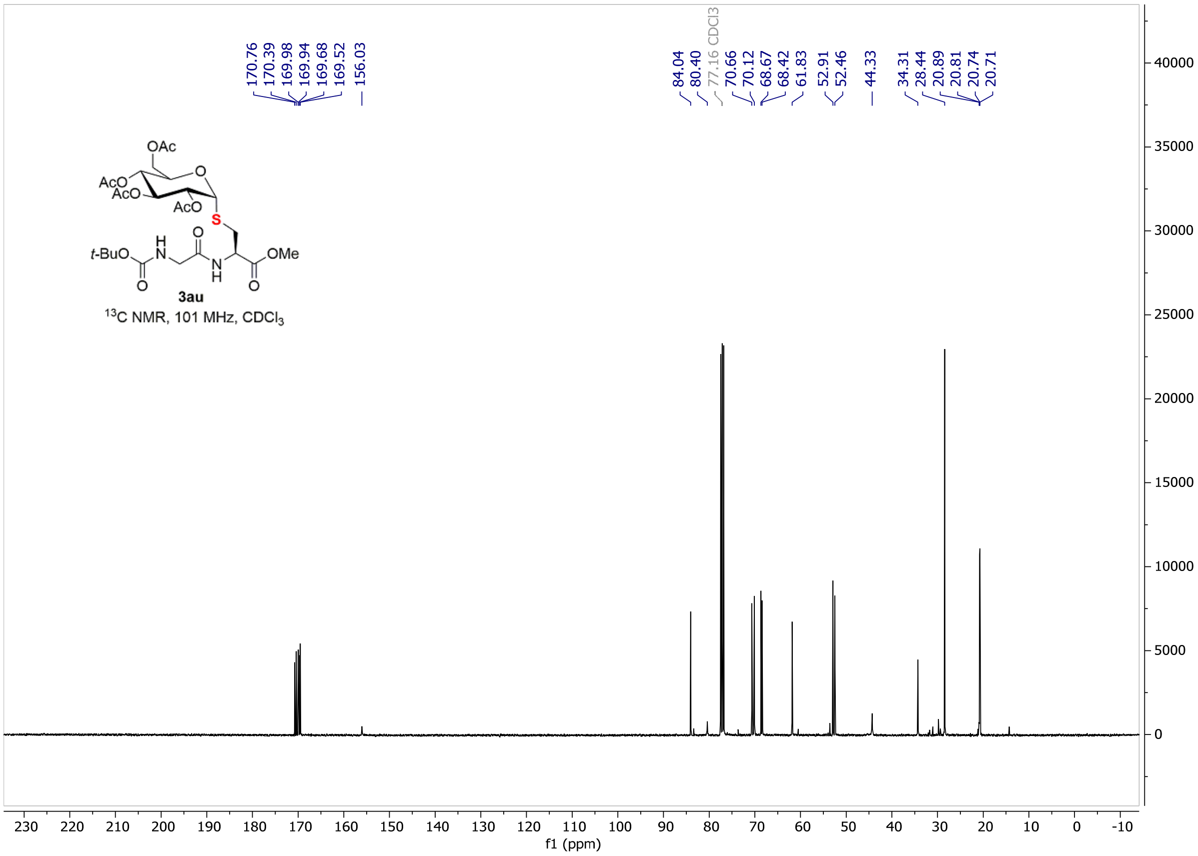
170.76
170.39
169.98
169.94
169.68
169.52
156.03
84.04
80.40
77.16 CDCl3
70.66
70.12
68.67
68.42
61.83
52.91
52.46
44.33
34.31
28.44
20.89
20.81
20.74
20.71
OAc
AcO
AcO
AcO
O
S
t-BuO
H
N
O
N
H
OMe
O
O
3au
13C NMR, 101 MHz, CDCl3
f1 (ppm)

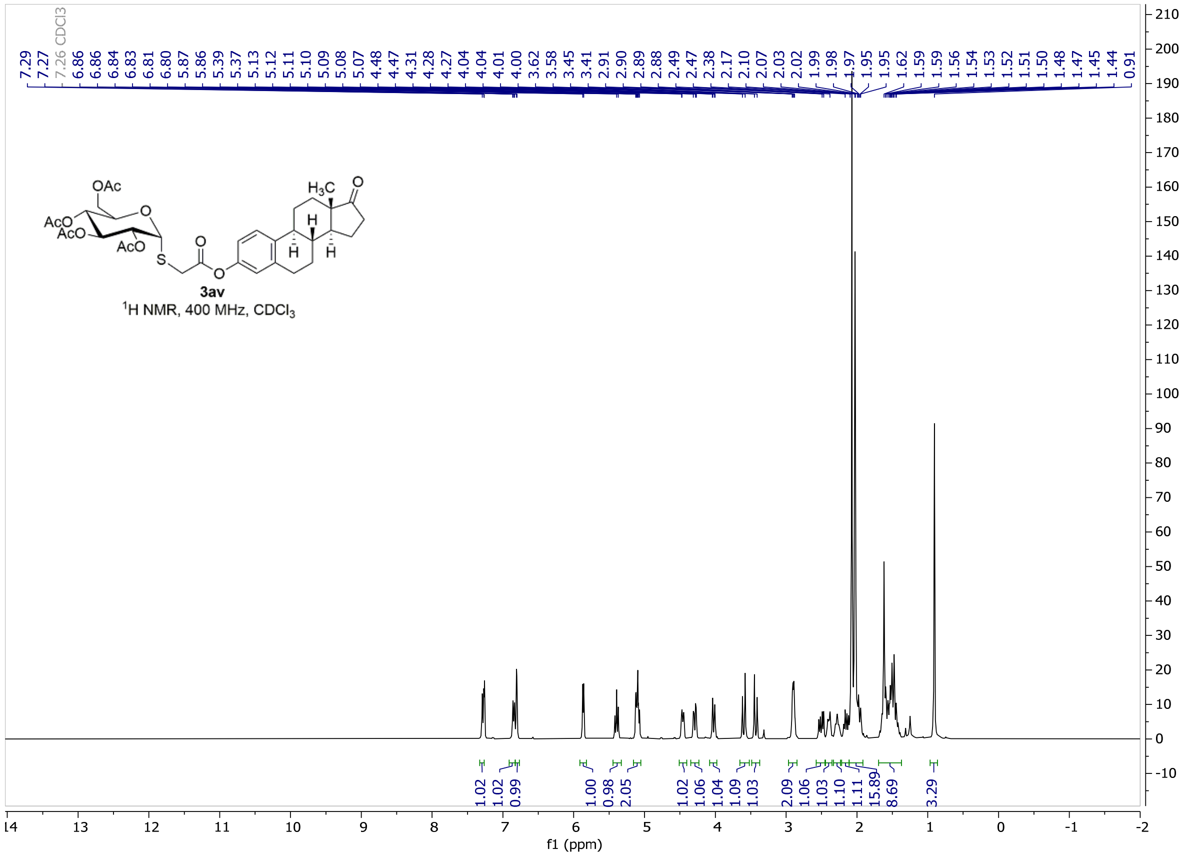

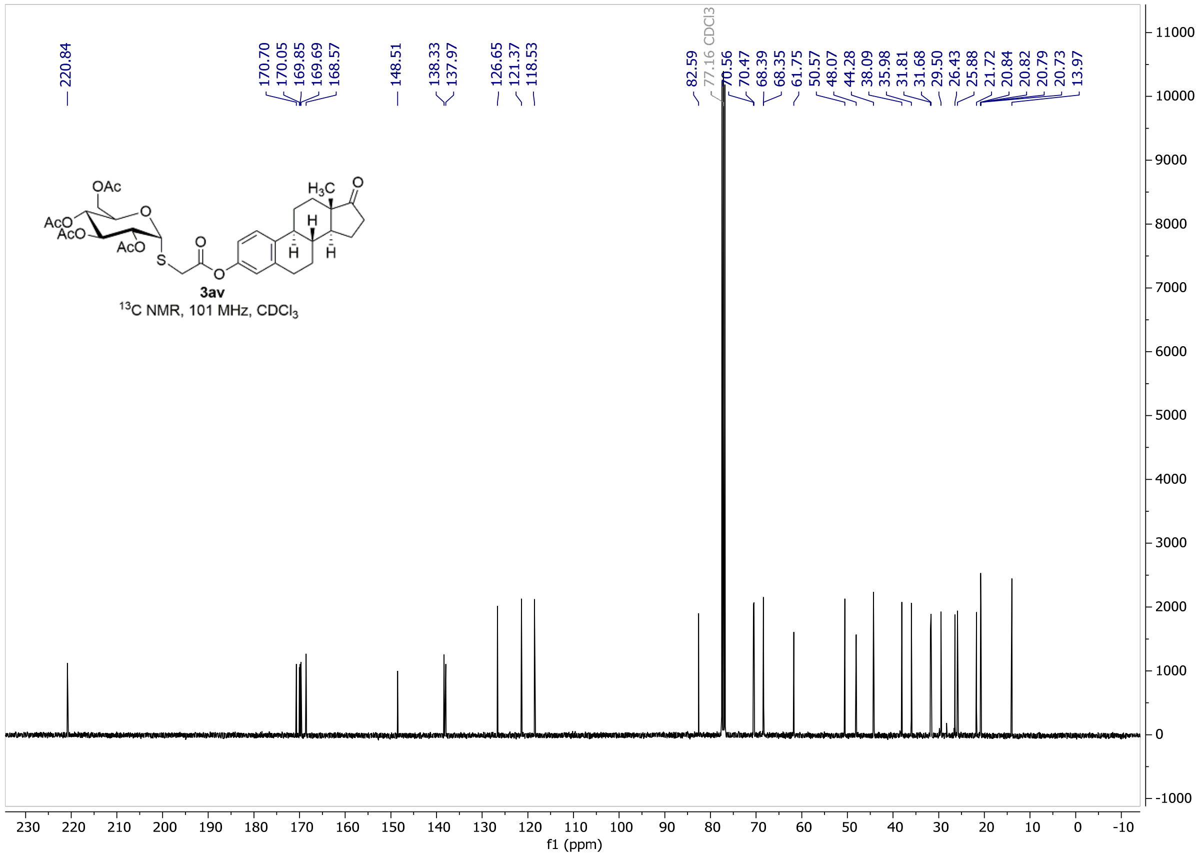

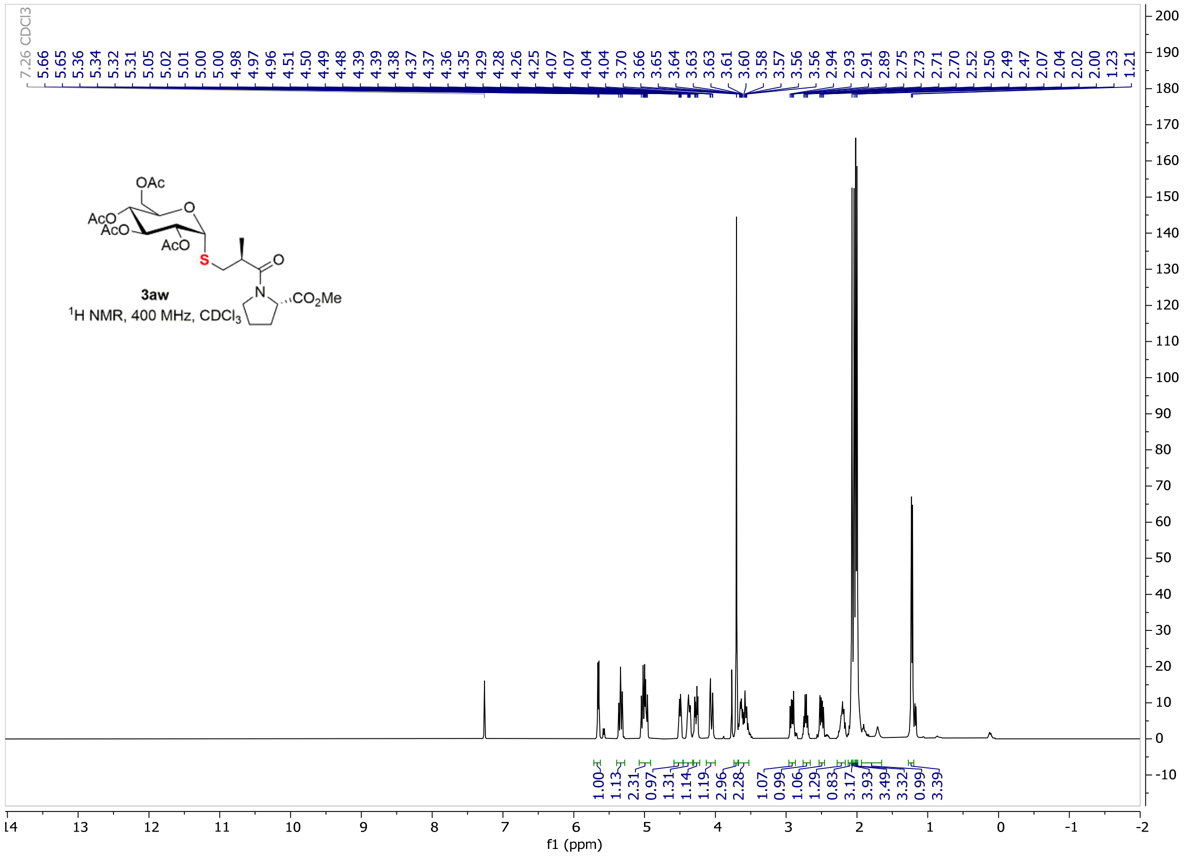
3aw
1H NMR, 400 MHz, CDCl3
f1 (ppm)

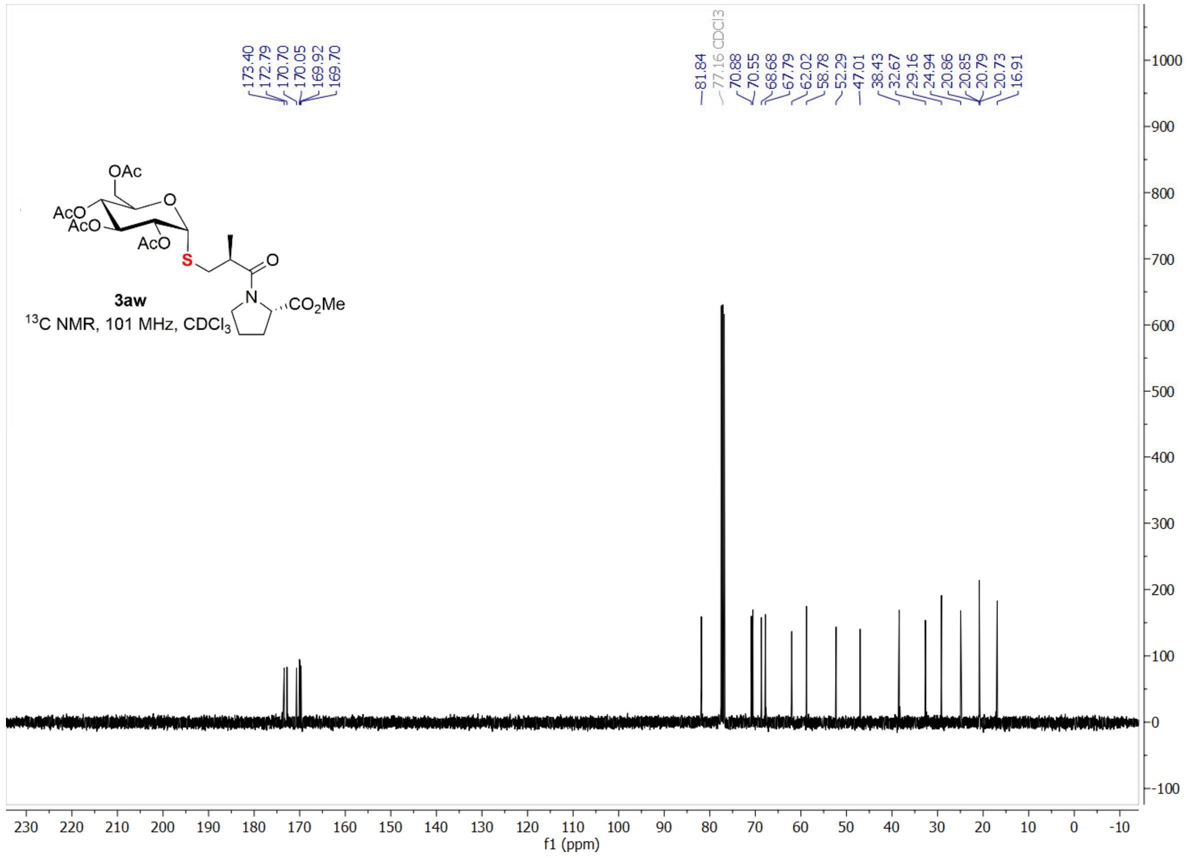
3aw
13C NMR, 101 MHz, CDCl3
f1 (ppm)

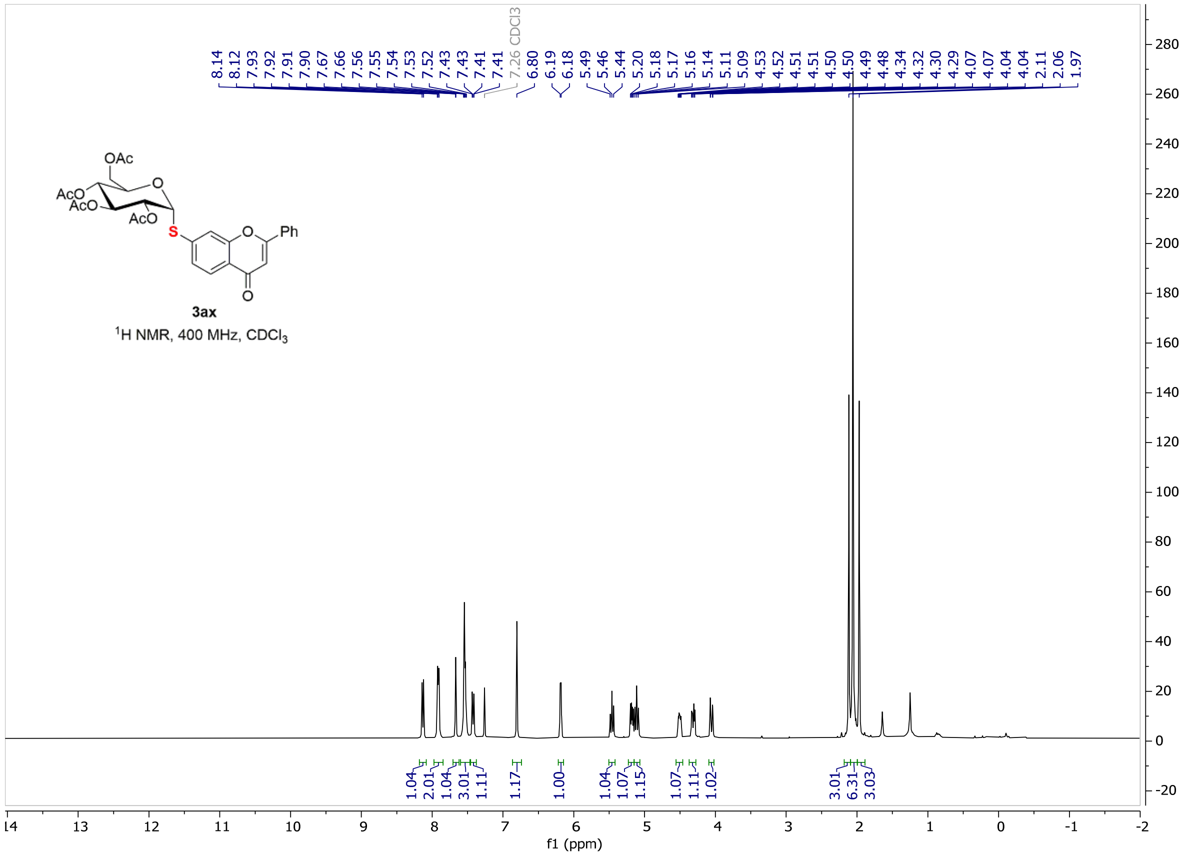

8.14
8.12
7.93
7.92
7.91
7.90
7.67
7.66
7.56
7.55
7.54
7.53
7.52
7.43
7.43
7.41
7.41
7.26 CDCl3
6.80
6.19
6.18
5.49
5.46
5.44
5.20
5.18
5.17
5.16
5.14
5.11
5.09
4.53
4.52
4.51
4.50
4.50
4.49
4.48
4.34
4.32
4.30
4.29
4.07
4.07
4.04
4.04
2.11
2.06
1.97
OAc
AcO
AcO
AcO
S
O
Ph
O
3ax
1H NMR, 400 MHz, CDCl3
1.04
2.01
1.04
3.01
1.11
1.17
1.00
1.04
1.07
1.15
1.07
1.11
1.02
3.01
6.31
3.03
f1 (ppm)

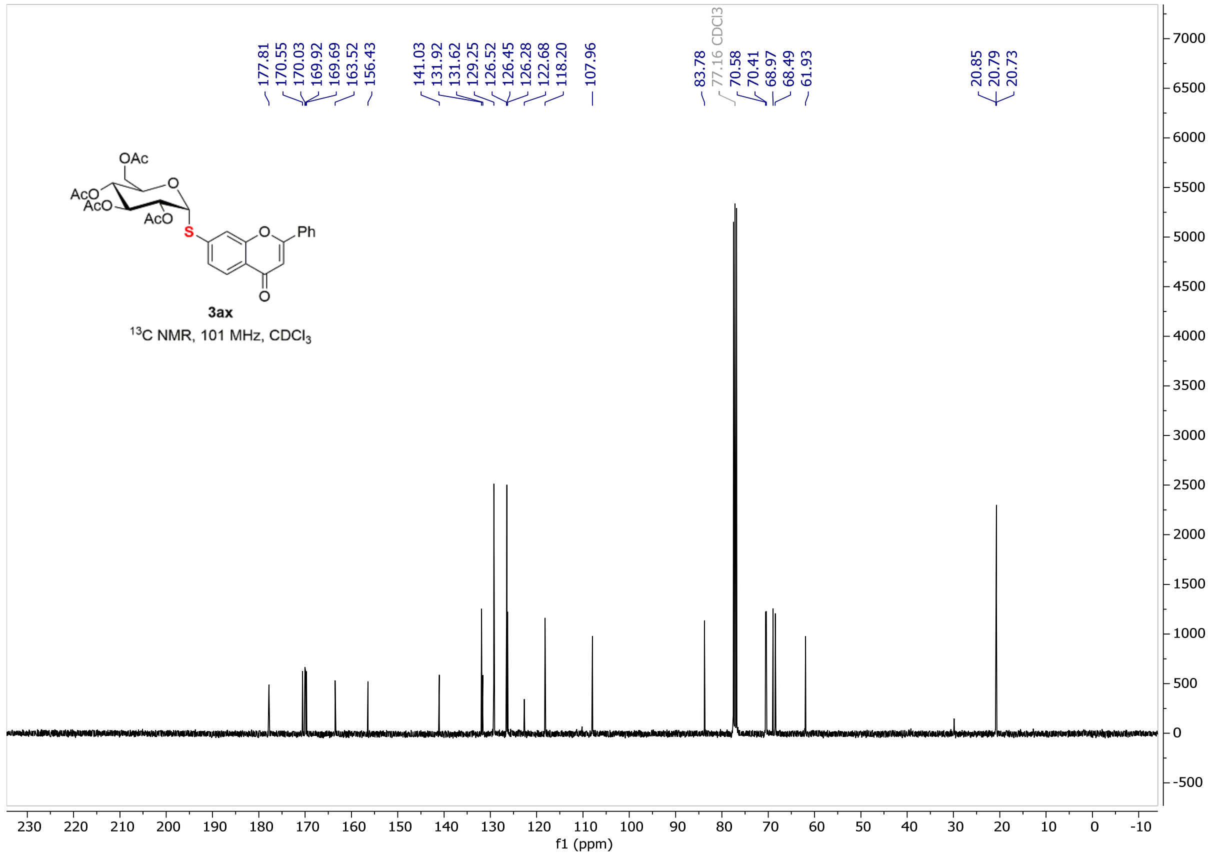

177.81
170.55
170.03
169.92
169.69
163.52
156.43
141.03
131.92
131.62
129.25
126.52
126.45
126.28
122.68
118.20
107.96
83.78
77.16 CDCl3
70.58
70.41
68.97
68.49
61.93
20.85
20.79
20.73
OAc
AcO
AcO
AcO
S
O
Ph
O
3ax
13C NMR, 101 MHz, CDCl3
f1 (ppm)

11. HRMS Spectra of 4a and 11

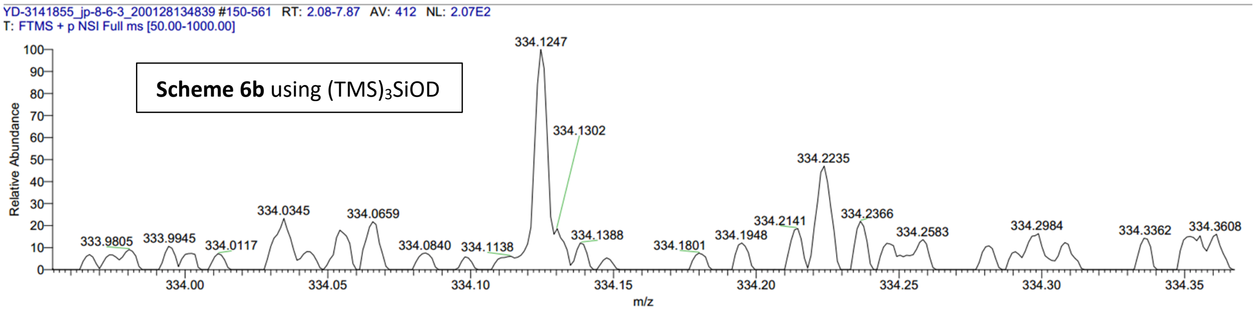

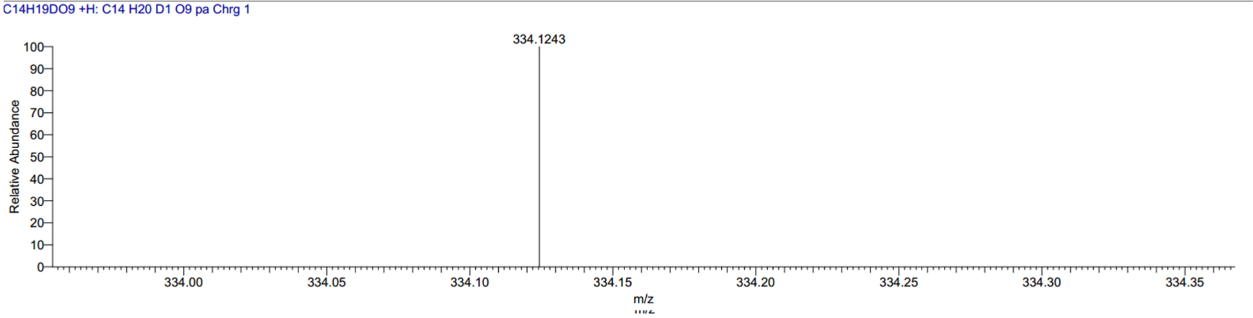

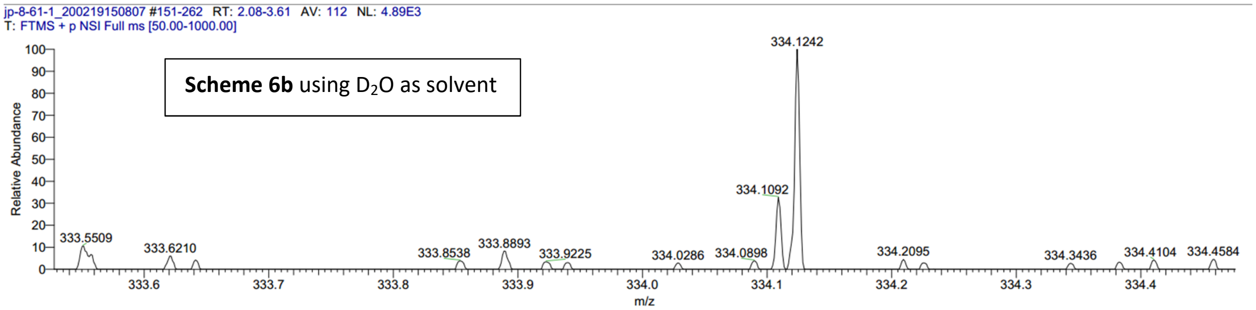

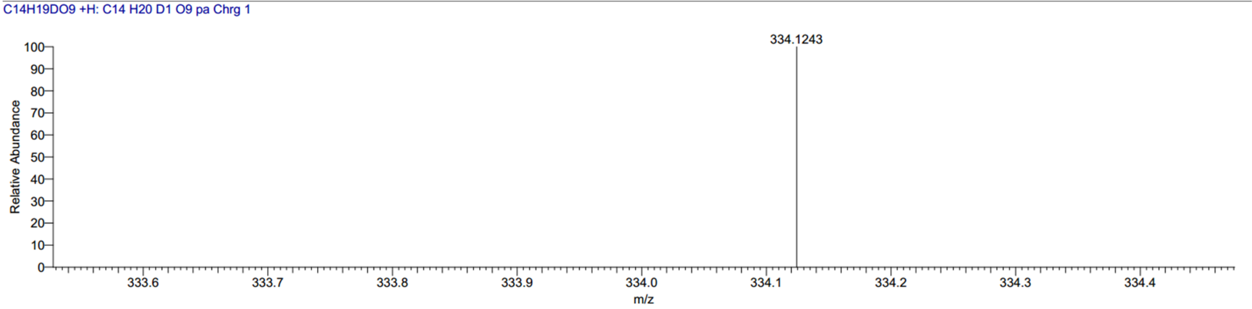

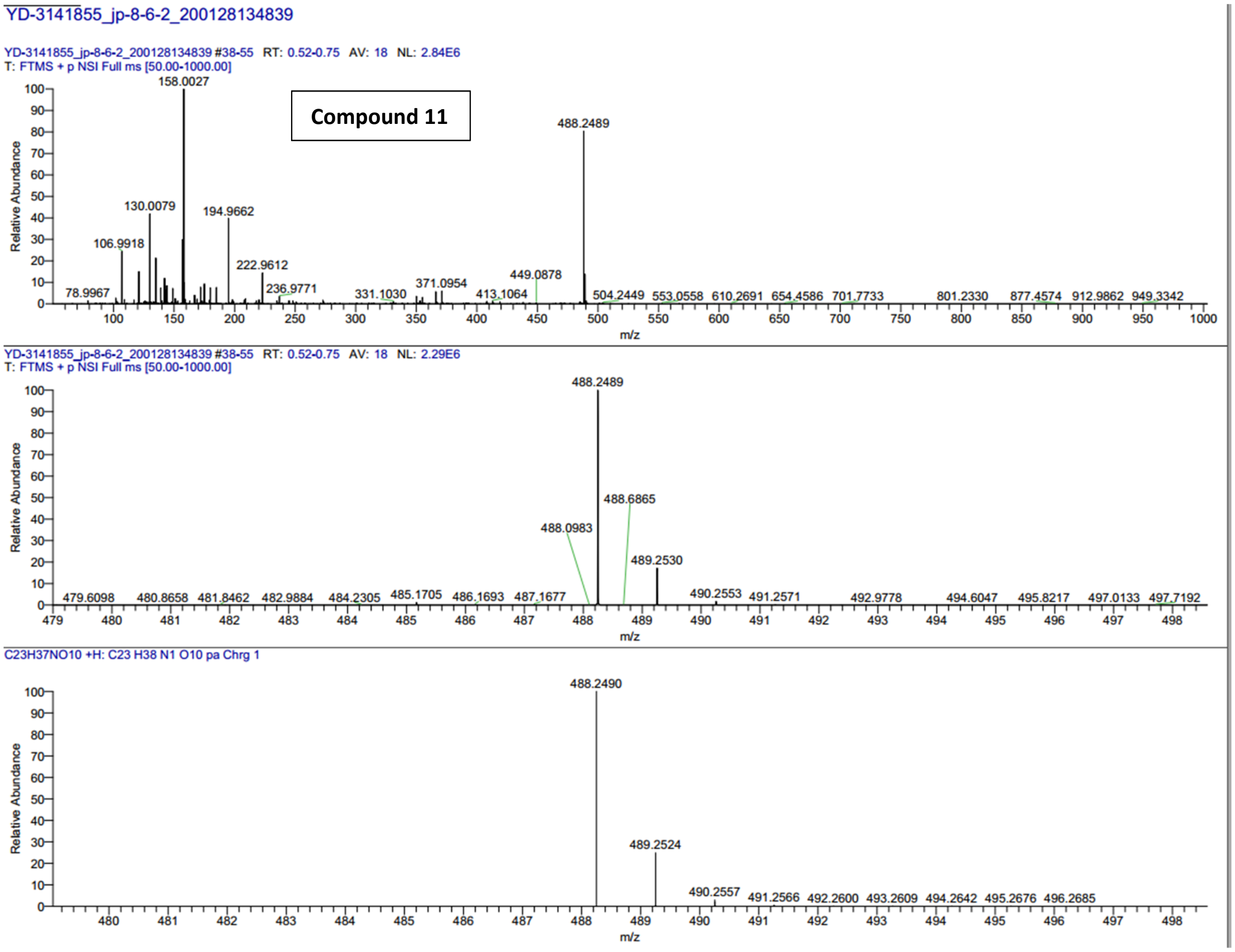
YD-3141855_jp-8-6-2_200128134839
YD-3141855_jp-8-6-2_200128134839 #38-55 RT: 0.52-0.75 AV: 18 NL: 2.84E6
T: FTMS + p NSI Full ms [50.00-1000.00]
Compound 11
158.0027
488.2489
130.0079
194.9662
106.9918
222.9612
78.9967
236.9771
331.1030
371.0954
413.1064
449.0878
504.2449
553.0558
610.2691
654.4586
701.7733
801.2330
877.4574
912.9862
949.3342
Relative Abundance
m/z
YD-3141855_jp-8-6-2_200128134839 #38-55 RT: 0.52-0.75 AV: 18 NL: 2.29E6
T: FTMS + p NSI Full ms [50.00-1000.00]
488.2489
488.6865
488.0983
489.2530
479.6098
480.8658
481.8462
482.9884
484.2305
485.1705
486.1693
487.1677
490.2553
491.2571
492.9778
494.6047
495.8217
497.0133
497.7192
C23H37NO10 +H: C23 H38 N1 O10 pa Chrg 1
488.2490
489.2524
490.2557
491.2566
492.2600
493.2609
494.2642
495.2676
496.2685
